# Pyrazole-Based Thrombin Inhibitors with a Serine-Trapping Mechanism of Action: Synthesis and Biological Activity

**DOI:** 10.3390/ph15111340

**Published:** 2022-10-28

**Authors:** Calvin Dunker, Lukas Imberg, Alena I. Siutkina, Catharina Erbacher, Constantin G. Daniliuc, Uwe Karst, Dmitrii V. Kalinin

**Affiliations:** 1 Institute of Pharmaceutical and Medicinal Chemistry, University of Münster, 48149 Münster, Germany; 2 Institute of Inorganic and Analytical Chemistry, University of Münster, 48149 Münster, Germany; 3 Institute for Organic Chemistry, University of Münster, 48149 Münster, Germany

**Keywords:** covalent inhibitor, thrombin, thrombosis, anticoagulants, pyrazole, Ullmann reaction, dabigatran, pyrazolo[5,1-*b*]quinazolin-9(4*H*)-ones, pyrazolo[1,5-*a*]quinazolin-5(4*H*)-ones

## Abstract

New antithrombotic drugs are needed to combat thrombosis, a dangerous pathology that causes myocardial infarction and ischemic stroke. In this respect, thrombin (FIIa) represents an important drug target. We herein report the synthesis and biological activity of a series of 1*H*-pyrazol-5-amine-based thrombin inhibitors with a serine-trapping mechanism of action. Among synthesized compounds, flexible acylated 1*H*-pyrazol-5-amines **24e**, **34a**, and **34b** were identified as potent 16–80 nM thrombin inhibitors, which showed practically no off-targeting effect against other physiologically relevant serine proteases. To prove that synthesized compounds are covalent thrombin inhibitors, the most potent derivative **24e** (FIIa IC_50_ = 16 nM) was studied in a mass-shift assay, where it has been shown that **24e** transfers its acyl moiety (pivaloyl) to the catalytic Ser195 of thrombin. Performed herein docking studies also confirmed the covalent mechanism of thrombin inhibition by synthesized compounds. Acylated aminopyrazoles found during this study showed only limited effects on plasma coagulation in activated partial thrombin time (aPTT) and prothrombin time (PT) in vitro assays. However, such thrombin inhibitors are expected to have virtually no effect on bleeding time and can be used as a starting point for developing a safer alternative to traditional non-covalent anticoagulants.

## 1. Introduction

**Figure 1:**
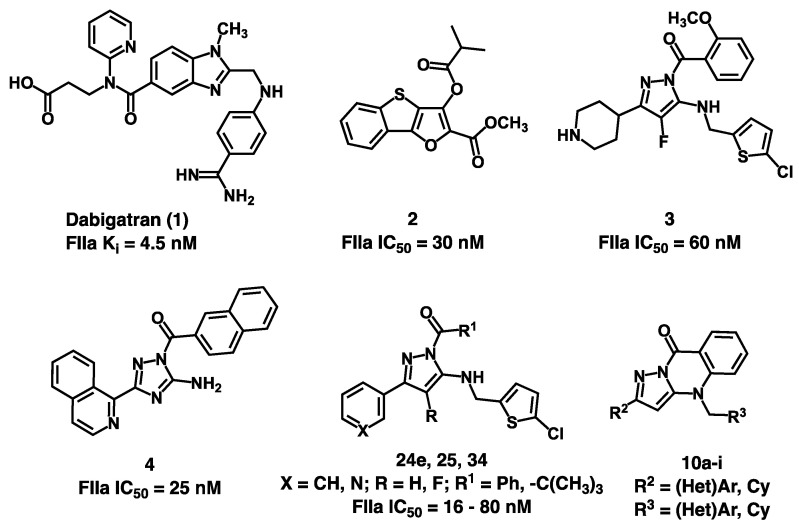
Exemplary structures of small molecule thrombin (FIIa) inhibitors. Dabigatran (**1**) [4] is a non-covalent direct thrombin inhibitor, compounds **2**–**4** [14,15,16] are covalent serine-trapping thrombin inhibitors. Potential inhibitors **24e**, **25**, **34**, and **10** developed in this work.

Hemostasis is an important physiological process necessary to prevent excessive blood loss in the event of a blood vessel injury. It is a tightly regulated process consisting of primary hemostasis, characterized by a weak platelet plug formation, and secondary hemostasis, associated with a cascade of biochemical reactions resulting in fibrin deposition. Dysregulation of hemostasis leads to thrombosis, which is a primary cause of mortality and morbidity worldwide. Thrombosis is a direct cause of myocardial infarction, ischemic stroke, and pulmonary embolism, which are life-threatening pathologies dramatically contributing to human mortality [1,2,3]. Novel antithrombotic drugs are required to prevent pathological thrombosis without affecting physiological hemostasis. However, this is not a trivial task as all clinically relevant anticoagulants, e.g., dabigatran (**1**, Figure 1) and rivaroxaban affect the enzymes of the blood coagulation cascade (thrombin and FXa) involved in both thrombosis and hemostasis [4,5]. Available anticoagulants therefore cause an unavoidable and potentially dangerous side effect of internal bleeding [6,7]. Hence, it is of high interest to develop new anticoagulants either affecting alternative targets of the blood coagulation cascade (e.g., FXIIa and FXIa [8,9,10,11]) or modulating known targets via alternative mechanisms.

Thrombin (FIIa) is a serine protease and a central enzyme of the blood coagulation cascade that converts soluble fibrinogen into insoluble fibrin that eventually seals the platelet plug forming a blood clot. It is additionally involved in a number of positive feedback loops of the cascade and in platelet activation further amplifying blood coagulation [12,13]. Thrombin, therefore, represents an important and well-established drug target in antithrombotic therapy.

Apart from well-known classical non-covalent thrombin inhibitors such as dabigatran (**1**), several developmental covalent thrombin inhibitors **2**–**4** have been reported in recent years (Figure 1) [14,15,16,17]. Unlike dabigatran (**1**) whose inhibitory properties rely solely on the non-covalent interactions with the residues in the thrombin’s active site, compounds **2**–**4** exhibit an electrophilic center (a carbonyl carbon atom) allowing for the covalent interaction with the catalytic Ser195 of thrombin. It has been experimentally proved that a nucleophilic attack of thrombin’s Ser195 on the electrophilic centers of compounds **2**–**4** results in the acylation of Ser195. Thereby, compounds **2**–**4** exhibit a so-called serine-trapping mechanism of action. Being acylated (trapped) Ser195 and, consequently, thrombin loses its catalytic and procoagulant properties. This type of covalent inhibition was shown to be transient (reversible) as formed acyl-enzyme complexes slowly degrade via hydrolysis [14,15,16]. The reversibility of thrombin inhibition with these type of covalent inhibitors was suggested as a beneficial property as an irreversibly trapped (*m*,odified) proteins tend to elicit undesired immunological response [18]. Moreover, it has been suggested that due to the distinctively different mechanism of inhibition, these covalent thrombin modifiers might be a safer alternative to classical covalent thrombin inhibitors [19]. To this end, recently reported acylated 1*H*-pyrazol-5-amine **3** and its patented analogs represent an interesting and yet underexplored class of potential antithrombotics with covalent (serine-trapping) mechanism of action [16,20,21]. Thus, for instance, clinical candidate VE-1902, structurally related to compound **3** (presumably an aminoazole bearing a pivaloyl moiety), was shown to prevent thrombosis in animal models showing only little to no influence on bleeding time [19]. Therefore, the development of new antithrombotic drugs based on the aminopyrazole scaffold is promising and of (pre)clinical interest.

We herein report the design, synthesis, and biological activity of a series of 1*H*-pyrazol-5-amine-based thrombin inhibitors with a serine-trapping mechanism of action. Particularly, the synthesis of acylated aminopyrazoles **24**, **25**, **34**, and their cyclized derivatives pyrazolo[5,1-*b*]quinazolin-9(4*H*)-ones **10a**–**i** is reported (Figure 1). Thrombin inhibitory properties of the synthesized compounds and their selectivity profile allowed to define structural features required for the successful covalent inhibition of thrombin.

## 2. Results and Discussion

### 2.1. Synthesis of Cyclic Aminoazoles

#### 2.1.1. Synthesis of pyrazolo[5,1-*b*]quinazolin-9(4H)-ones **10a**–**i**

**Figure 2:**
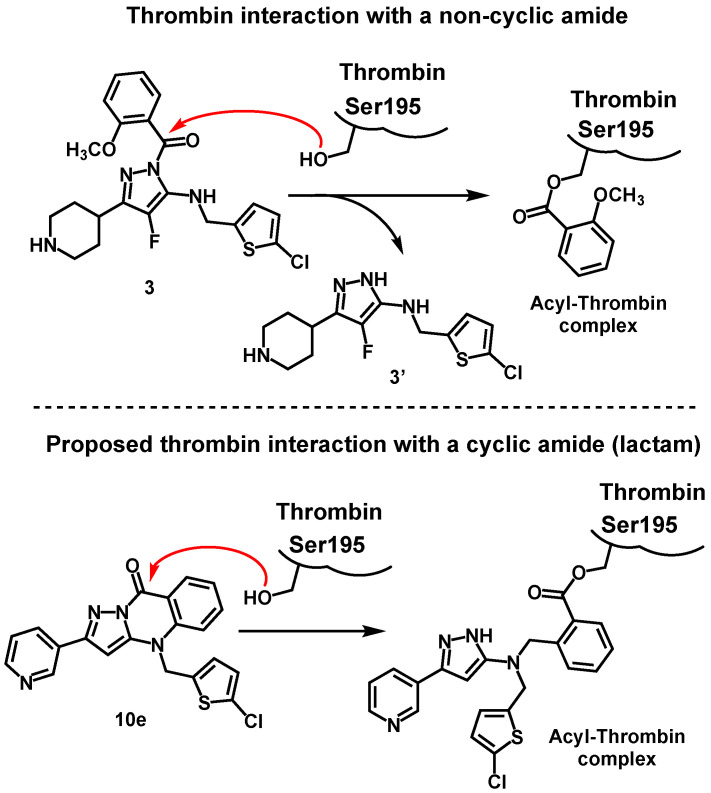
Schematic representation of thrombin’s Ser195 interaction with acylated 1*H*-pyrazol-5-amine **3** (**top**) vs. proposed interaction of thrombin with cyclic amides **10e** (**bottom**).

As shown in Figure 2, acylated 1*H*-pyrazol-5-amines possessing a non-cyclic amide moiety (e.g., compound **3**) upon interaction with the catalytic Ser195 of thrombin transfer only the acyl fragment to Ser195, whereas the cleaved off pyrazole residue (**3′**) leaves the active site [16]. This results in a transient inhibition of thrombin’s catalytic activity, as the acyl moiety bound to Ser195 forms only limited interactions with the active site. In contrast, the nucleophilic attack of Ser195 on the carbonyl C-atom of a lactam (e.g., **10e**, Figure 2) might result in an acyl-enzyme complex comprising the whole molecule of the inhibitor. This might allow a number of interactions between the active site of the enzyme and the covalent complex, which in turn should lead to an increase in the residence time of the inhibitor in the active site and a longer inhibition of the enzyme.

**Scheme 1:**
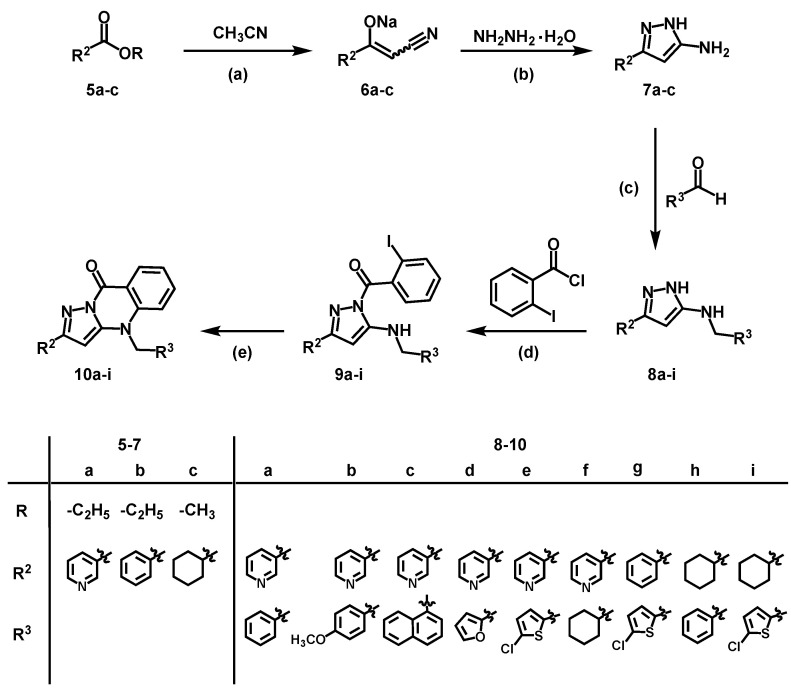
Synthesis of acylated pyrazoles **9** and pyrazolo[5,1-*b*]quinazolin-9(4*H*)-ones **10**. (**a**) NaH, THF, reflux under N_2_, 16 h, **6a** 88%, **6b** 75%, **6c** 72%; (**b**) 1. HCl (1M), 5 min; 2. NH_2_NH_2_·H_2_O, EtOH, reflux, 16 h, **7a** 75%, **7b** 94%, **7c** 76%; (**c**) 1. AcOH, molecular sieves (3Å), EtOH, r.t., 24 h; 2. NaBH_4_, EtOH, 0 °C to r.t., 16–24 h, **8a** 88%, **8b** 70%, **8c** 68%, **8d** 76%, **8e** 78%, **8f** 73%, **8g** 79%, **8h** 38%, **8i** 43%; (**d**) pyridine/THF, 0 °C to r.t., 2–3 h, **9a** 64%, **9b** 53%, **9c** 40%, **9d** 81%, **9e** 57%, **9f** 37%, **9g** 42%, **9h** 54%, **9i** 41%; (**e**) CuI, Cs_2_CO_3_, 1,10-phenanthroline, DMF, 80 °C, 0.5–3 h, **10a** 82%, **10b** 35%, **10c** 55%, **10d** 43%, **10e** 90%, **10f** 28%, **10g** 84%, **10h** 72%, **10i** 58%.

Therefore, to access 1*H*-pyrazol-5-amines exhibiting a carbonyl C-atom incorporated into the lactam ring, we developed a synthetic procedure towards pyrazolo[5,1-*b*]quinazolin-9(4*H*)-ones **10a**–**i** (Scheme 1). For this purpose, at first, β-ketonitriles **6a**–**c** were prepared from the commercially available esters **5a**–**c** by treating them with the deprotonated acetonitrile. The β-ketonitriles were isolated as stable sodium enolates [22]. Then, after the acidic treatment, keto-form of **6a**–**c** readily reacted with hydrazine monohydrate to form compounds **7a**–**c** exhibiting a 1*H*-pyrazol-5-amine-scaffold bearing heteroaromatic (**a**), aromatic (**b**), and cycloaliphatic (**c**) substituent in the 3-position (R^2^ substituent) (Scheme 1). Further structural diversity of compounds was achieved varying the substituent’s structure at the exocyclic primary amino group (R^3^). For this, a series of reductive amination reactions were performed accessing 1*H*-pyrazol-5-amines **8a**–**i** exhibiting an alkylated primary amino group. Among other moieties, 5-chlorothiophenyl group was introduced as it is present in previously reported covalent thrombin inhibitor **3** [16] (Figure 1). To facilitate subsequent cyclization reaction (a lactam ring formation), the annular nitrogen atom (N^1^) of the aminopyrazole scaffold of **8a**–**i** was acylated with 2-iodobenzoyl chloride. During the acylation process of **8a**–**i**, two main products were obtained, the desired N^1^-acylated compounds **9a**–**i** as well as the side product bearing the acyl moiety at the exocyclic amino group (in the ratio of approximately 3:1), which were separated by flash column chromatography. Acylated aminopyrazoles **9a**–**i** apart from being useful intermediates, are also potential inhibitors of thrombin and were screened in the enzyme inhibition tests. The final intramolecular cyclization step allowing us to access pyrazolo[5,1-*b*]quinazolin-9(4*H*)-ones **10** has not been reported in the literature and, therefore, requires development.

Considering that desired pyrazolo[5,1-*b*]quinazolin-9(4*H*)-ones **10** can be obtained in an Ullmann-type intramolecular cyclization reaction [23] from aryl iodides **9**, we screened different reaction conditions for a model reaction, in which **9e** was cyclized into desired product **10e** (Table 1).

Our first attempts to cyclize **9e** at room temperature using CuI as a catalyst and thiophene-2-carboxylic acid (entry 1, Table 1) or 1,10-phenanthroline (entry 2) as a ligand in the presence of Cs_2_CO_3_ produced **10e** in moderate yields of 40–44%. The reaction, however, required 72–96 h. Subsequent optimizations were performed using 1,10-phenanthroline as a ligand and DMF as a solvent. The reaction yield was significantly improved upon temperature elevation to 60 °C furnishing cyclized product **10e** in 4 h and 63% yield (entry 3). Further reaction temperature elevation to 80 °C allowed **10e** isolation with a high yield of 90% after 3 h (entry 4). During this reaction run, the TLC analysis showed practically full consumption of the starting material already after 30 min (entry 4). However, further attempts to change the solvent to DMSO and perform the reaction over 30 min resulted in a decreased yield (74%, entry 5). The replacement of Cs_2_CO_3_ with another base like K_3_PO_4_ proved to be fully compatible with the reaction allowing us to isolate **10e** with 90% yield (entry 6). In contrast, the use of K_2_CO_3_, triethylamine, or no base resulted in lower to no yields (entries 7–9). Similarly, the removal of 1,10-phenanthroline or its replacement with the other ligands like thiophene-2-carboxylic acid or L-proline resulted in the reaction yield reduction (entries 10–12). Additionally, our attempts to increase the reaction temperature to 100 °C, while reducing the reaction time to 15 min resulted in lower yields of product **10e** (entries 13–14) compared to reactions performed at 80 °C (entries 4 and 6). This may be due to the decomposition of the starting material or the product rather than insufficient reaction time since complete consumption of the starting material was observed after 15 min at 100 °C. Using the optimized reaction conditions (entry 3, Table 1, with a variable reaction time), a series of desired pyrazolo[5,1-*b*]quinazolin-9(4*H*)-ones **10a**–**i** were successfully synthesized (Scheme 1) to be further tested for their ability to inhibit thrombin.

**Figure 3:**
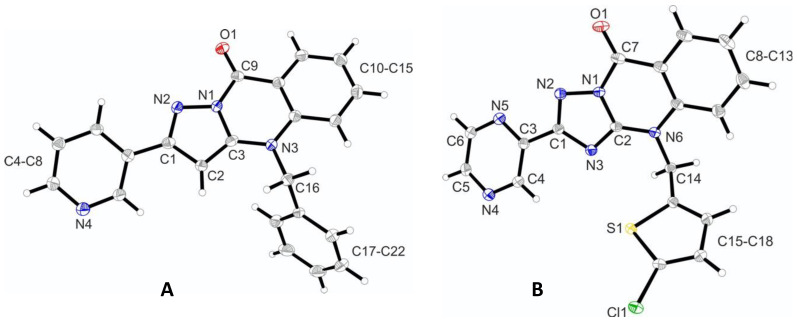
X-ray crystal structure of **10a** displaying the thermal ellipsoids at the 50% probability level (**A**); X-ray crystal structure of **19** displaying the thermal ellipsoids at the 50% probability level (**B**).

It is reported that 1*H*-pyrazol-5-amines exhibit annular tautomerism [24,25]. Therefore, upon their acylation, three different N-atoms could be theoretically acylated (two annular N-atoms and the exocyclic one). This, for example, is well-described for analogous 1,2,4-triazol-5-amines [26]. Moreover, according to the literature data and from our own experience, aminoazoles acylated at annular N^1^-atom undergo a so-called thermal acyl moiety migration [27,28] giving products bearing an acyl moiety at the exocyclic amino group. These peculiar properties of aminoazoles may lead to the mixture of reaction products and their misidentification. Therefore, to unambiguously prove the structure of cyclized products **10a**–**i**, we grown a crystal of compound **10a** and subjected it to X-ray crystallographic analysis (Figure 3A). The crystal structure revealed that the acylation indeed took place at the annular N^1^-atom followed by the successful ring closure without the acyl moiety migration. Moreover, X-ray crystal structure showed additional structural features of the synthesized pyrazolo[5,1-*b*]quinazolin-9(4*H*)-ones. Thus, the pyridyl moiety of **10a** appeared to be not coplanar to the pyrazolo[5,1-*b*]quinazolin tricyclic system exhibiting an offset of about 24 degrees. Additionally, the introduced flexible methylene bridge (C^16^-atom, Figure 3A) positions the N-benzyl moiety completely out of the plane of the fused tricyclic system further shaping the structure of the molecule and adding certain three-dimensional properties.

#### 2.1.2. Synthesis of Other Aminopyrazol- and Aminotriazole-Based Cyclic Derivatives

**Scheme 2:**
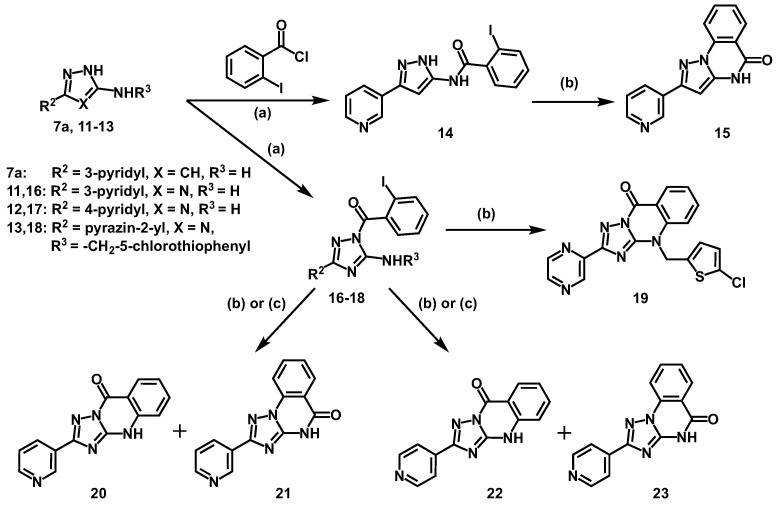
Synthesis of aminopyrazol- and aminotriazole-based cyclic derivatives **15**, and **19**–**23**. (**a**) pyridine/THF, 0 °C to r.t., 2–4 h, **14** 63%, **16**, 78% **17**, 74% **18** 59%; (**b**) CuI, Cs_2_CO_3_, 1,10-phenanthroline, DMF, 80 °C, 0.5–1 h, **15** 21%, **19** 80%, **20** + **21** (1:1) 79%, **22** + **23** (3:7) 68%; (**c**) CuI, Cs_2_CO_3_, 1,10-phenanthroline, DMF, microwave irrad. at 150 °C, 1 h, **20** + **21** (7:3) 92%, **22** + **23** (3:7) 95%.

To further explore the SAR and probe the scope of the developed Ullmann-type synthetic protocol, in addition to cyclized compounds **10a**–**i**, the synthesis of aminopyrazol- and aminotriazole-based cyclic derivatives **15** and **19**–**23** was performed (Scheme 2). For this, at first, aminopyrazole **14** (produced via the acylation of **7a** with 2-iodobenzoyl chloride) was successfully cyclized into lactam **15** following the developed Ullmann-type synthetic procedure. This showed that the developed protocol can also be used to access pyrazolo[1,5-*a*]quinazolin-5(4*H*)-ones, which are regioisomeric to compounds **10**, although, lacking the substituents at the amide N-atom.

Then, to better understand the influence of an additional N-atom at the pyrazole core on the biological activity, we prepared three 1,2,4-triazol-5-amines **16**–**18** bearing 2-iodobenzoyl moiety at their annular N^1^-atom (Scheme 2) and subjected them to the intramolecular cyclization reaction. Similarly to 1*H*-pyrazol-5-amines, their aminotriazole-based analog **18**, exhibiting an alkylated exocyclic amino group, smoothly produced lactam **19**. The structure of **19** was unambiguously confirmed by X-ray crystallography (Figure 3B). This further extends the scope of the developed synthetic protocol showing its utility for the synthesis of [1,2,4]triazolo[5,1-*b*]quinazolin-9(4*H*)-ones. Finally, pyridyl-derived aminotriazoles **16** and **17**, exhibiting the unsubstituted exocyclic amino group, were cyclized, yielding, however, two products in each case. In addition to the expected cyclization products **20** and **22**, their regioisomers **21** and **23** were isolated (Scheme 2).

**Scheme 3:**
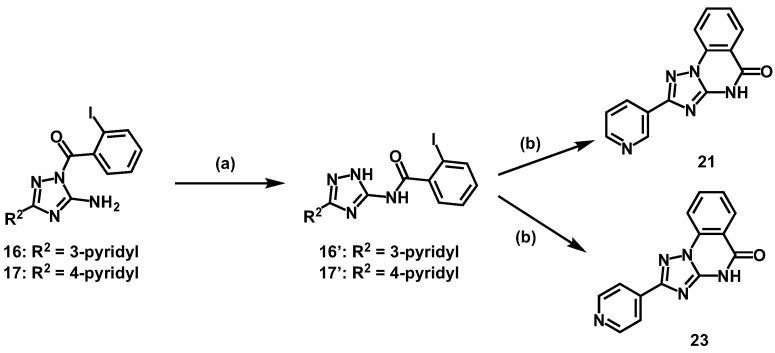
Synthesis of [1,2,4]triazolo[1,5-*a*]quinazolin-5(4*H*)-ones **21** and **23**. (**a**) 260 °C, 15 min, neat **16′** 80%, **17′** 76%; (**b**) CuI, Cs_2_CO_3_, 1,10-phenanthroline, DMF, 80 °C, 1 h, **21** 67%, **23** 42%.

Compounds **21** and **23** might be formed because of the thermal acyl moiety migration from the annular N^1^-atom to the exocyclic amino group, followed by the ring closure in the Ullmann-type reaction. This is probable as acyl migration for similar compounds is well documented in the literature [27,28]. Nevertheless, our attempts to improve the reaction regioselectivity by reducing the reaction temperature resulted in no significant improvement, once again yielding two cyclization products simultaneously. Additionally, the reaction performed at a higher temperature (150 °C) under the microwave irradiation (conditions (c), Scheme 2) yielded the mixture of regioisomers, although they were obtained with higher yields. We separated both regioisomers chromatographically to subsequently study their biological activity. Nevertheless, to unambiguously assign the structures of **20** and **21** as well as **22** and **23**, we performed the additional syntheses, which exclusively produced compounds **21** and **23** (Scheme 3). For this purpose, acylated aminotriazoles **16** and **17** were first subjected to the high temperature-promoted (260 °C) acyl moiety migration to afford intermediates **16′** and **17′**, which were then cyclized into [1,2,4]triazolo[1,5-*a*]quinazolin-5(4*H*)-ones **21** and **23** exclusively (Scheme 3).

### 2.2. Serine Protease (FIIa and FXIIa) Inhibition by Compounds **9a**–**i**, **10a**–**i**, **14**, **15**, and **19**–**23**

A series of synthesized cyclic compounds **10a**–**i**, **15**, and **19**–**23** were studied for their ability to inhibit the proteolytic activity of thrombin as well as of blood coagulation factor XIIa (FXIIa). FXIIa was additionally selected for the screening because acylated azoles of similar structure are reported to potently inhibit FXIIa [8,9]. We also tested the inhibitory properties of a series of noncyclic acylated aminopyrazoles **9a**-**i** and compound **14** (Table 2) as they share structural similarity with the lead compound **3**.

Among 1*H*-pyrazol-5-amines **9a**–**i** exhibiting 2-iodobenzoyl moiety, only two compounds, namely **9e** and **9g,** were able to inhibit thrombin with the IC_50_ values of 165 nM and 1.3 μM, respectively (Table 2). At that, both compounds **9e** and **9g** comprise the 5-chlorothiophene moiety linked to the exocyclic amino group via a methylene bridge. Interestingly, compounds bearing other cycloaliphatic (**9f**) and (hetero)aromatic (**9a**–**d**,**f**) substituents at the exocyclic amino group showed no thrombin inhibition (IC_50_ > 5 μM). The substituent’s structure in the 3-position of the pyrazole core also significantly influenced the ability of compounds to inhibit thrombin. Thus, compound **9e** exhibiting the 3-pyridyl moiety was about 8-fold more potent thrombin inhibitor than its 3-phenyl-substituted counterpart **9g** (IC_50_ 165 nM vs. 1.3 μM). Compound **9g**, in turn, outperformed the 3-cyclohexyl-substituted analog **9i** that showed no activity. None of the compounds inhibited FXIIa (IC_50_ > 5 μM).

Subsequently performed screening revealed that irrespective to the structure, none of the synthesized aminopyrazole- and aminotriazole-based cyclized derivatives **10a**–**i**, **15**, and **19**–**23** showed thrombin or FXIIa inhibitory properties (Table S1 in Supporting Information). Considering that the noncyclic precursors **9e** and **9g** were active inhibitors of thrombin, it was concluded that the activity drop is directly associated with the performed ring closure. It might be because of several reasons. Thus, compared to the non-cyclic derivatives, the carbonyl C-atom of the formed quinazolinone ring of the cyclized compounds is significantly less reactive in nucleophilic addition-elimination reactions. This might considerably reduce its ability to form covalent interactions with the catalytic Ser195 of thrombin. Additionally, cyclized compounds, e.g., **10a**–**i** are significantly more rigid compared to their noncyclic analogs **9a**–**i**. This rigidity might prohibit them from adopting an active conformation within the active site of the enzyme, e.g., the facile access to the cyclic compounds’ carbonyl C-atom might be prohibited. Therefore, further development of active covalent thrombin inhibitors with serine-trapping mechanism of action should be continued with more flexible derivatives.

### 2.3. Synthesis of Flexible Aminopyrazol-Based Thrombin Inhibitors

**Scheme 4:**
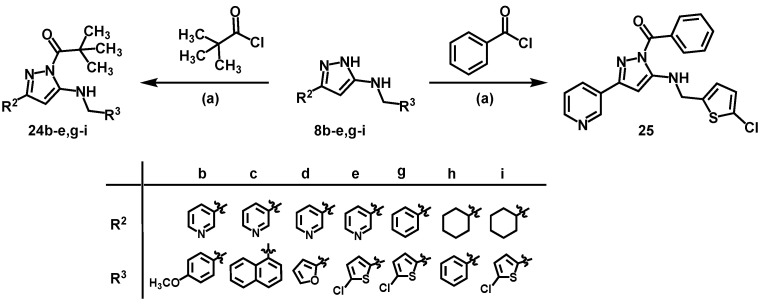
Synthesis of acylated 1*H*-pyrazol-5-amines **24b**–**e**,**g**–**i** and **25**. (**a**) pyridine/THF, 0 °C to r.t., 2–4 h, **24b** 44%, **24c** 40%, **24d** 56%, **24e** 52%, **24g** 44%, **24h** 53%, **24i** 30%, **25** 56%.

**Scheme 5:**
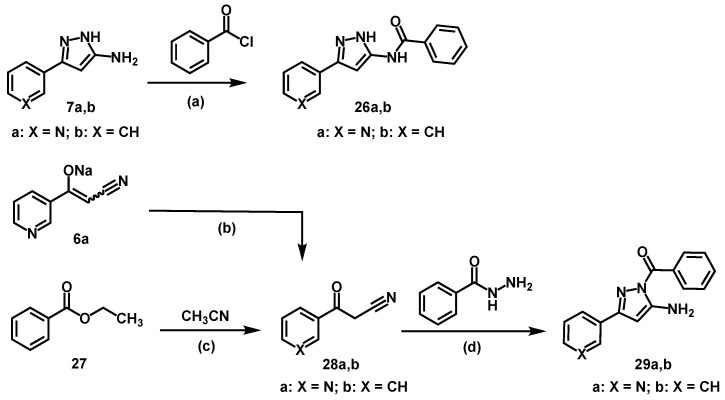
Synthesis of acylated 1*H*-pyrazol-5-amines **26a,b** and **29a,b**. (**a**) pyridine/THF, 0 °C to r.t., 2–4 h, **26a** 68%, **26b** 77%; (**b**) HCl (1M), 5 min; (**c**) N_2_, *n*-BuLi, ™78 °C to r.t., THF, 16 h, **28b** 59%; (**d**) MsOH, EtOH, reflux, 45 min, **29a** 56%, **29b** 97%.

**Scheme 6:**
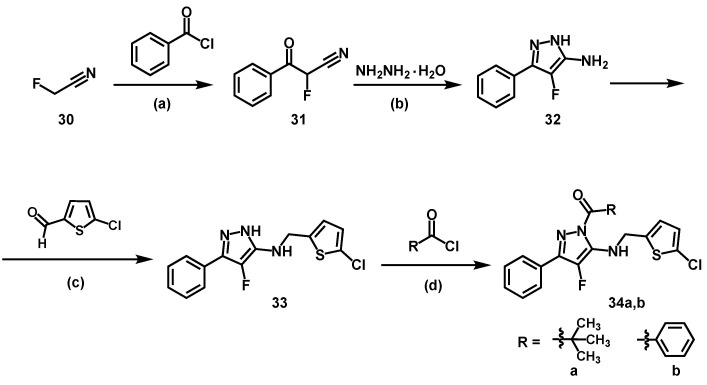
Synthesis of acylated 4-fluoro-3-phenyl-1*H*-pyrazol-5-amines **34a**,**b**. (**a**) N_2_, Ph_2_P(O)Cl, LiHMDS, ™78 °C to r.t., THF, 75 min, **31** 17%; (**b**) *i*-PrOH, reflux, 3 h, **32** 55%; (**c**) 1. AcOH, molecular sieves (3Å), EtOH, 0 °C to r.t., 24 h; 2. NaBH_4_, EtOH, 0 °C to r.t., 24 h, **33** 77%; (**d**) pyridine/THF, 0 °C to r.t., 24 h, **34a** 21%, **34b** 34%.

As cyclized aminopyrazole- and aminotriazole-based rigidified analogs of **3** appeared to be inactive inhibitors of thrombin, we decided to make one step back and synthesize a focused library of more flexible acylated 1*H*-pyrazol-5-amines **24b**–**e**,**g**–**i**, **25**, **29a**,**b**, and **34a**,**b** (Scheme 4, Scheme 5 and Scheme 6).

At first, acylated 1*H*-pyrazol-5-amines **24b**–**e**,**g**–**i** possessing the pivaloyl moiety in their N^1^-position were synthesized via the acylation of aminopyrazoles **8b**–**e**,**g**–**i** (Scheme 4). Pivaloyl moiety was selected intentionally as it is known to be selective towards thrombin [19,21]. In addition, benzoylated aminopyrazole **25** was prepared (Scheme 4) as benzoyl moiety is also known to be tolerated by thrombin [17], and it was interesting to compare the activity of compound **25** with its pivaloylated analog **24e**.

Then, to justify the necessity of a substituent at the exocyclic amino group, we attempted to prepare two benzoylated 1*H*-pyrazol-5-amines **29a**,**b** exhibiting the unsubstituted 5-amino group on their pyrazole scaffold (Scheme 5). However, our first attempt to directly acylate aminopyrazoles **7a**,**b** failed to deliver desired products **29a**,**b** bearing the acyl moiety in the N^1^-position. Instead, in the absence of a steric hindrance (unsubstituted primary amino group), regioisomeric compounds **26a**,**b** acylated in the undesired position were formed (Scheme 5). Therefore, an alternative approach towards **29a**,**b** was utilized. For this, the benzoyl moiety was introduced indirectly via the cyclocondensation reaction between β-ketonitriles **28a**,**b** and benzhydrazide already exhibiting the benzoyl fragment (Scheme 5).

Finally, to find out whether the fluorine atom present in the 4-position of the 1*H*-pyrazol-5-amine core of, e.g., inhibitor **3** is required for the successful inhibition of thrombin, we synthesized two fluorinated aminopyrazoles **34a**,**b** (Scheme 6). For this purpose, fluoroacetonitrile (**30**) was deprotonated and reacted with benzoyl chloride to furnish β-ketonitrile **31**, which was subsequently cyclized into the fluorinated 1*H*-pyrazol-5-amine **32** using hydrazine monohydrate [29]. Subsequently performed reductive amination reaction allowed to access *N*-alkylated product **33**, which was then N^1^-acylated either with pivaloyl chloride or benzoyl chloride to afford potential thrombin inhibitors **34a**,**b** bearing the fluorine atom in the 4-position.

### 2.4. Serine Protease (FIIa and FXIIa) Inhibition by Compounds **24b**–**e**,**g**–**i**, **25**, **29a**,**b**, and **34a**,**b**

A series of synthesized flexible acylated 1*H*-pyrazol-5-amines **24b**–**e**,**g**–**i**, **25**, **29a**,**b**, and **34a**,**b** were screened for their ability to inhibit the proteolytic activity of thrombin and FXIIa (Table 3). In general, seven compounds exhibited thrombin inhibitory properties, of which four appeared to be potent thrombin inhibitors.

Several structural elements influenced the compounds’ inhibitory properties towards thrombin. First, the structure of a substituent at the exocyclic amino group affected the compounds’ activity. Aminopyrazoles **29a** and **29b** exhibiting the unsubstituted primary amino moiety showed no inhibitory activity towards thrombin (IC_50_ > 5 μM). The introduction of bulky naphthyl- (compound **24c**) and 4-methoxyphenyl- (**24b**) residues or a smaller furyl (**24d**) moiety linked via the methylene bridge to the primary amino group either had no effect or resulted in thrombin inhibition in the micromolar range (IC_50_ ~ 1.3 μM, Table 3). In contrast, the introduction of the 5-chlorothiophen-2-yl moiety in this position shifted compounds’ inhibitory activity against thrombin in the nanomolar range. E.g., acylated 1*H*-pyrazol-5-amines **24e**, **24g**, and **25** bearing the mentioned moiety inhibited thrombin with low IC_50_ values of 16 nM, 419 nM, and 18 nM, respectively.

The structure of the substituent in the 3-position of the 1*H*-pyrazol-5-amine core was another important structural element influencing compounds’ inhibitory properties. Thus, in the line of otherwise identically substituted compounds, 3-pyridyl-substituted derivative **24e** was 26-fold more potent thrombin inhibitor (IC_50_ = 16 nM) than its 3-phenyl-substituted analog **24g** (IC_50_ = 419), which in turn, was more potent than 3-cyclohexyl-substituted compound **24i** showing no activity (IC_50_ > 5 μM, Table 3). Additionally, another 3-cyclohexyl-substituted compound **24h** appeared to be inactive in the performed assays.

The variety of acyl moieties was represented by only two residues namely pivaloyl and benzoyl. It has been found that both moieties are well tolerated by thrombin, and compounds bearing corresponding acyl fragments demonstrated similar thrombin inhibitory ability (e.g., pairs of compounds **24e** vs. **25** and **34a** vs. **34b**, Table 3).

Finally, the presence/absence of the fluorine atom in the 4-position of the pyrazole core was found to be important for the inhibitory properties. Thus, the fluorine atom introduction was able to partially compensate for the absence of a heteroatom in the aromatic substituent in the 3-position. To this end, fluorinated compounds **34a** and **34b** were more potent inhibitors of thrombin (IC_50_ = 80 nM and 71 nM, respectively) than their nonfluorinated analog **24g** (IC_50_ = 419 nM, Table 3).

### 2.5. In-Depth Biological Activity Evaluation of Thrombin Inhibitors **24e**, **25**, and **34a**,**b**

#### 2.5.1. Serine Protease Inhibitory Profile of **24e**, **25**, and **34a**,**b**

The four most potent thrombin inhibitors were selected for in-depth study of their biological activity. Particularly, compounds **24e**, **25**, **34a**, and **34b** were assayed for their selectivity against the panel of physiologically relevant serine proteases (eight enzymes), which similarly to thrombin exhibit catalytic Ser residue (Table 4). In general, apart from the benzoylated aminopyrazole **25** exhibiting pyridyl moiety in the 3-position, which to some extend off-targeted chymotrypsin (IC_50_ = 242 nM), plasma kallikrein (IC_50_ = 639 nM), and FXIa (1 μM), other three compounds were highly selective towards thrombin. For instance, fluorinated compound **34a**, an 80 nM thrombin inhibitor, showed no inhibition of other tested serine proteases being screened at 5 μM (Table 4). Additionally, being 16 nM thrombin inhibitor, compound **24e**, showed only a slight off-targeting effect against chymotrypsin, which required a 55-fold higher dose of **24e** to be inhibited by 50% than that required for thrombin (Table 4). Interestingly, the pivaloyl moiety seemed to be superior to the benzoyl moiety in terms of compounds’ selectivity towards thrombin.

#### 2.5.2. The Mechanism of Thrombin Inhibition

**Figure 4:**
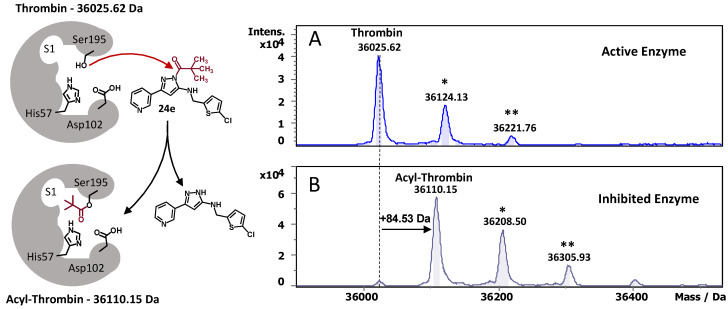
Deconvoluted ESI(+)-MS mass spectra of native thrombin (**A**) and acyl-thrombin complex (**B**) formed after the enzyme incubation with 79 μM thrombin inhibitor **24e**. The peaks of interest are labelled with the corresponding deconvoluted masses. A mass shift of 84.5 Da (**B**) was observed, which corresponds to the inhibitor’s acyl moiety adduct to thrombin. The schematic representation of thrombin and the covalent complexes of thrombin with the inhibitor are also shown. *—single and **—double sulfuric acid or phosphoric acid adduct [30].

To prove that synthesized acylated 1*H*-pyrazol-5-amines are covalent thrombin inhibitors with a serine-trapping mechanism of action, the most potent compound **24e** (Table 4) was studied in the mass-shift assay. For this purpose, the mass of native human thrombin was measured using a timsTOF instrument in ESI(+) mode (Figure 4A). Then, native thrombin was preincubated together with the excess of **24e** for 15 min, and the mass was measured again. It has appeared that the mass of the intact thrombin 36,025.62 Da was shifted to 36,110.15 Da upon the incubation with **24e** (Figure 4B). The observed mass-shift of Δm = 84.53 Da corresponds to the mass of the pivaloyl moiety of aminopyrazole **24e**. Thus, it proves that upon contact with thrombin, **24e** transfers its acyl moiety to the enzyme. As the mass of thrombin is increased by the mass of only one pivaloyl fragment, it implies that thrombin is acylated once in a specific position. This position is most probably located in the active site of the enzyme, where the most nucleophilic catalytic Ser195 resides. Being trapped (acylated) Ser195 loses its catalytic activity for a lifetime of the formed acyl-thrombin complex.

#### 2.5.3. The Influence of Compounds on Plasma Coagulation

**Figure 5:**
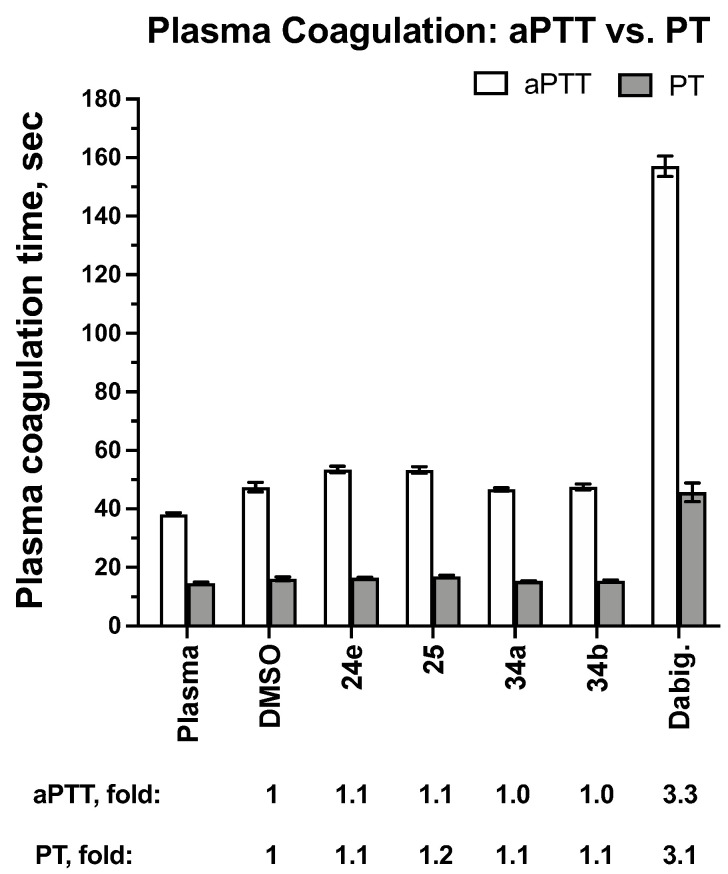
The influence of compounds on plasma coagulation (in vitro). Selected acylated 1*H*-pyrazol-5-amines were tested at 200 μM and dabigatran (**1**) was tested at 2 μM. The activated partial thromboplastin time (aPTT) and prothrombin time (PT) are shown in sec. The fold increase in aPTT and PT compared to the effect of DMSO is shown under the diagram. Tests were performed at least in triplicate, and the average with standard deviation (SD) is given.

It has been reported that clinical candidate VE-1902 (phase 1 clinical trials, trial ID ACTRN12618001509257) structurally related to compound **3** (Figure 1) exhibits covalent reversible mechanism of thrombin inhibition but practically has no influence on blood coagulation time in two standard tests namely activated partial thrombin time (aPTT) and prothrombin time (PT) [16,19,31]. These two tests determine the extent to which the test compound prolongs the clotting time, and also help determine which clotting pathway is affected—intrinsic or extrinsic. Thus, it has been shown that VE-1902 was not able to double the clotting time in aPTT and PT tests being tested at a relatively high dose of 100 μM (see the Supporting Information from the reference [19]). Despite this, VE-1902 was effective in a preclinical model of thrombosis, and with no direct effect on clotting time, it caused virtually no bleeding complications in mice [19]. So, it has been considered beneficial that VE-1902 has practically no influence on aPTT and PT. Keeping this in mind, we decided to test whether synthesized compounds **24e**, **25**, **34a**, and **34b** have a similar influence on the aPTT and PT. For this purpose, synthesized thrombin inhibitors were assayed in these two blood coagulation tests (Figure 5).

Interestingly, despite their pronounced thrombin inhibitory properties (Table 4), acylated 1*H*-pyrazol-5-amines **24e**, **25**, **34a**, and **34b** showed very little influence on blood coagulation time in aPTT and PT tests (Figure 5), which is, however, in agreement with previously published results [16,19]. Thus, at a high dose of 200 μM, tested compounds extended aPTT by maximum of 1.1-fold (**24e** and **25**), whereas PT was prolonged by 1.2-fold maximum (compound **25**). In this respect, dabigatran (**1**) being tested at a significantly lower dose of 2 μM, extended aPTT and PT by 3.3-fold and 3.1-fold, respectively.

From the mechanistic point of view, the compounds’ low influence on the plasma coagulation time might be related with their covalent mechanism of action. The catalytic Ser trapping is a relatively slow process [16] when compared with the non-covalent interactions taking place between the non-covalent inhibitor, e.g., dabigatran (**1**) and thrombin. The in vitro tests (aPTT and PT) utilize very high doses of activating agents that trigger either extrinsic or the intrinsic plasma coagulation. Thus, in this particular case, the aPTT and PT tests may not be entirely appropriate for characterizing the antithrombotic potential of covalent thrombin inhibitors, since the observed modulation of thrombin by this class of compounds was sufficient to cause an antithrombotic effect in vivo, as shown in the case of VE-1902 [19].

### 2.6. Binding Mode Study by Molecular Modeling

**Figure 6:**
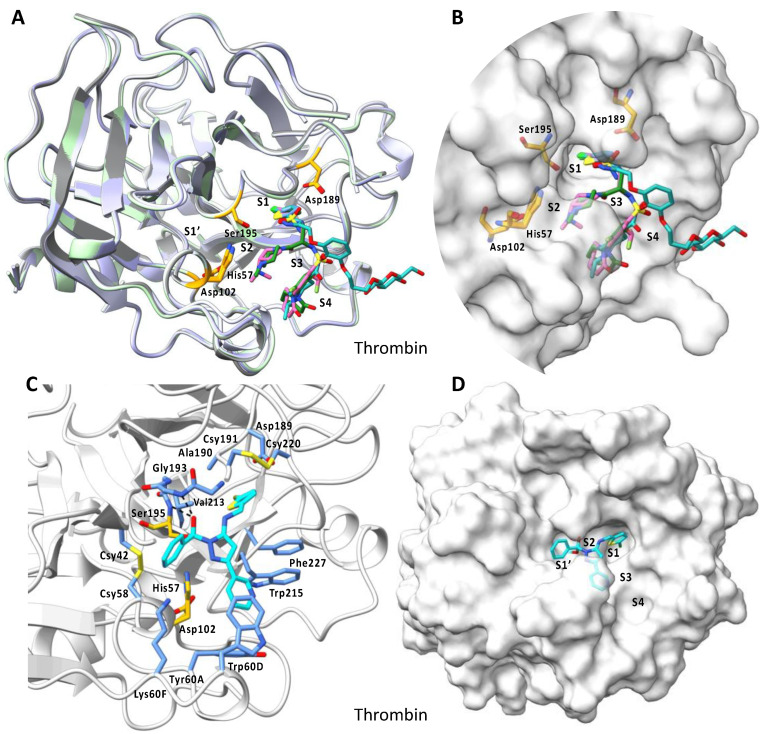
Four superimposed thrombin X-ray crystal structures (**A**—ribbons, **B**—molecular surface) with bound inhibitors exhibiting the 5-chlorothiophenyl moiety (PDB ID: 4LOY [32], 4LXB [32], 6EO8 [33], and 6YQV). In all superimposed structures, the inhibitors’ 5-chlorothiophenyl moiety resides in the S1 pocket. Calculated covalent binding conformation of inhibitor **25** (cyan stick model) in the active site of thrombin (**C**—close-up view and **D**—overall structure with molecular surface). Amino acid residues are depicted as orange or blue stick models and are numbered according to the amino acid sequence of chymotrypsinogen residue numbering. Oxygen, nitrogen, and sulfur atoms are colored in red, blue, and yellow, respectively. Substrate-binding sites are labeled (S1–S4 and S1′). Hydrogen bonds are black lines. PDB ID used for docking: 6CYM [16].

To rationalize the inhibitory activity of synthesized acylated 1*H*-pyrazol-5-amines and gain insight into their covalent binding conformation, inhibitor **25** exhibiting the 5-chlorothiophen-2-yl moiety (Table 4) was docked into the active site of thrombin (Figure 6). Before performing the actual docking, we searched through the Protein Data Bank (PDB) for thrombin inhibitors bearing the 5-chlorothiophen-2-yl moiety. We found four X-ray crystal structures satisfying this criterion (PDB ID: 4LOY [32], 4LXB [32], 6EO8 [33], and 6YQV). These four thrombin-inhibitor complexes were superimposed to reveal that irrespective to their structure, the inhibitors’ 5-chlorothiophenyl moiety resides in the S1 pocket adopting almost identical conformation (Figure 6A,B). Apart from thrombin, multiple inhibitors of blood coagulation factor Xa exhibit the 5-chlorothiophen-2-yl moiety that also binds to the S1 pocket of FXa [32,34,35]. Considering binding modes of different 5-chlorothiophen-2-yl-containing thrombin and FXa inhibitors as well as the fact that among synthesized aminopyrazoles only 5-chlorothiophen-2-yl-substituted derivatives were highly active, we assumed that the 5-chlorothiophen-2-yl moiety of the synthesized aminopyrazoles must bind to the S1 pocket of thrombin. Therefore, the constrained docking was performed using the 5-chlorothiophen-2-yl moiety as a pharmacophore feature residing in the S1 pocket (Figure 6C,D).

Performed docking revealed that exemplary compound **25** could covalently bind to the catalytic Ser195 in the active site of thrombin, forming thereby a tetrahedral intermediate (cyan stick model in Figure 6A). The tetrahedral intermediate is formed as a result of a nucleophilic attack of Ser195 on the carbonyl carbon of **25**. The resulting hydroxy group of **25** forms hydrogen bonds with the backbone amides of the “oxyanion hole” (Gly193 and Ser195), thereby mimicking the interactions between thrombin and a peptide substrate (e.g., fibrinogen). Apart from these interactions, the aromatic acyl fragment of **25** pointing towards the S1′ pocket forms lipophilic interactions with, e.g., Cys42, Cys58, His57, and Lys60F, whereas the pyridyl fragment resides in the narrow S2 pocket interacting with Trp60D and Tyr60A. The 5-chlorothiophen-2-yl moiety of **25** is also involved in several lipophilic interactions with the amino acid residues of the S1 pocket such as Val213, Ala190, Cys191, Cys220, Phe227 (backbone), and Trp215 (backbone) (Figure 6C,D). It is, however, reported that the inhibitory activity boost of the 5-chlorothiophen-2-yl-substituted compounds is explained not by these lipophilic interactions but mainly by their ability to displace a specific water molecule from the S1 pocket of trypsin-like serine proteases, e.g., thrombin and FXa. This single water molecule displacement is associated with an affinity boost [36,37,38].

## 3. Conclusions

In this study, we synthesized and evaluated the biological activity of a series of 1*H*-pyrazol-5-amine-based thrombin inhibitors with a serine-trapping mechanism of action. The initially synthesized series of acylated aminopyrazoles **9** and their cyclized derivatives pyrazolo[5,1-*b*]quinazolin-9(4*H*)-ones **10** (Scheme 1 and Scheme 2) appeared to be low active or inactive towards thrombin (Table 2 and Table S1). Similarly, their aminotriazole-based cyclized analogs showed no ability to inhibit thrombin (Table S1). In contrast, the synthesis of a focused library of flexible acylated 1*H*-pyrazol-5-amines **24**, **25**, **29**, and **34** (Scheme 4, Scheme 5 and Scheme 6) allowed to identify potent thrombin inhibitors (Table 3). Subsequently performed in-depth study of four most potent compounds revealed that acylated aminopyrazoles **24e**, **34a**, and **34b** are highly selective thrombin inhibitors showing low to no off-target inhibition of other tested physiologically relevant serine proteases (Table 4). To prove that synthesized compounds are covalent thrombin inhibitors, the most potent derivative **24e** (FIIa IC_50_ = 16 nM) was studied in the mass-shift assay (Figure 4). In this assay, it has been shown that **24e** transfers its acyl moiety (pivaloyl) to thrombin. Considering that **29e** acylated thrombin only once (according to the mass-shift), this process must be highly specific and, most probably, should take place at the catalytic Ser195 residing in the active site of thrombin. This is in agreement with two other facts such as the disturbance of thrombin catalytic activity in the presence of **24e** as well as the X-ray crystal structure of thrombin possessing the acyl moiety of compound **3** at its Ser195 [16]. Performed herein docking studies also confirmed that synthesized acylated aminopyrazoles may inactivate the catalytic activity of thrombin via a covalent interaction with its Ser195 and also forming other non-covalent interactions in the active site (Figure 6). Most active thrombin inhibitors **24e**, **25**, **34a**, and **34b** found during this study, were evaluated for their ability to influence plasma coagulation in aPTT and PT in vitro assays. None of the compounds showed significant influence on plasma coagulation, despite their high inhibitory potency toward thrombin. Nevertheless, this is in agreement with observations previously made by other authors for this class of covalent thrombin inhibitors [7,16]. It is expected that thrombin inhibitors of this kind should have little to no influence on bleeding time [19] and can be considered as a safer alternative to conventional non-covalent anticoagulants, which are often associated with potentially dangerous side effect of internal bleeding [6,7]. However, only in vivo tests could uncover the full antithrombotic potential of this series of covalent thrombin inhibitors like it has already been shown for the clinical candidate VE-1902 [19].

## 4. Materials and Methods

**Chemistry, General**. Unless otherwise mentioned, THF was dried with sodium/benzophenone and was freshly distilled before use. Thin layer chromatography (TLC): silica gel 60 F_254_ plates (Merck). Flash chromatography (FC): silica gel 60, 40–63 µm (Macherey-Nagel). Reversed phase thin layer chromatography (RP-TLC): silica gel 60 RP-18 F_254_S plates (Merck). Automatic flash column chromatography: Isolera One (Biotage); brackets include eluent, cartridge-type. Melting point (*m*,.p.): melting point apparatus SMP 3 (Stuart Scientific), uncorrected. ^1^H NMR (400 MHz), ^1^H NMR (600 MHz), and ^13^C NMR (151 MHz): Agilent DD2 400 and 600 MHz spectrometers; chemical shifts (δ) are reported in ppm against the reference substance tetramethylsilane and calculated using the solvent residual peak of the undeuterated solvent. IR: IR Prestige-21 (Shimadzu). HRMS: MicrOTOF-QII (Bruker). HPLC method to determine the purity of compounds: equipment 1: pump: L-7100, degasser: L-7614, autosampler: L-7200, UV detector: L-7400, interface: D-7000, data transfer: D-line, data acquisition: HSMS software (all from LaChrom, Merck Hitachi); equipment 2: pump: LPG-3400SD, degasser: DG-1210, autosampler: ACC-3000T, UV detector: VWD-3400RS, interface: Dionex UltiMate 3000, data acquisition: Chromeleon 7 (Thermo Fisher Scientific); column: LiChrospher 60 RP-select B (5 μm), LiChroCART 250–4 mm cartridge; flow rate: 1.0 mL/min; injection volume: 5.0 μL; detection at λ = 210 nm; solvents: A: demineralized water with 0.05% (*v*/*v*) trifluoroacetic acid, B: acetonitrile with 0.05% (*v*/*v*) trifluoroacetic acid; gradient elution (% A): 0–4 min: 90%; 4–29 min: gradient from 90 to 0%; 29–31 min: 0%; 31–31.5 min: gradient from 0 to 90%; 31.5–40 min: 90% [14,17].

**General procedure A.** Synthesis of β-ketonitriles. If not explicitly described otherwise, under N_2_, NaH (0.98 eq, 60% mineral oil dispersion) was added to a stirred solution of the respective ester (1.00 eq.) and dry CH_3_CN (0.98 eq.) in dry THF at rt. The resulting suspension was refluxed for 16 h, allowed to cool to rt, filtrated and washed with Et_2_O (3×). The product was dried in vacuo to yield the sodium enolate of β-ketonitrile, which was used without further purification in the next step.

**General procedure B.** Synthesis pyrazol-5-amines. If not explicitly described otherwise, the sodium enolate of the β-ketonitrile (1.00 eq.) was dissolved in aqueous HCl (1M) and stirred at rt for 5 min. The pH was adjusted to 4–5 with aqueous NaOH (2M), and the aqueous phase was extracted with EtOAc (6×). The combined organic layers were dried (Na_2_SO_4_), filtered and quickly concentrated in vacuo as decomposition of the β-ketonitrile was observed. To the orange oily residue (if the keto-form of β-ketonitrile was used, 1.00 eq of the keto-form was taken from this step on) was added EtOH and NH_2_NH_2_·H_2_O (2.00–3.00 eq.) and the solution was refluxed for 16 h. After cooling down, the reaction mixture was concentrated in vacuo and the residue was either crystallized or extracted and purified by flash column chromatography.

**General procedure C.** Reductive amination with pyrazol-5-amines. If not explicitly described otherwise, molecular sieve (3Å), the respective aldehyde (1.50 eq.) and AcOH (1.00 eq.) were added to a solution of the respective aminopyrazole (1.00 eq.) in dry EtOH. The reaction mixture was stirred at rt for 24 h. At 0 °C, NaBH_4_ (6.00 eq.) was added, and the reaction mixture was stirred at rt for 16 h and then quenched with H_2_O. The suspension was filtered over Celite**^®^** and washed with CH_3_OH (1×), EtOAc (2×), aqueous HCl (4M) and H_2_O (1×). The organic layer was washed with aqueous HCl (4M, 3×) and the combined aqueous layers were neutralized using aqueous KOH (8M). The aqueous layer was extracted with EtOAc (5×), the combined organic layers were dried (Na_2_SO_4_), filtered, concentrated in vacuo, and the residue was purified by flash column chromatography.

**General procedure D.** Einhorn acylation using acid chlorides. If not explicitly described otherwise, respective aminotriazole or aminopyrazole (1.00 eq.) was suspended in dry pyridine/dry THF mixture at 0 °C. A solution of respective acid chloride (1.00–1.40 eq.) in dry THF (1 mL) was added to this suspension dropwise via a syringe pump (1 mL/h) and afterwards, the suspension was stirred for 1 h at rt. The reaction mixture was quenched with H_2_O. If a precipitate was formed, it was filtered off, washed with H_2_O (3×) and dried in vacuo. Otherwise, the aqueous layer was extracted with EtOAc (3×), the combined organic layers were dried (N_2_SO_4_), filtered and concentrated in vacuo. The residue was purified by flash column chromatography.

**General procedure E.** Under N_2_, an azole exhibiting the 2-iodobenzoyl moiety (1.00 eq.), CuI (0.20 eq.), Cs_2_CO_3_ (2.00 eq.) and 1,10-phenanthroline (0.20 eq.) were dissolved in dry DMF. The reaction mixture was stirred at 80 °C until TLC analysis indicated the complete consumption of the azole bearing 2-iodobenzoyl moiety. After cooling, the suspension was diluted with H_2_O (30 mL) and extracted with EtOAc (3 × 50 mL). The combined organic layers were dried (Na_2_SO_4_) and concentrated under reduced pressure. The crude product was purified by flash column chromatography.

**General procedure F.** If not explicitly described otherwise, the respective acylated 1,2,4-triazol-5-amine (1.00 eq.), CuI (0.20 eq.), Cs_2_CO_3_ (2.00 eq.) and 1,10-phenanthroline (0.20 eq.) were dissolved in dry DMF (2 mL) at room temperature. The reaction mixture was flushed with N_2_ and was subjected to microwave irradiation (150 °C, 1 h, max 300 W). After the heated mixture was cooled to room temperature, the solvent was evaporated under reduced pressure. The crude product was purified by flash column chromatography (CH_2_Cl_2_/CH_3_OH = 97/3 or 100/0 → 90/10).

*sodium 2-cyano-1-(pyridin-3-yl)ethen-1-olate* (**6a**). According to general procedure A, NaH (2.59 g, 64.8 mmol), ester **5a** (9.01 mL, 66.2 mmol) and dry CH_3_CN (3.39 mL, 64.8 mmol) were reacted in dry THF (110 mL) yielding sodium enolate **6a** as a beige solid (9.84 g, 58.5 mmol, 88%). M.p.: >300 °C. TLC: Rf = 0.37 (CH_2_Cl_2_/CH_3_OH = 95/5). ^1^HNMR (600 MHz, DMSO-*d*_6_): *δ* (ppm) = 4.03 (s, 1H, CH), 7.27 (ddd, *J* = 7.8/4.7/0.9 Hz, 1H, 5-H_pyridyl_), 7.93 (dt, *J* = 7.9/2.0 Hz, 1H, 4-H_pyridyl_), 8.43 (dd, *J* = 4.7/1.7 Hz, 1H, 6-H_pyridyl_), 8.78 (dd, *J* = 2.3/0.9 Hz, 1H, 2-H_pyridyl_). The signals of the major isomer are given. ^13^C NMR (151 MHz, DMSO-*d*_6_): *δ* (ppm) = 52.7 (1C, CH), 122.8 (1C, C-5_pyridyl_), 126.8 (1C, CN), 133.0 (1C, C-4_pyridyl_), 137.6 (1C, C-3_pyidyl_), 147.3 (1C, C-2_pyridyl_), 148.8 (1C, C-6_pyridyl_), 177.4 (1C, CONa). The signals of the major isomer are given. IR (neat): *ṽ* [cm^−1^] = 2167, 1593, 1581, 1527, 1442, 1408, 1091, 1026, 1010, 821, 717, 694. HRMS (APCI): *m*/*z* = 147.0553 calculated for [C_8_H_6_N_2_O+H]^+^, found: 147.0553.

*sodium 2-cyano-1-phenylethen-1-olate* (**6b**). According to general procedure A, NaH (261 mg, 6.53 mmol), ester **5b** (952 μL, 6.66 mmol) and dry CH_3_CN (341 μL, 6.53 mmol) were reacted in dry THF (11 mL) yielding sodium enolate **6b** as a colorless solid (837 mg, 5.01 mmol, 75%). M.p.: 266–268 °C (decomp.). TLC: *R*_f_ = 0.37 (CH/EtOAc = 60/40). ^1^H NMR (600 MHz, DMSO-*d*_6_): *δ* (ppm) = 3.96 (s, 1H, CH), 7.22–7.25 (m, 3H, 2/4/6-H_phenyl_), 7.58–7.62 (m, 2H, 3/5-H_phenyl_). The signals of the major isomer are given. ^13^C NMR (151 MHz, DMSO-*d*_6_): *δ* (ppm) = 51.5 (1C, CH), 125.6 (2C, C-3/5_phenyl_), 127.3 (2C, C-2/6_phenyl_), 127.6 (1C, CN), 127.9 (1C, C-4_phenyl_), 143.1 (1C, C-1_phenyl_), 179.9 (1C, CONa). The signals of the major isomer are given. IR (neat): *ṽ* [cm^−1^] = 3271, 3213, 2164, 1550, 1508, 1481, 1442, 1404, 1226, 1002, 879, 694. HRMS (APCI): *m*/*z* = 146.0600 calculated for [C_9_H_7_NO+H]^+^, found: 146.0611.

*sodium 2-cyano-1-cyclohexylethen-1-olate* (**6c**). According to general procedure A, NaH (2.76 g, 68.9 mmol), ester **5c** (10.0 g, 70.3 mmol) and dry CH_3_CN (3.6 mL, 68.9 mmol) were reacted in dry THF (120 mL) yielding sodium enolate **6c** as a colorless solid (8.82 g, 72%). %). M.p.: >300 °C. TLC: *R*_f_ = 0.29 (EtOAc/CH = 20/80). ^1^H-NMR (600 MHz, DMSO-*d_6_*): *δ* (ppm) = 1.81–1.70 (*m*, 1H, H_cyclohexyl_); 1.68–1.50 (*m*, 4H, H_cyclohexyl_); 1.36–1.00 (*m*, 6H, H_cyclohexyl_), the signal of H_enolate_ cannot be seen on the spectrum. ^13^C-NMR (151 MHz, DMSO-*d_6_*): *δ* (ppm) = 179.9 (1C, *C*-ONa); 128.2 (1C, *C*≡N); 46.0 (1C, C_cyclohexyl_); 30.3 (2C, C_cyclohexyl_); 26.2 (1C, C_yclohexyl_); 25.9 (2C, C_cyclohexyl_), the signal of C_enolate_ cannot be seen on the spectrum. IR (neat): *ṽ* [cm^−1^] = 2926, 2851, 2166, 1560, 1537, 1501, 1443, 1414, 1356, 1281, 945, 773, 712. HRMS (APCI): *m*/*z* = 152.1074, calculated for C_9_H_14_NO^+^ [M + H]^+^ 152.1070.

*3-(pyridin-3-yl)-1H-pyrazol-5-amine* (**7a**). According to general procedure B, β-ketonitrile sodium enolate **6a** (8.00 g, 47.2 mmol, 1.00 eq.) was dissolved in aqueous HCl (120 mL, 1M), followed by work-up as described above. The orange oily residue of the keto-form of β-ketonitrile **6a** and NH_2_NH_2_·H_2_O (6.94 mL, 143 mmol, 3.00 eq.) were reacted in EtOH (87 mL). Then, the obtained residue was crystallized using EtOH and cyclohexane. The precipitate was collected by filtration, washed with cold EtOH (2×), and dried in vacuo yielding 4.68 g of **7a**, that was used without further purification. The filtrate was concentrated in vacuo and purified by flash column chromatography (CH_2_Cl_2_/CH_3_OH = 1/0 → 90/10) yielding 1.00 g of **7a** (combined 5.68 g, 35.5 mmol, 75%) as a beige solid. M.p.: 145 °C. TLC: *R*_f_ = 0.30 (CH_2_Cl_2_/CH_3_OH = 95/5). ^1^H NMR (600 MHz, DMSO-*d*_6_): *δ* (ppm) = 4.96 (bs, 2H, NH_2_), 5.82 (bs, 1H, 4-H_pyrazolyl_), 7.38 (dd, *J* = 7.9/4.8 Hz, 1H, 5-H_pyridyl_), 8.01 (dt, *J* = 8.0/2.0 Hz, 1H, 4-H_pyridyl_), 8.45 (dd, *J* = 4.8/1.7 Hz, 1H, 6-H_pyridyl_), 8.88 (dd, *J* = 2.3/0.9 Hz, 1H, 2-H_pyridyl_), 11.81 (bs, 1H, NH). ^13^C NMR (151 MHz, DMSO-*d*_6_): *δ* (ppm) = 123.7 (1C, C-5_pyridyl_), 131.8 (1C, C-4_pyridyl_), 146.0 (1C, C-2_pyridyl_), 148.1 (1C, C-6_pyridyl_). The signals for C-3_pyridyl_ and C-3/4/5_pyrazolyl_ are not seen in the spectrum. IR (neat): *ṽ* [cm^−1^] = 3390, 3290, 3194, 3116, 3047, 2858, 1631, 1612, 1573, 1516, 1469, 1192, 995, 956, 810, 771, 736, 702, 671. HRMS (APCI): *m*/*z* = 161.0822 calculated for [M + H]^+^, found: 161.0841. HPLC: *t*_R_ = 3.3 min, purity: 99.8%.

*3-phenyl-1H-pyrazol-5-amine* (**7b**). According to general procedure B, β-ketonitrile keto-form **6b** (500 mg, 3.44 mmol) and NH_2_NH_2_·H_2_O (345 µL, 6.89 mmol, 2.00 eq.) were reacted in EtOH (6.20 mL). The reaction mixture was quenched with H_2_O, the aqueous phase was extracted with EtOAc (3×), the combined organic layers were dried (Na_2_SO_4_), filtrated and concentrated in vacuo. The residue was purified by flash column chromatography (CH_2_Cl_2_/CH_3_OH = 1/0 → 91/9) yielded **7b** as a yellow solid (515 mg, 3.24 mmol, 94%). M.p.: 128 °C. TLC: *R*_f_ = 0.14 (CH_2_Cl_2_/CH_3_OH = 95/5). ^1^H NMR (600 MHz, DMSO-*d*_6_): *δ* (ppm) = 4.70 (bs, 2H, NH_2_), 5.74 (bs, 1H, 4-H_pyrazolyl_), 7.19–7.29 (m, 1H, 4-H_phenyl_), 7.32–7.40 (m, 2H, 3/5-H_phenyl_), 7.61–7.69 (m, 2H, 2/6-H_phenyl_), 11.68 (bs, 1H, NH). ^13^C NMR (151 MHz, DMSO-*d*_6_): *δ* (ppm) = 124.7 (2C, C-2/6_phenyl_), 127.2 (1C, C-4_phenyl_), 128.6 (2C, C-3/5_phenyl_). The signals for C-1_phenyl_ and C-3/4/5_pyrazolyl_ are not seen in the spectrum. IR (neat): *ṽ* [cm^−1^] = 3398, 3294, 3197, 1620, 1562, 1504, 1465, 1095, 1076, 1056, 995, 956, 918, 759, 694. HRMS (APCI): *m*/*z* = 160.0869 calculated for [M + H]^+^, found: 160.0874. HPLC: *t*_R_ = 11.1 min, purity: 99.7%.

*3-cyclohexyl-1H-pyrazol-5-amine* (**7c**). According to general procedure B, β-ketonitrile sodium enolate **6c** (6.92 g, 40 mmol, 1.00 eq.) was dissolved in aqueous HCl (120 mL, 1M), followed by work-up as described above. The residue of the keto-form of β-ketonitrile **6c** and NH_2_NH_2_·H_2_O (4.0 mL, 79.9 mmol, 2.00 eq.) were reacted in EtOH (80 mL). The product was purified by flash column chromatography (CH_2_Cl_2_/CH_3_OH = 1/0 → 90/10) yielding **7b** as a reddish oil (5.01 g, 76%). TLC: *R*_f_ = 0.17 (CH_2_Cl_2_/CH_3_OH = 95/5). ^1^H-NMR (600 MHz, DMSO-*d_6_*): *δ* (ppm) = 11.02 (*bs*, 1H, N*H*_pyrazolyl_); 5.15 (*s*, 1H, 4-H_pyrazolyl_); 4.39 (*bs*, 2H, N*H*_2_); 2.21 (*tdt*, *J* = 12.0/8.4/4.2 Hz, 1H, H_cyclohexyl_); 1.88–1.75 (*m*, 2H, H_cyclohexyl_); 1.75–1.66 (*m*, 2H, H_cyclohexyl_); 1.66–1.60 (*m*, 1H, H_cyclohexyl_); 1.35–1.23 (*m*, 4H, H_cyclohexyl_); 1.22–1.10 (*m*, 1H, H_cyclohexyl_). ^13^C-NMR (151 MHz, DMSO-*d_6_*): *δ* (ppm) = 153.6 (1C, C-5_pyrazolyl_); 149.9 (1C, C-3_pyrazolyl_); 87.9 (1C, C-4_pyrazolyl_); 35.2 (1C, C_cyclohexyl_); 32.3 (2C, C_cyclohexyl_); 25.6 (1C, C_yclohexyl_); 25.5 (2C, C_cyclohexyl_). IR (neat): *ṽ* [cm^−1^] = 3206, 2922, 2851, 1684, 1574, 1485, 1447, 1373, 1001, 986, 891, 758. HRMS (APCI): *m*/*z* = 166.1328, calculated for C_9_H_16_N_3_^+^ [M + H]^+^ 166.1339. HPLC: *t*_R_ = 12.9 min, purity: 96.2%.

*N-benzyl-3-(pyridin-3-yl)-1H-pyrazol-5-amine* (**8a**). According to general procedure C, aminopyrazole **7a** (100 mg, 624 µmol), benzaldehyde (95.6 µL, 936 µmol) and AcOH (35.7 µL, 624 µmol) were reacted in EtOH (3.0 mL). After reduction with NaBH_4_ (142 mg, 3.75 mmol) and flash column chromatography (CH_2_Cl_2_/CH_3_OH = 1/0 → 90/10) **8a** was obtained as a colorless solid (137 mg, 547 µmol, 88%). M.p.: 135–136 °C. TLC: *R*_f_ = 0.30 (CH_2_Cl_2_/CH_3_OH = 93/7). ^1^H NMR (600 MHz, DMSO-*d*_6_): *δ* (ppm) = 4.27 (d, *J* = 6.4 Hz, 2H, CH_2_), 5.82 (bs, 2 × 0.50H, 4-H_pyrazolyl_, N*H*CH_2_), 6.03 (bs, 0.50H, 4-H_pyrazolyl_), 6.21 (bs, 0.5H, N*H*CH_2_), 7.17–7.25 (m, 1H, 4-H_phenyl_), 7.27–7.34 (m, 2H, 3/5-H_phenyl_), 7.35–7.45 (m, 3H, 2/6-H_phenyl_, 5-H_pyridyl_), 8.00 (d, *J* = 8.1 Hz, 1H, 4-H_pyridyl_), 8.39–8.50 (m, 1H, 6-H_pyridyl_), 8.84–8.89 (m, 1H, 2-H_pyridyl_), 11.88 (bs, 0.50H, NH), 12.14 (bs, 0.50H, NH). The ratio of tautomers is 1:1. ^13^C NMR (151 MHz, DMSO-*d*_6_): *δ* (ppm) = 48.0 (1C, CH_2_), 83.5 (0.5C, C-4_pyrazolyl_), 88.9 (0.5C, C-4_pyrazolyl_), 123.8 (1C, C-5_pyridyl_), 126.8 (1C, C-4_phenyl_), 127.4 (2C, C-2/6_phenyl_), 128.1 (2C, C-3/5_phenyl_), 131.8 (1C, C-4_pyridyl_), 139.1 (1C, C-3_pyridyl_), 140.0 (0.5C, C-1_phenyl_), 141.3 (0.5C, C-1_phenyl_), 146.0 (1C, C-2_pyridyl_), 148.5 (1C, C-6_pyridyl_), 157.8 (1C, C-5_pyrazolyl_). The signals of the major tautomer are given, except of C-4_pyrazolyl_. The signal for C-3_pyrazolyl_ is not seen in the spectrum. IR (neat): *ṽ* [cm^−1^] = 3209, 3132, 3062, 2943, 2839, 2719, 1585, 1573, 1446, 1361, 1219, 1141, 1118, 1029, 956, 810, 736, 702. HRMS (APCI): *m*/*z* = 251.1291 calculated for [M + H]^+^, found: 251.1313. HPLC: *t*_R_ = 13.2 min, purity: 99.6%.

*N-(4-methoxybenzyl)-3-(pyridin-3-yl)-1H-pyrazol-5-amine* (**8b**). According to general procedure C, aminopyrazole **7a** (600 mg, 3.75 mmol, 1.00 eq.), 4-methoxybenzaldehyde (683 µL, 5.62 mmol) and AcOH (214 µL, 3.75 mmol) were reacted in EtOH (19.0 mL). After reduction with NaBH_4_ (6.00 eq.) and flash column chromatography (CH_2_Cl_2_/CH_3_OH = 94/6 → 90/10) **8b** was obtained as a beige solid (731 mg, 70%). M.p.: 134 °C. TLC: *R*_f_ = 0.33 (CH_2_Cl_2_/CH_3_OH = 94/6). ^1^H-NMR (600 MHz, DMSO-*d_6_*): *δ* (ppm) = 11.98 (*bs*, 1H, N*H*_pyrazolyl_); 8.87 (*dd*, *J* = 2.3/0.9 Hz, 1H, 2-H_pyridyl_); 8.45 (*dd*, *J* = 4.7/1.6 Hz, 1H, 6-H_pyridyl_); 8.00 (*ddd*, *J* = 7.9/2.3/1.7 Hz, 1H, 4-H_pyridyl_); 7.39 (*dd*, *J* = 8.0/4.8 Hz, 1H, 5-H_pyridyl_); 7.33–7.25 (*m*, 2H, 2/6-H_methoxyphenyl_); 6.92–6.83 (*m*, 2H, 3/5-H_methoxyphenyl_); 5.91 (*s*, 1H, 4-H_pyrazolyl_); 4.18 (*d*, *J* = 6.3 Hz, 2H, C*H*_2_); 3.72 (*s*, 3H, C*H*_3_), the signal of N*H* cannot be seen on the spectrum. ^13^C-NMR (151 MHz, DMSO-*d_6_*): *δ* (ppm) = 158.0 (1C, C-4_methoxyphenyl_); 148.1 (1C, C-6_pyridyl_); 146.0 (1C, C-2_pyridyl_); 132.3 (1C, C-1_methoxyphenyl_); 131.8 (1C, C-4_pyridyl_); 130.5 (1C, C-3_pyridyl_); 128.6 (2C, C-2/6_methoxyphenyl_); 123.6 (1C, C-5_pyridyl_); 113.5 (2C, C-3/5_methoxyphenyl_); 54.9 (1C, *C*H_3_); 47.2 (1C, *C*H_2_), the signals of C-3_pyridyl_, C-3_pyrazolyl_ and C-5_pyrazolyl_ cannot be seen on the spectrum. IR (neat): *ṽ* [cm^−1^] = 3240, 3213, 3132, 2835, 1603, 1574, 1514, 1462, 1441, 1398, 1302, 1244, 1180, 1118, 1084, 1030, 959, 864, 824, 804, 752. HRMS (APCI): *m*/*z* = 281.1380, calculated for C_16_H_17_N_4_O^+^ [M + H]^+^ 281.1397. HPLC: *t*_R_ = 13.4 min, purity: 99.7%.

*N-(naphthalen-1-ylmethyl)-3-(pyridin-3-yl)-1H-pyrazol-5-amine* (**8c**). According to general procedure C, aminopyrazole **7a** (600 mg, 3.75 mmol, 1.00 eq.), 1-naphthaldehyde (763 µL, 5.62 mmol) and AcOH (214 µL, 3.75 mmol) were reacted in EtOH (19.0 mL). After reduction with NaBH_4_ (6.00 eq.) and flash column chromatography (CH_2_Cl_2_/CH_3_OH = 100/0 → 90/10) **8c** was obtained as a beige solid (772 mg, 69%). M.p.: 78 °C. TLC: *R*_f_ = 0.29 (CH_2_Cl_2_/CH_3_OH = 94/6). ^1^H-NMR (400 MHz, DMSO-*d_6_*): *δ* (ppm) = 12.05 (*bs*, 1H, N*H*_pyrazolyl_); 8.89 (*dd*, *J* = 2.3/0.9 Hz, 1H, 2-H_pyridyl_); 8.46 (*dd*, *J* = 4.8/1.6 Hz, 1H, 6-H_pyridyl_); 8.18 (*d*, *J* = 8.3 Hz, 1H, 9-H_naphthyl_); 8.02 (*ddd*, *J* = 8.0/2.3/1.6 Hz, 1H, 4-H_pyridyl_); 7.96–7.92 (*m*, 1H, 6-H_naphthyl_); 7.83 (*d*, *J* = 8.2 Hz, 1H, 4-H_naphthyl_); 7.60–7.55 (*m*, 2H, 7/8-H_naphthyl_); 7.55–7.52 (*m*, 1H, 2-H_naphthyl_); 7.46 (*dd*, *J* = 8.2/7.0 Hz, 1H, 3-H_naphthyl_); 7.40 (*ddd*, *J* = 7.9/4.8/0.9 Hz, 1H, 5-H_pyridyl_); 6.00 (*s*, 1H, 4-H_pyrazolyl_); 4.74 (*d*, *J* = 5.9 Hz, 2H, C*H*_2_), the signal of N*H* cannot be seen on the spectrum. ^13^C-NMR (101 MHz, DMSO-*d_6_*): *δ* (ppm) = 146.0 (1C, C-2_pyridyl_); 131.8 (1C, C-4_pyridyl_); 133.3 (1C, C-10_naphthyl_); 131.1 (1C, C-5_naphthyl_); 128.4 (1C, C-6_naphthyl_); 127.2 (1C, C-4_naphthyl_); 125.9 (1C, C-7_naphthyl_); 125.6 (1C, C-2_naphthyl_); 125.3 (1C, C-3_naphthyl_); 125.1 (1C, C-8_naphthyl_); 123.7 (1C, C-5_pyridyl_); 123.6 (1C, C-9_naphthyl_); 45.5 (1C, *C*H_2_), the signals of C-1_naphthyl_, C-4_naphthyl_, C-6_pyridyl_, C-3_pyridyl_, C-3_pyrazolyl_ and C-5_pyrazolyl_ cannot be seen on the spectrum. IR (neat): *ṽ* [cm^−1^] = 2980, 2970, 2887, 1597, 1566, 1546, 1512, 1503, 1462, 1441, 1382, 1261, 1163, 1086, 1072, 1024, 955, 791, 752, 706. HRMS (APCI): *m*/*z* = 301.1477, calculated for C_19_H_17_N_4_^+^ [M + H]^+^ 301.1448. HPLC: *t*_R_ = 15.9 min, purity: 99.3%.

*N-(furan-2-ylmethyl)-3-(pyridin-3-yl)-1H-pyrazol-5-amine* (**8d**). According to general procedure C, aminopyrazole **7a** (600 mg, 3.75 mmol, 1.00 eq.), furaldehyde (465 µL, 5.62 mmol) and AcOH (214 µL, 3.75 mmol) were reacted in EtOH (19.0 mL). After reduction with NaBH_4_ (6.00 eq.) and flash column chromatography (CH_2_Cl_2_/CH_3_OH = 94/6) **8d** was obtained as a beige solid (687 mg, 76%). M.p.: 170 °C. TLC: *R*_f_ = 0.34 (CH_2_Cl_2_/CH_3_OH = 94/6). ^1^H-NMR (600 MHz, DMSO-*d_6_*): *δ* (ppm) = 12.06 (*bs*, 1H, N*H*_pyrazolyl_); 8.89 (*dd*, *J* = 2.3/0.9 Hz, 1H, 2-H_pyridyl_); 8.46 (*dd*, *J* = 4.8/1.7 Hz, 1H, 6-H_pyridyl_); 8.02 (*ddd*, *J* = 7.9/2.3/1.7 Hz, 1H, 4-H_pyridyl_); 7.56 (*dd*, *J* = 1.9/0.9 Hz, 1H, 5-H_furanyl_); 7.40 (*dd*, *J* = 8.0/4.8 Hz, 1H, 5-H_pyridyl_); 6.37 (*dd*, *J* = 3.2/1.9 Hz, 1H, 4-H_furanyl_); 6.30 (*dd*, *J* = 3.2 Hz, 1H, 3-H_furanyl_); 5.98 (*s*, 1H, 4-H_pyrazolyl_); 5.89 (*s*, 1H, N*H*); 4.24 (*dd*, *J* = 6.3/0.9 Hz, 2H, C*H*_2_). ^13^C-NMR (151 MHz, DMSO-*d_6_*): *δ* (ppm) = 153.6 (1C, C-2_furanyl_); 148.2 (1C, C-6_pyridyl_); 146.0 (1C, C-2_pyridyl_); 141.7 (1C, C-5_furanyl_); 131.8 (1C, C-4_pyridyl_); 123.7 (1C, C-5_pyridyl_); 110.3 (1C, C-4_furanyl_); 106.7 (1C, C-3_furanyl_); 41.0 (1C, *C*H_2_), the signals of C-3_pyridyl_, C-3_pyrazolyl_, C-4_pyrazolyl_ and C-5_pyrazolyl_ cannot be seen on the spectrum. IR (neat): *ṽ* [cm^−1^] = 3194, 1601, 1587, 1573, 1503, 1445, 1352, 1188, 1118, 1080, 1032, 957, 916, 881, 810, 758, 741, 723. HRMS (APCI): *m*/*z* = 241.1086, calculated for C_13_H_13_N_4_O^+^ [M + H]^+^ 241.1084. HPLC: *t*_R_ = 11.1 min, purity: 99.1%.

*N-[(5-chlorothiophen-2-yl)methyl]-3-(pyridin-3-yl)-1H-pyrazol-5-amine* (**8e**). According to general procedure C, aminopyrazole **7a** (100 mg, 624 µmol), 5-chlorothiophene-2-carbaldehyde (99.5 µL, 936 µmol) and AcOH (35.7 µL, 624 µmol) were reacted in EtOH (3.0 mL). After reduction with NaBH_4_ (142 mg, 3.75 mmol) and flash column chromatography (CH_2_Cl_2_/CH_3_OH = 1/0 → 90/10) **8e** was obtained as a colorless solid (142 mg, 488 µmol, 78%). M.p.: 158–159 °C. TLC: *R*_f_ = 0.32 (CH_2_Cl_2_/CH_3_OH = 93/7). ^1^H NMR (600 MHz, DMSO-*d*_6_): *δ* (ppm) = 4.37 (d, *J* = 6.4 Hz, 2H, CH_2_), 5.96 (bs, 0.7H, N*H*CH_2_), 6.04 (bs, 1H, 4-H_pyrazolyl_), 6.28 (bs, 0.3H, N*H*CH_2_*), 6.84–6.91 (m, 1H, 4-H_thiophenyl_), 6.91–6.97 (m, 1H, 3-H_thiophenyl_), 7.35–7.45 (m, 1H, 5-H_pyridyl_), 8.02 (dt, *J* = 8.0/2.0 Hz, 1H, 4-H_pyridyl_), 8.43–8.52 (m, 1H, 6-H_pyridyl_), 8.89 (d, *J* = 2.4 Hz, 1H, 2-H_pyridyl_), 11.97 (bs, 0.3H, NH*), 12.26 (bs, 0.7H, NH). The ratio of tautomers is 7:3, the minor tautomer is marked with an asterisk (*). ^13^C NMR (151 MHz, DMSO-*d*_6_): *δ* (ppm) = 43.0 (1C, CH_2_), 89.2 (1C, C-4_pyrazolyl_), 123.8 (1C, C-5_pyridyl_), 124.4 (1C, C-4_thiophenyl_), 126.1 (1C, C-3_thiophenyl_), 131.9 (1C, C-4_pyridyl_), 139.4 (1C, C-3_pyridyl_), 146.0 (1C, C-2_thiophenyl_), 146.0 (1C, C-2_pyridyl_), 148.5 (1C, C-6_pyridyl_), 157.0 (1C, C-5_pyrazolyl_). The signals of the major tautomer are given. The signals for C-3_pyrazolyl_ and C-5_thiophenyl_ are seen in the spectrum. IR (neat): *ṽ* [cm^−1^] = 3278, 3059, 2816, 1562, 1535, 1489, 1450, 1419, 1354, 1099, 991, 956, 786, 756, 702, 632. HRMS (APCI): *m*/*z* = 291.0466 calculated for [M + H]^+^, found: 291.0463. HPLC: *t*_R_ = 14.6 min, purity: 99.2%.

*N-(cyclohexylmethyl)-3-(pyridin-3-yl)-1H-pyrazol-5-amine* (**8f**). According to general procedure C, aminopyrazole **7a** (250 mg, 1.56 mmol) and cyclohexane carbaldehyde (284 µL, 2.34 mmol) were reacted in EtOH (7.50 mL). No AcOH was added. After reduction with NaBH_4_ (354 mg, 9.36 mmol) and flash column chromatography (CH_2_Cl_2_/CH_3_OH = 1/0 → 9/1) **8f** was obtained as a colorless solid (294 mg, 1.15 mmol, 73%). M.p.: 124 °C. TLC: *R*_f_ = 0.26 (CH_2_Cl_2_/CH_3_OH = 93/7). ^1^H NMR (600 MHz, DMSO-*d*_6_): *δ* (ppm) = 0.86-0.95 (m, 2H, 2/6-H_cyclohexyl_), 1.10–1.24 (m, 3H, 3/4/5-H_cyclohexyl_), 1.48–1.56 (m, 1H, 1-H_cyclohexyl_), 1.59–1.64 (m, 1H, 4-H_cyclohexyl_), 1.65–1.71 (m, 2H, 3/5-H_cyclohexyl_), 1.74–1.79 (m, 2H, 2/6-H_cyclohexyl_), 2.88 (t, *J* = 6.5 Hz, 1H, CH_2_), 5.17 (bs, 0.4H, N*H*CH_2_*), 5.61 (bs, 0.6H, N*H*CH_2_), 5.77 (bs, 0.6H, 4-H_pyrazolyl_), 5.97 (bs, 0.4H, 4-H_pyrazolyl_*), 7.32–7.46 (m, 1H, 5-H_pyridyl_), 8.02 (d, *J* = 7.9 Hz, 1H, 4-H_pyridyl_), 8.40–8.49 (m, 1H, 6-H_pyridyl_), 8.84–8.94 (m, 1H, 2-H_pyridyl_), 11.66 (bs, 0.6H, NH), 12.06 (bs, 0.4H, NH*). The ratio of tautomers is 6:4, the minor tautomer is marked with an asterisk (*). ^13^C NMR (151 MHz, DMSO-*d*_6_): *δ* (ppm) = 25.5 (2C, C-3/5_cyclohexyl_), 26.2 (1C, C-4_cyclohexyl_), 30.6 (2C, C-2/6_cyclohexyl_), 37.3 (1C, C-1_cyclohexyl_), 50.9 (1C, CH_2_), 82.7 (0.6C, C-4_pyrazolyl_), 88.5 (0.4C, C-4_pyrazolyl_), 123.6 (1C, C-5_pyridyl_), 131.8 (1C, C-5_pyridyl_), 146.0 (1C, C-5_pyridyl_), 147.9 (1C, C-6_pyridyl_), 151.4 (1C, C-3_pyrazolyl_), 158.4 (1C, C-5_pyrazolyl_). The signals of the major tautomer are given, except of C-4_pyrazolyl_. IR (neat): *ṽ* [cm^−1^] = 2920, 2843, 1593, 1442, 1188, 1145, 1118, 1029, 960, 806, 736, 705. HRMS (APCI): *m*/*z* = 257.1761 calculated for [M + H]^+^, found: 257.1746. HPLC: *t*_R_ = 15.4 min, purity: 98.1%.

*N-[(5-chlorothiophen-2-yl)methyl]-3-phenyl-1H-pyrazol-5-amine* (**8g**). According to general procedure C, aminopyrazole **7b** (200 mg, 1.26 mmol, 1.00 eq.), 5-chlorothiophene-2-carbaldehyde (200 µL, 1.88 mmol) and AcOH (72 µL, 1.26 mmol) were reacted in EtOH (6.3 mL). After reduction with NaBH_4_ (6.00 eq.) and flash column chromatography (CH_2_Cl_2_/CH_3_OH = 1/0 → 9/1) **8g** was obtained as a beige solid (288 mg, 79%). M.p.: 84–85 °C. TLC: *R*_f_ = 0.48 (CH_2_Cl_2_/CH_3_OH = 95/5). ^1^H-NMR (600 MHz, DMSO-*d_6_*): *δ* (ppm) = 12.01 (*bs*, 1H, N*H*_pyrazolyl_); 7.64 (*m*, 2H, H_phenyl_); 7.39 (*m*, 2H, H_phenyl_); 7.29 (*m*, 1H, H_phenyl_); 6.92 (*d*, J = 3.7 Hz, 1H, 3-H_chlorothiophenyl_); 6.89 (*d*, J = 3.7 Hz, 1H, 4-H_chlorothiophenyl_); 5.93 (*bs*, 1H, N*H*); 5.90 (*bs*, 1H, 4-H_pyrazolyl_); 4.37 (*d*, J = 5.5 Hz, 2H, C*H*_2_). ^13^C-NMR (151 MHz, DMSO-*d_6_*): *δ* (ppm) = 144.3 (1C, C-2_chlorothiophenyl_); 128.6 (2C, C_phenyl_); 127.5 (1C, C_phenyl_); 126.1 (1C, C-4_chlorothiophenyl_); 124.6 (2C, C_phenyl_); 124.4 (1C, C-3_chlorothiophenyl_); 87.4 (1C, C-4_pyrazolyl_); 43.1 (1C, *C*H_2_), the signals of C-5_chlorothiophenyl_, C-3_pyridyl_, C-3_pyrazolyl_ and C-5_pyrazolyl_ cannot be seen on the spectrum. IR (neat): *ṽ* [cm^−1^] = 3671, 2361, 1690, 1599, 1570, 1539, 1489, 1460, 1435, 1184, 1061, 1007, 930, 853, 746. HRMS (APCI): *m*/*z* = 290.0469, calculated for C_14_H_13_ClN_3_S^+^ [M + H]^+^ 290.0513. HPLC: *t*_R_ = 18.2 min, purity: 96.7%.

*N-benzyl-3-cyclohexyl-1H-pyrazol-5-amine* (**8h**). According to general procedure C, aminopyrazole **7c** (500 mg, 3.03 mmol), benzaldehyde (200 µL, 4.54 mmol) and AcOH (460 µL, 3.03 mmol) were reacted in EtOH (15 mL). After reduction with NaBH_4_ (6.00 eq.) and flash column chromatography (CH_2_Cl_2_/CH_3_OH = 1/0 → 9/1) **8h** was obtained as a beige oil (297 mg, 38%). TLC: *R*_f_ = 0.20 (CH_2_Cl_2_/CH_3_OH = 95/5). ^1^H-NMR (600 MHz, DMSO-*d_6_*): *δ* (ppm) = 7.36–7.32 (*m*, 2H, 2/6-H_phenyl_); 7.31–7.26 (*m*, 2H, 3/5-H_phenyl_); 7.22–7.16 (*m*, 1H, 4-H_phenyl_); 5.25 (*s*, 1H, 4-H_pyrazolyl_); 4.19 (*s*, 2H, C*H*_2_); 2.44 (*dd*, *J* = 8.9/5.5 Hz, 1H, H_cyclohexyl_); 1.89–1.79 (*m*, 2H, H_cyclohexyl_); 1.78–1.68 (*m*, 2H, H_cyclohexyl_); 1.67–1.59 (*m*, 1H, H_cyclohexyl_); 1.34–1.22 (*m*, 4H, H_cyclohexyl_); 1.22–1.09 (*m*, 1H, H_cyclohexyl_), the signals of N*H*_pyrazolyl_ and N*H* cannot be seen on the spectrum. ^13^C-NMR (151 MHz, DMSO-*d_6_*): *δ* (ppm) = 141.6 (1C, C-1_phenyl_); 128.5 (2C, C-3/5_phenyl_); 127.8 (2C, C-2/6_phenyl_); 126.8 (1C, C-4_phenyl_); 87.5 (1C, C-4_pyrazolyl_); 48.1 (2C, *C*H_2_), 35.6 (1C, C_cyclohexyl_); 32.8 (2C, C_cyclohexyl_); 26.1 (1C, C_cyclohexyl_); 26.0 (2C, C_cyclohexyl_), the signals of C-3_pyrazolyl_, C-5_pyrazolyl_ cannot be seen on the spectrum. IR (neat): *ṽ* [cm^−1^] = 3225, 2924, 2851, 2361, 1738, 1628, 1574, 1495, 1449, 1354, 1117, 986, 891, 733. HRMS (APCI): *m*/*z* = 256.1832, calculated for C_16_H_22_N_3_^+^ [M + H]^+^ 256.1808.

*N-[(5-chlorothiophen-2-yl)methyl]-3-cyclohexyl-1H-pyrazol-5-amine* (**8i**). According to general procedure C, aminopyrazole **7c** (100 mg, 605 µmol), 5-chlorothiophene-2-carbaldehyde (95 µL, 908 µmol) and AcOH (35 µL, 605 µmol) were reacted in EtOH (3 mL). After reduction with NaBH_4_ (6.00 eq.) and flash column chromatography (CH_2_Cl_2_/CH_3_OH = 1/0 → 9/1) **8i** was obtained as a yellowish oil (77 mg, 43%). TLC: *R*_f_ = 0.28 (CH_2_Cl_2_/CH_3_OH = 95/5). ^1^H-NMR (600 MHz, DMSO-*d_6_*): *δ* (ppm) = 6.90 (*d*, *J* = 3.7 Hz, 1H, 4-H_chlorothiophenyl_); 6.83 (*d*, *J* = 3.7 Hz, 1H, 3-H_chlorothiophenyl_); 5.59 (*s*, 1H, N*H*); 5.25 (*s*, 1H, 4-H_pyrazolyl_); 4.28 (*d*, *J* = 6.0 Hz, 2H, C*H*_2_); 2.45 (*m*, 1H, H_cyclohexyl_); 2.48–2.41 (*m*, 1H, H_cyclohexyl_); 1.89–1.80 (*m*, 2H, H_cyclohexyl_); 1.771.68 (*m*, 2H, H_cyclohexyl_); 1.68–1.60 (*m*, 1H, H_cyclohexyl_); 1.34–1.25 (*m*, 4H, H_cyclohexyl_); 1.24–1.13 (*m*, 1H, H_cyclohexyl_), the signal of N*H*_pyrazolyl_ cannot be seen on the spectrum. ^13^C-NMR (151 MHz, DMSO-*d_6_*): *δ* (ppm) = 43.6 (1C, *C*H_2_); 35.5 (1C, C_cyclohexyl_); 32.8 (2C, C_cyclohexyl_); 26.1 (1C, C_cyclohexyl_); 26.0 (2C, C_cyclohexyl_), the signals of C-3_pyrazolyl_, C-4_pyrazolyl_, C-5_pyrazolyl_, C-2_chlorothiophenyl_, C-3_chlorothiophenyl_, C-4_chlorothiophenyl_, C-5_chlorothiophenyl_ cannot be seen on the spectrum. IR (neat): *ṽ* [cm^−1^] = 3227, 2924, 2851, 1738, 1574, 1520, 1506, 1449, 1206, 1115, 1059, 989, 891, 789. HRMS (APCI): *m*/*z* = 296.0982, calculated for C_14_H_19_ClN_3_S^+^ [M + H]^+^ 296.0983.

*[5-(benzylamino)-3-(pyridin-3-yl)-1H-pyrazol-1-yl](2-iodophenyl)methanone* (**9a**). According to general procedure D, aminopyrazole **8a** (409 mg, 1.63 mmol) was acylated using 2-iodobenzoyl chloride (522 mg, 1.96 mmol) in dry pyridine/THF (18 mL/6 mL). Flash column chromatography (CH/EtOAc = 1/0 → 0/1) yielded **9a** as a yellowish solid (505 mg, 64%). M.p.: 115 °C. TLC: *R*_f_ = 0.73 (CH/EtOAc = 1/1). ^1^H-NMR (600 MHz, DMSO-*d_6_*): *δ* (ppm) = 8.81 (*dd*, *J* = 2.2/0.9 Hz, 1H, 2-H_pyridyl_); 8.54 (*dd*, *J* = 4.8/1.7 Hz, 1H, 6-H_pyridyl_); 7.96–7.94 (*m*, 1H, 3-H_benzoyl_); 7.94–7.92 (*m*, 1H, 4-H_pyridyl_); 7.92–7.89 (*m*, 1H, N*H*); 7.59 (*dd*, *J* = 7.6/1.7 Hz, 1H, 6-H_benzoyl_); 7.54 (*td*, *J* = 7.5/1.1 Hz, 1H, 5-H_benzoyl_); 7.50–7.47 (*m*, 2H, 2/6-H_phenyl_); 7.41–7.39 (*m*, 1H, 5-H_pyridyl_); 7.37 (*dd*, *J* = 8.3/7.1 Hz, 2H, 3/5-H_phenyl_); 7.31–7.28 (*m*, 1H, 4-H_phenyl_); 7.27 (*dt*, *J* = 8.6/1.5 Hz, 1H, 4-H_benzoyl_); 6.10 (*s*, 1H, 4-H_pyrazolyl_); 4.51 (*d*, *J* = 6.1 Hz, 2H, C*H*_2_). ^13^C-NMR (151 MHz, DMSO-*d_6_*): *δ* (ppm) = 170.5 (1C, *C*=O); 153.3 (1C, C-5_pyrazolyl_); 152.3 (1C, C-3_pyrazolyl_); 150.0 (1C, C-6_pyridyl_); 147.0 (1C, C-2_pyridyl_); 140.6 (1C, C-1_benzoyl_); 138.6 (1C, C-1_phenyl_); 138.4 (1C, C-3_benzoyl_); 133.0 (1C, C-4_pyridyl_); 131.4 (1C, C-4_phenyl_); 128.7 (1C, C-6_benzoyl_); 128.4 (2C, C-3/5_phenyl_); 127.6 (1C, C-5_benzoyl_); 127.5 (1C, C-3_pyridyl_); 127.3 (2C, C-2/6_phenyl_); 127.0 (1C, C-4_benzoyl_); 123.7 (1C, C-5_pyridyl_); 93.6 (1C, C-2_benzoyl_); 83.8 (1C, C-4_pyrazolyl_); 47.8 (1C, *C*H_2_). IR (neat): *ṽ* [cm^−1^] = 3385, 1684, 1607, 1574, 1526, 1495, 1449, 1429, 1366, 1020, 937, 924, 727. HRMS (APCI): *m*/*z* = 481.0511, calculated for C_22_H_18_IN_4_O^+^ [M + H]^+^ 481.0520. HPLC: *t*_R_ = 19.8 min, purity: 93%.

*(2-iodophenyl){5-[(4-methoxybenzyl)amino]-3-(pyridin-3-yl)-1H-pyrazol-1-yl}methanone* (**9b**). According to general procedure D, aminopyrazole **8b** (500 mg, 1.78 mmol) was acylated using 2-iodobenzoyl chloride (570 mg, 2.14 mmol) in dry pyridine/THF (10 mL/20 mL). Flash column chromatography (CH/EtOAc = 1/0 → 0/1) yielded **9b** as a yellowish solid (484 mg, 53%). M.p.: 136–137 °C. TLC: *R*_f_ = 0.67 (CH/EtOAc = 1/1). ^1^H-NMR (400 MHz, DMSO-*d_6_*): *δ* (ppm) = 8.82 (*dd*, *J* = 2.2/0.9 Hz, 1H, 2-H_pyridyl_); 8.55 (*dd*, *J* = 4.8/1.7 Hz, 1H, 6-H_pyridyl_); 7.96–7.92 (*m*, 1H, 4-H_pyridyl_); 7.94–7.92 (*m*, 1H, 3-H_benzoyl_); 7.80 (*t*, *J* = 6.2 Hz, 1H, N*H*); 7.57 (*dd*, *J* = 7.7/1.7 Hz, 1H, 6-H_benzoyl_); 7.53 (*td*, *J* = 7.4/1.1 Hz, 1H, 5-H_benzoyl_); 7.43–7.42 (*m*, 2H, 2/6-H_methoxyphenyl_); 7.39 (*dd*, *J* = 4.8/0.9 Hz, 1H, 5-H_pyridyl_); 7.29 (*ddd*, *J* = 7.9/7.2/2.0 Hz, 1H, 4-H_benzoyl_); 6.94–6.91 (*m*, 2H, 3/5-H_methoxyphenyl_); 6.12 (*s*, 1H, 4-H_pyrazolyl_); 4.42 (*d*, *J* = 6.1 Hz, 2H, C*H*_2_); 3.73 (*s*, 3H, C*H*_3_). ^13^C-NMR (101 MHz, DMSO-*d_6_*): *δ* (ppm) = 170.5 (1C, *C*=O); 158.4 (1C, C-4_methoxyphenyl_); 153.2 (1C, C-5_pyrazolyl_); 152.3 (1C, C-3_pyrazolyl_); 150.0 (1C, C-6_pyridyl_); 147.0 (1C, C-2_pyridyl_); 140.6 (1C, C-1_benzoyl_); 138.4 (1C, C-3_benzoyl_); 133.1 (1C, C-4_pyridyl_); 131.4 (1C, C-4_benzoyl_); 130.4 (1C, C-1_methoxyphenyl_); 128.8 (2C, C-2/6_methoxyphenyl_); 128.7 (1C, C-6_benzoyl_); 127.6 (1C, C-5_benzoyl_); 127.5 (1C, C-3_pyridyl_), 123.7 (1C, C-5_pyridyl_), 113.8 (2C, C-3/5_methoxyphenyl_); 93.6 (1C, C-2_benzoyl_); 83.8 (1C, C-4_pyrazolyl_); 55.0 (1C, *C*H_3_); 47.3 (1C, *C*H_2_). IR (neat): *ṽ* [cm^−1^] = 3408, 1684, 1597, 1576, 1512, 1418, 1362, 1240, 1179, 1016, 937, 920, 814, 737, 721, 704. HRMS (APCI): *m*/*z* = 511.0617, calculated for C_23_H_20_IN_4_O_2_^+^ [M + H]^+^ 511.0626. HPLC: *t*_R_ = 19.7 min, purity: 90.3%.

*(2-iodophenyl)(5-{[(naphthalen-1-yl)methyl]amino}-3-(pyridin-3-yl)-1H-pyrazol-1-yl)methanone* (**9c**). According to general procedure D, aminopyrazole **8c** (500 mg, 1.66 mmol) was acylated using 2-iodobenzoyl chloride (532 mg, 2.00 mmol) in dry pyridine/THF (19 mL/9 mL). Flash column chromatography (CH/EtOAc = 1/0 → 0/1) yielded **9c** as a yellowish solid (358 mg, 40%). M.p.: 137 °C. TLC: *R*_f_ = 0.35 (CH/EtOAc = 1/1). ^1^H-NMR (600 MHz, DMSO-*d_6_*): *δ* (ppm) = 8.80 (*dd*, *J* = 2.2/0.9 Hz, 1H, 2-H_pyridyl_); 8.51 (*dd*, *J* = 4.8/1.7 Hz, 1H, 6-H_pyridyl_); 8.26–8.20 (*m*, 1H, 9-H_naphthyl_); 7.99–7.90 (*m*, 3H, 4-H_pyridyl_, 3-H_benzoyl_, 6-H_naphthyl_); 7.88–7.82 (*m*, 2H, N*H*, 4-H_naphthyl_); 7.66–7.46 (*m*, 6H, 6-H_benzoyl_, 5-H_benzoyl_, 2-H_naphthyl_, 3-H_naphthyl_, 7-H_naphtyl_, 8-H_naphthyl_); 7.36 (*ddd*, *J* = 8.0/4.8/0.9 Hz, 1H, 5-H_pyridyl_); 7.27 (*ddd*, J = 8.0/7.3/1.8 Hz, 1H, 4-H_benzoyl_); 6.20 (*s*, 1H, 4-H_pyrazolyl_); 4.98 (*d*, *J* = 6.0 Hz, 2H, C*H*_2_). ^13^C-NMR (151 MHz, DMSO-*d_6_*): *δ* (ppm) = 170.6 (1C, *C*=O); 153.4 (1C, C-5_pyrazolyl_); 152.4 (1C, C-3_pyrazolyl_); 150.0 (1C, C-6_pyridyl_); 147.1 (1C, C-2_pyridyl_); 140.6 (1C, C-1_benzoyl_); 138.5 (1C, C-3_benzoyl_); 133.4 (1C, C-10_naphthyl_); 133.0 (1C, C-4_pyridyl_); 131.4 (1C, C-4_benzoyl_); 131.0 (1C, C-5_naphthyl_); 128.7 (1C, C-6_benzoyl_); 128.4 (1C, C-6_naphthyl_); 127.6 (1C, C-5_benzoyl_); 127.5 (1C, C-3_pyridyl_); 126.2 (1C, C-7_naphthyl_); 125.8 (1C, C-2_naphthyl_); 125.4 (1C, C-3_naphthyl_); 124.7 (1C, C-8_naphthyl_); 123.7 (1C, C-5_pyridyl_); 123.6 (1C, C-9_naphthyl_); 93.5 (1C, C-2_benzoyl_); 83.8 (1C, C-4_pyrazolyl_); 46.1 (1C, *C*H_2_), the signals of C-1_naphthyl_ and C-4_naphthyl_ cannot be seen on the spectrum. IR (neat): *ṽ* [cm^−1^] = 1692, 1599, 1568, 1489, 1435, 1368, 1342, 1184, 1063, 1005, 930, 853, 748, 704. HRMS (APCI): *m*/*z* = 531.0648, calculated for C_26_H_20_IN_4_O^+^ [M + H]^+^ 531.0676. HPLC: *t*_R_ = 21.2 min, purity: 94.2%.

*{5-[(furan-2-ylmethyl)amino]-3-(pyridin-3-yl)-1H-pyrazol-1-yl}(2-iodophenyl)methanone* (**9d**). According to general procedure D, aminopyrazole **8d** (500 mg, 2.08 mmol) was acylated using 2-iodobenzoyl chloride (665 mg, 2.5 mmol) in dry pyridine/THF (22 mL/13 mL). Flash column chromatography (CH/EtOAc = 1/0 → 0/1) yielded **9d** as a yellowish solid (792 mg, 81%). M.p.: 103–104 °C. TLC: *R*_f_ = 0.68 (CH/EtOAc = 1/1). ^1^H-NMR (600 MHz, DMSO-*d_6_*): *δ* (ppm) = 8.85 (*dd*, *J* = 2.2/0.8 Hz, 1H, 2-H_pyridyl_); 8.56 (*dd*, *J* = 4.8/1.7 Hz, 1H, 6-H_pyridyl_); 7.98–7.96 (*m*, 1H, 4-H_pyridyl_); 7.95 (*dd*, *J* = 8.1/1.1 Hz, 1H, 3-H_benzoyl_); 7.71 (*t*, *J* = 6.2 Hz, 1H, N*H*); 7.64 (*dd*, *J* = 1.9/0.9 Hz, 1H, 5-H_furanyl_); 7.57 (*dd*, *J* = 7.6/1.8 Hz, 1H, 6-H_benzoyl_); 7.53 (*td*, *J* = 7.5/1.1 Hz, 1H, 5-H_benzoyl_); 7.42 (*ddd*, *J* = 8.0/4.8/0.9 Hz, 1H, 5-H_pyridyl_); 7.29 (*ddd*, *J* = 8.0/7.4/1.8 Hz, 1H, 4-H_benzoyl_); 6.52–6.49 (*m*, 1H, 3-H_furanyl_); 6.43 (*dd*, *J* = 3.2/1.8 Hz, 1H, 4-H_furanyl_); 6.24 (*s*, 1H, 4-H_pyrazolyl_); 4.49 (*d*, *J* = 6.0 Hz, 2H, C*H*_2_). ^13^C-NMR (151 MHz, DMSO-*d_6_*): *δ* (ppm) = 170.5 (1C, *C*=O); 152.9 (1C, C-5_pyrazolyl_); 152.3 (1C, C-3_pyrazolyl_); 151.5 (1C, C-1_furanyl_); 150.0 (1C, C-6_pyridyl_); 147.0 (1C, C-2_pyridyl_); 142.5 (1C, C-4_furanyl_); 140.5 (1C, C-1_benzoyl_); 138.4 (1C, C-3_benzoyl_); 133.0 (1C, C-4_pyridyl_); 131.5 (1C, C-4_benzoyl_); 128.7 (1C, C-6_benzoyl_); 127.6 (1C, C-5_benzoyl_); 127.5 (1C, C-3_pyridyl_); 123.8 (1C, C-5_pyridyl_); 120.4 (1C, C-2_furanyl_); 107.9 (1C, C-3_furanyl_); 93.5 (1C, C-2_benzoyl_); 84.0 (1C, C-4*_pyrazol_*_yl_); 41.1 (1C, *C*H_2_). IR (neat): *ṽ* [cm^−1^] = 3381, 1692, 1603, 1576, 1522, 1501, 1373, 1238, 1196, 1153, 939, 901, 810, 735. HRMS (APCI): *m*/*z* = 471.0287, calculated for C_20_H_16_IN_4_O_2_^+^ [M + H]^+^ 471.0313. HPLC: *t*_R_ = 18.7 min, purity: 95.6%.

*(5-{[(5-chlorothiophen-2-yl)methyl]amino}-3-(pyridin-3-yl)-1H-pyrazol-1-yl)(2-iodophenyl)methanone* (**9e**). According to general procedure D, aminopyrazole **8e** (142 mg, 488 µmol) was acylated using 2-iodobenzoyl chloride (156 mg, 586 µmol) in dry pyridine/THF (4.50 mL/2.25 mL). Flash column chromatography (CH/EtOAc = 1/0 → 0/1) yielded **9e** as a yellowish solid (146 mg, 280 µmol, 57%). M.p.: 105–106 °C. TLC: *R*_f_ = 0.18 (CH/EtOAc = 70/30). ^1^H NMR (600 MHz, DMSO-*d*_6_): *δ* (ppm) = 4.60 (d, *J* = 6.5 Hz, 2H, CH_2_), 6.24 (s, 1H, 4-H_pyrazolyl_), 6.99 (d, *J* = 3.7 Hz, 1H, 4-H_thiophenyl_), 7.10–7.12 (m, 1H, 3-H_thiophenyl_), 7.29 (td, *J* = 7.6/1.8 Hz, 1H, 4-H_iodobenzoyl_), 7.41 (ddd, *J* = 7.9/4.8/0.9 Hz, 1H, 5-H_pyridyl_), 7.53 (td, *J* = 7.5/1.1 Hz, 1H, 5-H_iodobenzoyl_), 7.58 (dd, *J* = 7.6/1.7 Hz, 1H, 6-H_iodobenzoyl_), 7.93–8.01 (m, 3H, NH, 4-H_pyridyl_, 3-H_iodobenzoyl_), 8.56 (dd, *J* = 4.8/1.7 Hz, 1H, 6-H_pyridyl_), 8.84 (dd, *J* = 2.2/0.9 Hz, 1H, 2-H_pyridyl_). ^13^C NMR (151 MHz, DMSO-*d*_6_): *δ* (ppm) = 43.3 (1C, CH_2_), 84.2 (1C, C-4_pyrazolyl_), 93.6 (1C, C-2_iodobenzoyl_), 123.8 (1C, C-5_pyridyl_), 126.2 (1C, C-3_thiophenyl_), 126.3 (1C, C-4_thiophenyl_), 127.0 (1C, C-5_thiophenyl_), 127.5 (1C, C-3_pyridyl_), 127.7 (1C, C-5_iodobenzoyl_), 128.8 (1C, C-6_iodobenzoyl_), 131.5 (1C, C-4_iodobenzoyl_), 133.1 (1C, C-4_pyridyl_), 138.4 (1C, C-3_iodobenzoyl_), 140.6 (1C, C-1_iodobenzoyl_), 141.5 (1C, C-2_thiophenyl_), 147.1 (1C, C-2_pyridyl_), 150.1 (1C, C-6_pyridyl_), 152.3 (1C, C-3_pyrazolyl_), 152.6 (1C, C-5_pyrazolyl_), 170.4 (1C, CON). IR (neat): *ṽ* [cm^−1^] = 3379, 1685, 1589, 1516, 1419, 1350, 1230, 1018, 1002, 937, 918, 794, 740. HRMS (APCI): *m*/*z* = 520.9694 calculated for [M + H]^+^, found: 520.9716. HPLC: *t*_R_ = 20.5 min, purity: 95.8%.

*{5-[(cyclohexylmethyl)amino]-3-(pyridin-3-yl)-1H-pyrazol-1-yl}(2-iodophenyl)methanone* (**9f**). According to general procedure D, aminopyrazole **8f** (130 mg, 507 µmol) was acylated using 2-iodobenzoyl chloride (162 mg, 609 µmol) in dry pyridine/THF (6 mL/3 mL). Flash column chromatography (CH/EtOAc = 1/0 → 0/1) yielded **9e** as a beige solid (92 mg, 37%). M.p.: 123–124 °C. TLC: *R*_f_ = 0.48 (CH/EtOAc = 1/1). ^1^H-NMR (600 MHz, DMSO-*d_6_*): *δ* (ppm) = 8.87 (*dd*, *J* = 2.2/0.9 Hz, 1H, 2-H_pyridyl_); 8.56 (*dd*, *J* = 4.8/1.7 Hz, 1H, 6-H_pyridyl_); 7.99 (*dt*, *J* = 8.0/2.0 Hz, 1H, 3-H_benzoyl_); 7.94 (*dd*, *J* = 8.0/1.0 Hz, 1H, 4-H_pyridyl_); 7.57 (*dd*, *J* = 7.7/2.0 Hz, 1H, 6-H_benzoyl_); 7.53 (*td*, *J* = 7.4/1.0 Hz, 1H, 5-H_benzoyl_); 7.41 (*ddd*, J = 8.0/4.8/0.9 Hz, 1H, 5-H_pyridyl_); 7.32–7.29 (*m*, 1H, 4-H_benzoyl_); 7.29–7.26 (*m*, 1H, N*H*); 6.17 (*s*, 1H, 4-H_pyrazolyl_); 3.14 (*t*, *J* = 6.5 Hz, 2H, C*H*_2_); 1.86–1.60 (*m*, 6H, H_cyclohexyl_); 1.32–1.13 (*m*, 3H, H_cyclohexyl_); 1.09–0.95 (*m*, 2H, H_cyclohexyl_). ^13^C-NMR (151 MHz, DMSO-*d_6_*): *δ* (ppm) = 170.7 (1C, *C*=O); 153.8 (1C, C-5_pyrazolyl_); 152.5 (1C, C-3_pyrazolyl_); 150.0 (1C, C-6_pyridyl_); 147.1 (1C, C-2_pyridyl_); 140.7 (1C, C-1_benzoyl_); 138.2 (1C, C-4_pyridyl_); 133.1 (1C, C-3_benzoyl_); 131.4 (1C, C-4_benzoyl_); 128.7 (1C, C-6_benzoyl_); 127.7 (1C, C-5_benzoyl_); 123.7 (1C, C-5_pyridyl_); 93.5 (1C, C-2_benzoyl_); 83.2 (1C, C-4_pyrazolyl_); 50.8 (1C, *C*H_2_); 36.5 (1C, C_cyclohexyl_); 30.2 (2C, C_cyclohexyl_); 26.0 (1C, C_cyclohexyl_); 25.3 (2C, C_cyclohexyl_). IR (neat): *ṽ* [cm^−1^] = 3217, 3152, 2920, 2837, 2361, 1738, 1686, 1601, 1576, 1524, 1489, 1366, 1229, 1209, 1182, 1016, 853, 746, 725. HRMS (APCI): *m*/*z* = 487.0986, calculated for C_22_H_24_IN_4_O^+^ [M + H]^+^ 487.0989. HPLC: *t*_R_ = 22.0 min, purity: 99.3%.

*(5-{[(5-chlorothiophen-2-yl)methyl]amino}-3-phenyl-1H-pyrazol-1-yl)(2-iodophenyl)methanone* (**9g**). According to general procedure D, aminopyrazole **8g** (160 mg, 552 µmol) was acylated using 2-iodobenzoyl chloride (177 mg, 663 µmol) in dry pyridine/THF (6 mL/3 mL). Flash column chromatography (CH/EtOAc = 1/0 → 0/1) yielded **9g** as a colorless solid (121 mg, 42%). M.p.: 137–138 °C. TLC: *R*_f_ = 0.47 (CH/EtOAc = 80/20). ^1^H-NMR (600 MHz, DMSO-*d_6_*): *δ* (ppm) = 7.94 (*d*, *J* = 7.9 Hz, 1H, 3-H_benzoyl_); 7.88 (*t*, *J* = 6.3 Hz, 1H, N*H*); 7.65–7.59 (*m*, 2H, H_phenyl_); 7.85–7.49 (*m*, 2H, 5/6-H_benzoyl_); 7.41–7.33 (*m*, 3H, H_phenyl_); 7.28 (*td*, *J* = 7.4/2.0 Hz, 1H, 4-H_benzoyl_); 7.13–7.06 (*m*, 1H, 3-H_chlorothiophenyl_); 6.99 (*d*, *J* = 3.8/0.8 Hz, 1H, 4-H_chlorothiophenyl_); 6.11 (*s*, 1H, 4-H_pyrazolyl_); 4.60 (*d*, *J* = 6.3 Hz, 2H, C*H*_2_). ^13^C-NMR (151 MHz, DMSO-*d_6_*): *δ* (ppm) = 170.4 (1C, *C*=O); 154.6 (1C, C-5_pyrazolyl_); 152.4 (1C, C-3_pyrazolyl_); 141.6 (1C, C-2_chlorothiophenyl_); 140.8 (1C, C-1_benzoyl_); 138.4 (1C, C-3_benzoyl_); 131.7 (1C, C-_phenyl_); 131.4 (1C, C-4_benzoyl_); 129.2 (1C, C_phenyl_); 128.7 (1C, C-6_benzoyl_); 128.6 (2C, C_phenyl_); 127.6 (1C, C-5_benzoyl_); 126.9 (1C, C-5_chlorothiophenyl_); 126.2 (1C, C-4_chlorothiophenyl_); 126.0 (1C, C-3_chlorothiophenyl_); 125.9 (2C, C-_phenyl_); 93.5 (1C, C-2_benzoyl_); 84.3 (1C, C-4_pyrazolyl_); 43.3 (1C, *C*H_2_). IR (neat): *ṽ* [cm^−1^] = 3389, 1682, 1597, 1578, 1520, 1503, 1449, 1395, 1234, 1165, 1059, 1001, 935, 918, 800, 733. HRMS (APCI): *m*/*z* = 519.9739, calculated for C_21_H_16_ClIN_3_OS^+^ [M + H]^+^ 519.9742. HPLC: *t*_R_ = 24.9 min, purity: 96.7%.

*[5-(benzylamino)-3-cyclohexyl-1H-pyrazol-1-yl](2-iodophenyl)methanone* (**9h**). According to general procedure D, aminopyrazole **8h** (100 mg, 392 µmol) was acylated using 2-iodobenzoyl chloride (125 mg, 470 µmol) in dry pyridine/THF (4 mL/2 mL). Flash column chromatography (CH/EtOAc = 1/0 → 0/1) yielded **9g** as a colorless oil (103 mg, 54%). TLC: *R*_f_ = 0.60 (CH/EtOAc = 80/20). ^1^H-NMR (600 MHz, DMSO-*d_6_*): *δ* (ppm) = 7.91–7.86 (*m*, 1H, 3-H_benzoyl_); 7.59 (*t*, J = 6.2 Hz, 1H, N*H*); 7.51–7.45 (*m*, 2H, 5/6-H_benzoyl_); 7.44–7.39 (*m*, 2H, 2/6-H_phenyl_); 7.39–7.33 (*m*, 2H, 3/5-H_phenyl_); 7.29–7.25 (*m*, 1H, 4-H_phenyl_); 7.23 (*ddd*, *J* = 7.9/7.0/2.1 Hz, 1H, 4-H_benzoyl_); 5.33 (*s*, 1H, 4-H_pyrazolyl_); 4.39 (*d*, *J* = 6.0 Hz, 2H, C*H*_2_); 2.32–2.22 (*m*, 1H, H_cyclohexyl_), 1.73–1.62 (*m*, 4H, H_cyclohexyl_), 1.58 (*d*, *J* = 12.7 Hz, 1H, H_cyclohexyl_), 1.29–1.17 (*m*, 4H, H_cyclohexyl_), 1.18–1.07 (*m*, 0H, H_cyclohexyl_). ^13^C-NMR (151 MHz, DMSO-*d_6_*): *δ* (ppm) = 170.9 (1C, *C*=O); 163.3 (1C, C-5_pyrazolyl_); 152.9 (1C, C-3_pyrazolyl_); 141.7 (1C, C-1_benzoyl_); 139.3 (1C, C-1_phenyl_); 138.8 (1C, C-3_benzoyl_); 131.6 (1C, C-4_benzol_); 128.9 (3C, C-3/5_phenyl_, C-6_benzoyl_); 128.0 (1C, C-5_benzoyl_); 127.8 (2C, C-2/6_phenyl_); 127.5 (1C, C-4_phenyl_); 94.0 (1C, C-2_benzoyl_); 84.5 (1C, C-4_pyrazolyl_); 48.3 (1C, *C*H_2_); 37.6 (1C, C_cyclohexyl_); 32.1 (2C, C_cyclohexyl_); 26.8 (1C, C_cyclohexyl_); 25.8 (2C, C_cyclohexyl_). IR (neat): *ṽ* [cm^−1^] = 3387, 2924, 2849, 1682, 1593, 1524, 1466, 1431, 1373, 1290, 1229, 1202, 1165, 1119, 1016, 974, 926, 891, 856, 824, 735. HRMS (APCI): *m*/*z* = 486.1004, calculated for C_23_H_25_IN_3_O^+^ [M + H]^+^ 486.1037. HPLC: *t*_R_ = 25.5 min, purity: 97.0%.

*(5-{[(5-chlorothiophen-2-yl)methyl]amino}-3-cyclohexyl-1H-pyrazol-1-yl)(2-iodophenyl)methanone* (**9i**). According to general procedure D, aminopyrazole **8i** (72 mg, 243 µmol) was acylated using 2-iodobenzoyl chloride (78 mg, 292 µmol) in dry pyridine/THF (3 mL/1.5 mL). Flash column chromatography (CH/EtOAc = 1/0 → 0/1) yielded **9i** as a colorless oil (52 mg, 41%). TLC: *R*_f_ = 0.56 (CH/EtOAc = 80/20). ^1^H-NMR (600 MHz, DMSO-*d_6_*): *δ* (ppm) = 7.88 (*d*, *J* = 7.8/1.0 Hz, 1H, 3-H_benzoyl_); 7.66 (*t*, *J* = 6.3 Hz, 1H, N*H*); 7.51–7.43 (*m*, 2H, 5/6-H_benzoyl_); 7.23 (*ddd*, J = 8.0/7.2/2.0 Hz, 1H, 4-H_benzoyl_); 7.03–6.99 (*m*, 1H, 3-H_chlorothiophenyl_); 6.98 (*d*, *J* = 3.7 Hz, 1H, 4-H_chlorothiophenyl_); 5.47 (*s*, 1H, 4-H_pyrazolyl_); 4.50 (*d*, *J* = 6.1 Hz, 2H, C*H*_2_); 2.33–2.23 (*m*, 1H, H_cyclohexyl_), 1.78–1.63 (*m*, 4H, H_cyclohexyl_), 1.62–1.54 (*m*, 1H, H_cyclohexyl_), 1.32–1.20 (*m*, 4H, H_cyclohexyl_), 1.20–1.09 (*m*, 1H, H_cyclohexyl_). ^13^C-NMR (151 MHz, DMSO-*d_6_*): *δ* (ppm) = 170.7 (1C, *C*=O); 163.2 (1C, C-5_pyrazolyl_); 152.2 (1C, C-3_pyrazolyl_); 142.3 (1C, C-2_chlorothiophenyl_); 141.6 (1C, C-1_benzoyl_); 138.8 (1C, C-3_benzoyl_); 131.6 (1C, C-4_benzoyl_); 129.0 (1C, C-6_benzoyl_); 128.1 (1C, C-5_benzoyl_); 127.3 (1C, C-5_chlorothiophenyl_); 126.8 (1C, C-4_chlorothiophenyl_); 126.3 (1C, C-3_chlorothiophenyl_); 94.0 (1C, C-2_benzoyl_); 85.0 (1C, C-4_pyrazolyl_); 43.8 (1C, *C*H_2_); 37.6 (1C, C_cyclohexyl_); 32.1 (2C, C_cyclohexyl_); 26.0 (1C, C_cyclohexyl_); 25.8 (2C, C_cyclohexyl_). IR (neat): *ṽ* [cm^−1^] = 3379, 2924, 2849, 1682, 1591, 1524, 1449, 1371, 1348, 1117, 1061, 974, 920, 856, 793, 741. HRMS (APCI): *m*/*z* = 526.0207, calculated for C_21_H_22_ClIN_3_OS^+^ [M + H]^+^ 526.0211. HPLC: *t*_R_ = 26.1 min, purity: 96.0%.

*4-benzyl-2-(pyridin-3-yl)pyrazolo[5,1b]quinazolin-9(4H)-one* (**10a**). According to general procedure E, **9a** (63.2 mg, 131 µmol), CuI (5.0 mg, 26.3 µmol, 0.20 eq.), Cs_2_CO_3_ (85.7 mg, 263 µmol, 2.00 eq.) and 1,10-phenanthroline (4.7 mg, 26.3 µmol, 0.20 eq.) were dissolved in dry DMF (2 mL) and stirred at 80 °C for 3 h. Flash column chromatography (CH_2_Cl_2_/CH_3_OH = 96/4) yielded **10a** as a colorless solid (37.9 mg, 82%). M.p.: 266 °C. TLC: *R*_f_ = 0.44 (CH_2_Cl_2_/CH_3_OH = 96/4). ^1^H-NMR (400 MHz, DMSO-*d_6_*): *δ* (ppm) = 9.22 (*bs*, 1H, 2-H_pyridyl_); 8.68 (*bs*, 1H, 6-H_pyridyl_); 8.40–8.37 (*m*, 1H, 4-H_pyridyl_); 8.37–8.32 (*m*, 1H, 8-H_quinazolinyl_); 7.80 (*ddd*, *J* = 8.7/7.1/1.7 Hz, 1H, 6-H_quinazolinyl_); 7.59–7.54 (*m*, 1H, 5-H_pyridyl_); 7.52 (*d*, *J* = 8.7 Hz, 1H, 5-H_quinazolinyl_); 7.39–7.35 (*m*, 1H, 7-H_quinazolinyl_); 7.34 (*dd*, *J* = 0.9 Hz, 2H, 3/5-H_phenyl_); 7.34–7.33 (*m*, 2H, 2/6-H_phenyl_); 7.28 (*dd*, *J* = 8.6/3.3 Hz, 1H, 4-H_phenyl_); 7.14 (*s*, 1H, 3-H_quinazolinyl_); 5.64 (*s*, 2H, C*H*_2_). ^13^C-NMR (101 MHz, DMSO-*d_6_*): *δ* (ppm) = 155.1 (1C, C-9_quinazolinyl_, *C*=O); 152.4 (1C, C-2_quinazolinyl_); 150.2 (1C, C-6_pyridyl_); 147.3 (1C, C-2_pyridyl_); 146.1 (1C, C-3a_quinazolinyl_); 139.7 (1C, C-4a_quinazolinyl_); 135.3 (1C, C-6_quinazolinyl_); 135.1 (1C, C-1_phenyl_); 133.4 (1C, C-4_pyridyl_); 128.9 (2C, C-3/5_phenyl_); 128.4 (1C, C-8_quinazolinyl_); 126.4 (2C, C-2/6_phenyl_); 124.2 (1C, C-5_pyridyl_); 122.0 (1C, C-7_quinazolinyl_); 114.3 (1C, C-5_quinazolinyl_); 113.4 (1C, C-8a_quinazolinyl_); 86.3 (1C, C-3_quinazolinyl_); 50.7 (1C, *C*H_2_), the signal of C-3_pyridyl_ cannot be seen on the spectrum. IR (neat): *ṽ* [cm^−1^] = 3096, 1695, 1601, 1566, 1491, 1435, 1369, 1180, 1026, 951, 934, 854, 797, 745, 706. HRMS (APCI): *m*/*z* = 353.1408, calculated for C_22_H_17_N_4_O^+^ [M + H]^+^ 353.1397. HPLC: *t*_R_ = 16.6 min, purity: 94.3%.

*4-(4-methoxybenzyl)-2-(pyridin-3-yl)pyrazolo[5,1-b]quinazolin-9(4H)-one* (**10b**). According to general procedure E, **9b** (150 mg, 294 µmol), CuI (11.2 mg, 58.8 µmol, 0.20 eq.), Cs_2_CO_3_ (192 mg, 588 µmol, 2.00 eq.) and 1,10-phenanthroline (10.6 mg, 58.8 µmol, 0.20 eq.) were dissolved in dry DMF (6 mL) and stirred at 80 °C for 90 min. Flash column chromatography (CH_2_Cl_2_/CH_3_OH = 97/3) yielded **10b** as a colorless solid (39 mg, 35%). M.p.: 245 °C. TLC: *R*_f_ = 0.43 (CH_2_Cl_2_/CH_3_OH = 95/5). ^1^H-NMR (400 MHz, DMSO-*d_6_*): *δ* (ppm) = 9.24 (*bs*, 1H, 2-H_pyridyl_); 8.69 (*bs*, 1H, 6-H_pyridyl_); 8.38 (*d*, *J* = 8.0 Hz, 1H, 4-H_pyridyl_); 8.35 (*dd*, *J* = 8.0/1.6 Hz, 1H, 8-H_quinazolinyl_); 7.81 (*ddd*, *J* = 8.7/7.1/1.7 Hz, 1H, 6-H_quinazolinyl_); 7.63–7.51 (*m*, 2H, 5-H_pyridyl_, 5-H_quinazolinyl_); 7.36 (*ddd*, *J* = 8.0/7.1/0.8 Hz, 1H, 7-H_quinazolinyl_); 7.31–7.25 (*m*, 2H, 2/6-H_methoxyphenyl_); 7.16 (*s*, 1H, 3-H_quinazolinyl_); 6.92–6.85 (*m*, 2H, 3/5-H_methoxyphenyl_); 5.56 (*s*, 2H, C*H*_2_); 3.69 (*s*, 3H, C*H*_3_). ^13^C-NMR (101 MHz, DMSO-*d_6_*): *δ* (ppm) = 158.7 (1C, C-4_methoxyphenyl_); 155.1 (1C, C-9_quinazolinyl_, *C*=O); 152.4 (1C, C-2_quinazolinyl_); 150.2 (1C, C-6_pyridyl_); 147.3 (1C, C-2_pyridyl_); 146.0 (1C, C-3a_quinazolinyl_); 139.6 (1C, C-4a_quinazolinyl_); 135.2 (1C, C-6_quinazolinyl_); 133.4 (1C, C-4_pyridyl_); 128.3 (1C, C-8_quinazolinyl_); 127.9 (2C, C-2/6_methoxyphenyl)_; 127.0 (1C, C-1_methoxyphenyl_); 121.8 (1C, C-7_quinazolinyl_); 114.4 (1C, C-5_quinazolinyl_); 114.2 (2C, C-3/5_methoxyphenyl_); 113.3 (1C, C-8a_quinazolinyl_); 86.2 (1C, C-3_quinazolinyl_); 55.0 (3C, *C*H_3_); 50.1 (1C, *C*H_2_), the signals of C-3_pyridyl_ and C-5_pyridyl_ cannot be seen on the spectrum. IR (neat): *ṽ* [cm^−1^] = 3098, 3013, 2361, 1714, 1695, 1512, 1491, 1433, 1371, 1346, 1290, 1250, 1182, 1022, 928, 856, 743, 706. HRMS (APCI): *m*/*z* = 383.1502, calculated for C_23_H_19_N_4_O_2_^+^ [M + H]^+^ 383.1503. HPLC: *t*_R_ = 16.8 min, purity: 99.5%.

*4-[(naphthalen-1-yl)methyl]-2-(pyridin-3-yl)pyrazolo[5,1-b]quinazolin-9(4H)-one* (**10c**). According to general procedure E, **9c** (150 mg, 283 µmol), CuI (10.8 mg, 56.6 µmol, 0.20 eq.), Cs_2_CO_3_ (184 mg, 566 µmol, 2.00 eq.) and 1,10-phenanthroline (10.2 mg, 56.6 µmol, 0.20 eq.) were dissolved in dry DMF (6 mL) and stirred at 80 °C for 90 min. Flash column chromatography (CH_2_Cl_2_/CH_3_OH = 97/3) yielded **10c** as a beige solid (63 mg, 55%). M.p.: 252 °C. TLC: *R*_f_ = 0.41 (CH_2_Cl_2_/CH_3_OH = 95/5). ^1^H-NMR (600 MHz, DMSO-*d_6_*): *δ* (ppm) = 9.15 (*d*, *J* = 2.1 Hz, 1H, 2-H_pyridyl_); 8.60 (*dd*, *J* = 4.9/1.6 Hz, 1H, 6-H_pyridyl_); 8.41 (*dd*, *J* = 8.0/1.6 Hz, 1H, 8-H_quinazolinyl_); 8.37–8.31 (*m*, 2H, 9-H_naphthyl_, 4-H_pyridyl_); 8.07–8.01 (*m*, 1H, H_naphthyl_); 7.86 (*d*, *J* = 8.3 Hz, 1H, H_naphthyl_); 7.78–7.70 (*m*, 2H, 5/6-H_quinazolinyl_); 7.67 (*ddd*, *J* = 8.1/6.9/1.1 Hz, 1H, H_naphthyl_); 7.53–7.47 (*m*, 1H, 5-H_pyridyl_); 7.37 (*ddd*, *J* = 8.0/7.1/0.8 Hz, 1H, 7-H_quinazolinyl_); 7.33–7.25 (*m*, 2H, H_naphthyl_); 7.06 (*s*, 1H, 3-H_quinazolinyl_); 6.92 (*m*, 1H, H_naphthyl_); 6.12 (*s*, 2H, C*H*_2_). ^13^C-NMR (151 MHz, DMSO-*d_6_*): *δ* (ppm) = 155.2 (1C, C-9_quinazolinyl_, *C*=O); 152.4 (1C, C-2_quinazolinyl_); 150.2 (1C, C-6_pyridyl_); 147.4 (1C, C-2_pyridyl_); 146.0 (1C, C-3a_quinazolinyl_); 139.9 (1C, C-4a_quinazolinyl_); 135.2 (1C, C-6_quinazolinyl_); 133.4 (1C, C-4_pyridyl_); 130.2 (1C, C_naphthyl_); 130.2 (1C, C_naphthyl_); 129.5 (1C, C_naphthyl_); 128.6 (1C, C_naphthyl_); 128.4 (1C, C-8_quinazolinyl_); 127.7 (1C, C-3_pyridyl_); 126.4 (1C, C-5_quinazolinyl_); 126.2 (1C, C_naphthyl_); 125.5 (1C, C_naphthyl_); 123.9 (1C, C-5_pyridyl_); 123.3 (1C, C-9_naphthyl_); 122.1 (1C, C-7_quinazolinyl_); 121.7 (1C, C_naphthyl_); 114.2 (1C, C_naphthyl_); 113.5 (1C, C-8a_quinazolinyl_); 86.2 (1C, C-3_quinazolinyl_); 49.2 (1C, *C*H_2_), the signals of C-1_naphthyl_ and C-4_naphthyl_ cannot be seen on the spectrum. IR (neat): *ṽ* [cm^−1^] = 1707, 1601, 1566, 1493, 1435, 1412, 1296, 1184, 1024, 932, 787, 766, 706. HRMS (APCI): *m*/*z* = 403.1511, calculated for C_26_H_19_N_4_O^+^ [M + H]^+^ 403.1553. HPLC: *t*_R_ = 18.7 min, purity: 99.2%.

*4-[(furan-2-yl)methyl]-2-(pyridin-3-yl)pyrazolo[5,1-b]quinazolin-9(4H)-one* (**10d**). According to general procedure E, **9d** (100 mg, 213 µmol), CuI (8.1 mg, 42.5 µmol, 0.20 eq.), Cs_2_CO_3_ (139 mg, 425 µmol, 2.00 eq.) and 1,10-phenanthroline (7.7 mg, 42.5 µmol, 0.20 eq.) were dissolved in dry DMF (4 mL) and stirred at 80 °C for 45 min. Flash column chromatography (CH_2_Cl_2_/CH_3_OH = 97/3) yielded **10d** as a beige solid (31 mg, 43%). M.p.: 272 °C. TLC: *R*_f_ = 0.52 (CH_2_Cl_2_/CH_3_OH = 95/5). ^1^H-NMR (600 MHz, DMSO-*d_6_*): *δ* (ppm) = 9.26 (*bs*, 1H, 2-H_pyridyl_); 8.70 (*bs*, 1H, 6-H_pyridyl_); 8.40 (*dt*, *J* = 7.9/1.6 Hz, 1H, 8-H_quinazolinyl_); 8.33 (*dt*, *J* = 8.1/1.1 Hz, 1H, 4-H_pyridyl_); 7.94–7.87 (*m*, 2H, 5/6-H_quinazolinyl_); 7.62 (*dd*, *J* = 1.8/0.8 Hz, 1H, 5-H_furanyl_); 7.58 (*dd*, *J* = 8.0/4.4 Hz, 1H, 7-H_quinazolinyl_); 7.39 (*ddd*, *J* = 8.0/4.7/3.3 Hz, 1H, 5-H_pyridyl_); 7.24 (*s*, 1H, 3-H_quinazolinyl_); 6.75 (*dd*, *J* = 3.3/0.8 Hz, 1H, 3-H_furanyl_); 6.43 (*dd*, *J* = 3.3/1.8 Hz, 1H, 4-H_furanyl_); 5.62 (*s*, 2H, C*H*_2_). ^13^C-NMR (151 MHz, DMSO-*d_6_*): *δ* (ppm) = 155.0 (1C, C-9_quinazolinyl_, *C*=O); 152.3 (1C, C-2_quinazolinyl_); 150.2 (1C, C-6_pyridyl_); 148.4 (1C, C-2_furanyl_); 147.4 (1C, C-2_pyridyl_); 145.3 (1C, C-3a_quinazolinyl_); 143.5 (1C, C-5_furanyl_); 139.5 (1C, C-4a_quinazolinyl_); 135.1 (1C, C-6_quinazolinyl_); 133.5 (1C, C-4_pyridyl_); 128.3 (1C, C-8_quinazolinyl_); 122.1 (1C, C-5_pyridyl_); 114.3 (1C, C-5_quinazolinyl_); 113.2 (1C, C-8a_quinazolinyl_); 110.6 (1C, C-4_furanyl_); 109.9 (1C, C-3_furanyl_); 86.6 (1C, C-3_quinazolinyl_); 44.0 (1C, *C*H_2_), the signals of C-3_pyridyl_ and C-7_quinazolinyl_ cannot be seen on the spectrum. IR (neat): *ṽ* [cm^−1^] = 2970, 2363, 1738, 1692, 1599, 1568, 1489, 1435, 1368, 1344, 1230, 1180, 949, 851, 748. HRMS (APCI): *m*/*z* = 343.1152, calculated for C_20_H_15_N_4_O_2_^+^ [M + H]^+^ 343.1190. HPLC: *t*_R_ = 15.5 min, purity: 99.5%.

*4-[(5-chlorothiophen-2-yl)methyl]-2-(pyridin-3-yl)pyrazolo[5,1-b]quinazolin-9(4H)-one* (**10e**). According to general procedure E, **9e** (50.0 mg, 96.0 µmol), CuI (3.7 mg, 19.2 µmol, 0.20 eq.), Cs_2_CO_3_ (62.6 mg, 192 µmol, 2.00 eq.) and 1,10-phenanthroline (3.5 mg, 19.2 µmol, 0.20 eq.) were dissolved in dry DMF (2 mL) and stirred at 80 °C for 30 min. Flash column chromatography (CH_2_Cl_2_/CH_3_OH = 96/4) yielded **10e** as a colorless solid (34 mg, 90%). M.p.: 248–249 °C. TLC: *R*_f_ = 0.27 (CH_2_Cl_2_/CH_3_OH = 95/5). ^1^H NMR (600 MHz, DMSO-*d*_6_): *δ* (ppm) = 5.75 (s, 2H, CH_2_), 7.00 (d, *J* = 3.8 Hz, 1H, 4-H_thiophenyl_), 7.24 (d, *J* = 3.9 Hz, 1H, 3-H_thiophenyl_), 7.26 (s, 1H, 3-H_quinazolinyl_), 7.40 (ddd, *J* = 8.0/6.9/0.9 Hz, 1H, 7-H_quinazolinyl_), 7.57 (ddd, *J* = 7.9/4.7/0.9 Hz, 1H, 5-H_pyridyl_), 7.82 (d, *J* = 8.5 Hz, 1H, 5-H_quinazolinyl_), 7.90 (ddd, *J* = 8.6/7.0/1.7 Hz, 1H, 6-H_quinazolinyl_), 8.35 (dd, *J* = 8.0/1.6 Hz, 1H, 8-H_quinazolinyl_), 8.38 (dt, *J* = 7.9/1.9 Hz, 1H, 4-H_pyridyl_), 8.67 (dd, *J* = 4.9/1.6 Hz, 1H, 6-H_pyridyl_), 9.21 (d, *J* = 2.4 Hz, 1H, 2-H_pyridyl_). ^13^C NMR (151 MHz, DMSO-*d*_6_): *δ* (ppm) = 46.1 (1C, CH_2_), 86.5 (1C, C-3_quinazolinyl_), 113.5 (1C, C-8a_quinazolinyl_), 114.2 (1C, C-5_quinazolinyl_), 122.4 (1C, C-7_quinazolinyl_), 124.1 (1C, C-5_pyridyl_), 126.5 (1C, C-4_thiophenyl_), 127.7 (1C, C-3_thiophenyl_), 127.8 (1C, C-3_pyridyl_), 127.9 (1C, C-5_thiophenyl_), 128.6 (1C, C-8_quinazolinyl_), 133.5 (1C, C-4_pyridyl_), 135.3 (1C, C-6_quinazolinyl_), 136.4 (1C, C-2_thiophenyl_), 139.1 (1C, C-4a_quinazolinyl_), 145.2 (1C, C-3a_quinazolinyl_), 147.4 (1C, C-2_pyridyl_), 150.4 (1C, C-6_pyridyl_), 152.5 (1C, C-2_quinazolinyl_), 155.0 (1C, CON). IR (neat): *ṽ* [cm^−1^] = 1689, 1600, 1570, 1489, 1477, 1454, 1435, 1411, 1342, 1292, 1184, 1002, 817, 744, 702. HRMS (APCI): *m*/*z* = 393.0571 calculated for [M + H]^+^, found: 393.0550. HPLC: *t*_R_ = 17.8 min, purity: 97.9%.

*4-(cyclohexylmethyl)-2-(pyridin-3-yl)pyrazolo[5,1-b]quinazolin-9(4H)-one* (**10f**). According to general procedure E, **9f** (65.6 mg, 135 µmol), CuI (5.1 mg, 27.0 µmol, 0.20 eq.), Cs_2_CO_3_ (87.9 mg, 270 µmol, 2.00 eq.) and 1,10-phenanthroline (4.9 mg, 27.0 µmol, 0.20 eq.) were dissolved in dry DMF (3 mL) and stirred at 80 °C for 2 h. Flash column chromatography (CH_2_Cl_2_/CH_3_OH = 97/3) yielded **10f** as a yellowish solid (13.5 mg, 28%). M.p.: 235–236 °C. TLC: *R*_f_ = 0.38 (CH_2_Cl_2_/CH_3_OH = 95/5). ^1^H-NMR (600 MHz, DMSO-*d_6_*): *δ* (ppm) = 9.32 (*bs*, 1H, 2-H_pyridyl_); 8.73 (*bs*, 1H, 6-H_pyridyl_); 8.42 (*d*, *J* = 7.9 Hz, 1H, 4-H_pyridyl_); 8.33 (*dd*, *J* = 8.0/1.6 Hz, 1H, 8-H_quinazolinyl_); 7.88 (*ddd*, *J* = 8.7/7.0/1.7 Hz, 2H, 6-H_quinazolinyl_); 7.73 (*d*, *J* = 8.6 Hz, 1H, 5-H_quinazolinyl_); 7.63–7.53 (*m*, 1H, 5-H_pyridyl_); 7.37 (*ddd*, *J* = 7.9/7.0/0.8 Hz, 1H, 7-H_quinazolinyl_); 7.13 (*s*, 1H, 3-H_quinazolinyl_); 4.21 (*d*, *J* = 7.4 Hz, 2H, C*H*_2_); 2.08–1.95 (*m*, 1H, H_cyclohexyl_); 1.74–1.56 (*m*, 4H, H_cyclohexyl_); 1.27–1.08 (*m*, 6H, H_cyclohexyl_). IR (neat): *ṽ* [cm^−1^] = 3109, 2924, 2853, 2361, 1738, 1692, 1564, 1489, 1433, 1184, 1024, 874, 818, 746, 706. HRMS (APCI): *m*/*z* = 359.1963, calculated for C_22_H_23_N_4_O^+^ [M + H]^+^ 359.1866. HPLC: *t*_R_ = 18.6 min, purity: 98.6%.

*4-[(5-chlorothiophen-2-yl)methyl]-2-phenylpyrazolo[5,1-b]quinazolin-9(4H)-one* (**10g**). According to general procedure E, **9g** (100 mg, 192 µmol), CuI (7.3 mg, 38.5 µmol, 0.20 eq.), Cs_2_CO_3_ (125 mg, 385 µmol, 2.00 eq.) and 1,10-phenanthroline (6.9 mg, 38.5 µmol, 0.20 eq.) were dissolved in dry DMF (4 mL) and stirred at 80 °C for 2 h. Flash column chromatography (CH_2_Cl_2_/CH_3_OH = 97/3) yielded **10f** as a beige solid (63.3 mg, 84%). M.p.: 251–252 °C. TLC: *R*_f_ = 0.48 (CH_2_Cl_2_/CH_3_OH = 98/2). ^1^H-NMR (500 MHz, DMSO-*d_6_*): *δ* (ppm) = 8.34 (*dd*, *J* = 8.0/1.6 Hz, 1H, 8-H_quinazolinyl_); 8.06–8.00 (*m*, 2H, H_phenyl_); 7.88 (*ddd*, *J* = 8.6/7.0/1.6 Hz, 1H, 6-H_quinazolinyl_); 7.80 (*d*, *J* = 8.6 Hz, 1H, 5-H_quinazolinyl_); 7.57–7.51 (*m*, 2H, H_phenyl_); 7.49–7.43 (*m*, 1H, H_phenyl_); 7.39 (*ddd*, *J* = 7.9/7.0/0.9 Hz, 1H, 7-H_quinazolinyl_); 7.23 (*d*, *J* = 3.9 Hz, 1H, 3-H_chlorothiophenyl_); 7.15 (*s*, 1H, 3-H_quinazolinyl_); 7.00 (*d*, *J* = 3.8 Hz, 1H, 4-H_chlorothiophenyl_); 5.75 (*s*, 2H, C*H*_2_). ^13^C-NMR (126 MHz, DMSO-*d_6_*): *δ* (ppm) = 154.9 (1C, C-9_quinazolinyl_, *C*=O); 144.9 C-3a_quinazolinyl_); 139.1 (1C, C-4a_quinazolinyl_); 136.5 (1C, C-2_chlorothiophenyl_); 135.1 (1C, C-6_quinazolinyl_); 131.8 (1C, C_phenyl_); 129.4 (1C, C_phenyl_); 128.8 (2C, C_phenyl_); 128.5 (1C, C-8_quinazolinyl_); 127.8 (1C, C-5_chlorothiophenyl_); 127.8 (1C, C-3_pyridyl_); 127.6 (1C, C-3_chlorothiophenyl_); 126.4 (1C, C-4_chlorothiophenyl_); 126.3 (2C, C_phenyl_); 122.1 (1C, C-7_quinazolinyl_); 114.0 (1C, C-5_quinazolinyl_); 113.4 (1C, C-8a_quinazolinyl_); 86.3 (1C, C-3_quinazolinyl_); 45.9 (1C, *C*H_2_), the signal of C-2_quinazolinyl_ cannot be seen on the spectrum. IR (neat): *ṽ* [cm^−1^] = 3389, 1688, 1599, 1491, 1454, 1425, 1371, 1346, 1294, 1186, 1059, 1003, 974, 932, 854, 814, 739. HRMS (APCI): *m*/*z* = 392.0617, calculated for C_21_H_15_ClN_3_OS^+^ [M + H]^+^ 392.0619. HPLC: *t*_R_ = 22.2 min, purity: 97.4%.

*4-benzyl-2-cyclohexylpyrazolo[5,1-b]quinazolin-9(4H)-one* (**10h**). According to general procedure E, **9h** (80 mg, 165 µmol), CuI (6.3 mg, 33.0 µmol, 0.20 eq.), Cs_2_CO_3_ (107 mg, 330 µmol, 2.00 eq.) and 1,10-phenanthroline (5.9 mg, 33.0 µmol, 0.20 eq.) were dissolved in dry DMF (3 mL) and stirred at 80 °C for 6.5 h. Flash column chromatography (CH_2_Cl_2_/CH_3_OH = 97/3) yielded **10h** as a beige solid (34.4 mg, 58%). M.p.: 180–181 °C. TLC: *R*_f_ = 0.57 (CH_2_Cl_2_/CH_3_OH = 95/5). ^1^H-NMR (600 MHz, DMSO-*d_6_*): *δ* (ppm) = 8.30 (*dd*, *J* = 8.0/1.6 Hz, 1H, 8-H_quinazolinyl_); 7.75 (*ddd*, *J* = 8.7/7.1/1.7 Hz, 1H, 6-H_quinazolinyl_); 7.47 (*d*, *J* = 8.6 Hz, 1H, 5-H_quinazolinyl_); 7.35–7.22 (*m*, 6H, 2/6-H_phenyl_, 3/5-H_phenyl_, 4-H_phenyl_, 7-H_quinazolinyl_); 6.32 (*s*, 1H, 3-H_quinazolinyl_); 5.55 (*s*, 2H, C*H*_2_), 2.75–2.64 (*m*, 1H, H_cyclohexyl_); 2.02–1.92 (*m*, 2H. H_cyclohexyl_); 1.83–1.72 (*m*, 2H, H_cyclohexyl_); 1.71–1.62 (*m*, 1H, H_cyclohexyl_); 1.51–1.31 (*m*, 4H, H_cyclohexyl_), 1.28–1.18 (*m*, 1H, H_cyclohexyl_). ^13^C-NMR (151 MHz, DMSO-*d_6_*): *δ* (ppm) = 163.4 (1C, C-2_quinazolinyl_); 155.5 (1C, C-9_quinazolinyl_, *C*=O); 145.5 (1C, C-3a_quinazolinyl_); 140.2 (1C, C-4a_quinazolinyl_); 136.0 (C-1_phenyl_); 135.3 (1C, C-6_quinazolinyl_); 129.3 (2C, C_phenyl_); 128.8 (1C, C-8_quinazolinyl_); 127.9 (2C, C_phenyl_); 126.8 (1C, C_phenyl_); 122.1 (1C, C-7_quinazolinyl_); 114.5 (1C, C-5_quinazolinyl_); 113.6 (1C, C-8a_quinazolinyl_); 86.8 (1C, C-3_quinazolinyl_); 50.9 (1C, *C*H_2_); 38.1 (1C, C_cyclohexyl_); 32.5 (2C, C_cyclohexyl_); 26.1 (2C, C_cyclohexyl_); 26.1 (1C, C_cyclohexyl_). IR (neat): *ṽ* [cm^−1^] = 2924, 2851, 2361, 1738, 1697, 1593, 1570, 1487, 1356, 1287, 1229, 1202, 1175, 1042, 1024, 939, 860, 748, 708. HRMS (APCI): *m*/*z* = 358.1892, calculated for C_23_H_24_N_3_O^+^ [M + H]^+^ 358.1914. HPLC: *t*_R_ = 22.2 min, purity: 98.1%.

*4-[(5-chlorothiophen-2-yl)methyl]-2-cyclohexylpyrazolo[5,1-b]quinazolin-9(4H)-one* (**10i**). According to general procedure E, **9i** (40 mg, 76.1 µmol), CuI (2.9 mg, 15.2 µmol, 0.20 eq.), Cs_2_CO_3_ (49.6 mg, 152 µmol, 2.00 eq.) and 1,10-phenanthroline (2.7 mg, 15.2 µmol, 0.20 eq.) were dissolved in dry DMF (2 mL) and stirred at 80 °C for 45 min. Flash column chromatography (CH_2_Cl_2_/CH_3_OH = 97/3) yielded **10i** as a colorless solid (21.8 mg, 72%). M.p.: 210 °C. TLC: *R*_f_ = 0.56 (CH_2_Cl_2_/CH_3_OH = 95/5). ^1^H-NMR (500 MHz, DMSO-*d_6_*): *δ* (ppm) = 8.29 (*dd*, *J* = 8.0/1.6 Hz, 1H, 8-H_quinazolinyl_); 7.84 (*ddd*, *J* = 8.7/6.9/1.7 Hz, 1H, 6-H_quinazolinyl_); 7.76 (*d*, *J* = 8.6 Hz, 1H, 5-H_quinazolinyl_); 7.34 (*t*, *J* = 7.2 Hz, 1H, 7-H_quinazolinyl_); 7.16 (*d*, *J* = 3.8 Hz, 1H, 3-H_chlorothiophenyl_); 6.99 (*d*, *J* = 3.8 Hz, 1H, 4-H_chlorothiophenyl_); 6.47 (*s*, 1H, 3-H_quinazolinyl_); 5.66 (*s*, 2H, C*H*_2_); 2.77–2.67 (*m*, 1H, H_cyclohexyl_); 2.04–1.92 (*m*, 2H, H_cyclohexyl_); 1.83–1.75 (*m*, 2H, H_cyclohexyl_); 1.73–1.65 (*m*, 1H, H_cyclohexyl_); 1.57–1.30 (*m*, 4H, H_cyclohexyl_); 1.32–1.17 (*m*, 1H, H_cyclohexyl_). ^13^C-NMR (126 MHz, DMSO-*d_6_*): *δ* (ppm) = 163.4 (1C, C-2_quinazolinyl_); 155.3 (1C, C-9_quinazolinyl_, *C*=O); 144.5 C-3a_quinazolinyl_); 139.5 (1C, C-4a_quinazolinyl_); 137.2 (1C, C-2_chlorothiophenyl_); 135.4 (1C, C-6_quinazolinyl_); 129.0 (1C, C-8_quinazolinyl_); 128.2 (1C, C-5_chlorothiophenyl_); 128.0 (1C, C-3_chlorothiophenyl_); 126.9 (1C, C-4_chlorothiophenyl_); 122.4 (1C, C-7_quinazolinyl_); 114.4 (1C, C-5_quinazolinyl_); 113.8 (1C, C-8a_quinazolinyl_); 87.1 (1C, C-3_quinazolinyl_); 46.3 (1C, *C*H_2_). IR (neat): *ṽ* [cm^−1^] = 2932, 2851, 1697, 1595, 1574, 1487, 1470, 1443, 1389, 1287, 1198, 1061, 974, 856, 746, 712. HRMS (APCI): *m*/*z* = 398.1099, calculated for C_21_H_21_ClN_3_OS^+^ [M + H]^+^ 398.1088. HPLC: *t*_R_ = 23.2 min, purity: 97.8%.

*2-iodo-N-[3-(pyridin-3-yl)-1H-pyrazol-5-yl]benzamide* (**14**). According to general procedure D, aminopyrazole **7a** (925 mg, 1.40 mmol) was acylated using 2-iodobenzoyl chloride (449 mg, 1.69 mmol, 1.20 eq.) in dry pyridine/THF (16 mL/8 mL). Flash column chromatography (CH/EtOAc = 1/0 → 0/1) yielded **14** as a colorless solid (344 mg, 63%). M.p.: 205–206 °C. TLC: *R*_f_ = 0.09 (CH/EtOAc = 20/80). ^1^H-NMR (400 MHz, DMSO-*d_6_*): *δ* (ppm) = 13.08 (*bs*, 1H, N*H*_pyrazolyl_); 10.98 (bs, 1H, N*H*); 9.00 (*dd*, J = 2.3/0.9 Hz, 1H, 2-H_pyridyl_); 8.55 (*d*, *J* = 4.7 Hz, 1H, 6-H_pyridyl_); 8.15 (*ddd*, *J* = 8.0/2.3/1.6 Hz, 1H, 4-H_pyridyl_); 7.92 (*d*, *J* = 7.9 Hz, 1H, 3-H_benzoyl_); 7.54–7.39 (*m*, 3H, 5/6-H_benzoyl_, 5-H_pyridyl_); 7.22 (*t*, *J* = 7.5 Hz, 1H, 4-H_benzoyl_), the signal of 4-H_pyrazolyl_ cannot be seen on the spectrum. ^13^C-NMR (101 MHz, DMSO-*d_6_*): *δ* (ppm) = 167.2 (1C, *C*=O); 149.4 (1C, C-6_pyridyl_); 146.7 (1C, C-2_pyridyl_); 132.8 (1C, C-4_pyridyl_); 131.5 (1C, C-4_benzoyl_); 128.7 (1C, C-6_benzoyl_); 128.4 (1C, C-5_benzoyl_); 124.4 (1C, C-5_pyridyl_); 94.1 (1C, C-2_benzyol_), the signals of C-3_pyrazolyl_, and C-4_pyrazolyl_, C-5_pyrazolyl_, C-3_pyridyl_ and C-1_benzoyl_ cannot be seen on the spectrum. IR (neat): *ṽ* [cm^−1^] = 3389, 1684, 1597, 1578, 1522, 1396, 1306, 1016, 961, 895, 799, 735. HRMS (APCI): *m*/*z* = 391.0042, calculated for C_15_H_12_IN_4_O^+^ [M + H]^+^ 391.0050. HPLC: *t*_R_ = 13.3 min, purity: 96.2%.

*2-(pyridin-3-yl)pyrazolo[1,5-a]quinazolin-5(4H)-one* (**15**). According to general procedure E, **14** (150 mg, 384 µmol), CuI (14.6 mg, 76.9 µmol, 0.20 eq.), Cs_2_CO_3_ (251 mg, 769 µmol, 2.00 eq.) and 1,10-phenanthroline (13.9 mg, 76.9 µmol, 0.20 eq.) were dissolved in dry DMF (8 mL) and stirred at 80 °C for 1 h. Flash column chromatography (CH_2_Cl_2_/CH_3_OH = 1/0 → 95/5) yielded **15** as a colorless solid (21.3 mg, 21%). M.p.: >300 °C. TLC: *R*_f_ = 0.15 (CH_2_Cl_2_/CH_3_OH = 95/5). ^1^H-NMR (400 MHz, DMSO-*d_6_*): *δ* (ppm) = 12.38 (*s*, 1H, N*H*); 9.18 (*dd*, *J* = 2.2/0.9 Hz, 1H, 2-H_pyridyl_); 8.60 (*dd*, *J* = 4.7/1.7 Hz, 1H, 6-H_pyridyl_); 8.34 (*dt*, *J* = 7.9/2.0 Hz, 1H, 4-H_pyridyl_); 8.22–8.13 (*m*, 2H, H_quinazolinyl_); 7.92 (*ddd*, *J* = 8.4/7.3/1.5 Hz, 1H, H_quinazolinyl_); 7.56–7.47 (*m*, 2H, 5-H_pyridyl_, H_quinazolinyl_); 6.51 (*s*, 1H, 3-H_quinazolinyl_). ^13^C-NMR (101 MHz, DMSO-*d_6_*): *δ* (ppm) = 158.9 (1C, C-5_quinazolinyl_, *C*=O); 150.4 (1C, C-2_quinazolinyl_); 150.0 (1C, C-6_pyridyl_); 147.4 (1C, C-2_pyridyl_); 140.4 (1C, C-3a_quinazolinyl_); 137.6 (1C, C-_quinazolinyl_); 135.6 (1C, C-_quinazolinyl_); 133.6 (1C, C-4_pyridyl_); 128.7 (1C, C_quinazolinyl_); 128.6 (1C, C-3_pyridyl_); 126.0 (1C, C_quinazolinyl_); 124.4 (1C, C-5_pyridyl_); 116.9 (1C, C_quinazolinyl_); 114.9 (1C, C_quinazolinyl_); 86.6 (1C, C-3_quinazolinyl_). IR (neat): *ṽ* [cm^−1^] = 3115, 1659, 1574, 1479, 1454, 1425, 1375, 1321, 1188, 1134, 916, 791, 760, 704. HRMS (APCI): *m*/*z* = 263.0948, calculated for C_15_H_11_N_4_O^+^ [M + H]^+^ 263.0927. HPLC: *t*_R_ = 12.9 min, purity: 99.8%.

*(5-amino-3-(pyridin-3-yl)-1H-1,2,4-triazol-1-yl)(2-iodophenyl)methanone* (**16**). According to general procedure D, aminotriazole **11** (500 mg, 3.10 mmol) was acylated using 2-iodobenzoyl chloride (1.24 g, 4.65 mmol) in dry pyridine/THF (5 mL/5 mL). After the washing steps, pure product **16** was obtained as a colorless solid (844 mg, 70%). M.p.: 220 °C. TLC: *R*_f_ = 0.87 (EtOAc/MeOH= 9/1). ^1^H NMR (600 MHz, DMSO-*d*_6_) δ (in ppm) = 7.32 (td, *J* = 7.7, 1.7 Hz, 1H, 4-H_phenyl_), 7.46 (ddd, *J* = 7.9, 4.8, 0.9 Hz 1H, 5-H_pyridyl_), 7.56 (td, *J* = 7.5, 1.1 Hz, 1H, 5-H_phenyl_), 7.64 (dd, *J* = 7.6, 1.7 Hz_l_, 1H, 6-H_phenyl_), 7.97 (dd, *J* = 8.0, 1.1 Hz, 1H, 3-H_phenyl_), 8.02 (s, 2H, NH_2_), 8.10 (dt, *J* = 7.9, 2.0 Hz, 1H, 4-H_pyridyl_), 8.63 (dd, *J* = 4.8, 1.7 Hz, 1H, 6-H_pyridyl_), 8.97 (d, *J* = 2.2 Hz, 1H, 2-H_pyridyl_). ^13^C NMR (151 MHz, DMSO-*d*_6_) δ (in ppm) = 93.3 (1C, C-2_phenyl_), 123.9 (1C, C-5_pyridyl_), 125.8 (1C, C-3_pyridyl_), 127.8 (1C, C-5_phenyl_), 129.0 (1C, C-6_phenyl_), 132.0 (1C, C-4_phenyl_), 133.7 (1C, C-4_pyridyl_), 138.7 (1C, C-3_phenyl_), 139.7 (1C, C-1_phenyl_), 147.4 (1C, C-2_pyridyl_), 151.0 (1C, C-6_pyridyl_), 158.2 (1C, C-5_triazolyl_), 158.4 (1C, C-3_triazolyl_), 168.6 (1C, CO). IR (neat): *ṽ* [cm^−1^] = 3464, 2978, 1697, 1647, 1582, 1524, 1485, 1412, 1358, 1327, 1188, 1138, 1076, 1018, 961, 921, 795, 748, 706, 682, 640. HRMS (APCI): *m*/*z* = 391.9957, calculated for C_14_H_11_N_5_O^+^ [M + H]^+^ 392.0003.

*2-iodo-N-(3-(pyridin-3-yl)-1H-1,2,4-triazol-5-yl)benzamide* (**16′**). Aminotriazole **16** (250 mg, 639.1 μmol) was heated to 260 °C (neat) for 15 min. After cooling to room temperature, the crude product was purified by flash column chromatography (CH_2_Cl_2_/CH_3_OH = 100/0→ 90/10) to yield **16′** as a colorless solid (199 mg, 509.2 μmol, 80%). M.p.: 299–300 °C. TLC: *R*_f_ = 0.32 (DCM/MeOH = 95/5). ^1^H NMR (600 MHz, DMSO-*d*_6_) δ (in ppm) = 7.27 (t, *J* = 7.2 Hz, 1H, 4-H_benzoyl_), 7.52 (t, *J* = 7.2 Hz, 2H, 5-H_benzoyl_/5-H_pyridyl_), 7.56 (d, *J* = 7.0 Hz, 1H, 6-H_benzoyl_), 7.96 (d, *J* = 7.9 Hz, 1H, 3-H_benzoyl_), 8.29 (d, *J* = 7.9 Hz, 1H, 4-H_pyridyl_), 8.62 (d, *J* = 3.7 Hz, 1H, 6-H_pyridyl_), 9.15 (s, 1H, 2-H_pyridyl_), 12.27 (s, 1H, NH_triazolyl_), 13.95 (s, 1H, NH). ^13^C NMR (151 MHz, DMSO-*d*_6_) δ (in ppm) = 93.5 (1C, C-2_benzoyl_), 123.9 (1C, C-5_pyridyl_), 127.0 (1C, C-3_pyridyl_), 128.0 (1C, C-5_benzoyl_), 128.5 (1C, C-6_benzoyl_), 131.8 (1C, C-4_benzoyl_), 132.9 (1C, C-4_pyridyl_), 139.2 (1C, C-3_benzoyl_), 140.5 (1C, C-1_benzoyl_), 146.7 (1C, C-2_pyridyl_), 149.2 (1C, C-5_triazolyl_), 149.9 (1C, C-6_pyridyl_), 156.0 (1C, C-3_triazolyl_), 167.7 (1C, C=O). IR (neat): *ṽ* [cm^−1^] = 3254, 3057, 1672, 1597, 1425, 1300, 1140, 1084, 1053, 1037, 1016, 889, 795, 750, 667, 638, 629. HRMS (APCI): *m*/*z* = 392.0003 calculated for [M + H]^+^, found: 391.9994. HPLC: *t_R_* = 12.8 min, purity: 97.0%.

*(5-amino-3-(pyridin-4-yl)-1H-1,2,4-triazol-1-yl)(2-iodophenyl)methanone* (**17**). According to general procedure D, aminotriazole **12** (730 mg, 4.53 mmol) was acylated using 2-iodobenzoyl chloride (1.81 g, 6.80 mmol) in dry pyridine/THF (5 mL/5 mL). After the washing steps, pure product **17** was obtained as a colorless solid (1.56 g, 86%). M.p.: 191–193 °C. TLC: *R*_f_ = 0.80 (EtOAc/MeOH = 9/1). ^1^H NMR (400 MHz, DMSO-*d*_6_) δ (in ppm) = 7.33 (t, *J* = 7.6 Hz, 1H), 7.57 (t, *J* = 7.5 Hz, 1H), 7.64 (d, *J* = 6.7 Hz, 1H), 7.70 (d, *J* = 5.7 Hz, 2H), 7.97 (d, *J* = 7.9 Hz, 1H), 8.03 (br s, 2H, NH_2_), 8.65 (d, *J* = 5.5 Hz, 2H). ^13^C-NMR (101 MHz, DMSO-*d_6_*): *δ* (ppm) = 168.8 (NCO_methanone_), 158.5 (5-C_triazolyl_), 158.4 (3-C_triazolyl_), 150.3 (2-C_pyridyl_ and 6-C_pyridyl_), 139.6 (1-C_phenyl_), 138.7 (3-C_phenyl_), 137.2 (4-C_pyridyl_), 132.0 (4-C_phenyl_), 129.0 (6-C_phenyl_), 127.9 (5-C_phenyl_), 120.5 (3-C_pyridyl_ and 5-C_pyridyl_), 93.3 (2-C_phenyl_). IR (neat): *ṽ* [cm^−1^] = 3310, 3163, 1709, 1643, 1524, 1493, 1462, 1420, 1358, 1323, 1138, 1038, 968, 922, 741, 675. HRMS (APCI): *m*/*z* = 391.9946, calculated for C_14_H_11_N_5_O^+^ [M + H]^+^ 392.0003.

*2-iodo-N-(3-(pyridin-4-yl)-1H-1,2,4-triazol-5-yl)benzamide* (**17′**). Aminotriazole **17** (207 mg, 529.2 μmol) was heated to 260 °C (neat) for 15 min. After cooling to room temperature, the crude product was purified by flash column chromatography (CH_2_Cl_2_/CH_3_OH = 100/0→ 90/10) to yield **17′** as a colorless solid (158 mg, 403.9 μmol, 76%). M.p.: >300 °C. TLC: *R*_f_ = 0.31 (DCM/MeOH = 95/5). ^1^H NMR (600 MHz, DMSO-*d*_6_) δ (in ppm) = 7.27 (t, *J* = 10.9, 4.3 Hz, 1H, 4-H_benzoyl_), 7.52 (t, *J* = 7.4 Hz, 1H, 5-H_benzoyl_), 7.64 (d, *J* = 7.1 Hz, 1H, 6-H_benzoyl_), 7.88 (d, *J* = 5.9 Hz, 2H, 3-H_pyridyl_/5-H_pyridyl_), 7.96 (d, *J* = 7.8 Hz, 1H, 3-H_benzoyl_), 8.68 (d, *J* = 4.5 Hz, 1H, 2-H_pyridyl_/6-H_pyridyl_), 12.29 (s, 1H, NH_triazolyl_), 14.08 (s, 1H, NH). ^13^C NMR (151 MHz, DMSO-*d*_6_) δ (in ppm) = 93.5 (1C, C-2_benzoyl_), 119.8 (1C, C-3_pyridyl_/C-5_pyridyl_), 128.0 (1C, C-5_benzoyl_), 128.6 (1C, C-6_benzoyl_), 131.8 (1C, C-4_benzoyl_), 138.2 (1C, C-4_pyridyl_), 139.2 (1C, C-3_benzoyl_), 140.4 (1C, C-1_benzoyl_), 149.4 (1C, C-5_triazolyl_), 150.3 (C-2_pyridyl_/C-6_pyridyl_), 156.3 (1C, C-3_triazolyl_), 167.8 (1C, C=O). IR (neat): *ṽ* [cm^−1^] = 3347, 1659, 1422, 1306, 1138, 1090, 1053, 1016, 1003, 978, 953, 878, 837, 795, 746, 727, 704, 669, 635. HRMS (APCI): *m*/*z* = 392.0003 calculated for [M + H]^+^, found: 391.9996. HPLC: *t_R_* = 12.8 min, purity: 98.6%.

*(5-{[(5-chlorothiophen-2-yl)methyl]amino}-3-(pyrazin-2-yl)-1H-1,2,4-triazol-1-yl)(2-iodophenyl)methanone* (**18**). According to general procedure D, aminotriazole **13** (150 mg, 512 µmol) was acylated using 2-iodobenzoyl chloride (163 mg, 615 µmol, 1.20 eq.) in dry pyridine/THF (4.80 mL/2.40 mL). Flash column chromatography (CH/EtOAc = 1/0 → 0/1) yielded **18** as a yellowish solid (157 mg, 300 µmol, 59%). M.p.: 159–160 °C. TLC: *R*_f_ = 0.10 (CH/EtOAc = 70/30). ^1^H NMR (600 MHz, DMSO-*d*_6_): *δ* (ppm) = 4.80 (d, *J* = 6.2 Hz, 2H, CH_2_), 6.99 (d, *J* = 3.8 Hz, 1H, 4-H_thiophenyl_), 7.04–7.06 (m, 1H, 3-H_pyrazinyl_), 7.34 (td, *J* = 7.7/1.6 Hz, 1H, 4-H_iodobenzoyl_), 7.57 (td, *J* = 7.6/1.1 Hz, 1H, 5-H_iodobenzoyl_), 7.66 (dd, *J* = 7.7/1.6 Hz, 1H, 6-H_iodobenzoyl_), 7.98 (dd, *J* = 7.9/1.1 Hz, 1H, 3-H_iodobenzoyl_), 8.71 (dd, *J* = 2.5/1.5 Hz, 1H, 6-H_pyrazinyl_), 8.74 (d, *J* = 2.5 Hz, 1H, 5-H_pyrazinyl_), 8.79 (t, *J* = 6.3 Hz, 1H, NH), 9.16 (d, *J* = 1.5 Hz, 1H, 3-H_pyrazinyl_). ^13^C NMR (151 MHz, DMSO-*d*_6_): *δ* (ppm) = 41.8 (1C, CH_2_), 93.3 (1C, C-2_iodobenzoyl_), 126.2 (1C, C-4_thiophenyl_), 126.7 (1C, C-3_thiophenyl_), 127.3 (1C, C-5_thiophenyl_), 127.9 (1C, C-5_iodobenzoyl_), 129.0 (1C, C-6_iodobenzoyl_), 132.1 (1C, C-4_iodobenzoyl_), 138.7 (1C, C-3_iodobenzoyl_), 139.4 (1C, C-1_iodobenzoyl_), 140.5 (1C, C-2_thiophenyl_), 143.5 (1C, C-3_pyrazinyl_), 144.1 (1C, C-2_pyrazinyl_), 144.8 (1C, C-6_pyrazinyl_), 145.9 (1C, C-5_pyrazinyl_), 157.8 (1C, C-3/5_triazolyl_), 157.8 (1C, C-3/5_triazolyl_), 168.8 (1C, CON). IR (neat): *ṽ* [cm^−1^] = 3371, 2924, 1697, 1535, 1373, 1338, 1219, 1157, 1049, 921, 771, 748. HRMS (APCI): *m*/*z* = 522.9599 calculated for [M + H]^+^, found: 522.9613. HPLC: *t*_R_ = 22.3 min, purity: 93.8%.

*4-[(5-chlorothiophen-2-yl)methyl]-2-(pyrazin-2-yl)-[1,2,4]triazolo[5,1-b]quinazolin-9(4H)-one* (**19**). According to general procedure E, **18** (73.0 mg, 140 µmol, 1.00 eq.), CuI (65.3 mg, 27.9 µmol, 0.20 eq.), Cs_2_CO_3_ (91.0 mg, 279 µmol, 2.00 eq.) and 1,10-phenanthroline (5.0 mg, 27.9 µmol, 0.20 eq.) were dissolved in dry DMF (2.8 mL) and stirred at 80 °C for 30 min. Flash column chromatography (CH_2_Cl_2_/CH_3_OH = 1/0 → 95/5) yielded **19** as a colorless solid (44 mg, 111 µmol, 80%). M.p.: 246–247 °C (decomp.). TLC: *R*_f_ = 0.55 (CH_2_Cl_2_/CH_3_OH = 95/5). ^1^H NMR (600 MHz, DMSO-*d*_6_): *δ* (ppm) = 5.94 (s, 2H, CH_2_), 7.00 (d, *J* = 3.9 Hz, 1H, 4-H_thiophenyl_), 7.25–7.27 (m, 1H, 3-H_thiophenyl_), 7.51 (ddd, *J* = 8.0/4.6/3.5 Hz, 1H, 7-H_quinazolinyl_), 7.94–7.98 (m, 2H, 5/6-H_quinazolinyl_), 8.38 (dt, *J* = 8.0/1.1 Hz, 1H, 8-H_quinazolinyl_), 8.83 (d, *J* = 2.4 Hz, 1H, 5-H_pyrazinyl_), 8.87 (dd, *J* = 2.5/1.5 Hz, 1H, 6-H_pyrazinyl_), 9.45 (d, *J* = 1.5 Hz, 1H, 3-H_pyrazinyl_). ^13^C NMR (151 MHz, DMSO-*d*_6_): *δ* (ppm) = 44.7 (1C, CH_2_), 115.1 (1C, C-5/8a_quinazolinyl_), 115.2 (1C, C-5/8a_quinazolinyl_), 123.7 (1C, C-7_quinazolinyl_), 126.4 (1C, C-4_thiophenyl_), 127.9 (1C, C-3_thiophenyl_), 128.3 (1C, C-5_thiophenyl_), 128.5 (1C, C-8_quinazolinyl_), 135.7 (1C, C-6_quinazolinyl_), 136.1 (1C, C-2_thiophenyl_), 138.7 (1C, C-4a_quinazolinyl_), 143.7 (1C, C-3_pyrazinyl_), 144.3 (1C, C-2_pyrazinyl_), 145.0 (1C, C-6_pyrazinyl_), 146.1 (1C, C-5_pyrazinyl_), 52.7 (1C, C-3a_quinazolinyl_), 155.0 (1C, CON), 159.2 (1C, C-2_quinazolinyl_). IR (neat): *ṽ* [cm^−1^] = 3082, 1705, 1604, 1562, 1489, 1442, 1334, 1157, 1014, 933, 860, 794, 759, 717. HRMS (APCI): *m*/*z* = 395.0476 calculated for [M + H]^+^, found: 395.0462. HPLC: *t*_R_ = 19.7 min, purity: 95.1%.

*2-(pyridin-3-yl)-[1,2,4]triazolo[5,1-b]quinazolin-9(4H)-one* (**20**). According to general procedure E, **20** was synthesized using **16** (50 mg, 127.8 μmol, 1.00 eq.), CuI (5 mg, 25.6 μmol, 0.20 eq.), Cs_2_CO_3_ (83 mg, 255.6 μmol, 2.00 eq.) and 1,10-phenanthroline (5 mg, 25.6 μmol, 0.20 eq.) in dry DMF (2 mL). The mixture was stirred at 80 °C for 1 h. After the first purification (CH_2_Cl_2_/CH_3_OH = 100/0 → 90/10), product **20** was obtained as a mixture with compound **21** (**20** + **21**, 27 mg, 101.2 μmol, 79%). According to HPLC measurement **20**:**21** ratio was 1:1. Synthesis in microwave conditions (procedure F) allowed increasing the yield of the isomers’ mixture up to 92% (**20** + **21**, 31 mg, 127.8 μmol) with the ratio 7:3. Further purification (CH_2_Cl_2_/CH_3_OH = 97/3) gave pure product **20** as a colorless solid (21 mg, 78.6 μmol, 62%). M.p.: >300 °C. TLC: *R*_f_ = 0.33 (DCM/MeOH = 95/5). ^1^H NMR (600 MHz, DMSO-*d*_6_) δ (in ppm) = 7.41 (ddd, *J* = 8.1, 7.2, 1.0 Hz, 1H, 7-H_quinazolinyl_), 7.52 (d, *J* = 8.0 Hz, 1H, 5-H_quinazolinyl_), 7.60 (dd, *J* = 7.8, 4.8 Hz, 1H, 5-H_pyridyl_), 7.86 (ddd, *J* = 8.5, 7.1, 1.5 Hz, 1H, 6-H_quinazolinyl_), 8.25 (dd, *J* = 8.1, 1.2 Hz, 1H, 8-H_quinazolinyl_), 8.48 (dt, *J* = 7.9, 1.9 Hz, 1H, 4-H_pyridyl_), 8.73 (d, *J* = 3.0 Hz, 1H, 6-H_pyridyl_), 9.31 (s, 1H, 2-H_pyridyl_), 13.42 (s, 1H, NH). ^13^C NMR (151 MHz, DMSO-*d*_6_) δ (in ppm) = 113.5 (1C, C-8a_quinazolinyl_), 116.9 (1C, C-5_quinazolinyl_), 123.0 (1C, C-7_quinazolinyl_), 124.1 (1C, C-5_pyridyl_), 126.0 (1C, C-3_pyridyl_), 127.5 (1C, C-8_quinazolinyl_), 134.2 (1C, C-4_pyridyl_), 135.2 (1C, C-6_quinazolinyl_), 139.3 (1C, C-4a_quinazolinyl_), 147.7 (1C, C-2_pyridyl_), 151.3 (1C, C-6_pyridyl_), 151.6 (1C, C-3a_quinazolinyl_), 155.5 (1C, C-9_quinazolinyl_, C=O), 159.7 (1C, C-2_quinazolinyl_). IR (neat): *ṽ* [cm^−1^] = 3059, 2666, 1651, 1566, 1524, 1481, 1420, 1319, 1204, 1138, 1038, 945, 813, 787, 748, 702, 683, 633. HRMS (APCI): *m*/*z* = 264.0880 calculated for [M + H]^+^, found: 264.0889. HPLC: *t_R_* = 11.8 min, purity: 100.0%.

*2-(pyridin-3-yl)-[1,2,4]triazolo[1,5-a]quinazolin-5(4H)-one* (**21**). According to general procedure E, **21** was synthesized using **16′** (191 mg, 487.0 μmol, 1.00 eq.), CuI (19 mg, 97.4 μmol, 0.20 eq.), Cs_2_CO_3_ (317 mg, 974.0 μmol, 2.00 eq.) and 1,10-phenanthroline (18 mg, 97.4 μmol, 0.20 eq.) in dry DMF (3 mL). The mixture was stirred at 80 °C for 1 h. After purification, product **21** was obtained as a colorless solid (86 mg, 327.4 μmol, 67%); M.p.: >300 °C. TLC: *R*_f_ = 0.36 (DCM/MeOH = 95/5). ^1^H NMR (600 MHz, DMSO-*d*_6_) δ (in ppm) = 7.52–7.66 (m, 2H, 4-H_pyridyl_/7-H_quinazolinyl_), 7.97 (t, *J* = 8.3 Hz, 1H, 8-H_quinazolinyl_), 8.10 (d, *J* = 8.1 Hz, 1H, 9-H_quinazolinyl_), 8.21 (d, *J* = 7.8 Hz, 1H, 6-H_quinazolinyl_), 8.41 (d, *J* = 7.9 Hz, 1H, 4-H_pyridyl_), 8.67–8.72 (m, 1H, 6-H_pyridyl_), 9.26 (s, 1H, 2-H_pyridyl_), 13.18 (s, 1H, NH). ^13^C NMR (151 MHz, DMSO-*d*_6_) δ (in ppm) = 114.7 (1C, C-9_quinazolinyl_), 117.1 (1C, C-5a_quinazolinyl_), 124.1 (1C, C-5_pyridyl_), 126.1 (1C, C-3_pyridyl_), 126.3 (1C, C-7_quinazolinyl_), 128.4 (1C, C-6_quinazolinyl_), 133.6 (1C, C-4_pyridyl_), 135.4 (1C, C-8_quinazolinyl_), 135.6 (1C, C-9a_quinazolinyl_), 147.2 (1C, C-2_pyridyl_), 149.1 (1C, C-3a_quinazolinyl_), 150.8 (1C, C-6_pyridyl_), 158.5 (1C, C-2_quinazolinyl_), 159.7 (1C, C-5_quinazolinyl_, C=O). IR (neat): *ṽ* [cm^−1^] = 2708, 1690, 1609, 1582, 1524, 1481, 1416, 1393, 1319, 1123, 1038, 818, 752, 706, 633. HRMS (APCI): *m*/*z* = 264.0880 calculated for [M + H]^+^, found: 264.0898. HPLC: *t_R_* = 12.2 min, purity: 100.0%.

*2-(pyridin-4-yl)-[1,2,4]triazolo[5,1-b]quinazolin-9(4H)-one* (**22**). According to general procedure E, **22** was synthesized using **17** (50 mg, 127.8 μmol, 1.00 eq.), CuI (5 mg, 25.6 μmol, 0.20 eq.), Cs_2_CO_3_ (83 mg, 255.6 μmol, 2.00 eq.) and 1,10-phenanthroline (5 mg, 25.6 μmol, 0.20 eq.) in dry DMF (2 mL). The mixture was stirred at 80 °C for 1 h. After the first purification (CH_2_Cl_2_/CH_3_OH = 100/0 → 90/10), product **22** was obtained as a mixture with compound **23** (**22** + **23**, 23 mg, 86.6 μmol, 68%). According to HPLC measurement **22**:**23** ratio was 3:7. Synthesis in microwave conditions (procedure F) allowed increasing the yield of the isomers’ mixture up to 95% (**22** + **23**, 32 mg, 121.9 μmol), but the ratio remained the same. Further purification (CH_2_Cl_2_/CH_3_OH/DMEA = 95/4/1) gave pure product **22** as a colorless solid (9 mg, 35.7 μmol, 28%); M.p.: >300 °C. TLC: *R*_f_ = 0.29 (DCM/MeOH = 95/5). ^1^H NMR (600 MHz, DMSO-*d*_6_) δ (in ppm) = 7.40 (t, *J* = 7.4 Hz, 1H, 7-H_quinazolinyl_), 7.53 (d, *J* = 8.2 Hz, 1H, 5-H_quinazolinyl_), 7.85 (t, *J* = 7.3 Hz, 1H, 6-H_quinazolinyl_), 8.07 (s, 2H, 3-H_pyridyl_/5-H_pyridyl_), 8.25 (d, *J* = 7.8 Hz, 1H, 8-H_quinazolinyl_), 8.79 (s, 2H, 2-H_pyridyl_/6-H_pyridyl_), 13.38 (br s, 1H, NH). ^13^C NMR (151 MHz, DMSO-*d*_6_) δ (in ppm) = 113.5 (1C, C-8a_quinazolinyl_), 117.2 (1C, C-5_quinazolinyl_), 120.8 (2C, C-3_pyridyl_/C-5_pyridyl_), 122.9 (1C, C-7_quinazolinyl_), 127.5 (1C, C-8_quinazolinyl_), 135.2 (1C, C-6_quinazolinyl_), 137.4 (1C, C-4_pyridyl_), 139.8 (1C, C-4a_quinazolinyl_), 150.6 (2C, C-2_pyridyl_/C-6_pyridyl_), 151.9 (1C, C-2_quinazolinyl_), 155.6 (1C, C-3a_quinazolinyl_), 159.8 (1C, C-9_quinazolinyl_, C=O). IR (neat): *ṽ* [cm^−1^] = 2978, 2924, 2662, 1697, 1643, 1608, 1570, 1481, 1427, 1389, 1207, 1161, 1065, 1003, 953, 837, 791, 748, 714, 679. HRMS (APCI): *m*/*z* = 264.0880 calculated for [M + H]^+^, found: 264.0862. HPLC: *t_R_* = 11.5 min, purity: 100.0%.

*2-(pyridin-4-yl)-[1,2,4]triazolo[1,5-a]quinazolin-5(4H)-one* (**23**). According to general procedure E, **23** was synthesized using **17′** (99 mg, 253.1 μmol, 1.00 eq.), CuI (10 mg, 50.6 μmol, 0.20 eq.), Cs_2_CO_3_ (165 mg, 506.2 μmol, 2.00 eq.) and 1,10-phenanthroline (9 mg, 50.6 μmol, 0.20 eq.) in dry DMF (2 mL). The mixture was stirred at 80 °C for 1 h. After purification, product **23** was obtained as a colorless solid (28 mg, 107.5 μmol, 42%); M.p.: >300 °C. TLC: *R*_f_ = 0.31 (DCM/MeOH = 95/5). ^1^H NMR (600 MHz, DMSO-*d*_6_) δ (in ppm) = 7.58–7.63 (m, 1H, 7-H_quinazolinyl_), 7.97 (dd, *J* = 8.3, 1.2 Hz, 1H, 8-H_quinazolinyl_), 8.00 (dd, *J* = 4.5, 1.6 Hz, 2H, 3-H_pyridyl_/5-H_pyridyl_), 8.10 (d, *J* = 8.1 Hz, 1H, 9-H_quinazolinyl_), 8.22 (dd, *J* = 7.9, 1.0 Hz, 1H, 6-H_quinazolinyl_), 8.75 (dd, *J* = 4.5, 1.4 Hz, 2H, 2-H_pyridyl_/6-H_pyridyl_), 13.21 (s, 1H, NH). ^13^C NMR (151 MHz, DMSO-*d*_6_) δ (in ppm) = 114.7 (1C, C-9_quinazolinyl_), 117.2 (1C, C-5a_quinazolinyl_), 120.2 (2C, C-3_pyridyl_/C-5_pyridyl_), 126.5 (1C, C-7_quinazolinyl_), 128.4 (1C, C-6_quinazolinyl_), 135.4 (1C, C-8_quinazolinyl_), 135.6 (1C, C-9a_quinazolinyl_), 137.3 (2C, C-4_pyridyl_), 149.2 (1C, C-3a_quinazolinyl_), 150.5 (2C, C-2_pyridyl_/C-6_pyridyl_), 158.5 (1C, C-2_quinazolinyl_), 159.7 (1C, C-5_quinazolinyl_, C=O). IR (neat): *ṽ* [cm^−1^] = 2978, 2920, 2662, 1682, 1609, 1485, 1427, 1381, 1119, 1011, 837, 748, 698, 679. HRMS (APCI): *m*/*z* = 264.0880 calculated for [M + H]^+^, found: 264.0902. HPLC: *t_R_* = 11.8 min, purity: 98.9%.

*1-{5-[(4-methoxybenzyl)amino]-3-(pyridin-3-yl)-1H-pyrazol-1-yl}-2,2-dimethylpropan-1-one* (**24b**). According to general procedure D, aminopyrazole **8b** (100 mg, 357 µmol) was acylated using pivaloyl chloride (55 µL, 428 µmol, 1.20 eq.) in dry pyridine/THF (4.0 mL/2.0 mL). Flash column chromatography (CH/EtOAc = 1/0 → 0/1) yielded **24b** as a yellowish solid (56.7 mg, 44%). M.p.: 130 °C. TLC: *R*_f_ = 0.52 (CH/EtOAc = 1/1). ^1^H-NMR (600 MHz, DMSO-*d_6_*): *δ* (ppm) = 9.01 (*dd*, *J* = 2.3/0.9 Hz, 1H, 2-H_pyridyl_); 8.59 (*dd*, *J* = 4.8/1.6 Hz, 1H, 6-H_pyridyl_); 8.16 (*ddd*, *J* = 7.9/2.3/1.7 Hz, 1H, 4-H_pyridyl_); 7.73 (*t*, *J* = 6.1 Hz, 1H, N*H*); 7.48 (*dd*, *J* = 7.9/4.8/0.9 Hz, 1H, 5-H_pyridyl_); 7.38–7.33 (*m*, 2H, 2/6-H_methoxyphenyl_); 6.94–6.86 (*m*, 2H, 3/5-H_methoxyphenyl_); 6.03 (*s*, 1H, 4-H_pyrazolyl_); 4.31 (*d*, *J* = 6.1 Hz, 2H, C*H*_2_); 3.72 (*s*, 3H, C*H*_3_); 1.48 (*s*, 9H, C(C*H*_3_)_3_). ^13^C-NMR (151 MHz, DMSO-*d_6_*): *δ* (ppm) = 179.7 (1C, *C*=O); 158.4 (1C, C-4_methoxyphenyl_); 153.8 (1C, C-5_pyrazolyl_); 150.2 (1C, C-3_pyrazolyl_); 149.9 (1C, C-6_pyridyl_); 146.9 (1C, C-2_pyridyl_); 133.0 (1C, C-4_pyridyl_); 130.5 (1C, C-1_methoxyphenyl_); 128.8 (2C, C-2/6_methoxyphenyl_); 127.9 (1C, C-3_pyridyl_), 123.8 (1C, C-5_pyridyl_), 113.8 (2C, C-3/5_methoxyphenyl_); 82.6 (1C, C-4_pyrazolyl_); 54.9 (1C, *C*H_3_); 47.2 (1C, *C*H_2_); 41.7 (1C, *C*(CH_3_)_3_). IR (neat): *ṽ* [cm^−1^] = 3395, 3105, 1686, 1514, 1422, 1364, 1319, 1256, 1188, 1065, 1028, 943, 797, 772, 706. HRMS (APCI): *m*/*z* = 365.2107, calculated for C_21_H_25_N_4_O_2_^+^ [M + H]^+^ 365.1972. HPLC: *t*_R_ = 20.1 min, purity: 99.6%.

*2,2-dimethyl-1-(5-{[(naphthalen-1-yl)methyl]amino}-3-(pyridin-3-yl)-1H-pyrazol-1-yl)propan-1-one* (**24c**). According to general procedure D, aminopyrazole **8c** (150 mg, 499 µmol) was acylated using pivaloyl chloride (75 µL, 599 µmol, 1.20 eq.) in dry pyridine/THF (6.0 mL/3.0 mL). Flash column chromatography (CH/EtOAc = 1/0 → 0/1) yielded **24c** as a beige solid (76.8 mg, 40%). M.p.: 130–131 °C. TLC: *R*_f_ = 0.20 (CH/EtOAc = 80/20). ^1^H-NMR (600 MHz, DMSO-*d_6_*): *δ* (ppm) = 9.02 (*dd*, *J* = 2.2/0.9 Hz, 1H, 2-H_pyridyl_); 8.59 (*dd*, *J* = 4.8/1.7 Hz, 1H, 6-H_pyridyl_); 8.21–8.16 (*m*, 2H, 4-H_pyridyl_, 9-H_naphthyl_); 7.99–7.96 (*m*, 1H, H_naphthyl_); 7.89–7.85 (*m*, 1H, H_naphthyl_); 7.80 (*t*, *J* = 6.0 Hz, NH); 7.65–7.54 (*m*, 3H, H_naphthyl_); 7.51–7.45 (*m*, 2H, H_naphthyl_, 5-H_pyridyl_); 6.17 (*s*, 1H, 4-H_pyrazolyl_); 4.89 (*d*, *J* = 5.9 Hz, 2H, C*H*_2_), 1.49 (*s*, 9H, C(C*H*_3_)_3_). ^13^C-NMR (151 MHz, DMSO-*d_6_*): *δ* (ppm) = 179.9 (1C, *C*=O); 154.0 (1C, C-5_pyrazolyl_); 150.3 (1C, C-3_pyrazolyl_); 149.8 (1C, C-6_pyridyl_); 147.0 (1C, C-2_pyridyl_); 133.4 (1C, C_naphthyl_); 133.4 (1C, C_naphthyl_); 133.0 (1C, C-4_pyridyl_); 130.8 (1C, C_naphthyl_); 128.5 (1C, C_naphthyl_); 127.8 (1C, C-3_pyridyl_); 127.7 (1C, C_naphthyl_); 126.3 (1C, C_naphthyl_); 125.8 (1C, C_naphthyl_); 125.4 (1C, C_naphthyl_); 125.1 (1C, C_naphthyl_); 123.8 (1C, C_naphthyl_); 123.4 (1C, C-9_naphthyl_); 82.7 (1C, C-4_pyrazolyl_); 46.0 (1C, *C*H_2_); 41.8 (1C, *C*(CH_3_)_3_); 27.1 (3C, C(*C*H_3_)_3_). IR (neat): *ṽ* [cm^−1^] = 1738, 1682, 1572, 1422, 1366, 1317, 1217, 1190, 959, 795, 766, 708. HRMS (APCI): *m*/*z* = 385.2031, calculated for C_24_H_25_N_4_O^+^ [M + H]^+^ 385.2023. HPLC: *t*_R_ = 21.8 min, purity: 99.6%.

*1-(5-{[(furan-2-yl)methyl]amino}-3-(pyridin-3-yl)-1H-pyrazol-1-yl)-2,2-dimethylpropan-1-one* (**24d**). According to general procedure D, aminopyrazole **8d** (130 mg, 541 µmol) was acylated using pivaloyl chloride (80 µL, 649 µmol, 1.20 eq.) in dry pyridine/THF (6.0 mL/3.0 mL). Flash column chromatography (CH/EtOAc = 1/0 → 0/1) yielded **24d** as a brownish solid (99.1 mg, 56%). M.p.: 114–115 °C. TLC: *R*_f_ = 0.17 (CH/EtOAc = 80/20). ^1^H-NMR (600 MHz, DMSO-*d_6_*): *δ* (ppm) = 9.03 (*dd*, *J* = 2.3/0.9 Hz, 1H, 2-H_pyridyl_); 8.60 (*dd*, *J* = 4.8/1.7 Hz, 1H, 6-H_pyridyl_); 8.18 (*ddd*, *J* = 7.9/2.3/1.7 Hz, 1H, 4-H_pyridyl_); 7.65 (*t*, *J* = 6.1 Hz, 1H, N*H*); 7.61 (*dd*, *J* = 1.9/0.9 Hz, 1H, 5-H_furanyl_); 7.49 (*ddd*, *J* = 7.9/4.8/0.9 Hz, 1H, 5-H_pyridyl_); 6.48–6.45 (*m*, 1H, 3-H_furanyl_); 6.40 (*dd*, *J* = 3.2/1.8 Hz, 1H, 4-H_furanyl_); 6.15 (*s*, 1H, 4-H_pyrazolyl_); 4.40 (*d*, *J* = 6.0 Hz, 2H, C*H*_2_); 1.48 (*s*, 9H, C(C*H*_3_)_3_). IR (neat): *ṽ* [cm^−1^] = 3383, 1676, 1597, 1520, 1418, 1335, 1229, 1053, 980, 949, 814, 748, 706. HRMS (APCI): *m*/*z* = 325.1671, calculated for C_18_H_21_N_4_O_2_^+^ [M + H]^+^ 325.1659. HPLC: *t*_R_ = 19.0 min, purity: 99.3%.

*1-(5-{[(5-chlorothiophen-2-yl)methyl]amino}-3-(pyridin-3-yl)-1H-pyrazol-1-yl)-2,2-dimethylpropan-1-one* (**24e**). According to general procedure D, aminopyrazole **8e** (150 mg, 516 µmol) was acylated using pivaloyl chloride (75 µL, 619 µmol, 1.20 eq.) in dry pyridine/THF (6.0 mL/3.0 mL). Flash column chromatography (CH/EtOAc = 1/0 → 0/1) yielded **24e** as a yellowish solid (101 mg, 52%). M.p.: 96–97 °C. TLC: *R*_f_ = 0.13 (CH/EtOAc = 80/20). ^1^H-NMR (600 MHz, DMSO-*d_6_*): *δ* (ppm) = 9.02 (*dd*, *J* = 2.3/0.9 Hz, 1H, 2-H_pyridyl_); 8.60 (*dd*, *J* = 4.8/1.7 Hz, 1H, 6-H_pyridyl_); 8.17 (*ddd*, *J* = 7.9/2.2/1.7 Hz, 1H, 4-H_pyridyl_); 7.88 (*m*, 1H, N*H*); 7.49 (*ddd*, *J* = 7.9/4.8/0.9 Hz, 1H, 5-H_pyridyl_); 7.06 (*dd*, *J* = 3.7/0.9 Hz, 1H, 3-H_chlorothiophenyl_); 6.97 (*d*, *J* = 3.7 Hz, 1H, 4-H_chlorothiophenyl_); 6.14 (*s*, 1H, 4-H_pyrazolyl_); 4.51 (*dd*, *J* = 6.4/1.0 Hz, 2H, C*H*_2_); 1.48 (s, 9H, C(C*H*_3_)_3_). ^13^C-NMR (151 MHz, DMSO-*d_6_*): *δ* (ppm) = 179.5 (1C, *C*=O); 153.2 (1C, C-5_pyrazolyl_); 150.1 (1C, C-3_pyrazolyl_); 149.9 (1C, C-6_pyridyl_); 147.0 (1C, C-2_pyridyl_); 141.5 (1C, C-2_chlorothiophenyl_); 132.9 (1C, C-4_pyridyl_); 127.8 (1C, C-3_pyridyl_); 126.9 (1C, C-5_chlorothiophenyl_); 126.2 (1C, C-4_chlorothiophenyl_); 126.1 (1C, C-3_chlorothiophenyl_); 123.9 (1C, C-5_pyridyl_); 82.9 (1C, C-4_pyrazolyl_); 43.2 (1C, *C*H_2_); 41.7 (1C, *C*(CH_3_)_3_); 27.1 (3C, C(*C*H_3_)_3_). IR (neat): *ṽ* [cm^−1^] = 3374, 1692, 1574, 1454, 1422, 1364, 1317, 1217, 1005, 795. HRMS (APCI): *m*/*z* = 375.1177, calculated for C_18_H_20_ClN_4_OS^+^ [M + H]^+^ 375.1041. HPLC: *t*_R_ = 21.0 min, purity: 99.2%.

*1-(5-{[(5-chlorothiophen-2-yl)methyl]amino}-3-phenyl-1H-pyrazol-1-yl)-2,2-dimethylpropan-1-one* (**24g**). According to general procedure D, aminopyrazole **8g** (60 mg, 207 µmol) was acylated using pivaloyl chloride (30 µL, 248 µmol, 1.20 eq.) in dry pyridine/THF (2.0 mL/1.2 mL). Flash column chromatography (CH/EtOAc = 1/0 → 0/1) yielded **24g** as a yellowish solid (34.2 mg, 44%). TLC: *R*_f_ = 0.51 (CH/EtOAc = 95/5). ^1^H-NMR (600 MHz, DMSO-*d_6_*): *δ* (ppm) = 9.04 (*dd*, *J* = 2.2/0.9 Hz, 1H, H_phenyl_); 8.61 (*dd*, *J* = 4.8/1.6 Hz, 1H, H_phenyl_); 8.20 (*ddd*, *J* = 7.9/1.9 Hz, 1H, H_phenyl_); 7.65 (*t*, J = 6.1 Hz, 1H, N*H*); 7.60 (*ddd*, *J* = 1.9/0.9/0.3 Hz, 1H, H_phenyl_); 7.51 (*ddd*, *J* = 7.9/4.8/0.9 Hz, 1H, H_phenyl_); 6.46 (*d*, *J* = 3.2/0.8 Hz, 1H, 3-H_chlorothiophenyl_); 6.40 (*dd*, *J* = 3.2/1.9 Hz, 1H, 4-H_chlorothiophenyl_); 6.15 (*s*, 1H, 4-H_pyrazolyl_); 4.40 (*d*, *J* = 6.0 Hz, 2H, C*H*_2_); 1.48 (s, 9H, C(C*H*_3_)_3_). ^13^C-NMR (151 MHz, DMSO-*d_6_*): *δ* (ppm) = 179.7 (1C, *C*=O); 153.6 (1C, C-5_pyrazolyl_); 151.5 (1C, C-2_chlorothiophenyl_); 150.1 (1C, C-3_pyrazolyl_); 149.7 (1C, C_phenyl_); 146.8 (1C, C_phenyl_); 142.5 (1C, C_phenyl_); 133.1 (1C, C_phenyl_); 127.9 (1C, C-5_chlorothiophenyl_); 123.9 (1C, C_phenyl_); 110.4 (1C, C-4_chlorothiophenyl_); 107.8 (1C, C-3_chlorothiophenyl_); 82.7 (1C, C-4_pyrazolyl_); 41.7 (1C, *C*(CH_3_)_3_); 41.0 (1C, *C*H_2_); 27.1 (3C, C(*C*H_3_)_3_). IR (neat): *ṽ* [cm^−1^] = 3387, 2928, 1682, 1578, 1522, 1504, 1481, 1450, 1395, 1358, 1327, 1234, 1200, 1061, 1003, 945, 193, 731. HRMS (APCI): *m*/*z* = 374.1091, calculated for C_19_H_21_ClN_3_OS^+^ [M + H]^+^ 374.1088. HPLC: *t*_R_ = 26.5 min, purity: 98.1%.

*1-[5-(benzylamino)-3-cyclohexyl-1H-pyrazol-1-yl]-2,2-dimethylpropan-1-one* (**24h**). According to general procedure D, aminopyrazole **8h** (100 mg, 392 µmol) was acylated using pivaloyl chloride (60 µL, 470 µmol, 1.20 eq.) in dry pyridine/THF (4.0 mL/2.0 mL). Flash column chromatography (CH/EtOAc = 1/0 → 0/1) yielded **24h** as a colorless solid (70.1 mg, 53%). M.p.: 47 °C. TLC: *R*_f_ = 0.68 (CH/EtOAc = 95/5). ^1^H-NMR (500 MHz, DMSO-*d_6_*): *δ* (ppm) = 7.56 (*t*, J = 6.1 Hz, 1H, N*H*); 7.39–7.30 (*m*, 4H, 2/4/5/6-H_phenyl_); 7.25 (*t*, J = 6.7 Hz, 1H, 4-H_phenyl_); 5.21 (*s*, 1H, 4-H_pyrazolyl_); 4.28 (*d*, *J* = 5.9 Hz, 2H, C*H*_2_); 2.45–2.32 (*m*, 1H, H_cyclohexyl_); 1.89–1.79 (*m*, 2H, H_cyclohexyl_); 1.74–1.66 (*m*, 2H, H_cyclohexyl_); 1.65–1.58 (*m*, 1H, H_cyclohexyl_); 1.41 (*s*, 9H, C(C*H*_3_)_3_); 1.37–1.24 (*m*, 4H, H_cyclohexyl_); 1.25–1.13 (*m*, 1H, H_cyclohexyl_). ^13^C-NMR (126 MHz, DMSO-*d_6_*): *δ* (ppm) = 179.5 (1C, *C*=O); 160.0 (1C, C-3_pyrazolyl_); 152.9 (1C, C-5_pyrazolyl_); 138.8 (1C, C-1_phenyl_); 128.2 (2C, C-2/6_phenyl_); 127.2 (1C, C-3/5_phenyl_); 126.9 (1C, C-4_phenyl_); 82.9 (1C, C-4_pyrazolyl_); 47.9 (1C, *C*H_2_); 41.5 (1C, *C*(CH_3_)_3_); 36.9 (1C, C_cyclohexyl_); 31.3 (2C, C_cyclohexyl_); 27.0 (3C, C(*C*H_3_)_3_); 25.6 (1C, C_cyclohexyl_); 25.4 (2C, C_cyclohexyl_). IR (neat): *ṽ* [cm^−1^] = 3406, 2918, 2851, 1682, 1584, 1518, 1481, 1391, 1337, 1227, 1157, 1119, 993, 976, 947, 814, 737. HRMS (APCI): *m*/*z* = 340.2431, calculated for C_21_H_30_N_3_O^+^ [M + H]^+^ 340.2383. HPLC: *t*_R_ = 28.0 min, purity: 98.0%.

*1-(5-{[(5-chlorothiophen-2-yl)methyl]amino}-3-cyclohexyl-1H-pyrazol-1-yl)-2,2-dimethylpropan-1-one* (**24i**). According to general procedure D, aminopyrazole **8i** (50 mg, 169 µmol) was acylated using pivaloyl chloride (25 µL, 203 µmol, 1.20 eq.) in dry pyridine/THF (2.0 mL/1.2 mL). Flash column chromatography (CH/EtOAc = 1/0 → 0/1) yielded **24i** as a yellowish solid (19.3 mg, 30%). M.p.: 92–93 °C. TLC: *R*_f_ = 0.63 (CH/EtOAc = 95/5). ^1^H-NMR (600 MHz, DMSO-*d_6_*): *δ* (ppm) = 7.61 (*t*, *J* = 6.2 Hz, 1H, N*H*); 6.98–6.94 (*m*, 2H, 3/4-H_chlorothiophenyl_); 5.35 (*s*, 1H, 4-H_pyrazolyl_); 4.40 (*d*, *J* = 6.0 Hz, 2H, C*H*_2_); 2.47–2.38 (*m*, 1H, H_cyclohexyl_); 1.92–1.81 (*m*, 2H, H_cyclohexyl_); 1.76–1.68 (*m*, 2H, H_cyclohexyl_); 1.66–1.58 (*m*, 1H, H_cyclohexyl_); 1.40 (*s*, 9H, C(C*H*_3_)_3_); 1.37–1.27 (*m*, 4H, H_cyclohexyl_); 1.25–1.14 (*m*, 1H, H_cyclohexyl_). ^13^C-NMR (151 MHz, DMSO-*d_6_*): *δ* (ppm) = 179.8 (1C, *C*=O); 160.5 (1C, C-3_pyrazolyl_); 152.8 (1C, C-5_pyrazolyl_); 142.4 (1C, C-2_chlorothiophenyl_); 127.2 (1C, C-5_chlorothiophenyl_); 126.8 (1C, C-4_chlorothiophenyl_); 126.2 (1C, C-3_chlorothiophenyl_); 84.0 (1C, C-4_pyrazolyl_); 43.7 (1C, *C*H_2_); 42.0 (1C, *C*(CH_3_)_3_); 37.4 (1C, C_cyclohexyl_); 31.9 (2C, C_cyclohexyl_); 27.6 (3C, C(*C*H_3_)_3_); 26.1 (1C, C_cyclohexyl_); 25.9 (2C, C_cyclohexyl_). IR (neat): *ṽ* [cm^−1^] = 3397, 2922, 2851, 1682, 1585, 1518, 1452, 1391, 1364, 1335, 1261, 1227, 1117, 1003, 976, 941, 814, 797. HRMS (APCI): *m*/*z* = 380.1563, calculated for C_19_H_27_ClN_3_OS^+^ [M + H]^+^ 380.1558. HPLC: *t*_R_ = 28.5 min, purity: 96.6%.

*(5-{[(5-chlorothiophen-2-yl)methyl]amino}-3-(pyridin-3-yl)-1H-pyrazol-1-yl)(phenyl)methanone* (**25**). According to general procedure D, aminopyrazole **8e** (200 mg, 688 µmol) was acylated using benzoyl chloride (79.9 µL, 688 µmol, 1.00 eq.) in dry pyridine/THF (6.4 mL/3.2 mL). Flash column chromatography (CH/EtOAc = 1/0 → 0/1) yielded **25** as a colorless solid (152 mg, 56%). M.p.: 127–128 °C. TLC: *R*_f_ = 0.21 (CH/EtOAc = 70/30). ^1^H NMR (600 MHz, DMSO-*d*_6_): *δ* (ppm) = 4.59 (d, *J* = 6.3 Hz, 2H, CH_2_), 6.28 (s, 1H, 4-H_pyrazolyl_), 6.98 (d, *J* = 3.8 Hz, 1H, 4-H_thiophenyl_), 7.10–7.19 (m, 1H, 3-H_thiophenyl_), 7.46 (ddd, *J* = 7.9/4.7/0.9 Hz, 1H, 5-H_pyridyl_), 7.54–7.58 (m, 2H, 3/5-H_benzoyl_), 7.64–7.67 (m, 1H, 4-H_benzoyl_), 7.96 (t, *J* = 6.3 Hz, 1H, NH), 8.06–8.09 (m, 2H, 2/6-H_benzoyl_), 8.10–8.13 (m, 1H, 4-H_pyridyl_), 8.59 (dd, *J* = 4.8/1.7 Hz, 1H, 6-H_pyridyl_), 8.99 (dd, *J* = 2.3/0.9 Hz, 1H, 2-H_pyridyl_). ^13^C NMR (151 MHz, DMSO-d_6_): *δ* (ppm) = 43.4 (1C, CH_2_), 84.1 (1C, C-4_pyrazolyl_), 123.9 (1C, C-5_pyridyl_), 126.2 (1C, C-3_thiophenyl_), 126.3 (1C, C-4_thiophenyl_), 127.0 (1C, C-5_thiophenyl_), 127.7 (1C, C-3_pyridyl_), 127.9 (2C, C-3/5_benzoyl_), 130.9 (2C, C-2/6_benzoyl_), 132.5 (1C, C-4_benzoyl_), 132.8 (1C, C-1_benzoyl_), 133.2 (1C, C-4_pyridyl_), 141.5 (1C, C-2_thiophenyl_), 147.2 (1C, C-2_pyridyl_), 150.1 (1C, C-6_pyridyl_), 151.6 (1C, C-3_pyrazolyl_), 153.3 (1C, C-5_pyrazolyl_), 169.3 (1C, CON). IR (neat): *ṽ* [cm^−1^] = 3302, 3059, 1685, 1585, 1573, 1512, 1454, 1446, 1419, 1346, 1327, 1226, 1199, 1056, 999, 914, 794, 736, 705, 690, 659. HRMS (APCI): *m*/*z* = 395.0728 calculated for [M + H]^+^, found: 395.0733. HPLC: *t*_R_ = 20.2 min, purity: 93.2%.

*N-[3-(pyridin-3-yl)-1H-pyrazol-5-yl]benzamide* (**26a**). According to general procedure D, aminopyrazole **7a** (56.0 mg, 350 µmol) was acylated using BzCl (40.6 µL, 350 µmol, 1.00 eq.) in dry pyridine/THF (3.00 mL/1.50 mL) and stirred first for 1 h at 0 °C and then for 2 h at rt. Flash column chromatography (CH_2_Cl_2_/CH_3_OH = 95/5) yielded **26a** as a colorless solid (63 mg, 238 µmol, 68%). M.p.: 270 °C (decomp.). TLC: *R*_f_ = 0.15 (CH_2_Cl_2_/CH_3_OH = 95/5). ^1^H NMR (600 MHz, DMSO-*d*_6_): *δ* (ppm) = 7.19 (bs, 0.2H, 4-H_pyrazolyl_*), 7.19 (bs, 0.8H, 4-H_pyrazolyl_), 7.43–7.65 (m, 4H, 3/4/5-H_benzoyl_, 5-H_pyridyl_), 7.99–8.08 (m, 2H, 2/6-H_benzoyl_), 8.13–8.19 (m, 1H, 4-H_pyridyl_), 8.50–8.59 (m, 1H, 6-H_pyridyl_), 8.98–9.05 (m, 1H, 2-H_pyridyl_), 10.91 (bs, 0.8H, CONH), 10.91 (bs, 0.2H, CONH*), 12.86 (bs, 0.2H, NH*), 13.13 (bs, 0.8H, NH). The ratio of tautomers is 8:2, the minor tautomer is marked with an asterisk (*). ^13^C NMR (151 MHz, DMSO-*d*_6_): *δ* (ppm) = 95.5 (1C, C-4_pyrazolyl_), 124.0 (1C, C-5_pyridyl_), 125.4 (1C, C-3_pyridyl_), 127.8 (2C, C-2/6_benzoyl_), 128.3 (2C, C-3/5_benzoyl_), 131.6 (1C, C-4_benzoyl_), 132.3 (1C, C-4_pyridyl_), 134.0 (1C, C-1_benzoyl_), 138.9 (1C, C-3_pyrazolyl_), 146.1 (1C, C-2_pyridyl_), 148.6 (1C, C-5_pyrazolyl_), 149.0 (1C, C-6_pyridyl_), 164.6 (1C, CONH). The signals of the major tautomer are given. IR (neat): *ṽ* [cm^−1^] = 3275, 1651, 1577, 1562, 1543, 1311, 1002, 960, 894, 790, 705, 690, 655. HRMS (APCI): *m*/*z* = 265.1084 calculated for [M + H]^+^, found: 265.1058. HPLC: *t*_R_ = 12.4 min, purity: 99.4%.

*N-(3-phenyl-1H-pyrazol-5-yl)benzamide* (**26b**). According to general procedure D, aminopyrazole **7b** (50.0 mg, 314 µmol) was acylated using BzCl (36.5 µL, 314 µmol, 1.00 eq.) in dry pyridine/THF (3.00 mL/1.50 mL) and stirred first for 1 h at 0 °C and then for 2 h at rt. The solid was filtered off and washed with H_2_O (2×) to yield **26b** as a colorless solid (64 mg, 77%). M.p.: 194–195 °C. TLC: *R*_f_ = 0.13 (CH/EtOAc = 60/40). ^1^H NMR (600 MHz, DMSO-*d*_6_): *δ* (ppm) = 6.55 (bs, 0.1H, 4-H_pyrazolyl_*), 7.07 (bs, 0.9H, 4-H_pyrazolyl_), 7.32–7.39 (m, 1H, 4-H_phenyl_), 7.44–7.49 (m, 2H, 3/5-H_phenyl_), 7.49–7.53 (m, 2H, 3/5-H_benzoyl_), 7.55–7.60 (m, 1H, 4-H_benzoyl_), 7.73–7.83 (m, 2H, 2/6-H_phenyl_), 8.00–8.07 (m, 2H, 2/6-H_benzoyl_), 10.85 (bs, 0.9H, CONH), 11.07 (bs, 0.1H, CONH*), 12.66 (bs, 0.1H, NH*), 12.95 (bs, 0.9H, NH). The ratio of tautomers is 9:1, the minor tautomer is marked with an asterisk (*). ^13^C NMR (151 MHz, DMSO-*d*_6_): *δ* (ppm) = 94.8 (1C, C-4_pyrazolyl_), 125.0 (2C, C-2/6_phenyl_), 127.8 (2C, C-2/6_benzoyl_), 128.1 (1C, C-4_phenyl_), 128.3 (2C, C-3/5_benzoyl_), 129.0 (2C, C-3/5_phenyl_), 129.4 (1C, C-1_phenyl_), 131.6 (1C, C-4_benzoyl_), 134.1 (1C, C-1_benzoyl_), 141.8 (1C, C-3_pyrazolyl_), 148.3 (1C, C-5_pyrazolyl_), 164.6 (1C, CONH). The signals of the major tautomer are given. IR (neat): *ṽ* [cm^−1^] = 3282, 3059, 1654, 1570, 1543, 1489, 1307, 1280, 1002, 894, 794, 759, 675. HRMS (APCI): *m*/*z* = 264.1131 calculated for [M + H]^+^, found: 264.1107. HPLC: *t*_R_ = 17.5 min, purity: 99.6%.

*3-oxo-3-phenylpropanenitrile* (**28b**). Under N_2_, at ™78 °C, *n*-BuLi (4.58 mL, 7.32 mmol, 1.10 eq, 1.6m in *n*-hexane) was added dropwise over 20 min to a solution of CH_3_CN (417 µL, 7.99 mmol, 1.20 eq.) in dry THF (12.0 mL) and the reaction mixture was stirred for 1 h. A solution of ethyl benzoate (952 µL, 6.66 mmol, 1.00 eq.) in dry THF (3.00 mL) was added dropwise to the reaction mixture at ™78 °C and the mixture was allowed to warm up to rt over 16 h. The suspension was quenched with aqueous HCl (4M), the aqueous phase was extracted with EtOAc (3×), the combined organic layers were dried (Na_2_SO_4_), filtered and concentrated in vacuo. The residue was purified by flash column chromatography (CH/EtOAc = 1/0 → 50/50) to yield **28b** as a yellow solid (566 mg, 3.90 mmol, 59%). M.p.: 79–80 °C. TLC: *R*_f_ = 0.37 (CH/EtOAc = 60/40). ^1^H NMR (400 MHz, DMSO-*d*_6_): *δ* (ppm) = 4.76 (s, 2H, CH_2_), 7.53–7.61 (m, 2H, 3/5-H_phenyl_), 7.68–7.73 (m, 1H, 4-H_phenyl_), 7.90–7.97 (m, 2H, 2/6-H_phenyl_). ^13^C NMR (101 MHz, DMSO-*d*_6_): *δ* (ppm) = 30.0 (1C, CH_2_), 115.9 (1C, CN), 128.4 (2C, C-2/6_phenyl_), 128.9 (2C, C-3/5_phenyl_), 134.2 (1C, C-4_phenyl_), 134.6 (1C, C-1_phenyl_), 189.7 (1C, CO). IR (neat): *ṽ* [cm^−1^] = 3360, 3070, 2978, 2954, 2924, 2256, 1685, 1597, 1581, 1450, 1392, 1327, 1215, 999, 925, 752, 682. HRMS (APCI): *m*/*z* = 146.0600 calculated for [M + H]^+^, found: 146.0569. HPLC: *t*_R_ = 14.4 min, purity: 94.6%.

*[5-amino-3-(pyridin-3-yl)-1H-pyrazol-1-yl](phenyl)methanone* (**29a**). According to general procedure B, β-ketonitrile sodium enolate **6a** (200 mg, 1.19 mmol, 1.00 eq.) was dissolved in aqueous HCl (5 mL, 1M), followed by work-up as described above. The orange oily residue of the keto-form of β-ketonitrile (**28a**) and benzhydrazide (113 mg, 833 µmol, 0.70 eq.) were reacted in a solution of methanesulfonic acid (7.7 µL, 103 µmmol, 0.1 eq.) in dry EtOH (2.00 mL). Flash column chromatography (CH_2_Cl_2_/CH_3_OH = 1/0 → 90/10) yielded **29a** as a yellow solid (124 mg, 56%, calculated for 0.70 eq benzhydrazide). TLC: *R*_f_ = 0.25 (CH_2_Cl_2_/CH_3_OH = 95/5). ^1^H NMR (600 MHz, DMSO-*d*_6_): *δ* (ppm) = 5.99 (s, 1H, 4-H_pyrazolyl_), 6.93 (bs, 2H, NH_2_), 7.45 (ddd, *J* = 7.9/4.8/0.9 Hz, 1H, 5-H_pyridyl_), 7.54–7.58 (m, 2H, 3/5-H_benzoyl_), 7.64–7.68 (m, 1H, 4-H_benzoyl_), 8.06–8.09 (m, 2H, 2/6-H_benzoyl_), 8.11 (ddd, *J* = 7.9/2.3/1.7 Hz, 1H, 4-H_pyridyl_), 8.58 (dd, *J* = 4.8/1.7 Hz, 1H, 6-H_pyridyl_), 8.96 (dd, *J* = 2.3/0.9 Hz, 1H, 2-H_pyridyl_). ^13^C NMR (151 MHz, DMSO-*d*_6_): *δ* (ppm) = 84.9 (1C, C-4_pyrazolyl_), 123.8 (1C, C-5_pyridyl_), 127.88 (2C, C-3/5_benzoyl_), 127.91 (1C, C-3_pyridyl_), 130.9 (2C, C-2/6_benzoyl_), 132.4 (1C, C-4_benzoyl_), 133.0 (1C, C-1_benzoyl_), 133.2 (1C, C-4_pyridyl_), 147.2 (1C, C-2_pyridyl_), 149.9 (1C, C-6_pyridyl_), 151.5 (1C, C-3_pyrazolyl_), 153.3 (1C, C-5_pyrazolyl_), 169.5 (1C, CON). IR (neat): *ṽ* [cm^−1^] = 3452, 3275, 3197, 3066, 1681, 1627, 1597, 1581, 1450, 1365, 1334, 1315, 941, 921, 740, 721, 698. HRMS (APCI): *m*/*z* = 265.1084 calculated for [M + H]^+^, found: 265.1068. HPLC: *t*_R_ = 14.2 min, purity: 98.3%.

*(5-amino-3-phenyl-1H-pyrazol-1-yl)(phenyl)methanone* (**29b**). According to general procedure B, β-ketonitrile keto-form **28b** (150 mg, 1.03 mmol, 1.00 eq.) and benzhydrazide (141 mg, 1.03 mmol, 1.00 eq.) were reacted in a solution of methanesulfonic acid (6.7 µL, 103 µmmol, 0.1 eq.) in dry EtOH (2.00 mL). Flash column chromatography (CH/EtOAc = 1/0 → 50/50) yielded **29b** as a yellow solid (264 mg, 97%). TLC: *R*_f_ = 0.56 (CH/EtOAc = 60/40). ^1^H NMR (600 MHz, DMSO-*d*_6_): *δ* (ppm) = 5.90 (s, 1H, 4-H_pyrazolyl_), 6.85 (bs, 2H, NH_2_), 7.36–7.44 (m, 3H, 3/4/5-H_phenyl_), 7.54–7.58 (m, 2H, 3/5-H_benzoyl_), 7.63–7.67 (m, 1H, 4-H_benzoyl_), 7.74–7.77 (m, 2H, 2/6-H_phenyl_), 8.05–8.08 (m, 2H, 2/6-H_benzoyl_). ^13^C NMR (151 MHz, DMSO-*d*_6_): *δ* (ppm) = 84.9 (1C, C-4_pyrazolyl_), 126.0 (2C, C-2/6_phenyl_), 127.8 (2C, C-3/5_benzoyl_), 128.6 (2C, C-3/5_phenyl_), 129.0 (1C, C-4_phenyl_), 130.8 (2C, C-2/6_benzoyl_), 132.1 (1C, C-1_phenyl_), 132.3 (1C, C-4_benzoyl_), 133.2 (1C, C-1_benzoyl_), 153.2 (1C, C-5_pyrazolyl_), 153.9 (1C, C-3_pyrazolyl_), 169.5 (1C, CON). IR (neat): *ṽ* [cm^−1^] = 3406, 3294, 3209, 3116, 1670, 1612, 1577, 1473, 1446, 1361, 1311, 1222, 1188, 1134, 921, 752, 678, 651. HRMS (APCI): *m*/*z* = 264.1131 calculated for [M + H]^+^, found: 264.1150. HPLC: *t*_R_ = 21.1 min, purity: 98.3%.

*2-fluoro-3-oxo-3-phenylpropanenitrile* (**31**). Synthesis was performed as previously reported [29]. Fluoroacetonitrile (300 mg, 5.1 mmol, 1.00 eq.) and Ph_2_P(O)Cl (1.20 g, 5.1 mmol, 1.00 eq.) were dissolved in dry THF (45 mL) in an inert atmosphere. At ™78 °C, LiHMDS (10 mL, 10.2 mmol, 1 M in THF, 2.00 eq.) was slowly added, leading to a red solution. After stirring for 15 min at ™78 °C, benzoyl chloride (786 mg, 5.6 mmol, 1.10 eq.) was added dropwise and the temperature was allowed to rise to room temperature. After 1 h, saturated brine (30 mL) was added to the reaction mixture, and the aqueous layer was extracted with diethyl ether (3 × 30 mL). The combined organic layers were dried with Na_2_SO_4_. After filtration and evaporation of the solvents in vacuo, the crude product was purified by flash chromatography (hexane/EtOAc 7:3, *R*_f_ = 0.30) to yield product **31** as a red oil (144 mg, 0.9 mmol, 17%). ^1^H NMR (400 MHz, CDCl_3_) *δ* (in ppm) = 6.15 (dt, *J* = 46.7, 1.9 Hz, 1H, CHF), 7.57 (t, *J* = 8.0 Hz, 2H, 3-H_phenyl_/5-H_phenyl_), 7.72 (t, *J* = 7.5 Hz, 4-H_phenyl_), 8.00 (d, *J* = 7.5 Hz, 2H, 2-H_phenyl_/6-H_phenyl_). ^1^H NMR spectral data of **31** were in accordance with those reported in the literature [29].

*3-amino-4-fluoro-5-phenyl-1H-pyrazole* (**32**). Synthesis was performed as previously reported [29]. In a 50 mL flask, **31** (100 mg, 613.0 μmol, 1.00 eq.) of was dissolved in isopropanol (2.2 mL). To this solution, hydrazine hydrate (31 mg, 98% in water, 613.0 μmol, 1.00 eq.) was added and the mixture was refluxed for 3 h. After completion of the reaction, the solvents were evaporated in vacuo and the crude oil was purified by flash chromatography (EtOAc, *R*_f_ = 0.33) to yield product **31** as an orange solid (60 mg, 389.0 μmol, 55% yield). ^1^H NMR (400 MHz, CDCl_3_): *δ* (in ppm) = 3.67 (br s, 2H, NH_2_), 7.34–7.38 (m, 1H, 4-H_phenyl_), 7.42–7.47 (m, 2H, 3-H_phenyl_/5-H_phenyl_), 7.58–7.60 (m, 2H, 2-H_phenyl_/6-H_phenyl_), NH invisible. ^19^F NMR (376 MHz, CDCl_3_): *δ* (in ppm) = –186.2 (1F, s, CF). ^13^C NMR (101 MHz, CDCl_3_): *δ* (in ppm) = 125.3 (d, *J* = 3.8 Hz, 2C, C-2_phenyl_/C-6_phenyl_), 127.5 (1C, C-1_phenyl_), 127.9 (d, *J* = 4.3 Hz, 1C, C-5_pyrazolyl_), 128.7 (1C, C-4_phenyl_), 129.2 (2C, C-3_phenyl_/C-5_phenyl_), 135.7 (d, *J_C-F_* = 244.1 Hz, 1C, C-4_pyrazolyl_), C-3_pyrazolyl_ invisible. ^1^H and ^13^C NMR spectral data of **31** were in accordance with those reported in the literature [29]. HRMS (APCI): *m*/*z* = 178.0775 calculated for [M + H]^+^, found: 178.0784.

*N-((5-chlorothiophen-2-yl)methyl)-4-fluoro-5-phenyl-1H-pyrazol-3-amine* (**33**). Under N_2_, **32** (54 mg, 305.0 μmol, 1.00 eq.) was dissolved in dry EtOH (3 mL). To this solution, glacial acetic acid (18 mg, 305.0 μmol, 1.00 eq.) and molecular sieves (3 Å) were added. After cooling the mixture to 0 °C, 5-chlorothiophene-2-carbaldehyde (67 mg, 457.0 μmol, 1.50 eq.) was added dropwise over a period of 10 min, and the solution was stirred at 0 °C for 1 h and for another 23 h, while gradually warming to room temperature. After cooling the solution again to 0 °C, NaBH_4_ (69 mg, 1.8 mmol, 6.00 eq.) was added, and the mixture was stirred for another 24 h, while gradually warming to room temperature. The suspension was cooled to 0 °C and diluted with H_2_O (3 mL) and HCl (1 M, 3 mL, aq.), filtered over Celite^®^ and was washed with H_2_O (10 mL), CH_3_OH (10 mL) and EtOAc (10 mL). The organic layer was washed with HCl (1 M, 3x10 mL, aq.) and subsequent neutralization of the combined aqueous layers were carried out with NaOH (1 M, 30 mL, aq.). After re-extraction of the combined aqueous layers with EtOAc (3x50 mL), the combined organic layers were dried (Na_2_SO_4_) and concentrated under reduced pressure, and the residue was purified by flash column chromatography (CH/EtOAc 1:0 → 0:1) to yield product **33** as a colorless solid (72 mg, 234.0 μmol, 77%). M.p.: 121–122 °C. ^1^H NMR (600 MHz, CDCl_3_): δ (in ppm) = 3.92 (br s, 1H, NHCH_2_); 4.56 (s, 2H, CH_2_); 6.75 (d, *J* = 3.7 Hz, 1H, 3-H_chlorothiophenyl_); 6.80 (d, *J* = 3.8 Hz, 1H, 4-H_chlorothiophenyl_); 7.37 (t, *J* = 7.5 Hz, 1H, 4-H_phenyl_); 7.45 (t, *J* = 7.7 Hz, 2H, 3-H_phenyl_/5-H_phenyl_); 7.56 (t, *J* = 7.0 Hz, 2H, 2-H_phenyl_/6-H_phenyl_); 8.86 (br s, 1H, NH). ^19^F NMR (376 MHz, CDCl_3_): δ (in ppm) = –186.0 (1F, s, CF). ^13^C NMR (151 MHz, CDCl_3_): δ (in ppm) = 43.6 (1C, CH_z_); 124.8 (1C, C-3_chlorothiophenyl_); 125.3 (d, *J_C-F_* = 3.5 Hz, 2C, C-2_phenyl_/C-6_phenyl_); 125.8 (1C, C-4_chlorothiophenyl_); 127.6 (1C, C-1_phenyl_), 128.8 (1C, C-4_phenyl_); 129.2 (1C, C-2_chlorothiophenyl_); 129.3 (2C, C-3_phenyl_/C-5_phenyl_); 133.5 (d, *J_C-F_* = 256.7 Hz, 1C, C-4_pyrazolyl_); 141.9 (1C, C-5_chlorothiophenyl_); 144.6 (1C, C-3_pyrazolyl_); C-5_pyrazolyl_ invisible. IR (neat): *ṽ* [cm^–1^] = 3381, 3177, 2901, 2857, 1560, 1518, 1456, 1443, 1420, 1354, 1236, 1177, 1065, 1003, 959, 800, 762, 719, 685, 662. HRMS (APCI): *m*/*z* = 308.0419 calculated for [M + H]^+^, found: 308.0413.

*1-(5-(((5-chlorothiophen-2-yl)methyl)amino)-4-fluoro-3-phenyl-1H-pyrazol-1-yl)-2,2-dimethylpropan-1-one* (**34a**). According to general procedure D, aminopyrazole **33** (81 mg, 262.0 μmol, 1.00 eq.) was acylated using pivaloyl chloride (38 mg, 314.0 μmol, 1.20 eq.) in dry pyridine/THF (3.0 mL/1.5 mL). Flash column chromatography (CH/EtOAc = 9/1) yielded **34a** as a colorless oil (22 mg, 21%). ^1^H NMR (600 MHz, CDCl_3_): δ (in ppm) = 1.53 (s, 9H, *t*-Bu); 4.63 (ddd, *J* = 6.7, 1.7, 0.9 Hz, 2H, NHC*H*_2_); 6.76 (d, *J* = 3.8 Hz, 1H, 3-H_chlorothiophenyl_); 6.79 (d, *J* = 3.8 Hz, 1H, 4-H_chlorothiophenyl_); 7.14 (t, *J* = 6.7 Hz, 1H, NH); 7.40 (t, *J* = 7.3 Hz, 1H, 4-H_phenyl_); 7.45 (t, *J* = 7.3 Hz, 2H, 3-H_phenyl_/5-H_phenyl_); 7.91 (d, *J* = 7.0 Hz, 2H, 2-H_phenyl_/6-H_phenyl_). ^19^F NMR (376 MHz, CDCl_3_): δ (in ppm) = –184.9 (1F, s, CF). ^13^C NMR (151 MHz, CDCl_3_): δ (in ppm) = 27.9 (3C, C(*C*H_3_)_3_); 42.6 (1C, *C*(CH_3_)_3_); 43.0 (d, *J* = 6.3 Hz, 1C, CH_2_), 125.0 (1C, C-3_chlorothiophenyl_); 126.0 (1C, C-4_chlorothiophenyl_); 126.9 (d, *J* = 4.1 Hz, 2C, C-2_phenyl_/C-6_phenyl_); 128.8 (2C, C-3_phenyl_/C-5_phenyl_); 129.4 (1C, C-4_phenyl_); 129.5 (1C, C-2_chlorothiophenyl_); 130.5 (d, J = 4.0 Hz, 1C, C-1_phenyl_); 132.2 (d, *J_C-F_* = 242.8 Hz, 1C, C-4_pyrazolyl_); 137.0 (d, *J_C-F_* = 17.3 Hz, 1C, C-3_pyrazolyl_); 141.1 (d, *J_C-F_* = 6.9 Hz, 1C, C-5_chlorothiophenyl_), 141.5 (d, *J_C-F_* = 6.9 Hz, 1C, C-5_pyrazolyl_), 181.7 (1C, CO). IR (neat): *ṽ* [cm^–1^] = 3366, 2957, 2930, 2870, 1694, 1632, 1518, 1416, 1395, 1360, 1323, 1234, 1001, 943, 795, 770, 692. HRMS (APCI): *m*/*z* = 392.0994 calculated for [M + H]^+^, found: 392.1027. HPLC: *t*_R_ = 27.3 min, purity: 99.1%.

*(5-(((5-chlorothiophen-2-yl)methyl)amino)-4-fluoro-3-phenyl-1H-pyrazol-1-yl)(phenyl)methanone* (**34b**). According to general procedure D, aminopyrazole **33** (79 mg, 255.0 μmol, 1.00 eq.) was acylated using benzoyl chloride (43 mg, 306.0 μmol, 1.20 eq.) in dry pyridine/THF (3.0 mL/1.5 mL). Flash column chromatography (CH/EtOAc = 9/1) yielded **34b** as a yellowish oil (36 mg, 34%). ^1^H NMR (600 MHz, CDCl_3_): δ (in ppm) = 4.71 (d, *J* = 6.8 Hz, 2H, NHC*H_2_*); 6.78 (d, *J* = 3.7 Hz, 1H, 3-H_chlorothiophenyl_); 6.84 (d, *J* = 3.8 Hz, 1H, 4-H_chlorothiophenyl_); 7.16 (t, *J* = 6.7 Hz, 1H, NH); 7.45–7.38 (m, 3H, 3-H_phenyl_/4-H_phenyl_/5-H_phenyl_); 7.52–7.47 (m, 2H, 3-H_benzoyl_/5-H_benzoyl_); 7.60 (ddt, *J* = 8.8, 7.0, 1.3 Hz, 1H, 4-H_benzoyl_); 7.87 (ddt, *J* = 6.9, 2.3, 1.1 Hz, 2H, 2-H_phenyl_/6-H_phenyl_); 8.11 (dd, *J* = 8.4, 1.4 Hz, 2H, 2-H_benzoyl_/6-H_benzoyl_). ^19^F NMR (376 MHz, CDCl_3_): δ (in ppm) = –182.9 (1F, s, CF). ^13^C NMR (151 MHz, CDCl_3_): δ (in ppm) = 43.1 (d, *J* = 5.8 Hz, 1C, CH_2_); 125.1 (1C, C-3_chlorothiophenyl_); 126.1 (1C, C-4_chlorothiophenyl_); 127.1 (d, *J* = 3.9 Hz, 2C, C-2_phenyl_/C-6_phenyl_); 128.0 (2C, C-3_benzoyl_/C-5_benzoyl_); 128.8 (2C, C-3_phenyl_/C-5_phenyl_); 129.6 (1C, C-4_phenyl_); 129.7 (1C, C-2_chlorothiophenyl_); 130.1 (d, *J_C-F_* = 3.9 Hz, 1C, C-1_phenyl_); 131.7 (2C, C-2_benzoyl_/C-6_benzoyl_); 132.3 (1C, C-1_benzoyl_); 132.8 (d, *J_C-F_* = 244.5 Hz, 1C, C-4_pyrazolyl_); 132.9 (1C, C-4_benzoyl_); 137.32 (d, *J_C-F_* = 17.9 Hz, 1C, C-3_pyrazolyl_); 141.0 (1C, C-5_chlorothiophenyl_); 143.3 (d, *J_C-F_* = 7.0 Hz, 1C, C-5_pyrazolyl_); 171.0 (1C, CO). IR (neat): *ṽ* [cm^–1^] = 3368, 3061, 2930, 2857, 1682, 1630, 1580, 1518, 1462, 1414, 1348, 1202, 1001, 920, 795, 772, 710, 691. HRMS (APCI): *m*/*z* = 412.0681 calculated for [M + H]^+^, found: 412.0715. HPLC: *t*_R_ = 25.7 min, purity: 98.3%.

**X-ray diffraction**. Data sets for compounds **10a** and **19** were collected with a Bruker D8 Venture Photon III Diffractometer. Programs used: data collection: *APEX4* Version 2021.4-0 [39]; cell refinement: *SAINT* Version 8.40B [39]; data reduction: *SAINT* Version 8.40B [39]; absorption correction, *SADABS* Version 2016/2 [39]; structure solution *SHELXT-*Version 2018-3 [40]; structure refinement *SHELXL-*Version 2018-3 [41] and graphics, *XP* [42]. R-values are given for observed reflections, and wR^2^ values are given for all reflections. CCDC-2211140 (**10a**) and CCDC-2211141 (**19**) contain the supplementary crystallographic data for this paper. These data can be obtained free of charge from The Cambridge Crystallographic Data Centre via www.ccdc.cam.ac.uk/data_request/cif, accessed on 14 October 2022. X-ray crystal structure analysis of **10a** and **19** is given in the Supporting Information.

**Serine protease inhibition assays**. The inhibitory activity of synthesized compounds towards thrombin, FXIIa, FXa, FXIa, plasmin, plasma kallikrein, chymotrypsin, and trypsin was measured by quantifying the hydrolysis rate of the fluorogenic substrate as reported previously [14,17]. Briefly, the activity was tested in buffer (10 mM Tris-Cl, 150 mM NaCl, 10 mM MgCl_2_·6H_2_O, 1 mM CaCl_2_·2H_2_O, 0.1% *w*/*v* BSA, 0.01% *v*/*v* Triton-X100, pH = 7.4) utilizing clear flat-bottom, black polystyrene 96 well-plates. The enzymes (human β-FXIIa, HFXIIAB, >95% purity; Molecular Innovations, 2.5 nM–final concentration; human α-thrombin (active) protein, ABIN2127880, >95% purity; antibodies-online, 0.25 nM–final concentration; human Factor Xa, HFXA, >95% purity; Molecular Innovations, 2.5 nM–final concentration; human Factor XIa, HFXIA, >95% purity; Molecular Innovations, 0.5 nM–final concentration; human plasmin, >95% purity; Innovative Research, 10 nM–final concentration; human plasma kallikrein, >94% purity; Innovative Research, 0.25 nM–final concentration; porcine trypsin; Merck, 3.5 nM–final concentration; human chymotrypsin; >95% purity; Innovative Research, 0.5 nM–final concentration) and the fluorogenic substrates for thrombin: Boc-Val-Pro-Arg-AMC (Pepta Nova, 25 µM–final concentration, *K*_m_ = 18 µM); for FXIIa: Boc-Gln-Gly-Arg-AMC (Pepta Nova, 25 µM–final concentration, *K*_m_ = 167 µM); for FXa: Boc-Ile-Glu-Gly-Arg-AMC (Pepta Nova, 25 µM–final concentration); for FXIa: Boc-Glu(OBzl)-Ala-Arg-AMC (Pepta Nova, 25 µM–final concentration); for plasmin: Boc-Val-Leu-Lys-AMC (Pepta Nova, 25 µM–final concentration); for plasma kallikrein: Z-Phe-Lys-AMC (Pepta Nova, 25 µM–final concentration); for chymotrypsin: Suc-Ala-Ala-Pro-Phe-AMC (Sigma-Aldrich, 25 µM–final concentration); for trypsin: Z-Gly-Gly-Arg-AMC (Sigma-Aldrich, 25 µM–final concentration). Dilutions of test-compounds ranging from 2 nM to 32 µM (final concentrations) in DMSO were prepared. The fluorogenic substrate solution was added into the wells followed by the addition of 2 µL of test-compounds solution, and the reaction was triggered by addition of the enzyme solution (final testing volume–152 µL). In case of blank (substrate + buffer) and control (substrate + enzyme) wells, 2 µL of DMSO was added instead of the test-compounds’ solution. Fluorescence intensity was measured with Microplate Reader Mithras LB 940 (Berthold Technologies, excitation at 355 nm, emission at 460 nm) for a period of 1 h (thrombin, FXIIa, FXa, FXIa, and trypsin), 30 min (plasma kallikrein, chymotrypsin), or 15 min (plasmin) with a read every minute. The reactions were performed at 25 °C. To derive IC_50_ values, endpoint RFU (single fluorescence reading) was used. Sigmoidal curves were prepared in GraphPad Prism software and IC_50_ values were derived from the fitted curves [14,17].

**In vitro plasma coagulation assays (aPTT and PT)**. All measurements were performed using commercially available citrated (3.8%) human pooled plasma (Dunn Labortechnik GmbH, Germany) on a semi-automated coagulometer (Thrombotimer-2, Behnk Elektronik, Germany) according to the manufacturer instructions as previously reported [14,17]. For aPTT measurement, plasma (100 μL) was placed into the incubation cuvettes of the instrument and incubated for 2 min at 37 °C. Then, test compound solution (10 μL) or solvent (DMSO, 10 μL) was added with a pipette. After 1 min of incubation, 100 μL of prewarmed (37 °C) aPTT reagent (Convergent Technologies, Germany) was added and incubated for additional 2 min. The cuvettes were transferred to a measuring position, the coagulation was initiated by addition of 100 μL of CaCl_2_ solution (25 mM, prewarmed at 37 °C, Convergent Technologies, Germany) and the clotting time was recorded. For PT assays, plasma (100 μL) was incubated for 2 min at 37 °C. Then, test compound solution (10 μL) or solvent (DMSO, 10 μL) was added with a pipette. After 3 min of incubation, the cuvettes were transferred to a measuring position, the coagulation was initiated by addition of 100 μL of PT assay reagent already containing CaCl_2_ (prewarmed at 37 °C, Biolabo, France) and the clotting time was recorded [14,17].

**Analysis of the covalent thrombin-inhibitor complex by LC/ESI-MS.** The analysis of covalent thrombin-inhibitor complex was performed utilizing LC/ESI-MS as reported previously [14,17] with variations. Briefly, 2 µL of the stock solution (128 µM) of human α-thrombin (active) protein (ABIN2127880, >95% purity; antibodies-online) were diluted with 68 µL of purified water. An aliquot of 35 µL was mixed with 3 µL of inhibitor **24e** solution (1 mM in DMSO) to prepare the enzyme-inhibitor solution, which was then analyzed via LC/ESI-MS. Reversed phase liquid chromatography was performed using a Discovery BIO Wide Pore C5 column (100 × 2.1 mm, 3 µm particle size, 300 Å pore size) from Supelco (Bellefonte, PA, USA). For chromatographic analysis of the sample solutions, 0.1% formic acid in purified water (A) and 0.1% formic acid in acetonitrile (B) were used as mobile phase. The following gradient of 15 min in total was applied: starting with 5% B, B is increased up to 40% within 3 min. After holding 40% B for 1.5 min, B is decreased to 5% within 0.5 min and kept at 5% for 1 min. B is then increased to 95% within 4 min and kept at this value for 1 min. After decreasing B to 5% within 1 min, this ratio is kept for 3 min. The flow rate was 0.4 mL/min, the column oven temperature was 30 °C and the injection volume was set to 5 μL. Mass spectrometric detection was carried out using a timsTOF time-of-flight mass spectrometer from Bruker Daltonics (Bremen, Germany) equipped with an electrospray ionization interface. The enzyme-inhibitor complex was analyzed in the positive ion mode using the following ESI-MS parameters: The mass range was set to *m*/*z* 1000–4000, nebulizer gas (nitrogen) was 1.6 bar, dry gas (nitrogen) was 9.0 L/min, dry gas temperature was 200 °C, Funnel 1 RF was 450 V, pre pulse storage time was 30 µs and transfer time was 100 µs. Deconvoluted mass spectra were obtained by averaging frames from 4.0 min to 4.6 min retention time and subsequent deconvolution using multiple charge states of the respective protein species.

**Molecular Modeling**. To perform molecular docking studies, the crystal structure of human thrombin (PDB ID: 6CYM [16]) was used as a protein model. The protein was prepared using the LigX module of MOE (version 2019.01; Chemical Computing Group, Montreal, Canada) according to the following procedure. All solvent particles were removed, hydrogen atoms were automatically added, and tautomeric forms and protonation states of the amino acid residues were automatically assigned, using the “Protonate 3D” module at pH 7.0, and model was subjected to restrained energy minimization as reported before [43]. Energy minimized structures of the ligands were generated in MOE modeling software using the MMFF94x force field within the RMS gradient of 0.1 kcal/mol/Å. Covalent docking with pharmacophore-based constrains was carried out using the MOE Covalent Docking procedure (“Rigid Receptor”) with a protocol defined by a chemical reaction in which the hydroxy group of a Ser residue undergoes a nucleophilic addition to a carbonyl carbon atom. The active serine residues (Ser219 in structure 6CYM [16]) was selected as an attachment point.

## Figures and Tables

**Table 1:**
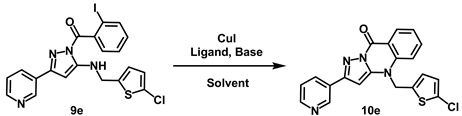
Optimization of the model reaction ^a^.

No.	Base	Ligand	Solvent	T [°C]	Time	Yield [%] ^b^
1	Cs_2_CO_3_	thiophene-2-carboxylic acid	DMF	r.t.	96 h	40
2	Cs_2_CO_3_	1,10-phenanthroline	DMSO	r.t.	72 h	44
3	Cs_2_CO_3_	1,10-phenanthroline	DMF	60	4 h	63
4	Cs_2_CO_3_	1,10-phenanthroline	DMF	80	3 h	90 ^c^
5	Cs_2_CO_3_	1,10-phenanthroline	DMSO	80	30 min	74
6	K_3_PO_4_	1,10-phenanthroline	DMF	80	1 h	90
7	K_2_CO_3_	1,10-phenanthroline	DMF	80	1 h	66
8	-	1,10-phenanthroline	DMF	80	2 h	-
9	NEt_3_	1,10-phenanthroline	DMF	80	3.5 h	-
10	Cs_2_CO_3_	-	DMF	80	75 min	46
11	Cs_2_CO_3_	thiophene-2-carboxylic acid	DMF	80	45 min	55
12	Cs_2_CO_3_	L-proline	DMF	80	90 min	75
13	Cs_2_CO_3_	1,10-phenanthroline	DMF	100	15 min	73
14	K_3_PO_4_	1,10-phenanthroline	DMF	100	15 min	77

^a^—Reaction conditions: 50 mg of **9e** (1 eq.), CuI (0.2 eq.), base (2 eq.), ligand (0.2 eq.), solvent (2.6 mL); ^b^—Isolated yield; ^c^—According to the TLC analysis, full consumption of the starting material was observed already after 30 min.

**Table 2:** Thrombin and FXIIa Inhibition by Acylated Aminopyrazoles **9a**–**i** and **14**.

Code	Structure	Serine Protease IC_50_ ± SD (nM) ^a^		Code	Structure	Serine Protease IC_50_ ± SD (nM) ^a^	
		Thrombin	FXIIa			Thrombin	FXIIa
**9a**	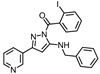	>5000	>5000	**9g**	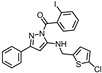	1308 ± 32	>5000
**9b**	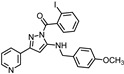	>5000	>5000	**9h**	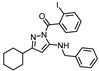	>5000	>5000
**9c**	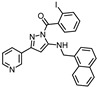	>5000	>5000	**9i**	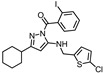	>5000	>5000
**9d**	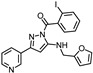	>5000	>5000	**14**	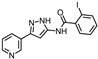	>5000	>5000
**9e**	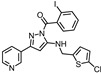	165 ± 5	>5000	**Dabigatran**		6.4 ± 0.4	>5000
**9f**	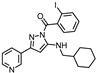	>5000	>5000				

^a^—measurements were performed in triplicate; the substrate concentration [S]_0_ = 25 µM; measured FXIIa *K*_m_ = 167 ± 4 µM for Boc-Gln-Gly-Arg-AMC substrate; measured thrombin *K*_m_ = 18 ± 1 µM for Boc-Val-Pro-Arg-AMC substrate. The *K_i_*-values could not be directly obtained from the Cheng–Prusoff equation in this case due to the enzyme-inhibitor covalent interaction [17].

**Table 3:** Serine Protease Inhibition by Acylated Aminopyrazoles **24b**–**e**,**g**–**i**, **25**, **29a**,**b**, and **34a**,**b**.

Code	Structure	Serine Protease IC_50_ ± SD (nM) ^a^		Code	Structure	Serine Protease IC_50_ ± SD (nM) ^a^	
		Thrombin	FXIIa			Thrombin	FXIIa
**24b**	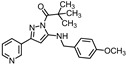	1282 ± 9	>5000	**24i**	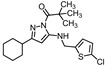	>5000	>5000
**24c**	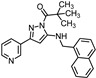	>5000	>5000	**25**	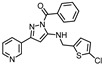	18 ± 1	>5000
**24d**	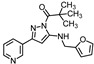	1307 ± 32	>5000	**29a**	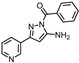	>5000	>5000
**24e**	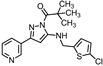	16 ± 1	>5000	**29b**	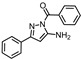	>5000	>5000
**24g**	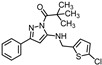	419 ± 99	>5000	**34a**	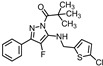	80 ± 2	>5000
**24h**	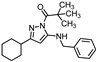	>5000	>5000	**34b**	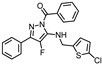	71 ± 3	>5000
**Dabigatran**		6.4 ± 0.4	>5000				

^a^—measurements were performed in triplicate; the substrate concentration [S]_0_ = 25 µM; measured FXIIa *K*_m_ = 167 ± 4 µM for Boc-Gln-Gly-Arg-AMC substrate; measured thrombin *K*_m_ = 18 ± 1 µM for Boc-Val-Pro-Arg-AMC substrate. The *K_i_*-values could not be directly obtained from the Cheng–Prusoff equation in this case due to the enzyme-inhibitor covalent interaction [17].

**Table 4:** Selectivity Profile of Acylated 1*H*-pyrazol-5-amines **24e**, **25**, **34a**, and **34b**.

Serine Protease IC_50_ ± SD (nM) ^a^	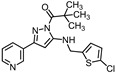 24e	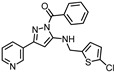 25	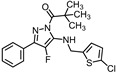 34a	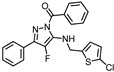 34b
Thrombin	16 ± 1	18 ± 1	80 ± 2	71 ± 3
FXIIa	>5000	>5000	>5000	>5000
FXIa	>5000	1018 ± 90	>5000	>5000
FXa	>5000	~5000 ^b^	>5000	>5000
Plasmin	>5000	>5000	>5000	>5000
PK	>5000	639 ± 156	>5000	>5000
Trypsin	>5000	>5000	>5000	>5000
Chymotrypsin	875 ± 59	242 ± 13	>5000	1151 ± 110

^a^—Screened at 5 μM in at least two independent determinations; for active compounds, IC_50_ measurements were performed in triplicate, the substrate concentration [S]_0_ = 25 µM; measured FXIIa *K*_m_ = 167 ± 4 µM for Boc-Gln-Gly-Arg-AMC substrate; measured thrombin *K*_m_ = 18 ± 1 µM for Boc-Val-Pro-Arg-AMC substrate. The *K_i_*-values could not be directly obtained from the Cheng–Prusoff equation in this case due to the enzyme-inhibitor covalent interaction [17]; ^b^ 54% of FXa inhibition at 5 μM.

## Data Availability

X-ray crystal structure of **10a** (CCDC-2211140) and **19** (CCDC-2211141) can be obtained free of charge from the Cambridge Crystallographic Data Centre via www.ccdc.cam.ac.uk/data_request/cif, accessed on 24 October 2022.

## Supplementary Materials

The following supporting information can be downloaded at: https://www.mdpi.com/article/10.3390/ph15111340/s1, ^1^H and ^13^C NMR spectra for all synthesized compounds. X-ray crystal structure analysis of **10a** and **19**.
