# Supplementary material for: Pyrazole-Based Thrombin Inhibitors with a Serine-Trapping Mechanism of Action: Synthesis and Biological Activity
Source: Pharmaceuticals (Basel). 2022 Oct 28;15(11):1340. doi: 10.3390/ph15111340 (PMC9696832; doi:10.3390/ph15111340)
Supplement: Supplementary file 1 [file pharmaceuticals-15-01340-s001.zip › pharmaceuticals-1984583-supplementary.pdf]

Supporting Information

Pyrazole-Based Thrombin Inhibitors with a Serine-Trapping Mechanism of Action: Synthesis and Biological Activity

Calvin Dunker [1], Lukas Imberg [1], Alena I. Siutkina [1], Catharina Erbacher [2], Constantin G. Daniliuc [3], Uwe Karst [2], and Dmitrii V. Kalinin [1,]*

[1]Institute of Pharmaceutical and Medicinal Chemistry, University of Münster, 48149 Münster, Germany

[2]Institute of Inorganic and Analytical Chemistry, University of Münster, 48149 Münster, Germany

[3]Institute for Organic Chemistry, University of Münster, 48149 Münster, Germany

*To whom correspondence should be addressed. Tel.: +49-251-8333372;

E-mail: dmitrii.kalinin@uni-muenster.de (Dmitrii V. Kalinin)

Page

Contents

X-ray crystal structure analysis of **10a** and **19**	S3
Thrombin and FXIIa Inhibition by compounds **10a-i**, **15**, and **19-23**	S6
NMR spectral data	S7
References	S72

X-RAY CRYSTAL STRUCTURE ANALYSIS OF 10A AND 19.

X-Ray diffraction: Data sets for compounds **10a** and **19** were collected with a Bruker D8 Venture Photon III Diffractometer. Programs used: data collection: *APEX4* Version 2021.4-0 [1] (Bruker AXS Inc., **2021**); cell refinement: *SAINT* Version 8.40B (Bruker AXS Inc., **2021**); data reduction: *SAINT* Version 8.40B (Bruker AXS Inc., **2021**); absorption correction, *SADABS* Version 2016/2 (Bruker AXS Inc., **2021**); structure solution *SHELXT*-Version 2018-3 [2] (Sheldrick, G. M. *Acta Cryst.,* **2015**, *A71*, 3-8); structure refinement *SHELXL*-Version 2018-3 [3] (Sheldrick, G. M. *Acta Cryst.,* **2015**, *C71* (1), 3-8) and graphics, *XP* [4] (Version 5.1, Bruker AXS Inc., Madison, Wisconsin, USA, **1998**). *R*-values are given for observed reflections, and wR^2 values are given for all reflections.

X-ray crystal structure analysis of 10a (dan10208): A colorless, plate-like specimen of $C_{22}H_{16}N_4O$, approximate dimensions 0.031 mm x 0.095 mm x 0.131 mm, was used for the X-ray crystallographic analysis. The X-ray intensity data were measured on a single crystal diffractometer Bruker D8 Venture Photon III system equipped with a micro focus tube Cu ImS (CuKα, λ = 1.54178 Å) and a MX mirror monochromator. A total of 1257 frames were collected. The total exposure time was 19.22 hours. The frames were integrated with the Bruker SAINT software package using a wide-frame algorithm. The integration of the data using a monoclinic unit cell yielded a total of 31087 reflections to a maximum θ angle of 66.66° (0.84 Å resolution), of which 2940 were independent (average redundancy 10.574, completeness = 99.8%, R_{int} = 19.19%, R_{sig} = 6.62%) and 2066 (70.27%) were greater than $2\sigma(F^2)$. The final cell constants of a = 9.1968(3) Å, b = 17.2175(5) Å, c = 10.6021(3) Å, β = 97.421(2)°, volume = 1664.74(9) $Å^3$, are based upon the refinement of the XYZ-centroids of 3533 reflections above 20 σ(I) with 9.698° < 2θ < 133.2°. Data were corrected for absorption effects using the Multi-Scan method (SADABS). The ratio of minimum to maximum apparent transmission was 0.872. The calculated minimum and maximum transmission coefficients (based on crystal size) are 0.9120 and 0.9780. The structure was solved and refined using the Bruker SHELXTL Software Package, using the space group $P2_1/c$, with Z = 4 for the formula unit, $C_{22}H_{16}N_4O$. The final anisotropic full-matrix least-squares refinement on F^2 with 245 variables converged at R1 = 5.16%, for the observed data and wR2 = 13.38% for all data. The goodness-of-fit was 1.045. The largest peak in the final difference electron density synthesis was 0.313 $e^-/Å^3$ and the largest hole was -0.294 $e^-/Å^3$ with an RMS deviation of 0.083 $e^-/Å^3$. On the basis of the final model, the calculated density was 1.406 g/cm^3 and F(000), 736 e^-. CCDC Nr.: 2211140.

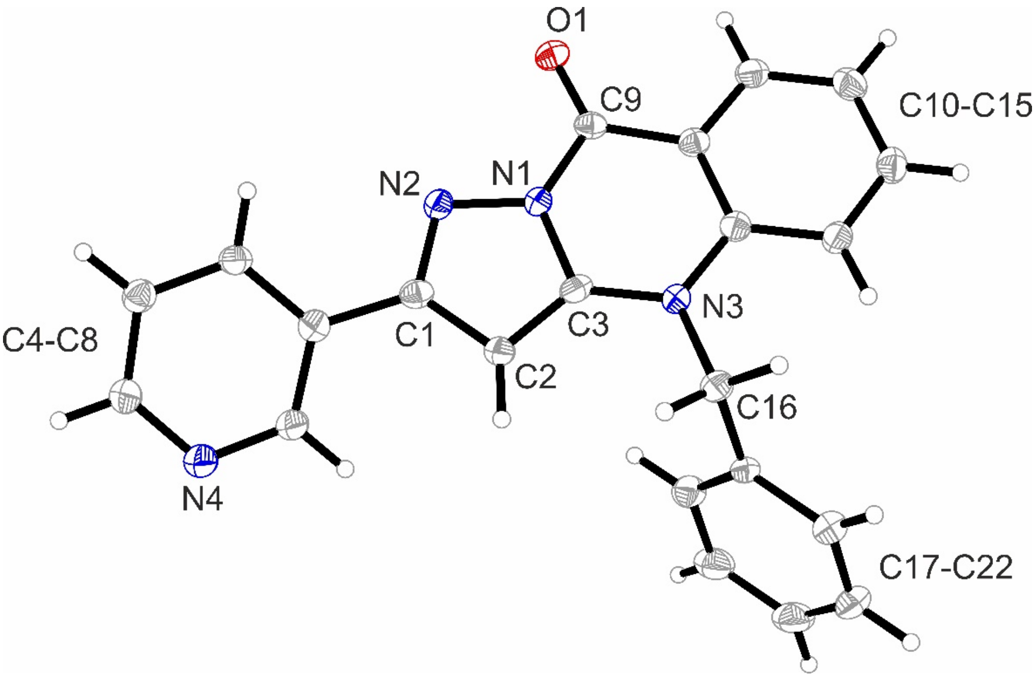

Figure S1: Crystal structure of compound **10a**.
Thermal ellipsoids are shown at 50% probability.

X-ray crystal structure analysis of 19 (dan10272): A colorless, plate-like specimen of $C_{18}H_{11}ClN_6OS$, approximate dimensions 0.047 mm x 0.114 mm x 0.138 mm, was used for the X-ray crystallographic analysis. The X-ray intensity data were measured on a single crystal diffractometer Bruker D8 Venture Photon III system equipped with a micro focus tube Mo ImS (MoKα, λ = 0.71073 Å) and a MX mirror monochromator. A total of 564 frames were collected. The total exposure time was 3.13 hours. The frames were integrated with the Bruker SAINT software package using a narrow-frame algorithm. The integration of the data using a triclinic unit cell yielded a total of 18462 reflections to a maximum θ angle of 27.51° (0.77 Å resolution), of which 3792 were independent (average redundancy 4.869, completeness = 99.2%, R_{int} = 5.03%, R_{sig} = 3.61%) and 3233 (85.26%) were greater than $2\sigma(F^2)$. The final cell constants of a = 8.7008(3) Å, b = 8.8719(2) Å, c = 11.8236(4) Å, α = 68.5590(10)°, β = 78.4850(10)°, γ = 80.8570(10)°, volume = 828.73(4) $Å^3$, are based upon the refinement of the XYZ-centroids of 4692 reflections above 20 σ(I) with 4.799° < 2θ < 54.91°. Data were corrected for absorption effects using the Multi-Scan method (SADABS). The ratio of minimum to maximum apparent transmission was 0.939. The calculated minimum and maximum transmission coefficients (based on crystal size) are 0.9490 and 0.9820. The structure was solved and refined using the Bruker SHELXTL Software Package, using the space group *P*-1, with Z = 2 for the formula unit, $C_{18}H_{11}ClN_6OS$. The final anisotropic full-matrix least-squares refinement on F^2 with 244 variables converged at R1 = 3.33%, for the observed data and wR2 = 8.38% for all data. The goodness-of-fit was 1.070. The largest peak in the final difference electron density synthesis was 0.355 $e^-/Å^3$ and the largest hole was -0.215 $e^-/Å^3$ with an RMS deviation of 0.054 $e^-/Å^3$. On the basis of the final model, the calculated density was 1.582 g/cm^3 and F(000), 404 e^-. CCDC Nr.: 2211141.

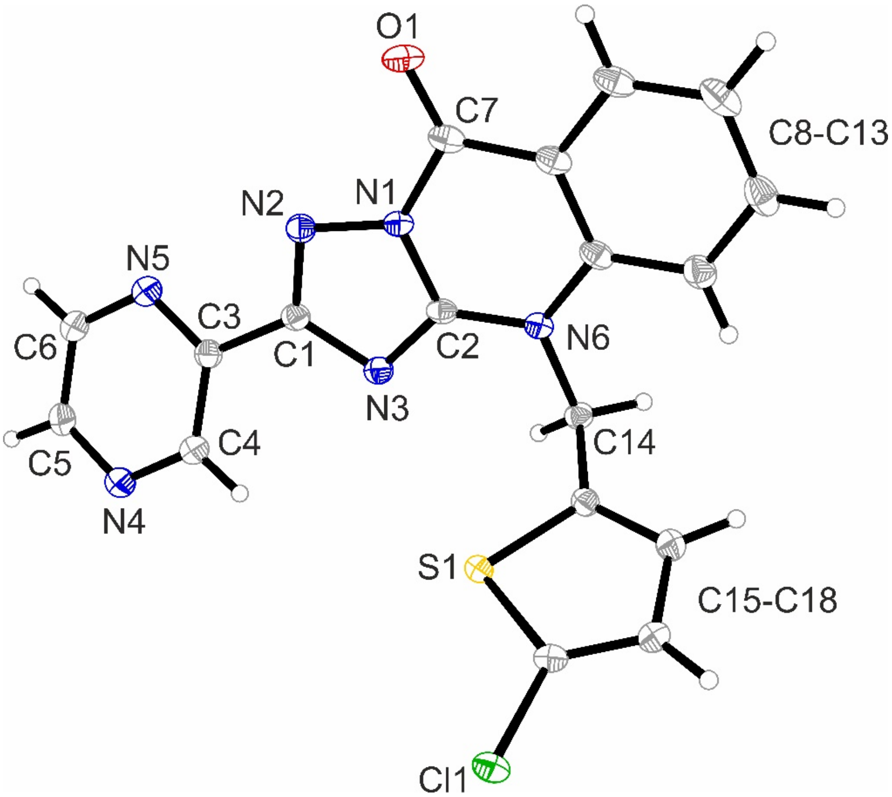

Figure S2: Crystal structure of compound **19**.
Thermal ellipsoids are shown at 50% probability.

Table S1. Thrombin and FXIIa Inhibition by compounds **10a-i**, **15**, and **19-23**

Code	Structure	Serine Protease IC_{50} ± SD (nM)[a]		Code	Structure	Serine Protease IC_{50} ± SD (nM)[a]	
		Thrombin	FXIIa			Thrombin	FXIIa
**10a**	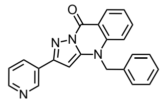	>5000	>5000	**10i**	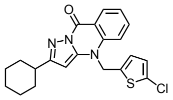	>5000	>5000
**10b**	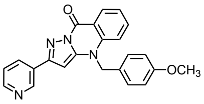	>5000	>5000	**15**	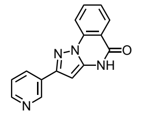	>5000	>5000
**10c**	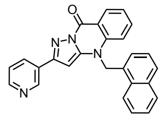	>5000	>5000	**19**	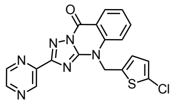	>5000	>5000
**10d**	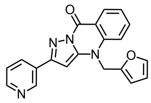	>5000	>5000	**20**	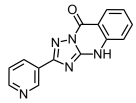	>5000	>5000
**10e**	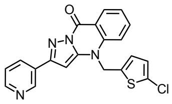	>5000	>5000	**21**	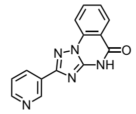	>5000	>5000
**10f**	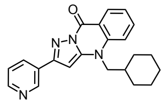	>5000	>5000	**22**	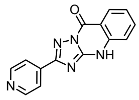	>5000	–
**10g**	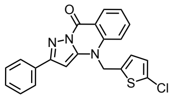	>5000	>5000	**23**	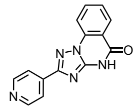	>5000	>5000
**10h**	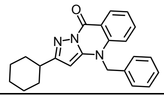	>5000	>5000	**Dabigatran**		6.4 ± 0.4	>5000

[a] – measurements were performed in triplicate; the substrate concentration $[S]_0$ = 25 μM; measured FXIIa K_m = 167 ± 4 μM for Boc-Gln-Gly-Arg-AMC substrate; measured thrombin K_m = 18 ± 1 μM for Boc-Val-Pro-Arg-AMC substrate. The K_i-values couldn't be directly obtained from the Cheng-Prusoff equation in this case due to the enzyme-inhibitor covalent interaction;

NMR SPECTRAL DATA

sodium 2-cyano-1-(pyridin-3-yl)ethen-1-olate (6a)

^{1}H NMR (600 MHz, DMSO-d_6)

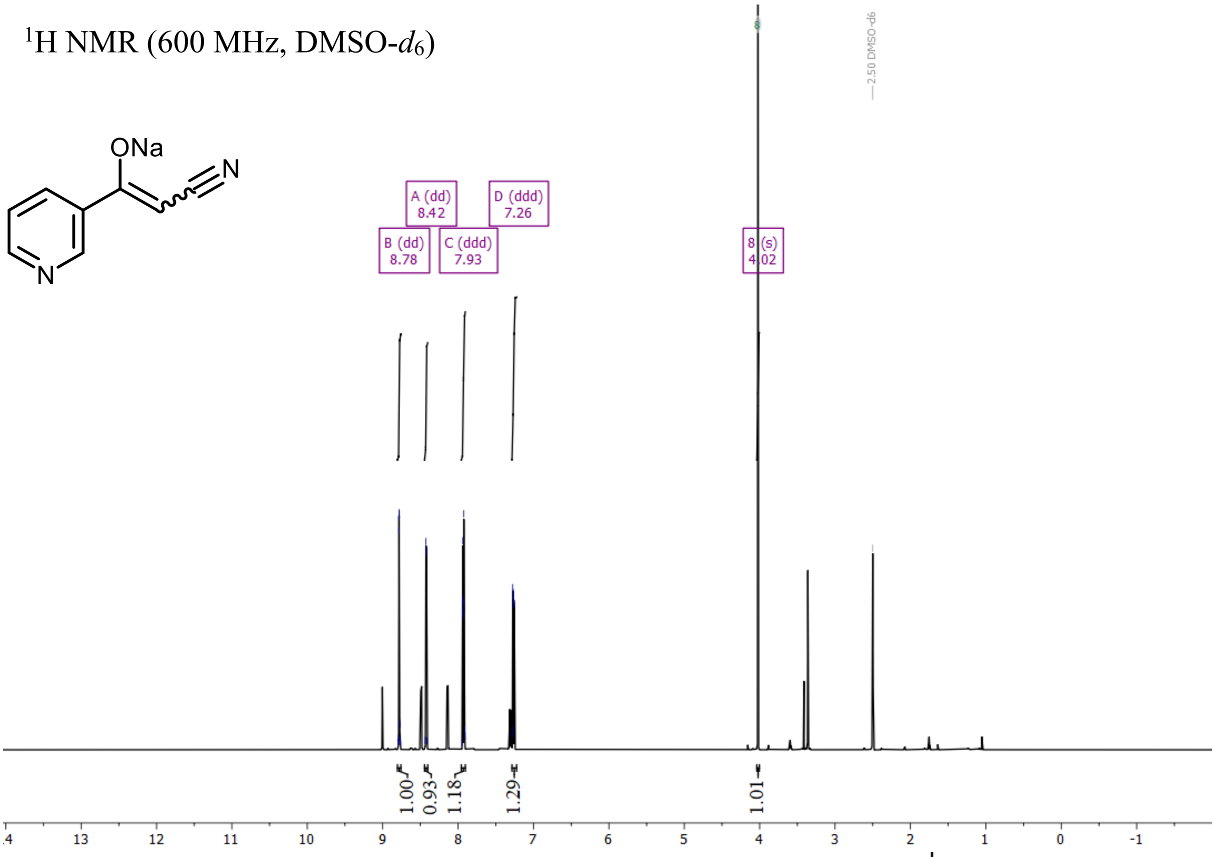

^{13}C NMR (151 MHz, DMSO-d_6)

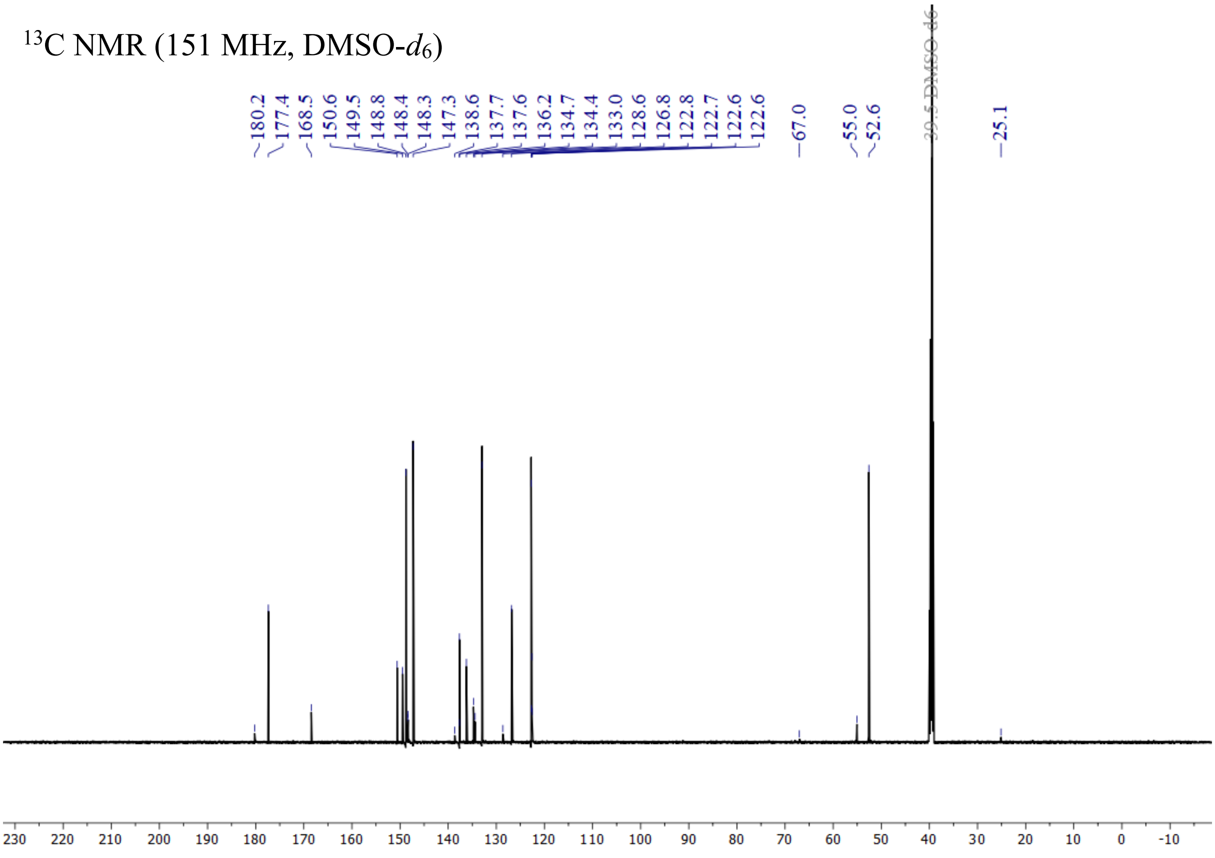

The signals of the major isomer are given.

sodium 2-cyano-1-phenylethen-1-olate (6b)

^{1}H NMR (600 MHz, DMSO-d_6)

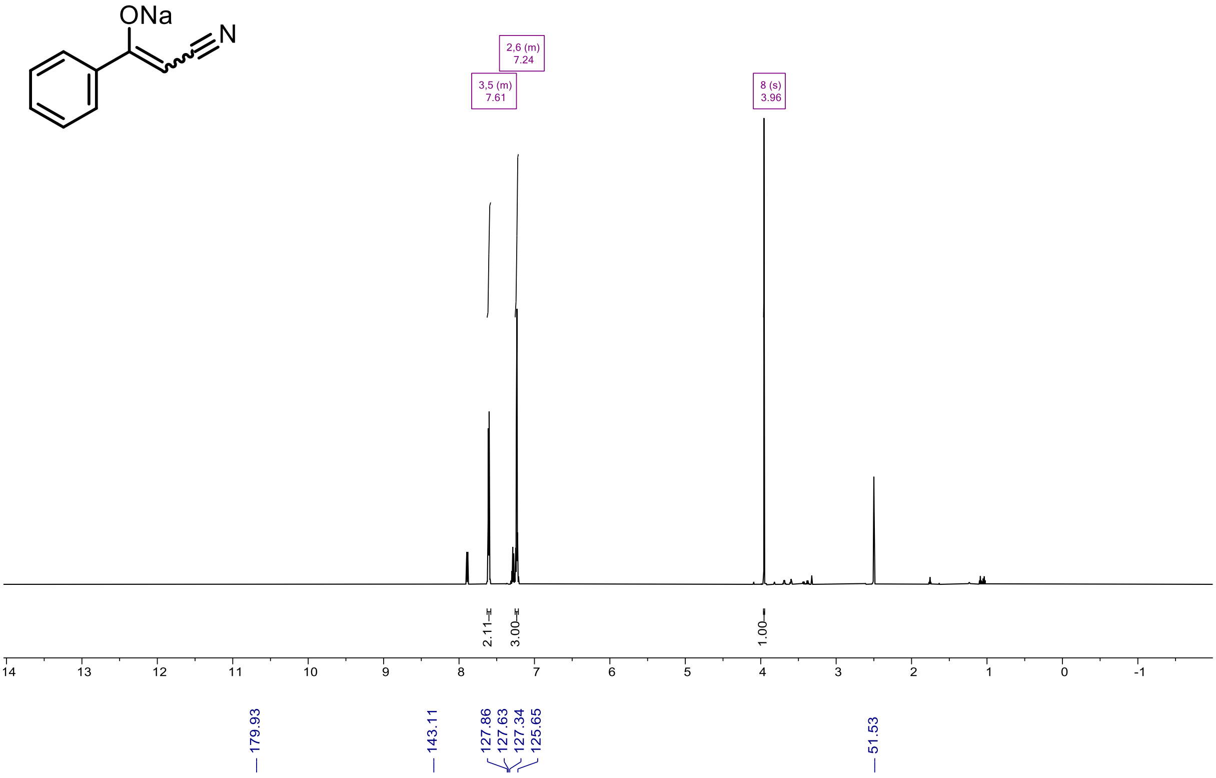

^{13}C NMR (151 MHz, DMSO-d_6)

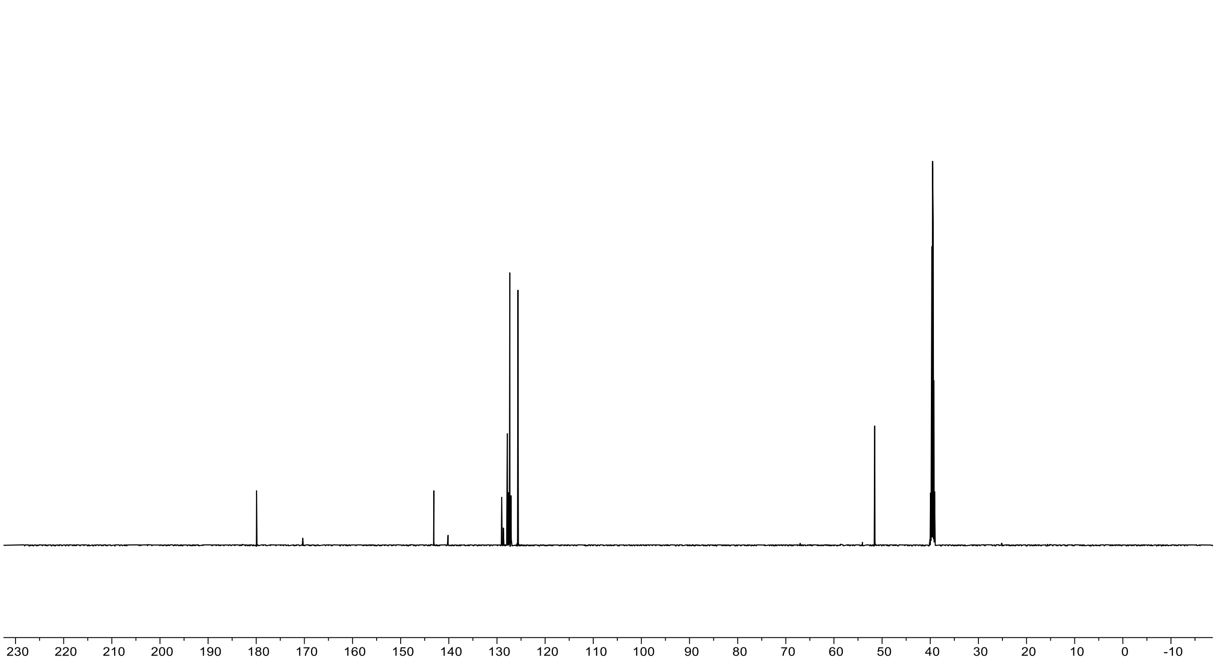

The signals of the major isomer are given.

sodium 2-cyano-1-cyclohexylethen-1-olate (6c)

^{1}H NMR (600 MHz, DMSO-d_6)

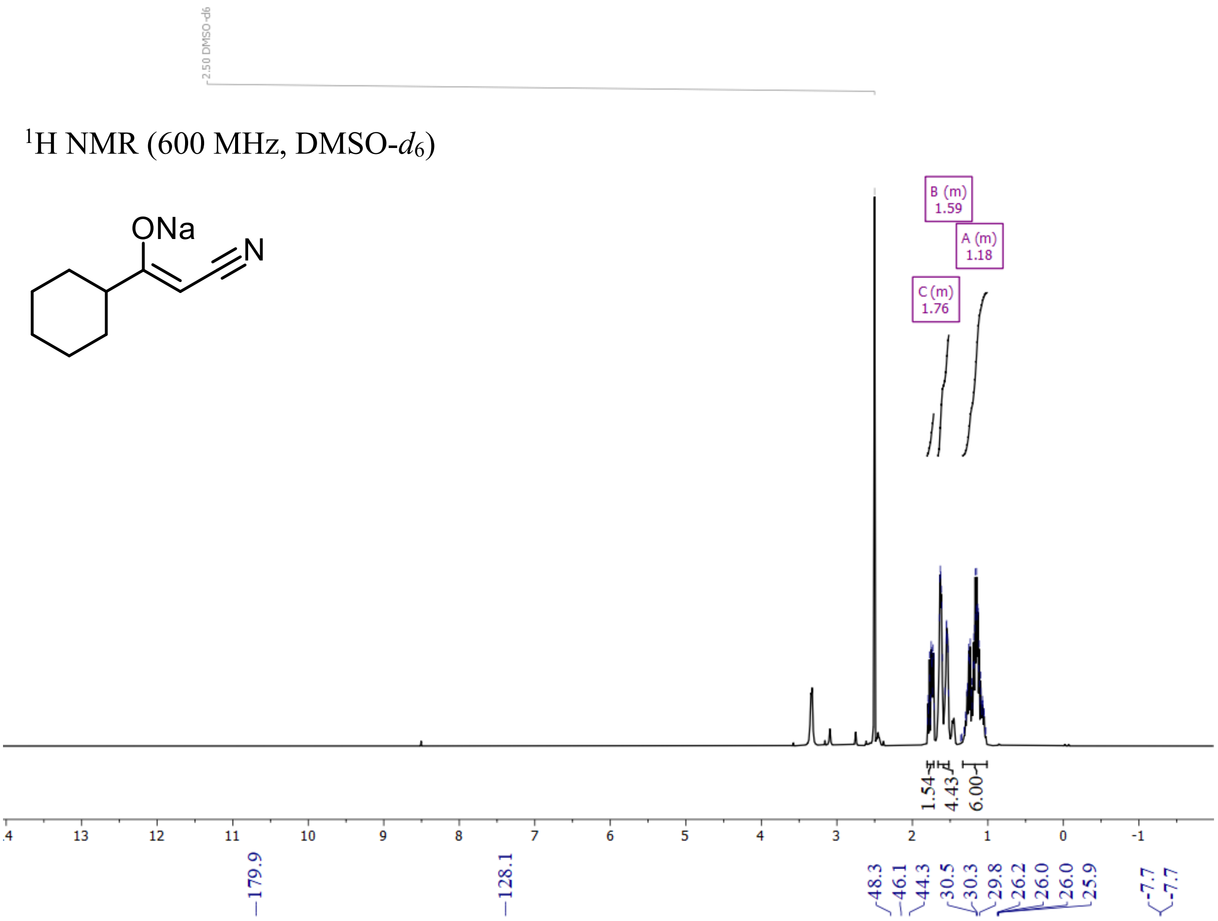

^{13}C NMR (151 MHz, DMSO-d_6)

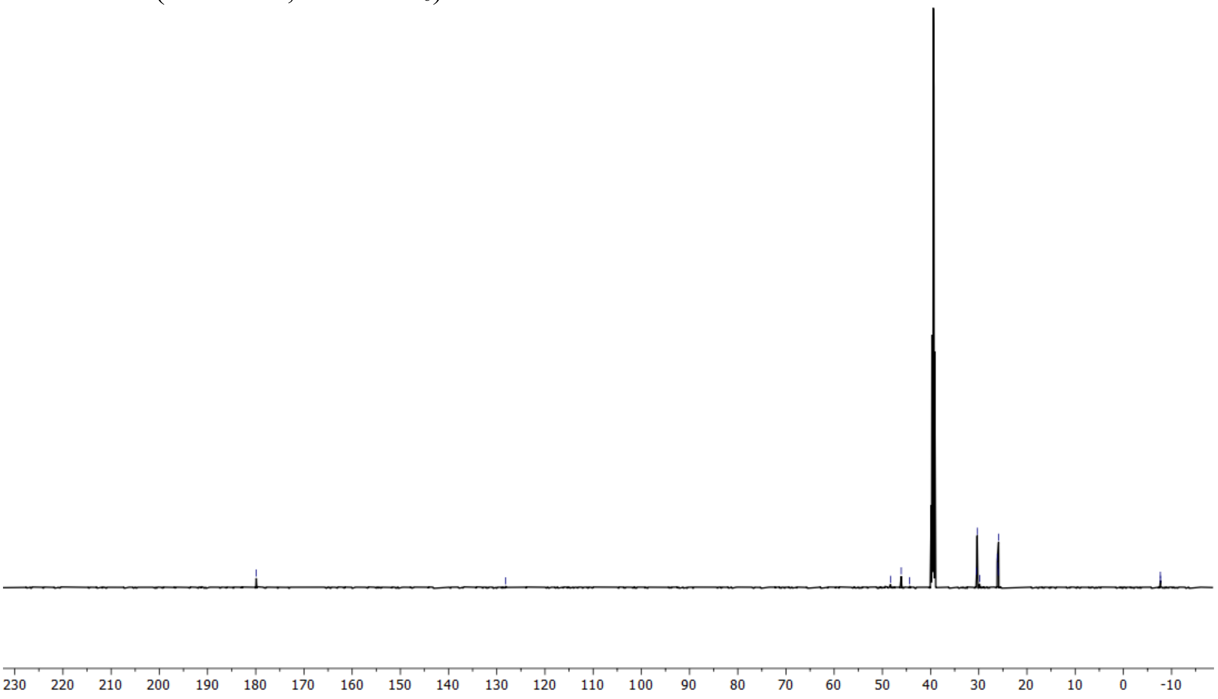

3-(pyridin-3-yl)-1*H*-pyrazol-5-amine (7a)

^{1}H NMR (600 MHz, DMSO-d_6)

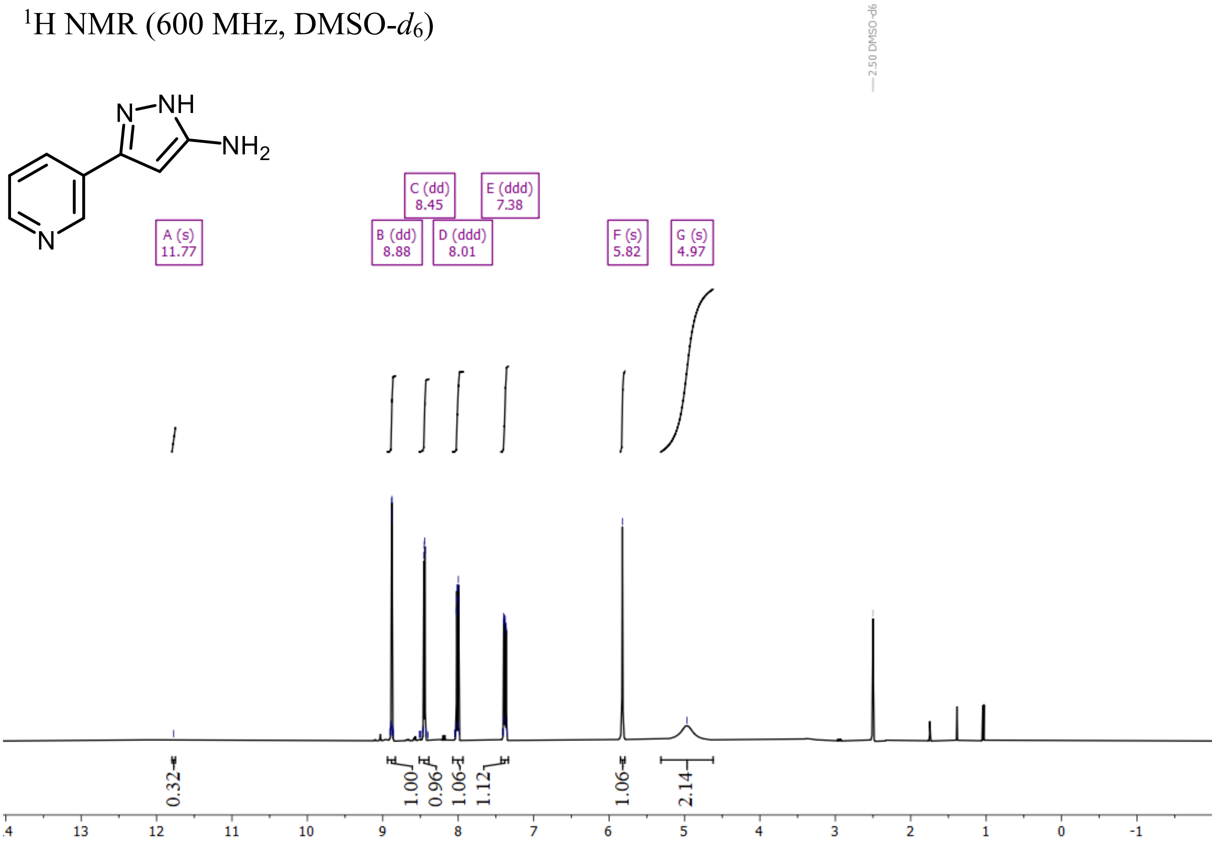

^{13}C NMR (151 MHz, DMSO-d_6)

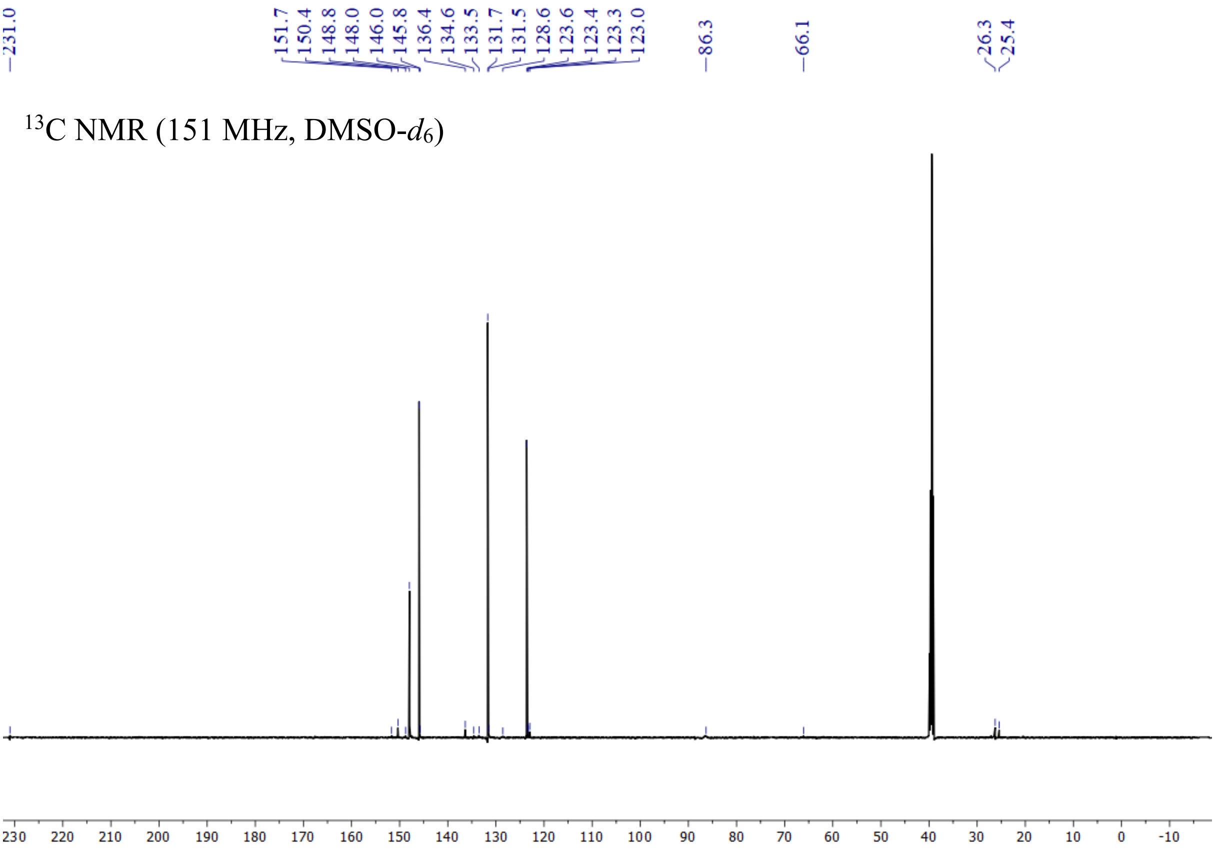

The signals for C-3$_{pyridyl}$ and C-3/4/5$_{pyrazole}$ are not seen in the spectrum.

3-phenyl-1*H*-pyrazol-5-amine (7b)

^{1}H NMR (600 MHz, DMSO-d_6)

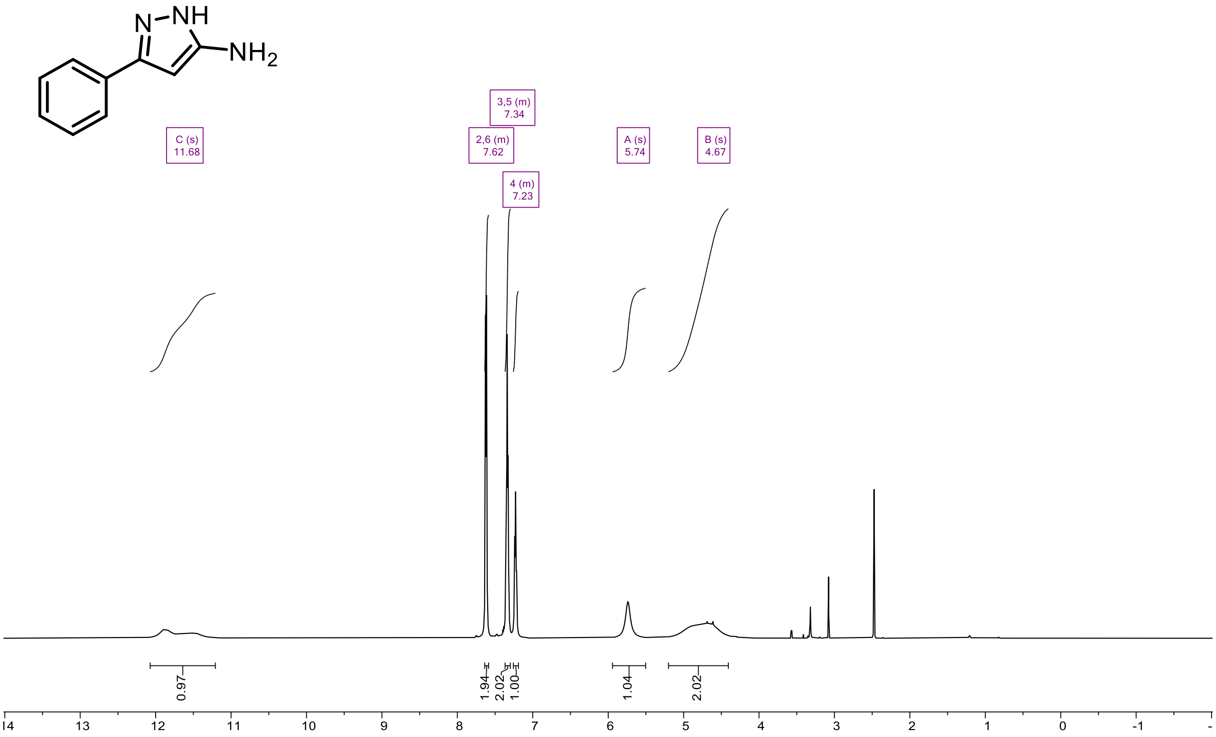

^{13}C NMR (151 MHz, DMSO-d_6)

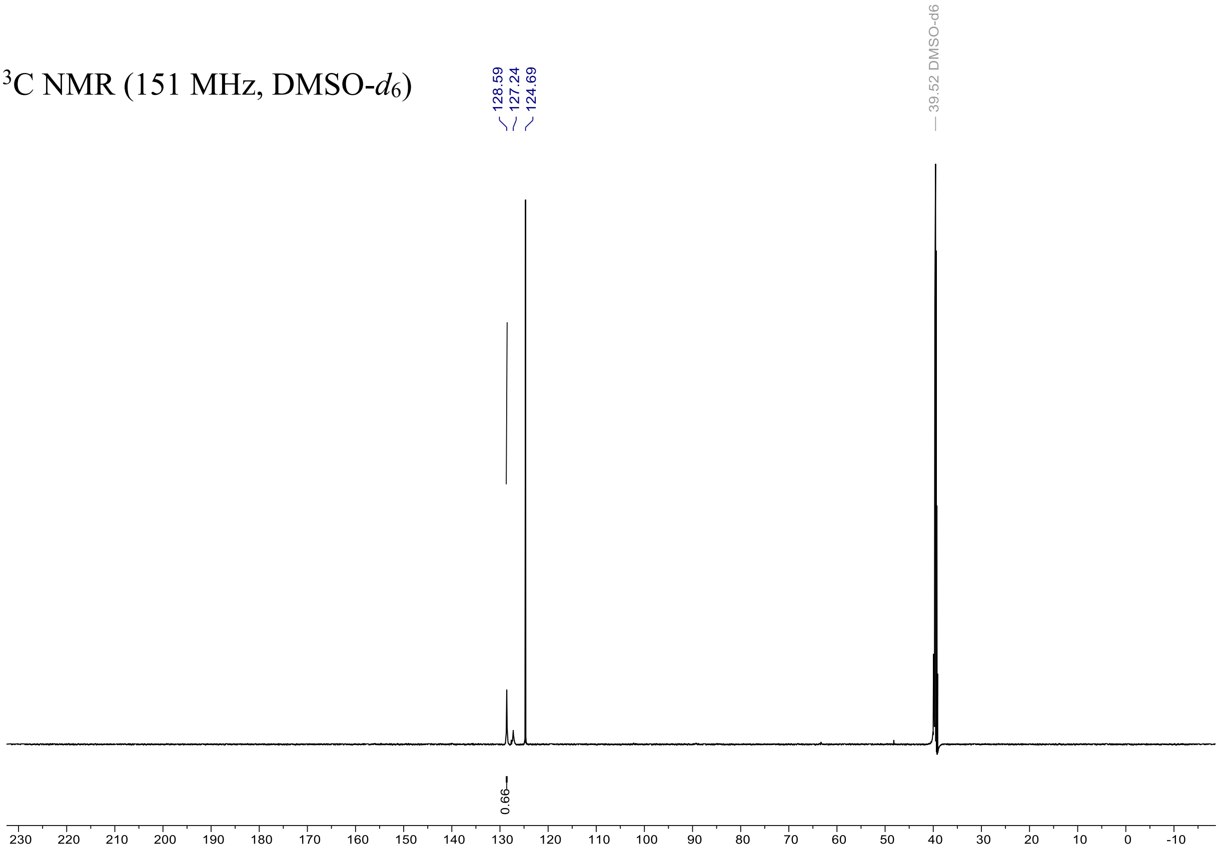

The signals for C-1$_{phenyl}$ and C-3/4/5$_{pyrazole}$ are not seen in the spectrum.

3-cyclohexyl-1*H*-pyrazol-5-amine (7c)

^{1}H NMR (600 MHz, DMSO-d_6)

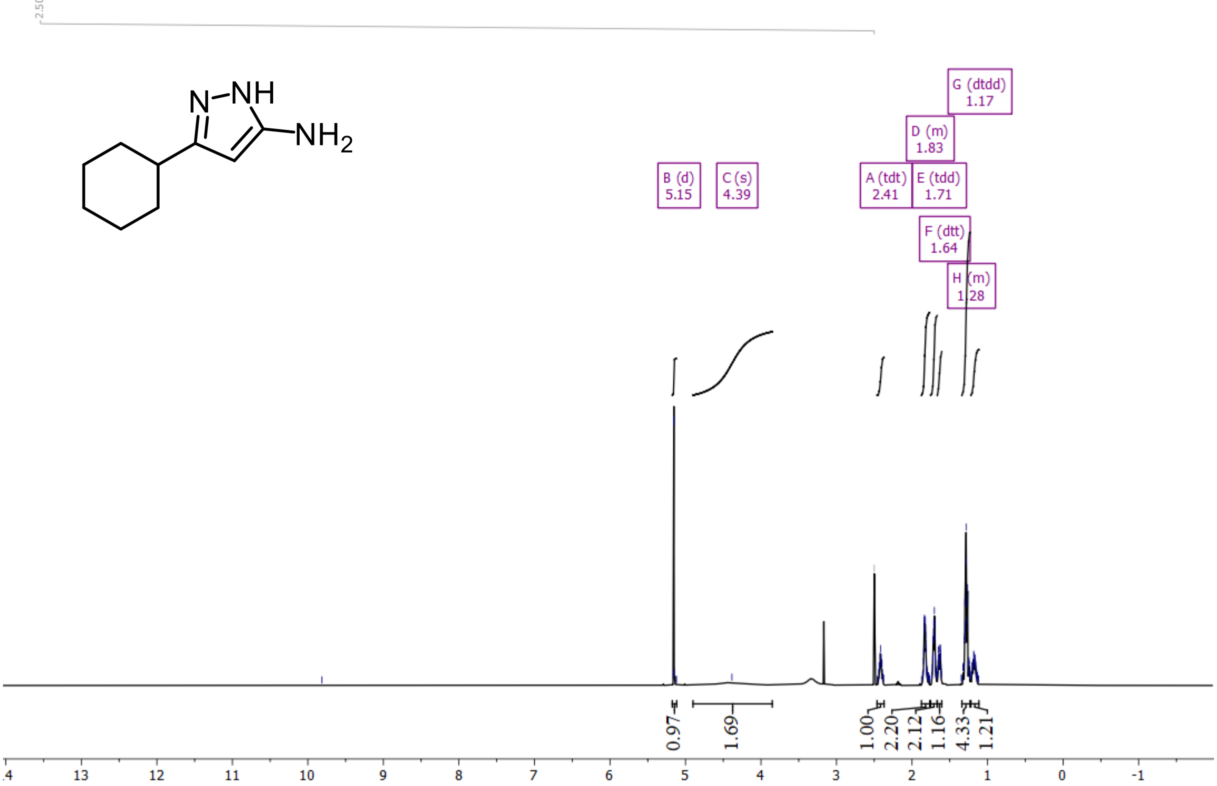

^{13}C NMR (151 MHz, DMSO-d_6)

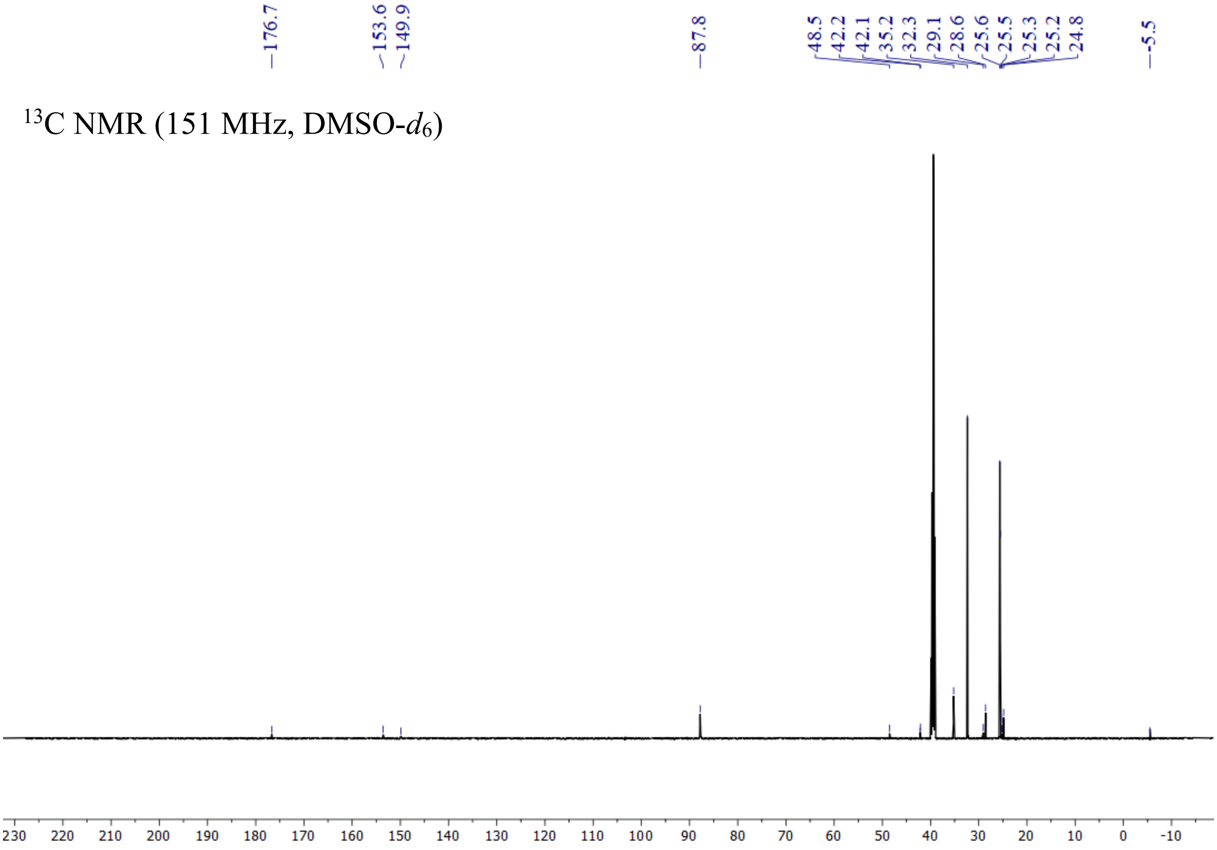

N-benzyl-3-(pyridin-3-yl)-1*H*-pyrazol-5-amine (8a)

^{1}H NMR (600 MHz, DMSO-d_6)

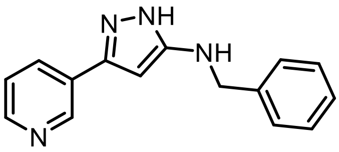

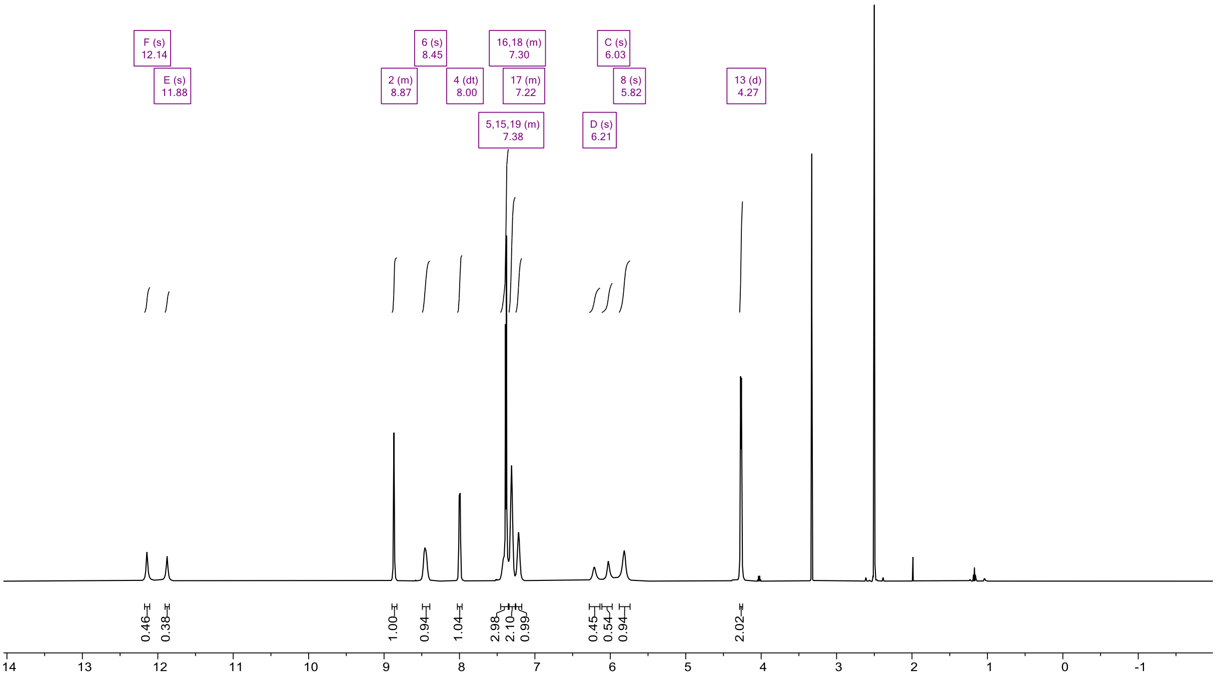

^{13}C NMR (151 MHz, DMSO-d_6)

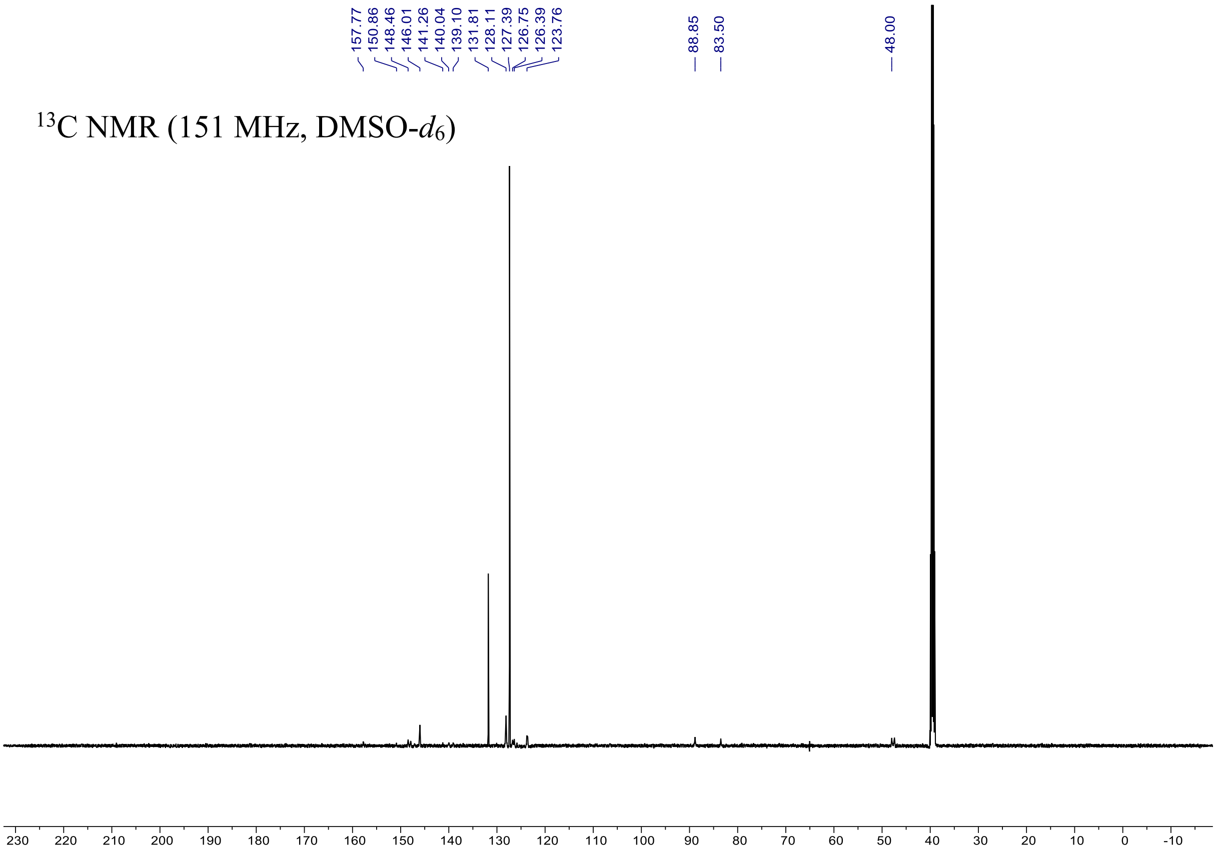

The ratio of tautomers is 1:1. The signal for C-3$_{pyrazole}$ is not seen in the spectrum.

N-(4-methoxybenzyl)-3-(pyridin-3-yl)-1*H*-pyrazol-5-amine (8b)

^{1}H NMR (600 MHz, DMSO-d_6)

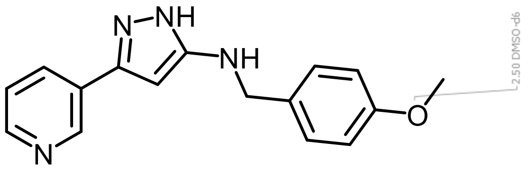

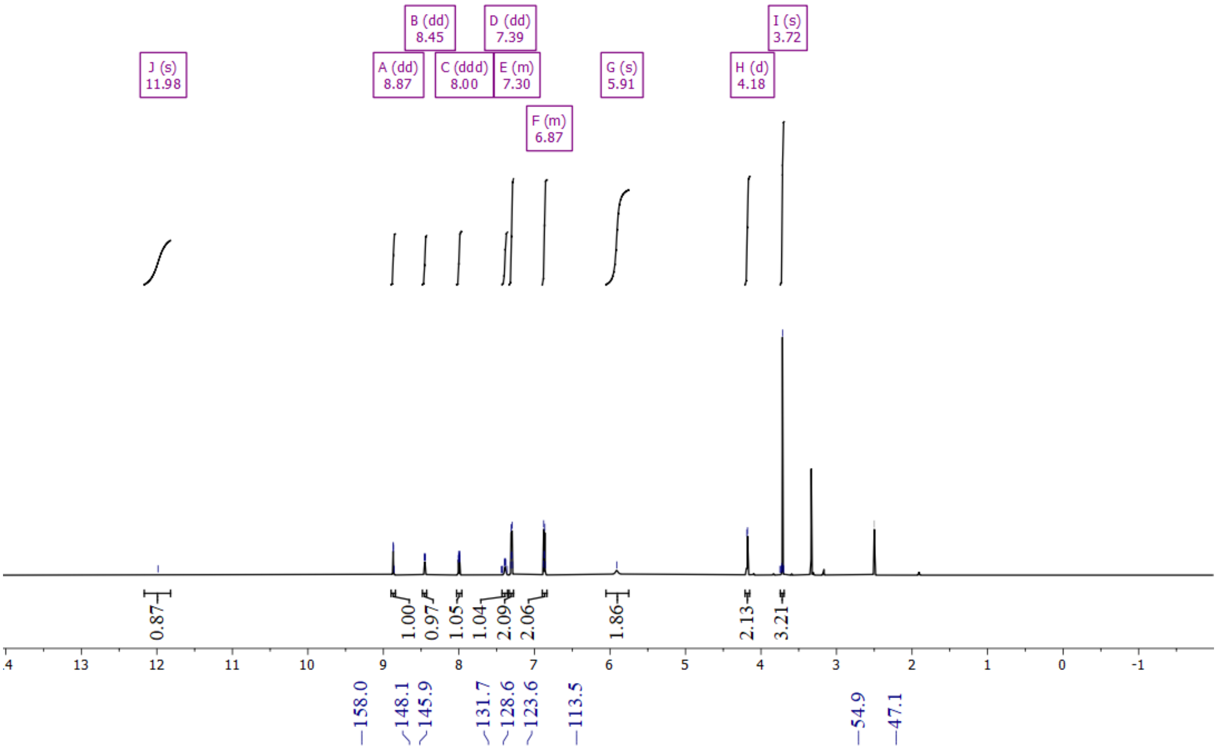

^{13}C NMR (151 MHz, DMSO-d_6)

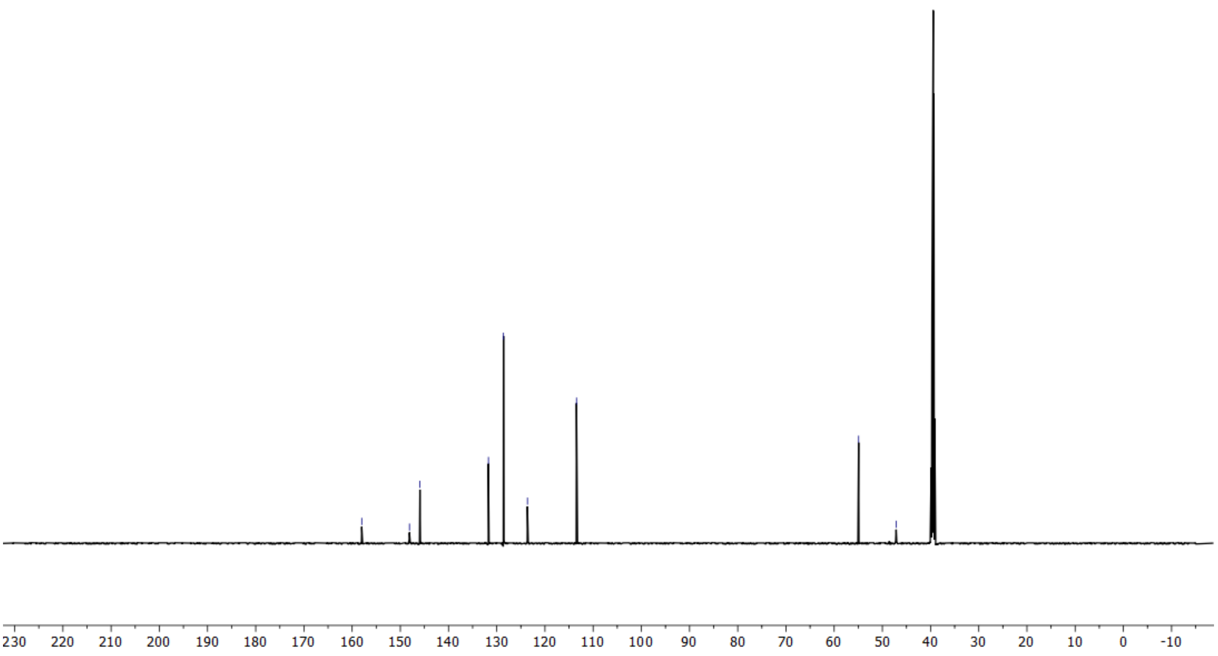

N-(naphthalen-1-ylmethyl)-3-(pyridin-3-yl)-1H-pyrazol-5-amine (8c)

^{1}H NMR (600 MHz, DMSO-d_6)

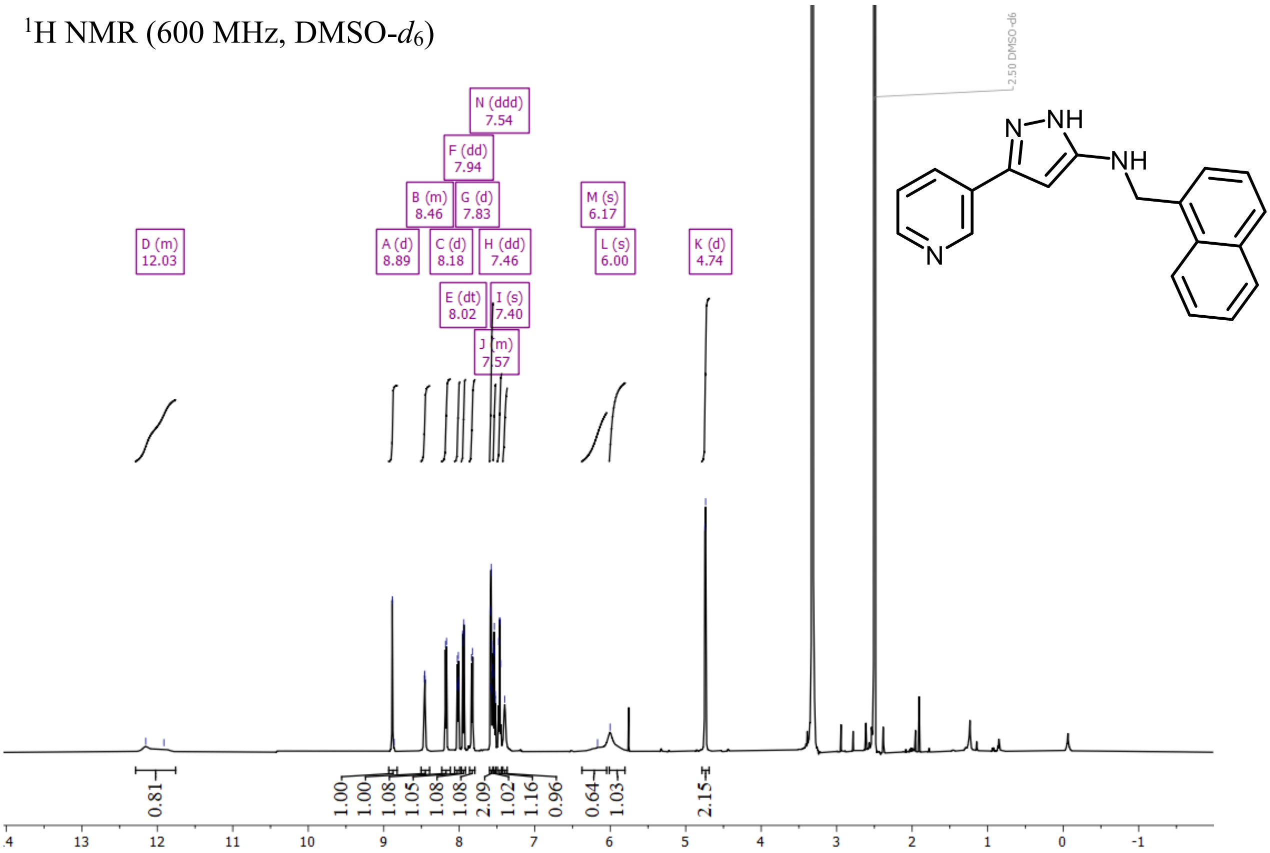

^{13}C NMR (151 MHz, DMSO-d_6)

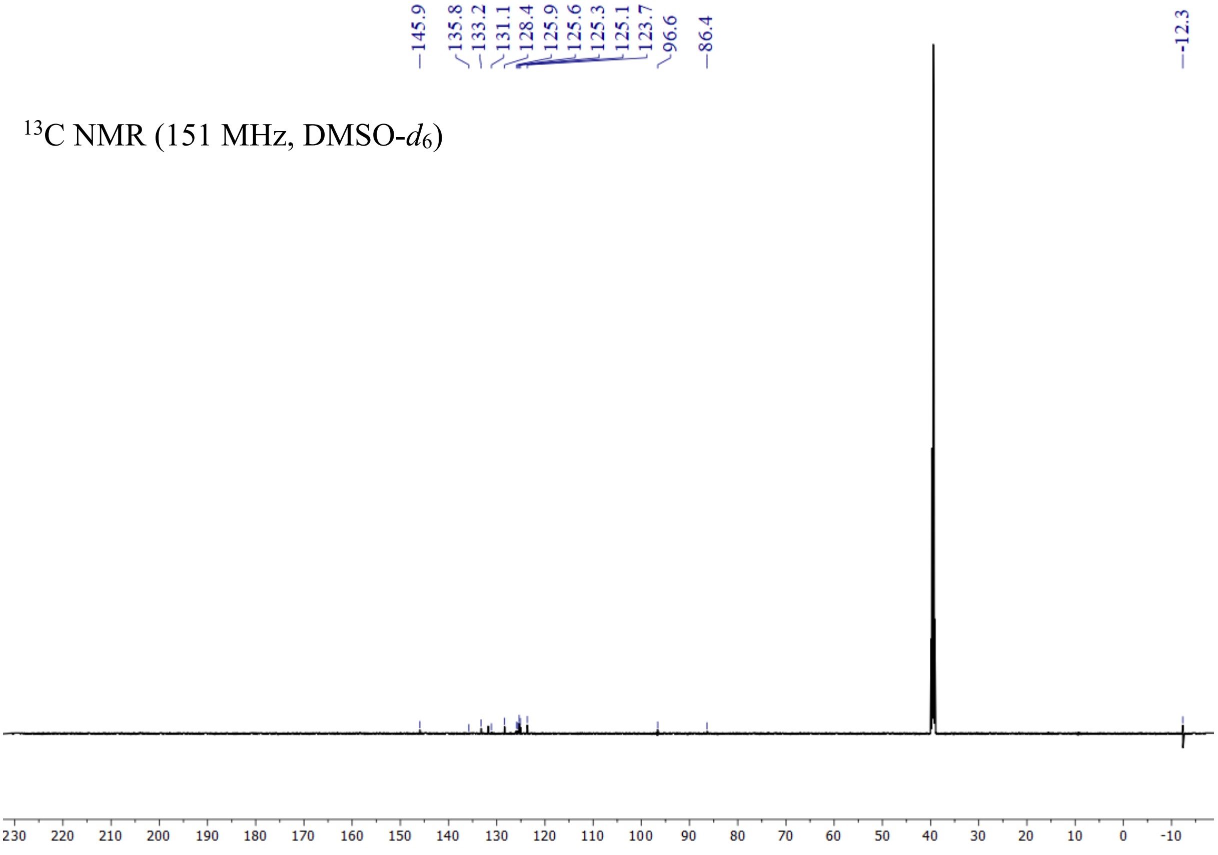

_N_-(furan-2-ylmethyl)-3-(pyridin-3-yl)-1_H_-pyrazol-5-amine (8d)

^{1}H NMR (600 MHz, DMSO-d_6)

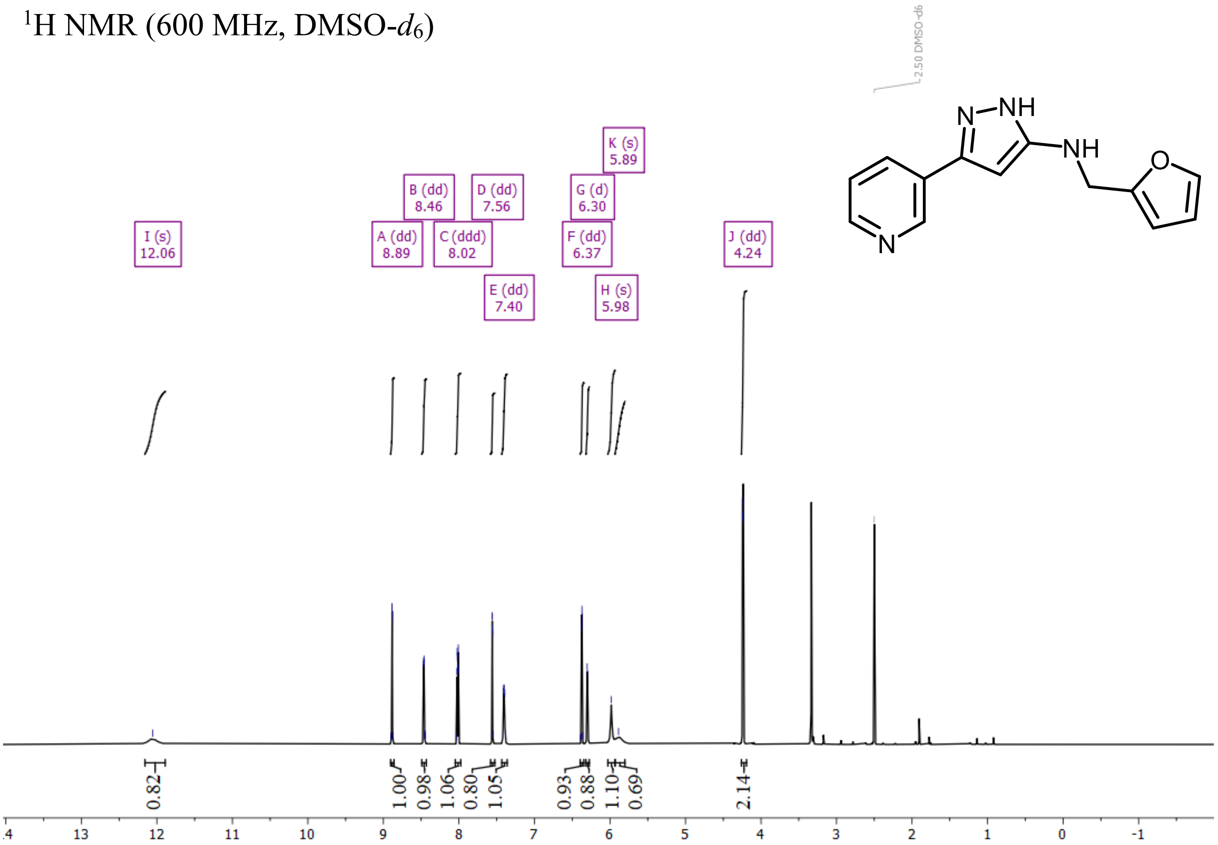

^{13}C NMR (151 MHz, DMSO-d_6)

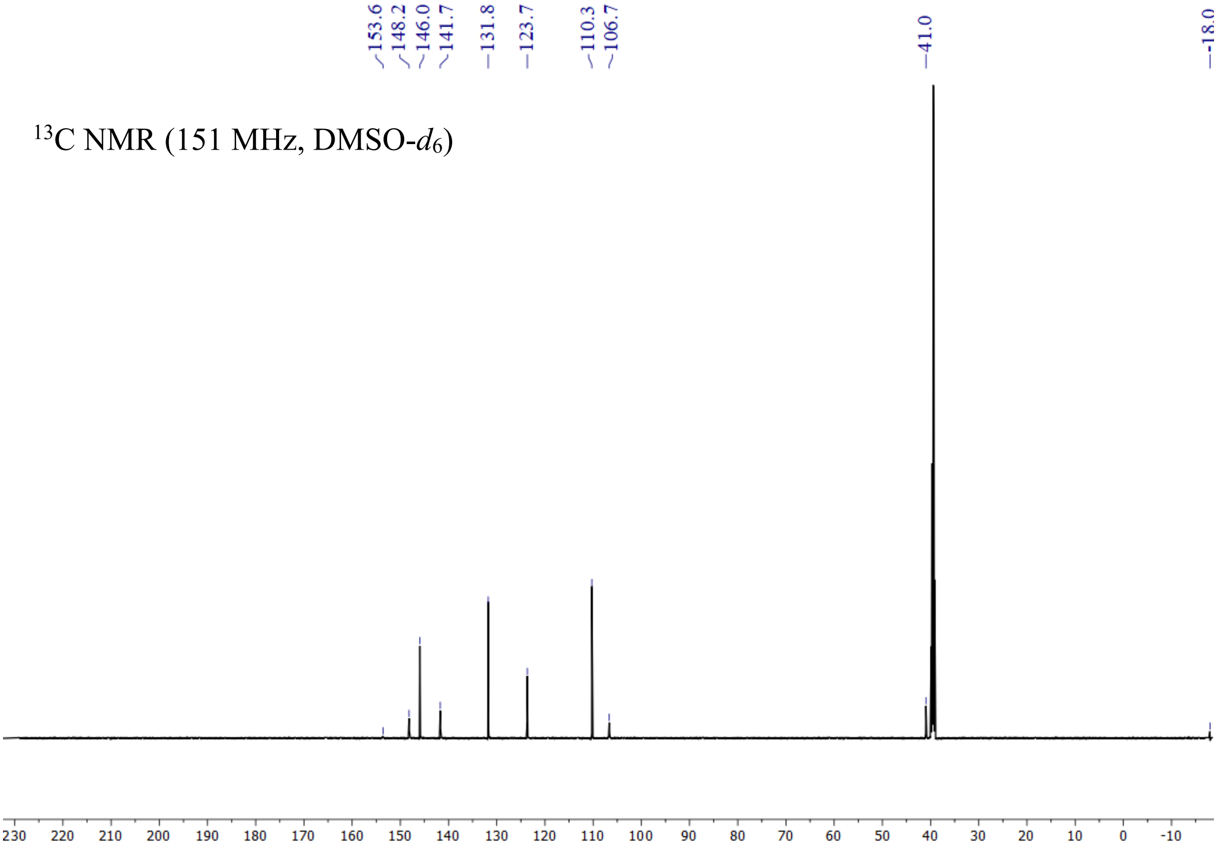

N-((5-chlorothiophen-2-yl)methyl)-3-(pyridin-3-yl)-1H-pyrazol-5-amine (8e)

^{1}H NMR (600 MHz, DMSO-d_6)

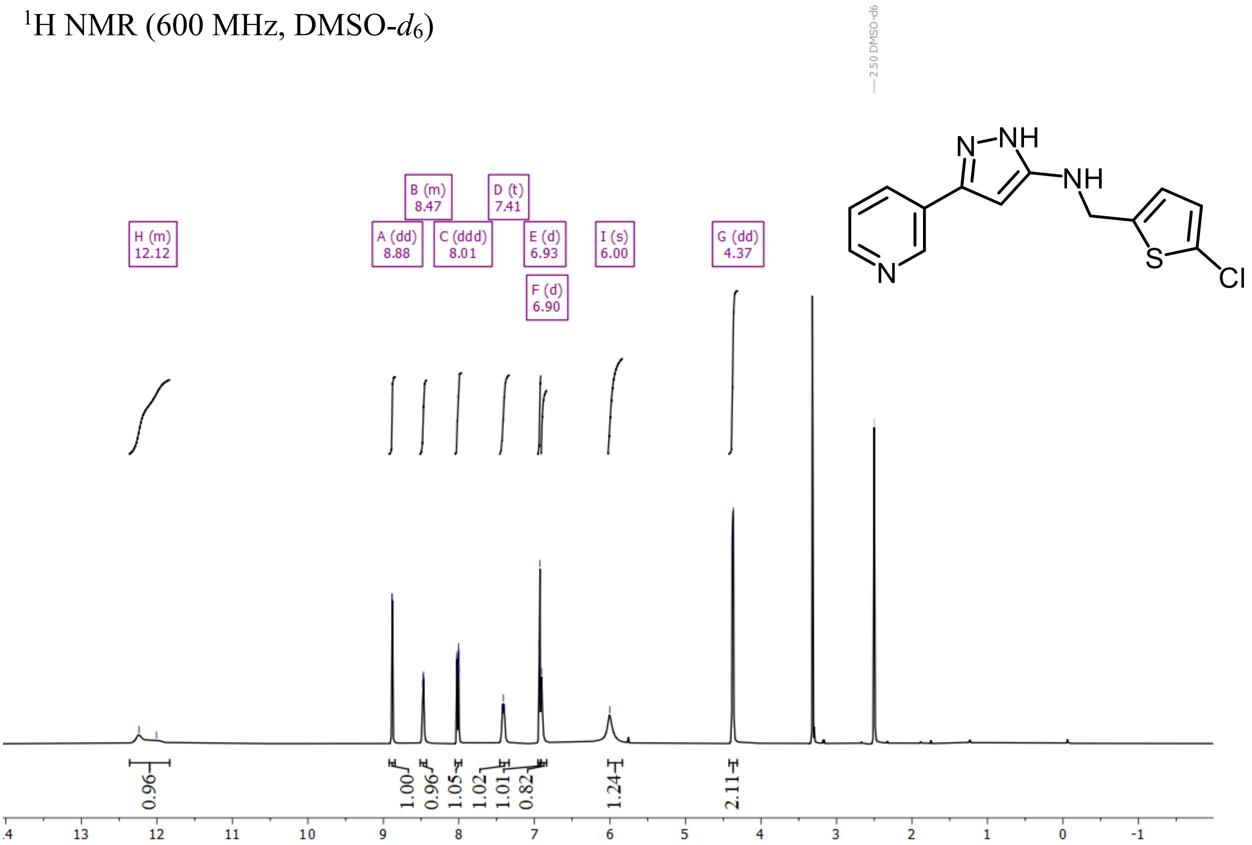

^{13}C NMR (151 MHz, DMSO-d_6)

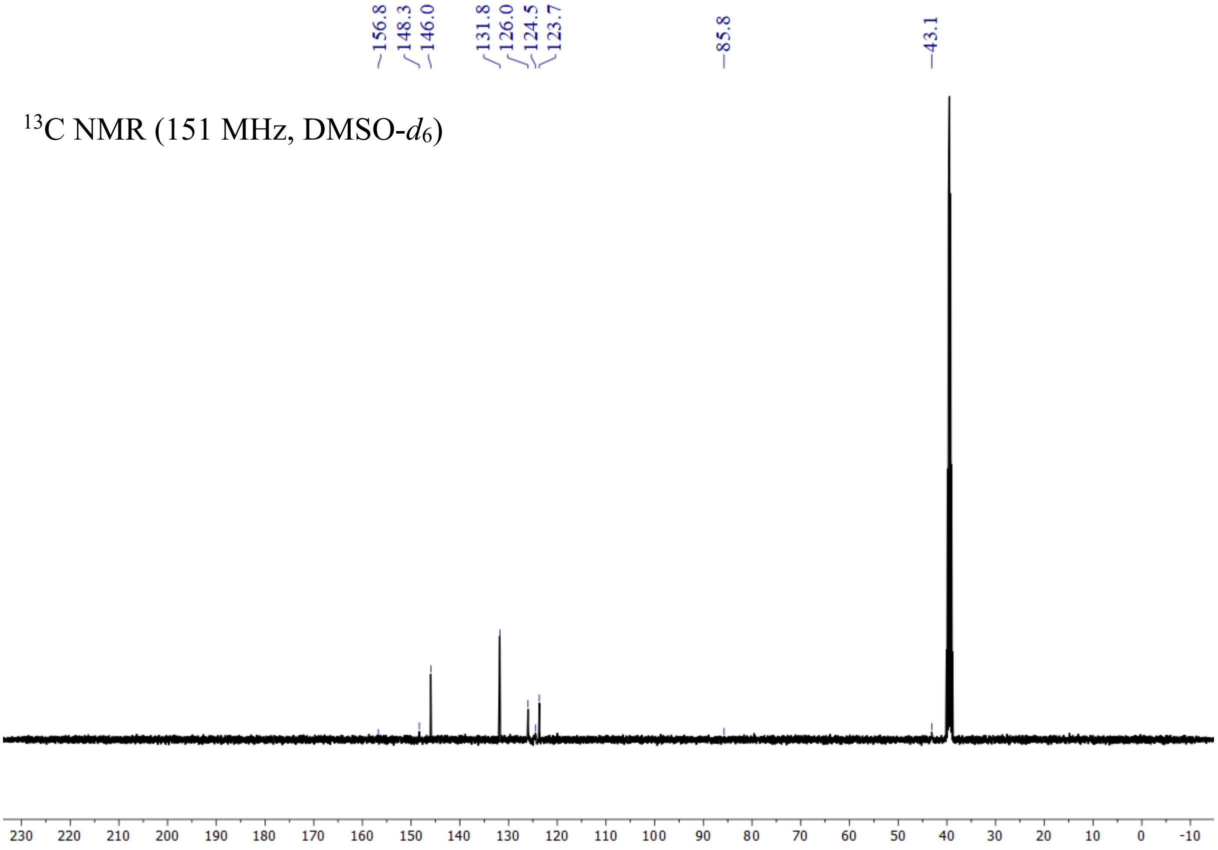

***N*-(cyclohexylmethyl)-3-(pyridin-3-yl)-1*H*-pyrazol-5-amine (8f)**

^{1}H NMR (600 MHz, DMSO-d_6)

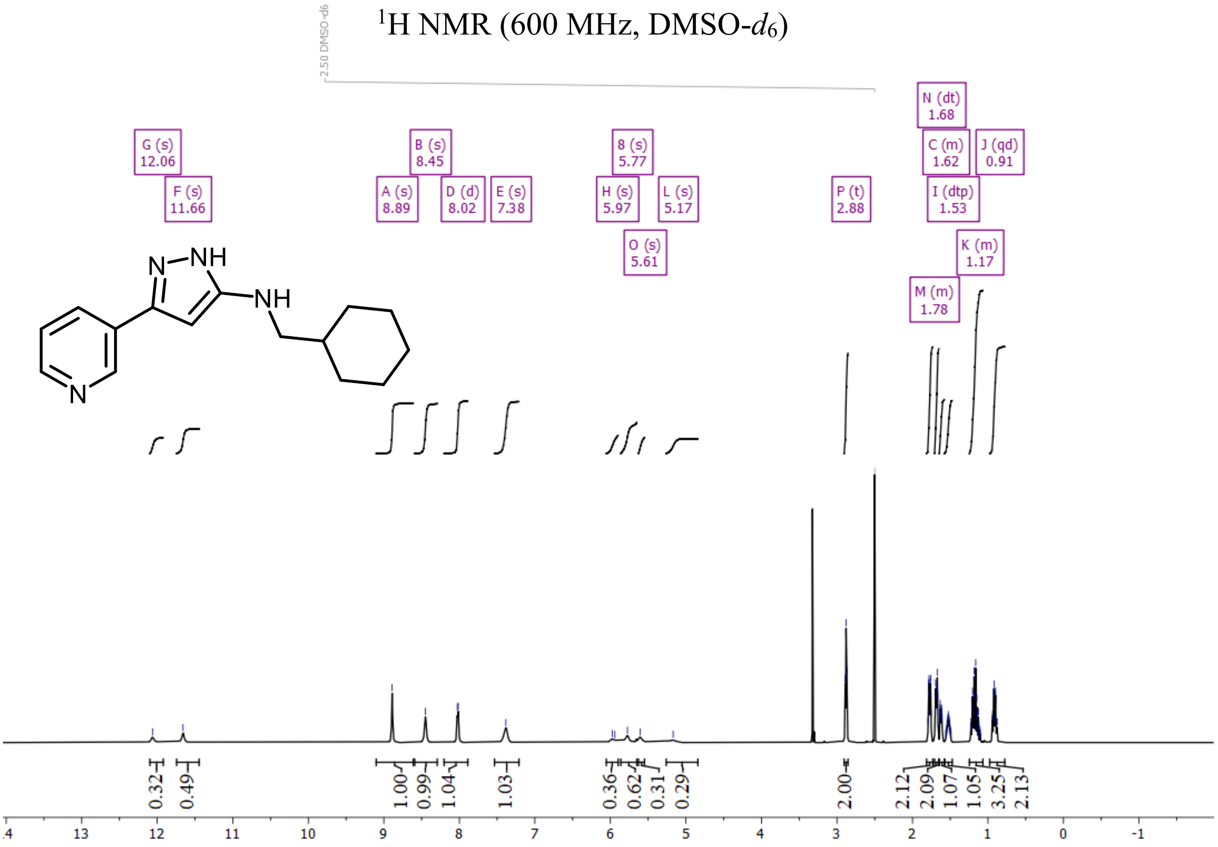

^{13}C NMR (151 MHz, DMSO-d_6)

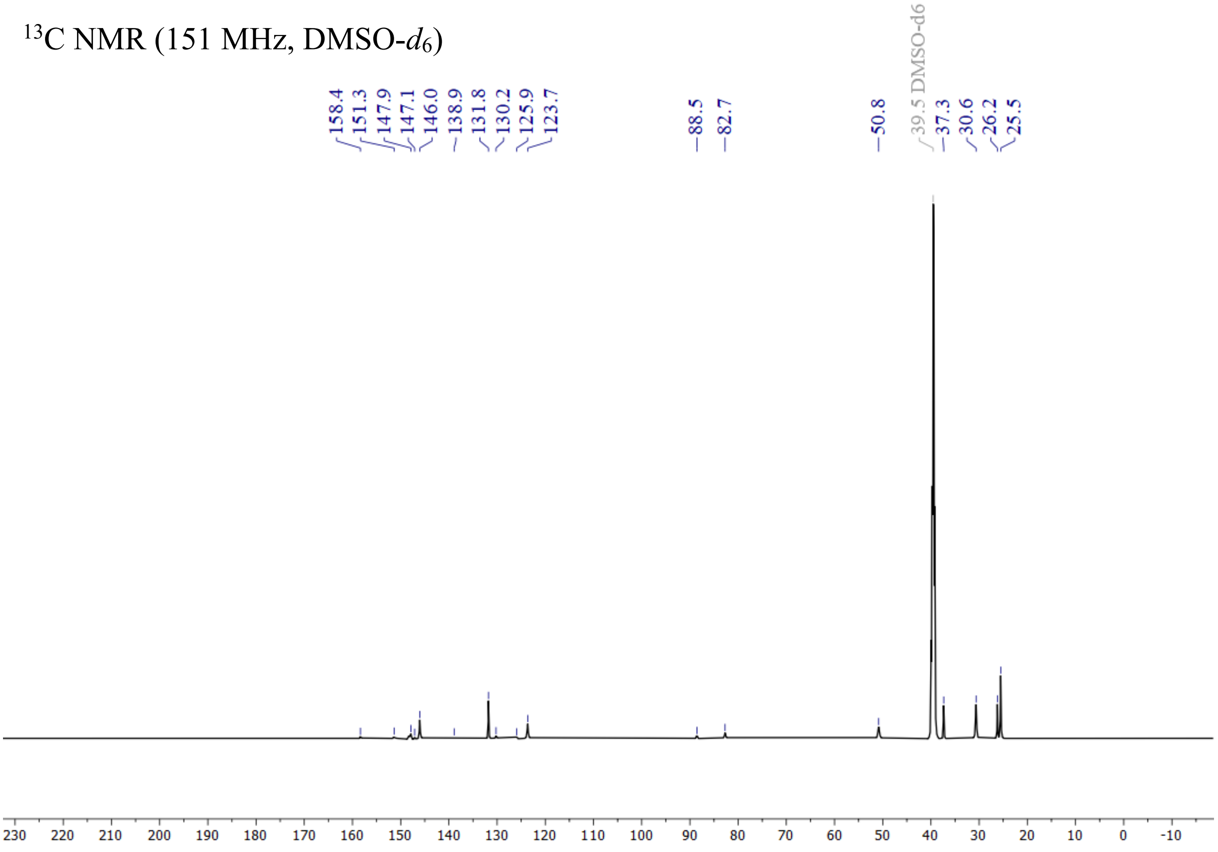

***N*-((5-chlorothiophen-2-yl)methyl)-3-phenyl-1*H*-pyrazol-5-amine (8g)**

^{1}H NMR (600 MHz, DMSO-d_6)

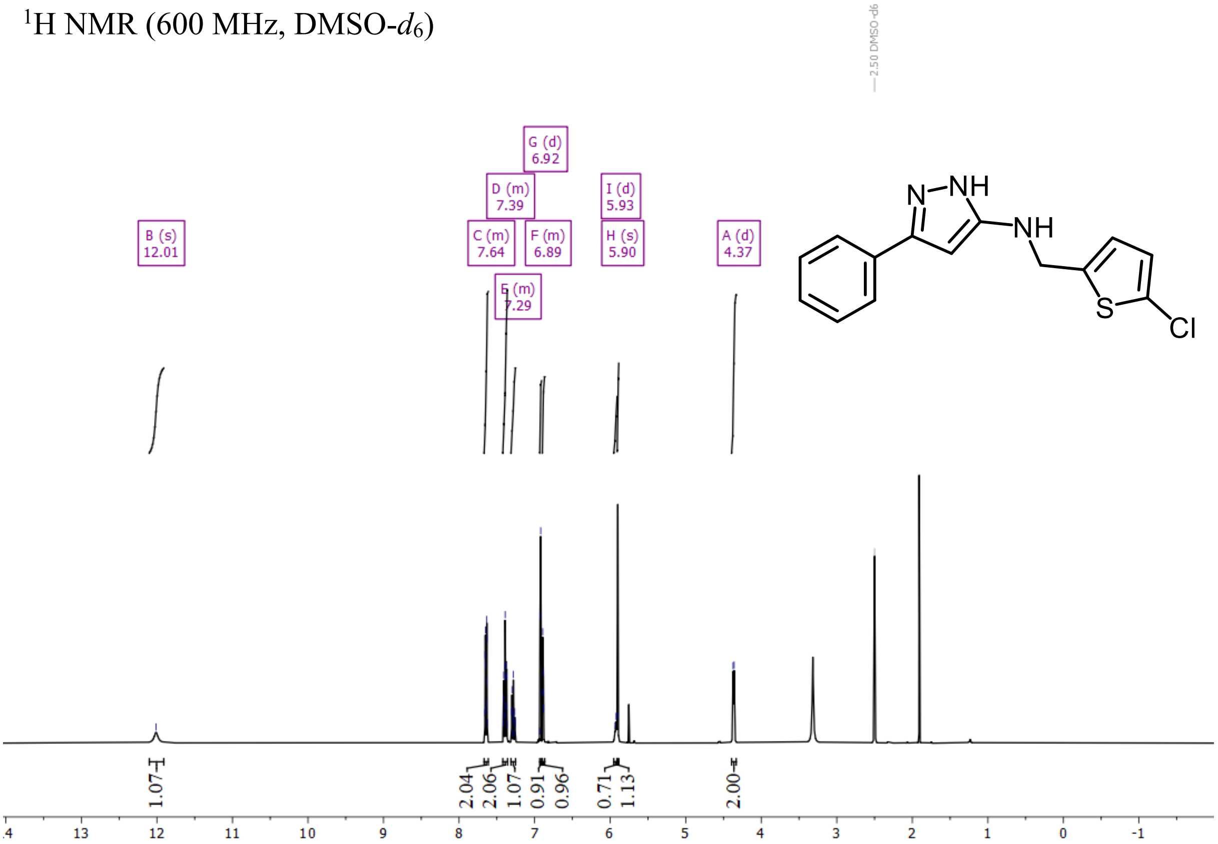

^{13}C NMR (151 MHz, DMSO-d_6)

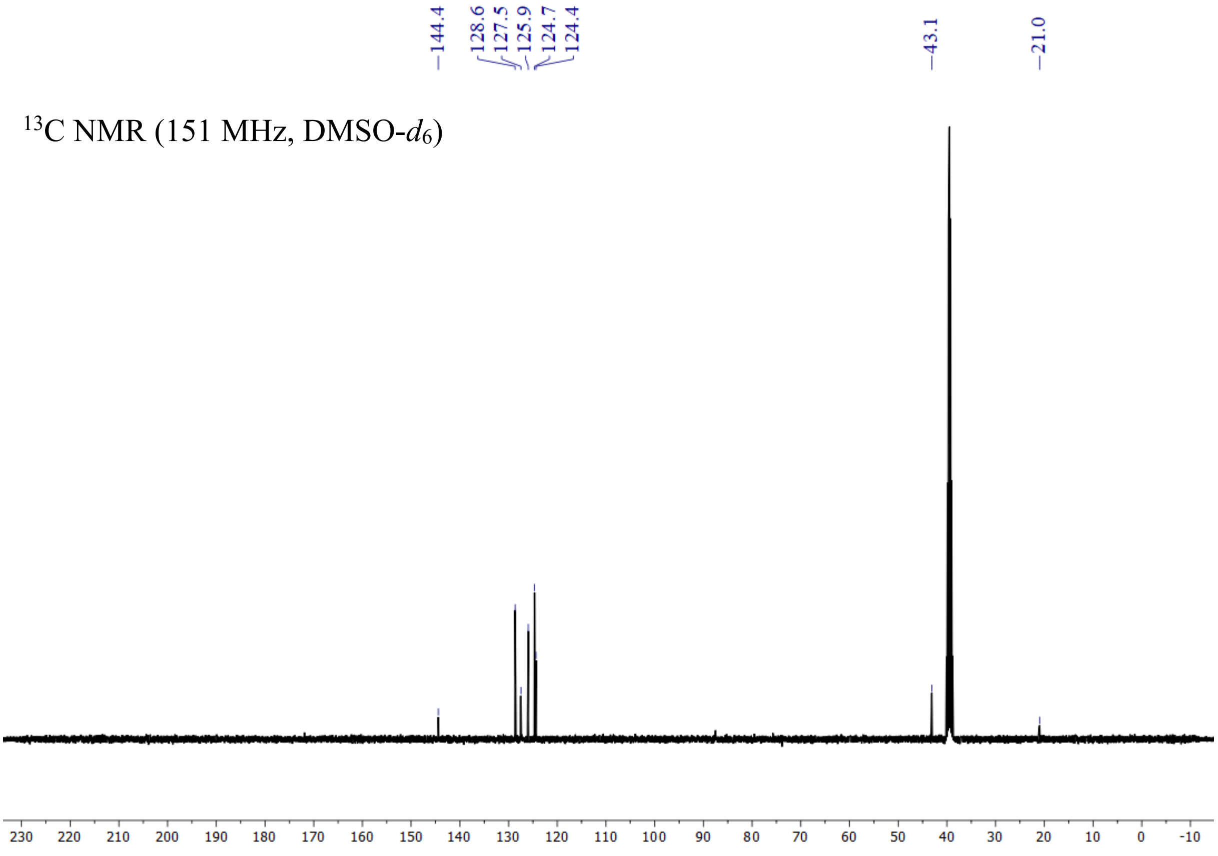

N-benzyl-3-cyclohexyl-1H-pyrazol-5-amine (8h)

^{1}H NMR (600 MHz, DMSO-d_6)

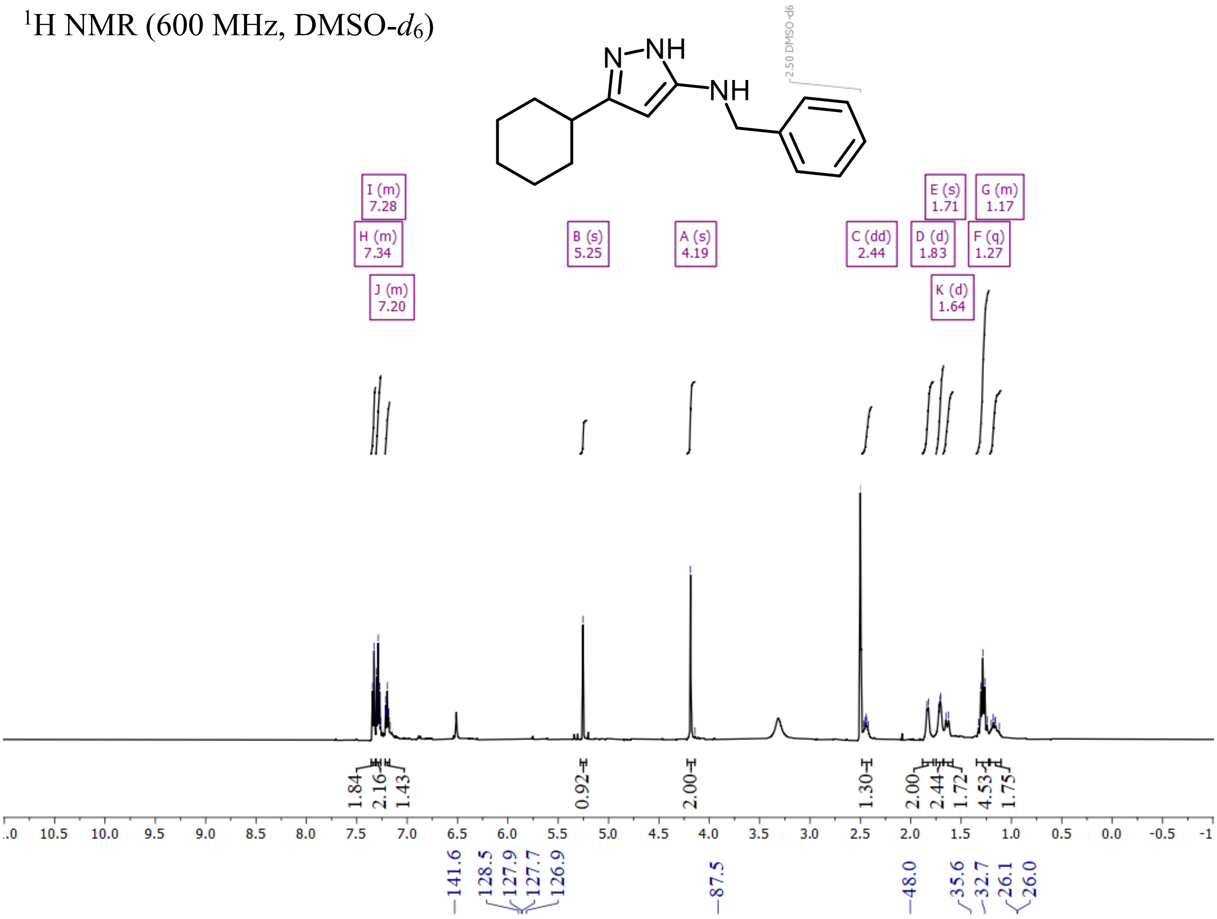

^{13}C NMR (151 MHz, DMSO-d_6)

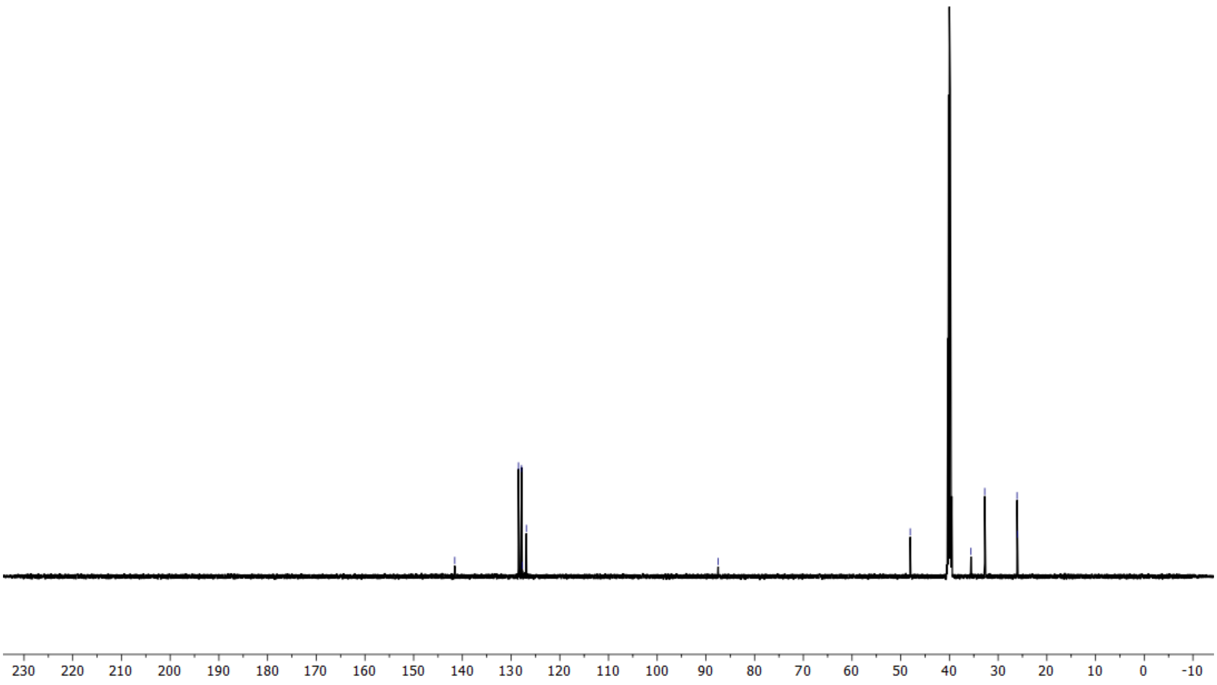

_N_-((5-chlorothiophen-2-yl)methyl)-3-cyclohexyl-1_H_-pyrazol-5-amine (8i)

^{1}H NMR (600 MHz, DMSO-d_6)

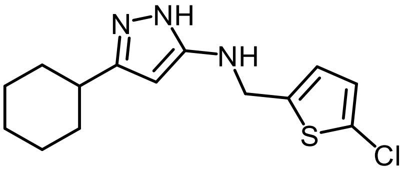

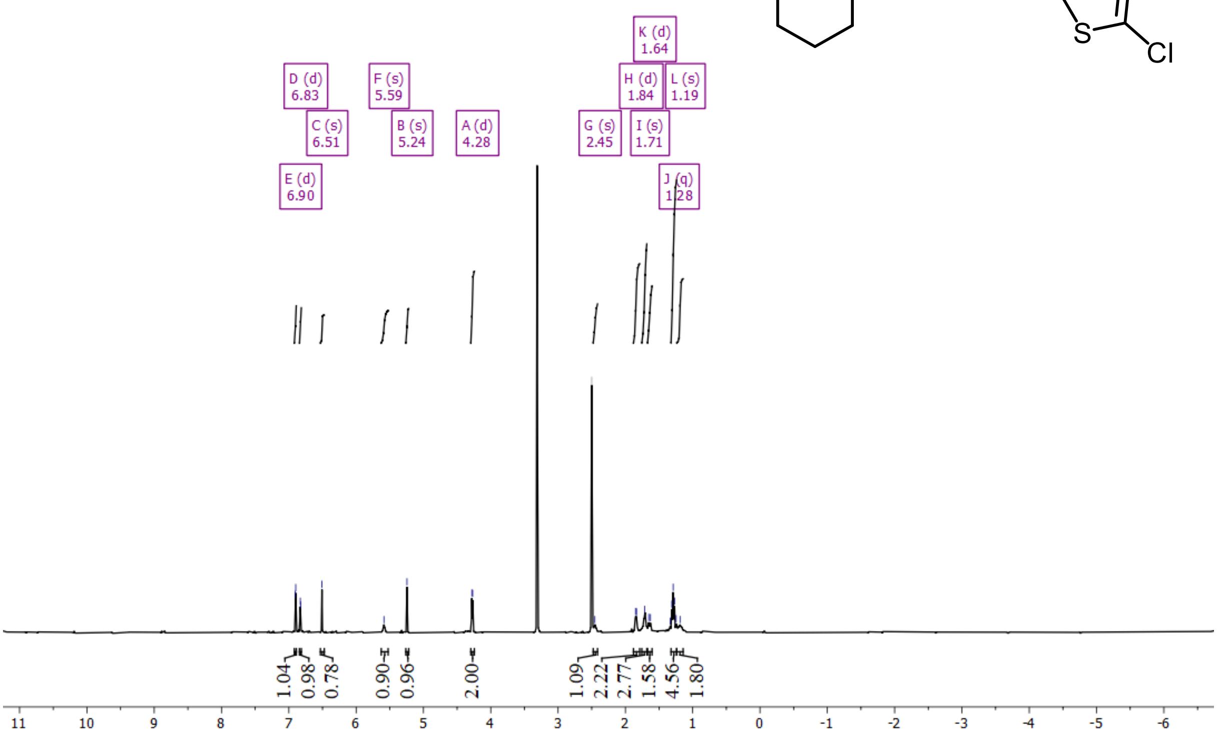

(5-(benzylamino)-3-(pyridin-3-yl)-1*H*-pyrazol-1-yl)(2-iodophenyl)methanone (9a)

^{1}H NMR (600 MHz, DMSO-d_6)

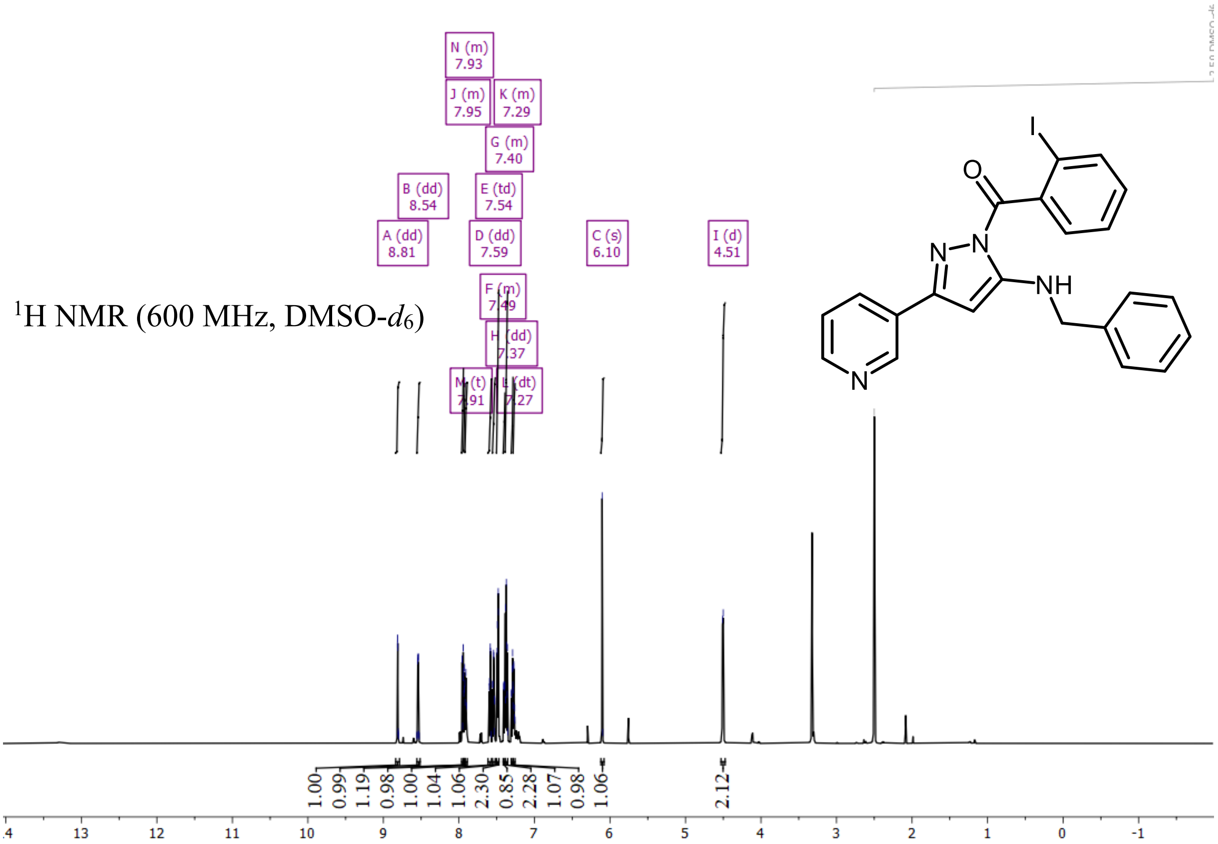

^{13}C NMR (151 MHz, DMSO-d_6)

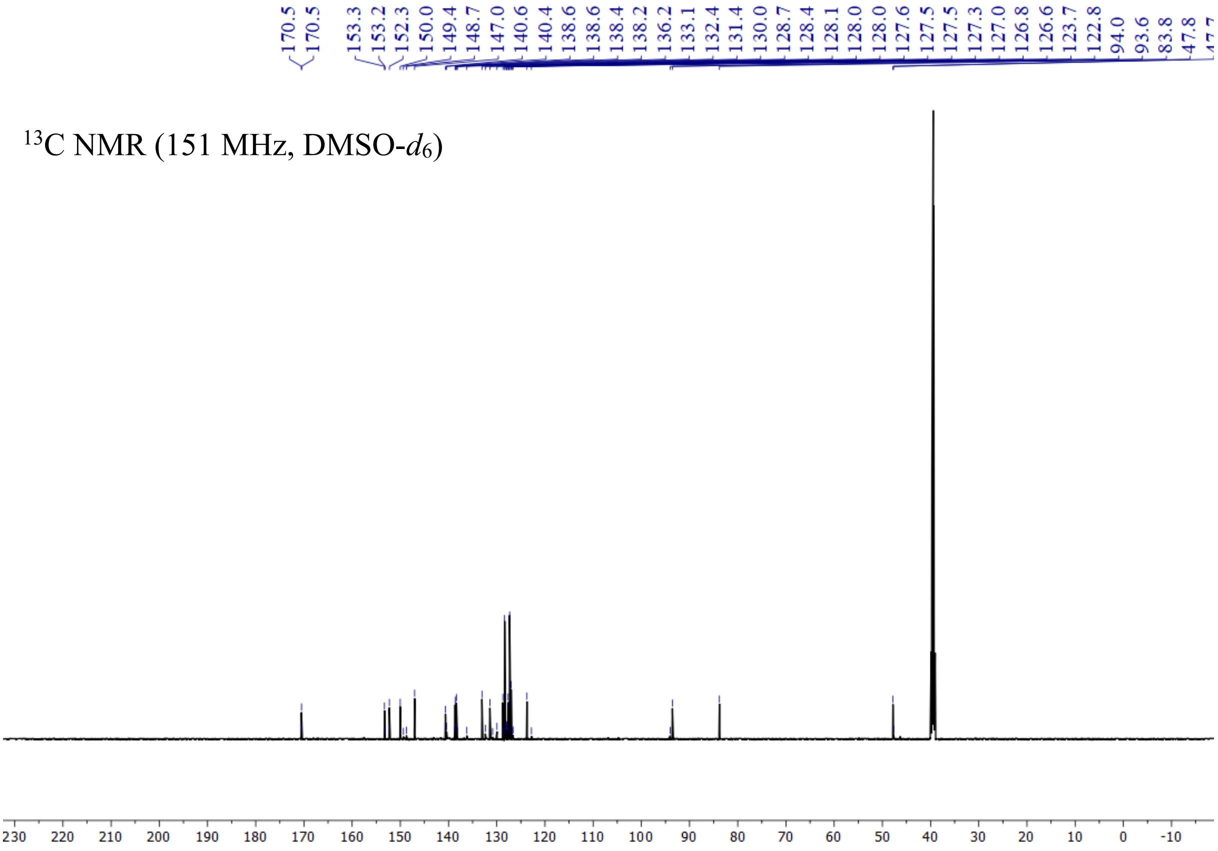

(2-iodophenyl)(5-((4-methoxybenzyl)amino)-3-(pyridin-3-yl)-1*H*-pyrazol-1-yl)methanone (9b)

^{1}H NMR (600 MHz, DMSO-d_6)

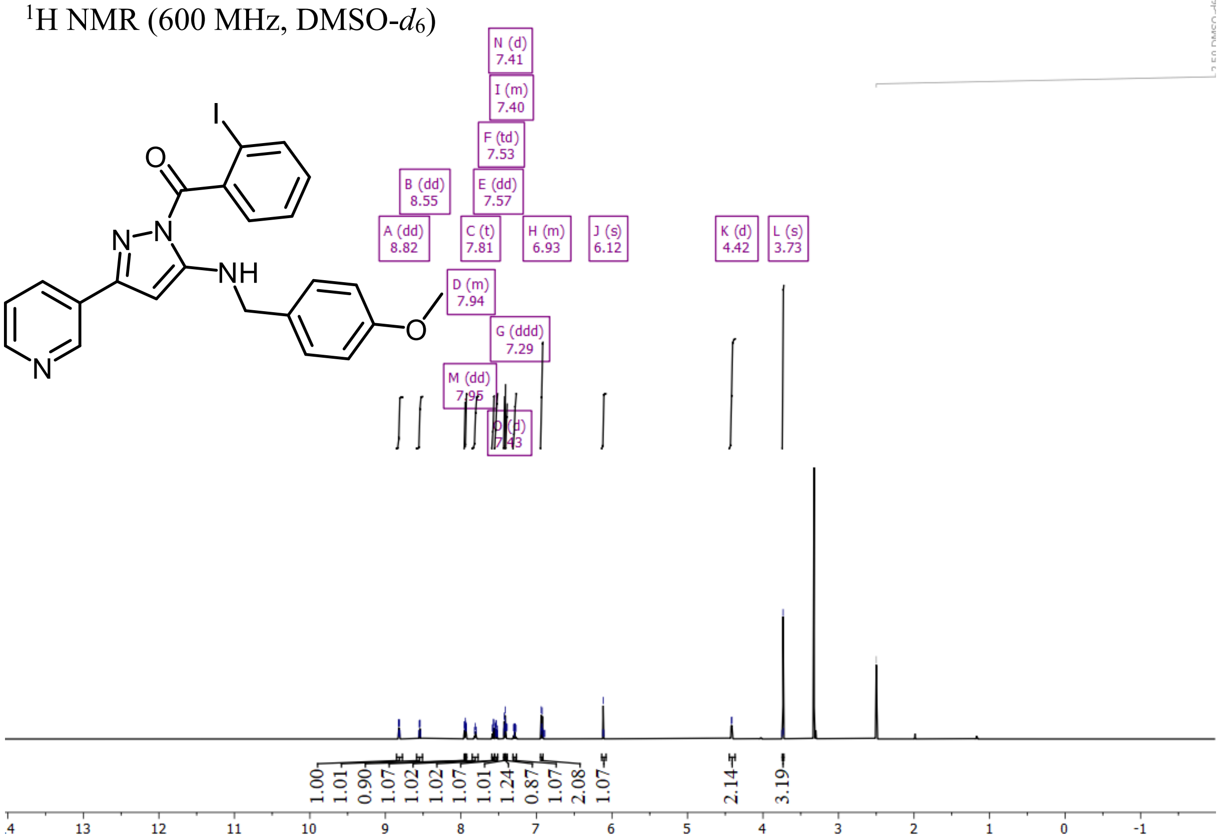

^{13}C NMR (151 MHz, DMSO-d_6)

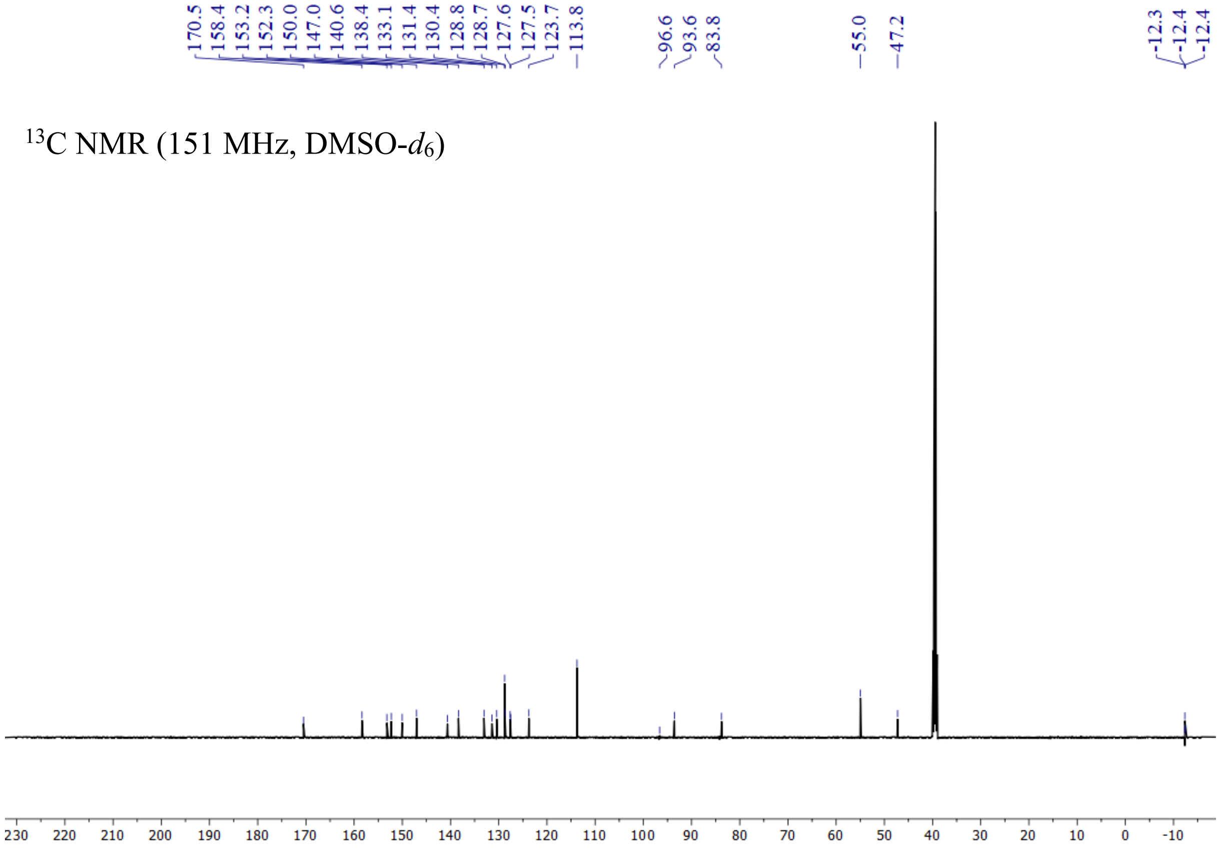

(2-iodophenyl)(5-((naphthalen-1-ylmethyl)amino)-3-(pyridin-3-yl)-1*H*-pyrazol-1-yl)methanone (9c)

^{1}H NMR (600 MHz, DMSO-d_6)

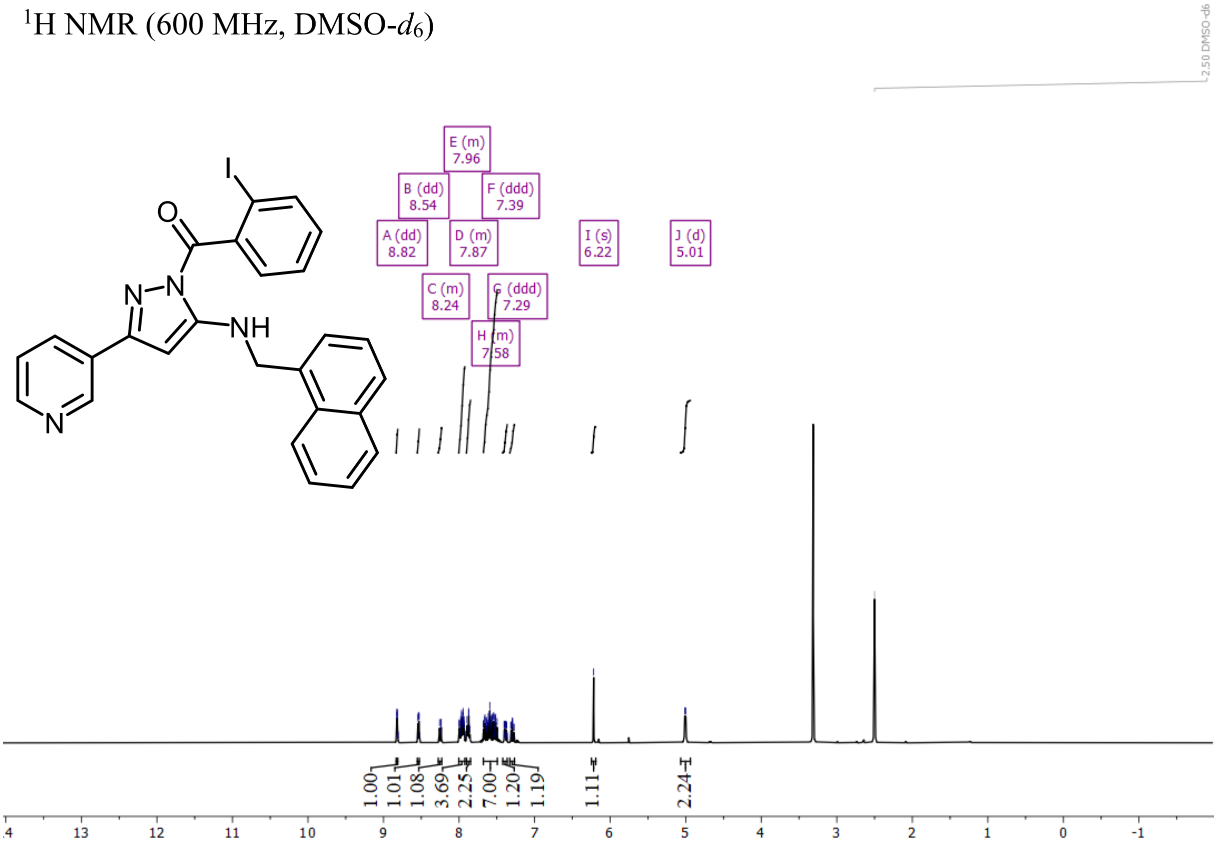

^{13}C NMR (151 MHz, DMSO-d_6)

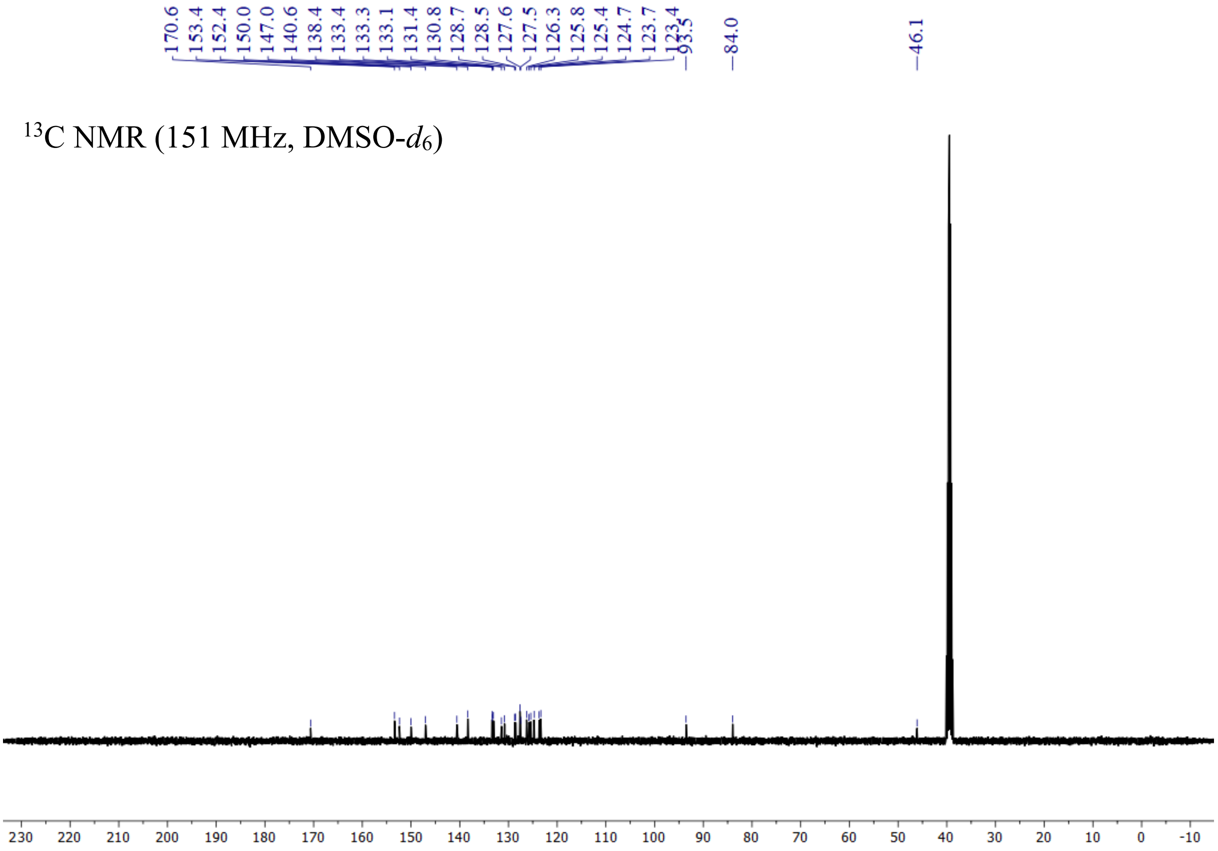

(5-((furan-2-ylmethyl)amino)-3-(pyridin-3-yl)-1*H*-pyrazol-1-yl)(2-iodophenyl)methanone (9d)

^{1}H NMR (600 MHz, DMSO-d_6)

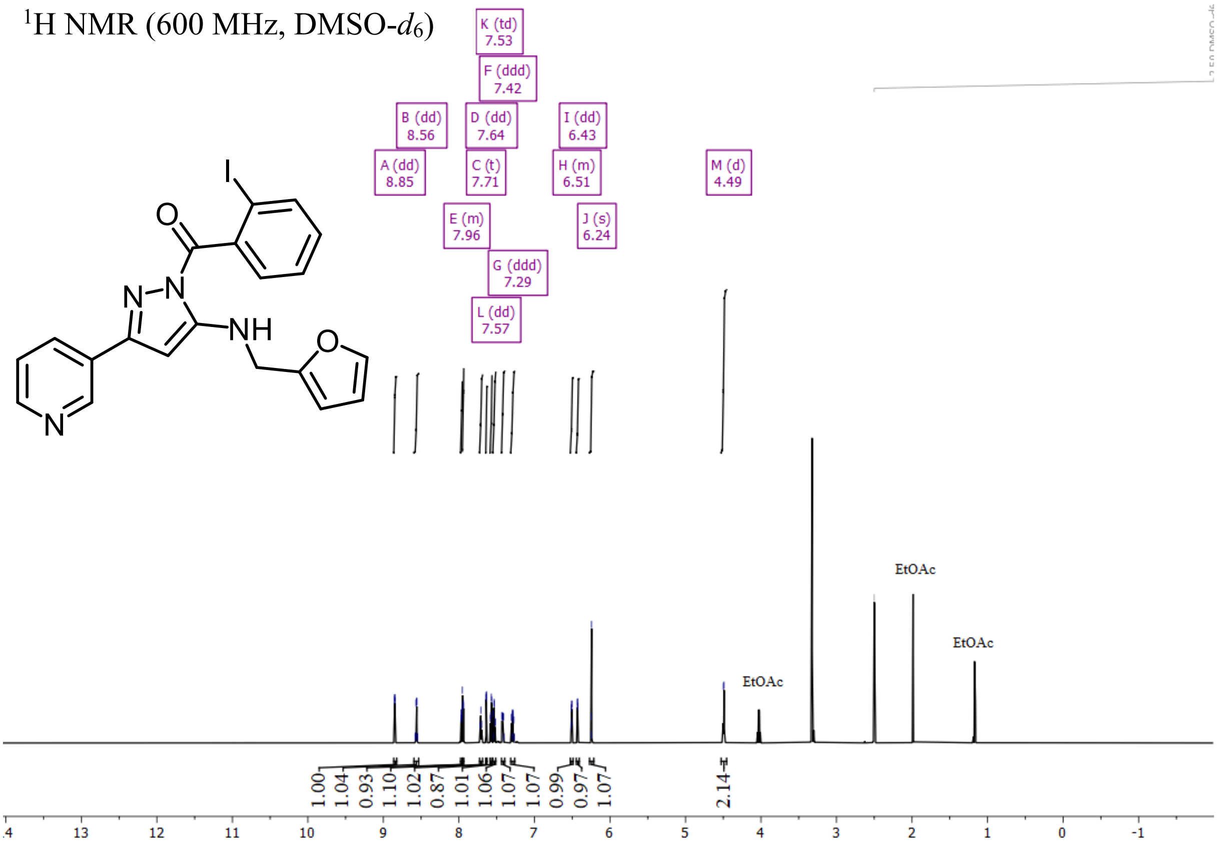

^{13}C NMR (151 MHz, DMSO-d_6)

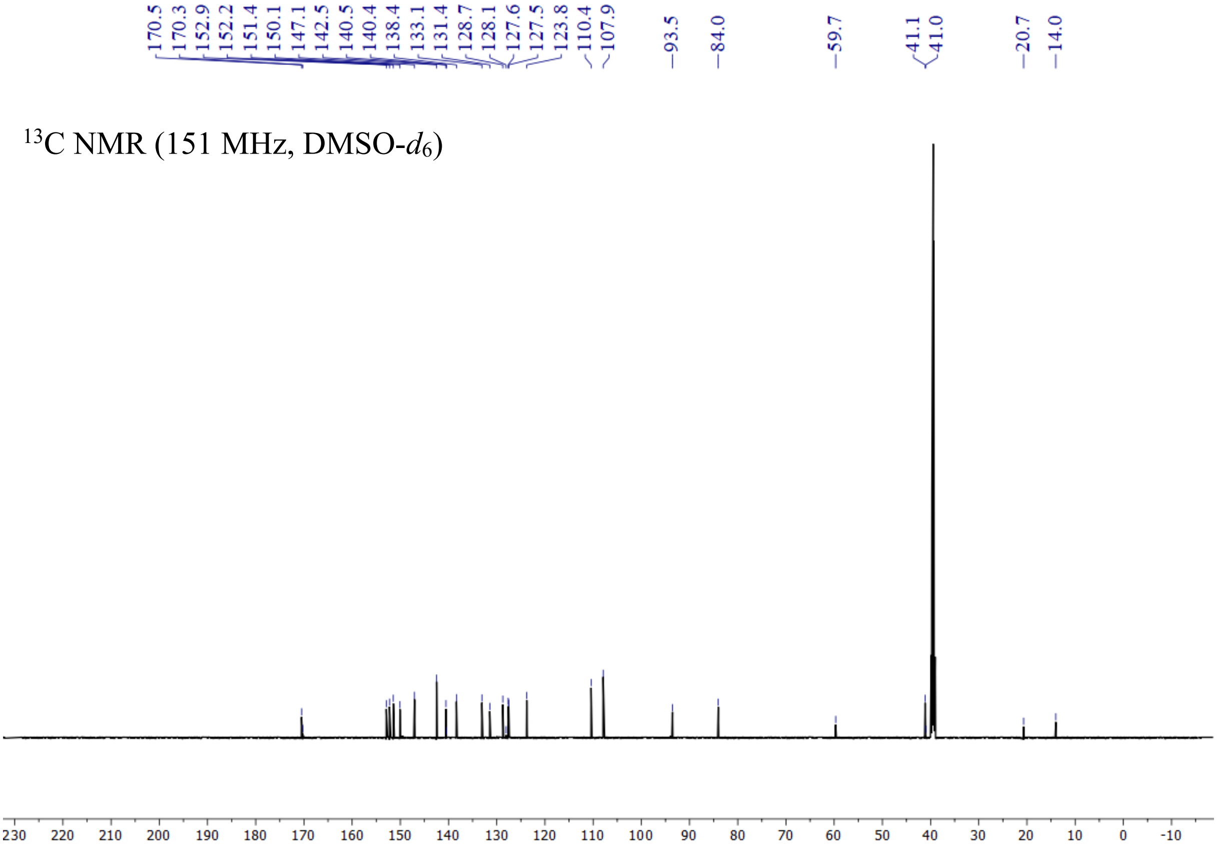

(5-{[(5-chlorothiophen-2-yl)methyl]amino}-3-(pyridin-3-yl)-1*H*-pyrazol-1-yl)(2-iodophenyl)methanone (9e)

^{1}H NMR (600 MHz, DMSO-d_6)

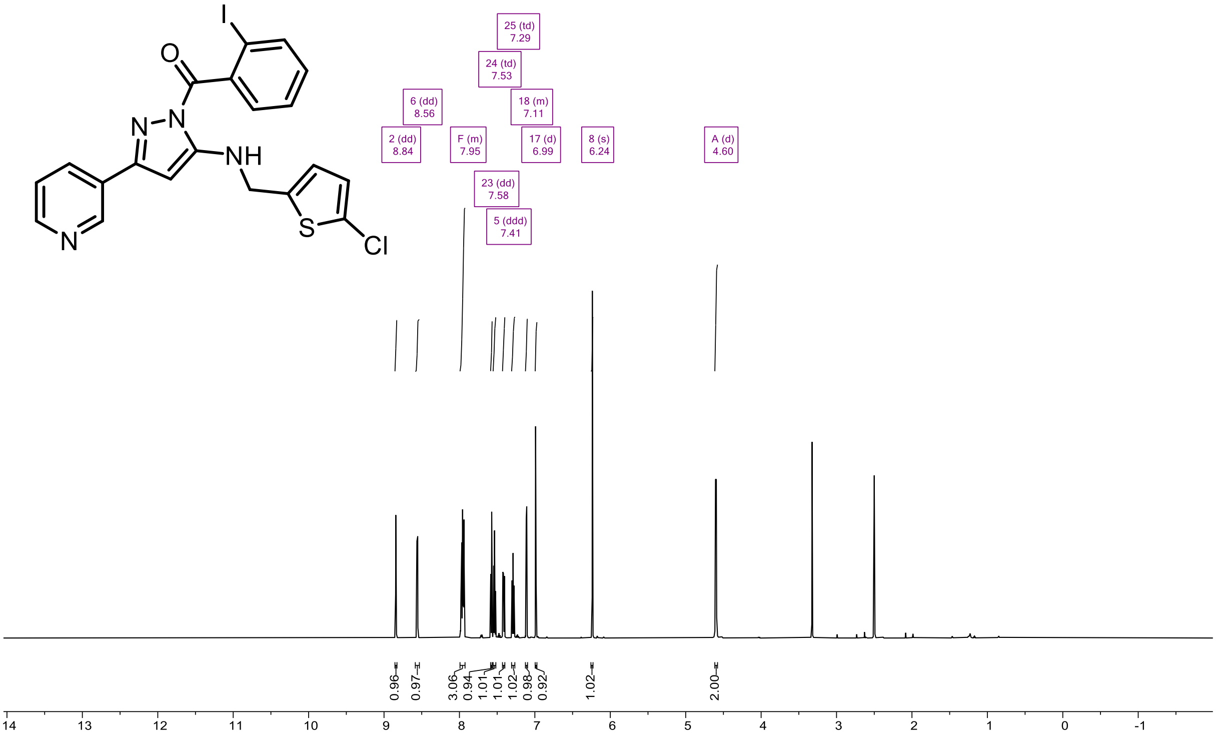

^{13}C NMR (151 MHz, DMSO-d_6)

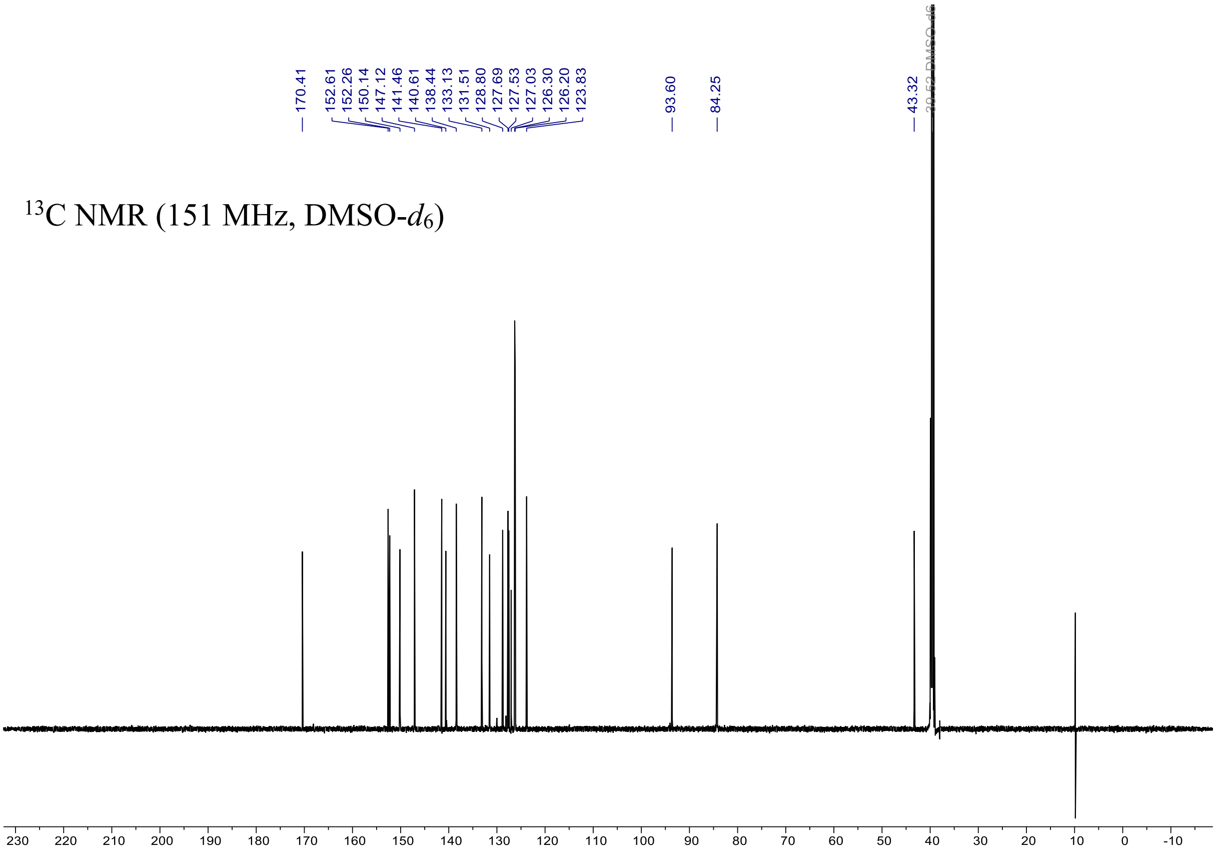

(5-((cyclohexylmethyl)amino)-3-(pyridin-3-yl)-1*H*-pyrazol-1-yl)(2-iodophenyl)methanone (9f)

^{1}H NMR (600 MHz, DMSO-d_6)

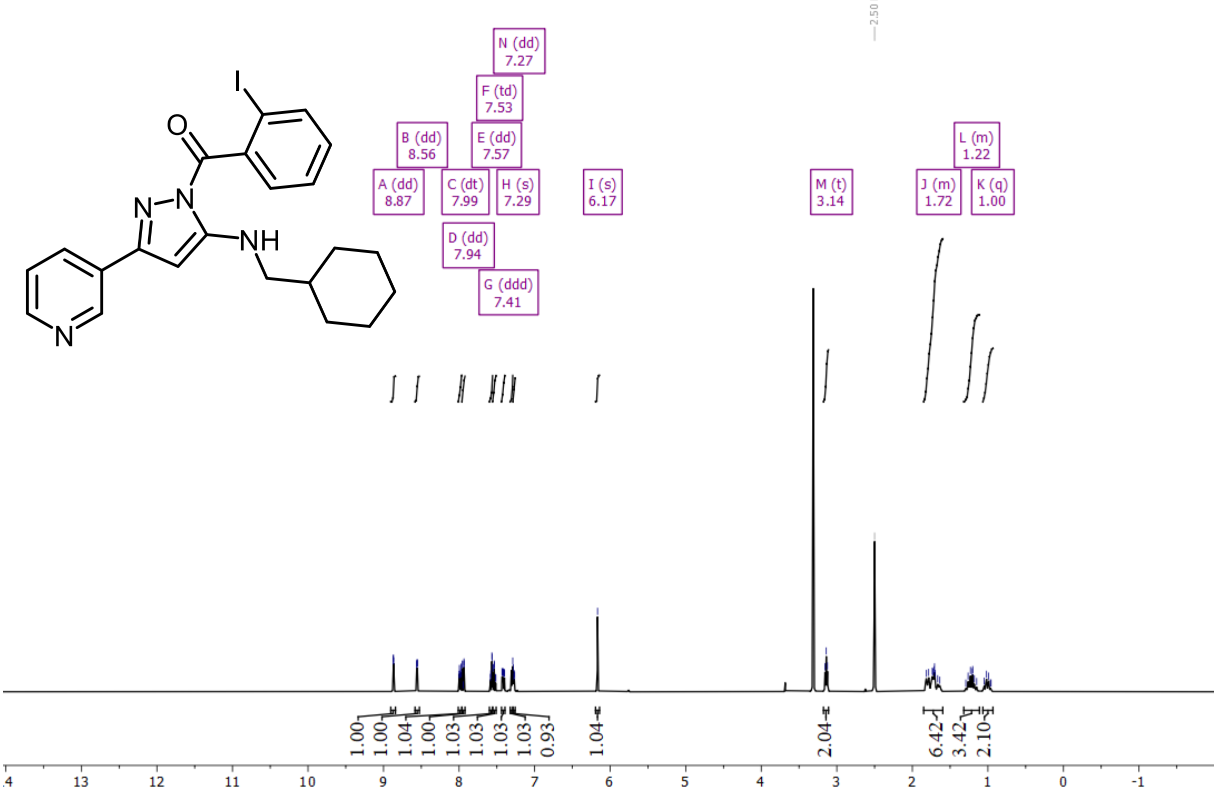

^{13}C NMR (151 MHz, DMSO-d_6)

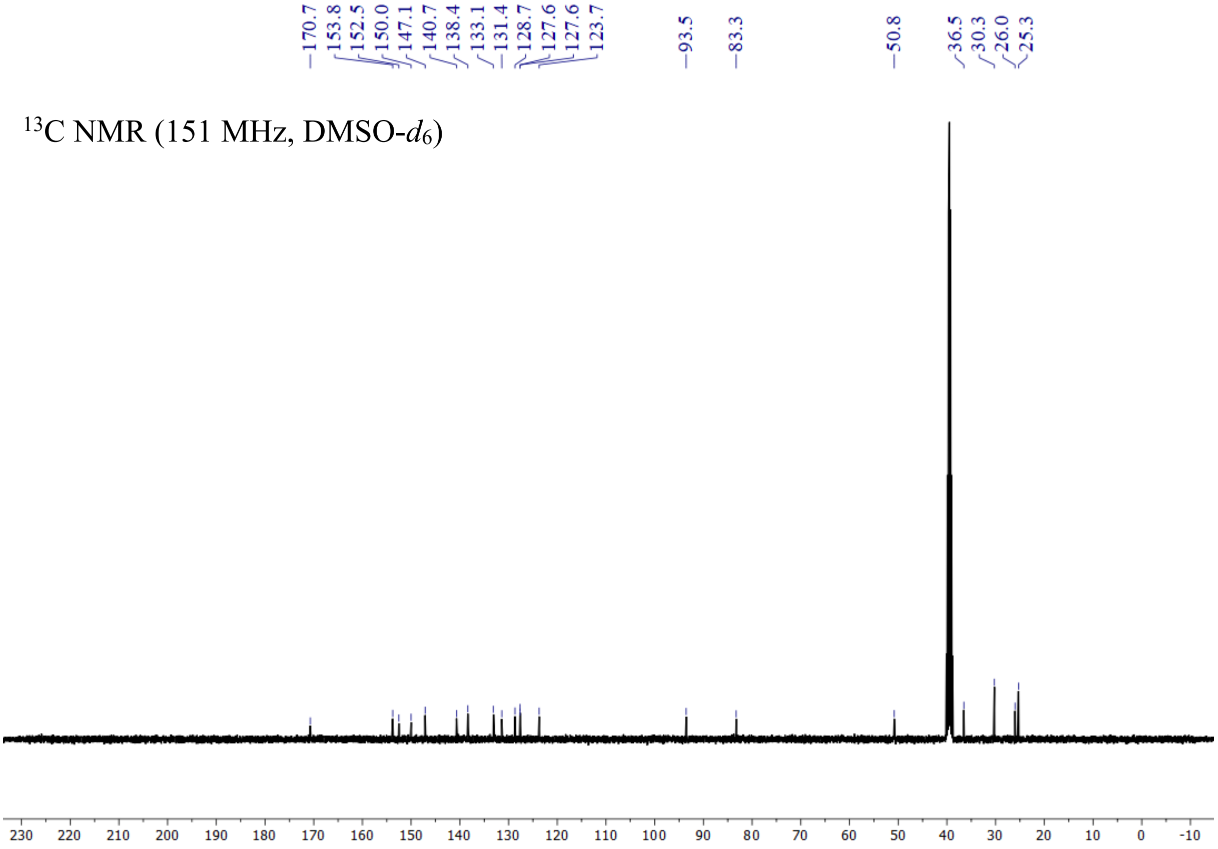

(5-(((5-chlorothiophen-2-yl)methyl)amino)-3-phenyl-1*H*-pyrazol-1-yl)(2-iodophenyl)methanone (9g)

^{1}H NMR (600 MHz, DMSO-d_6)

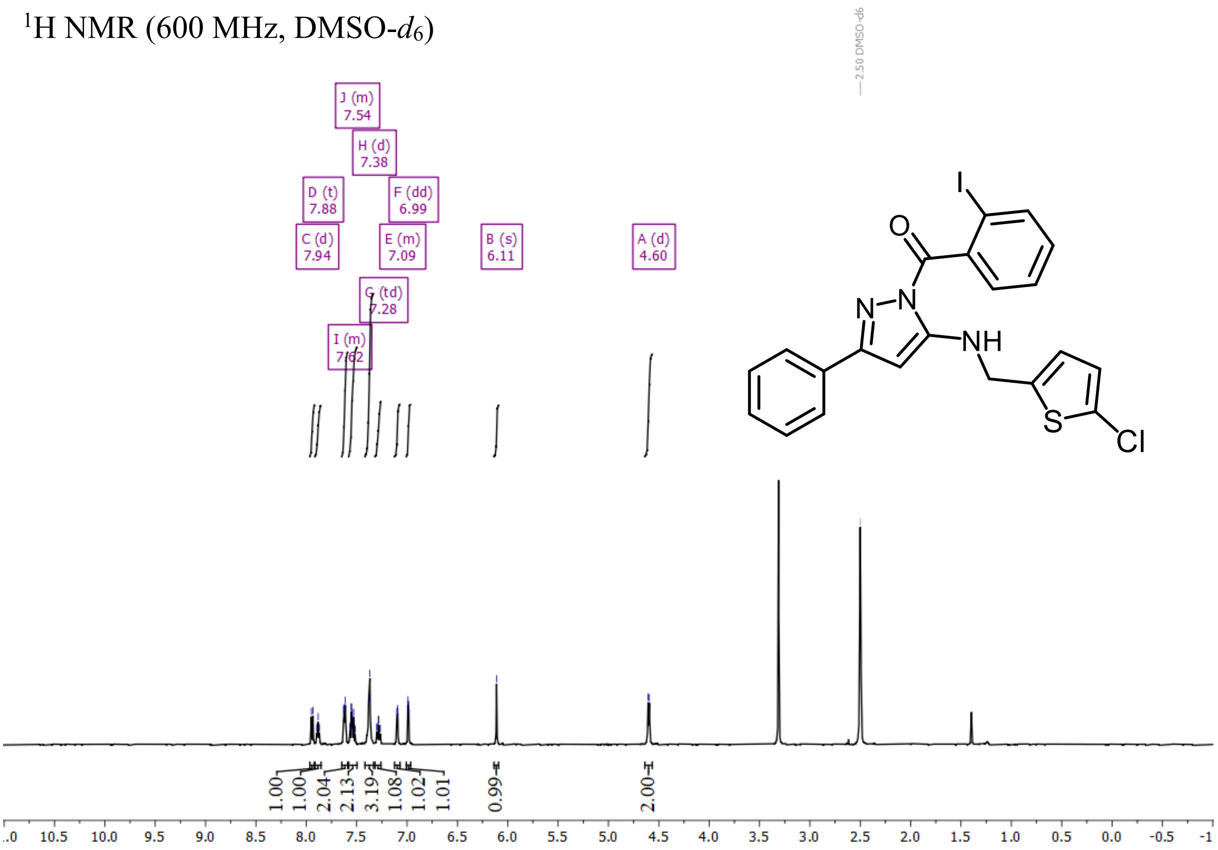

^{13}C NMR (151 MHz, DMSO-d_6)

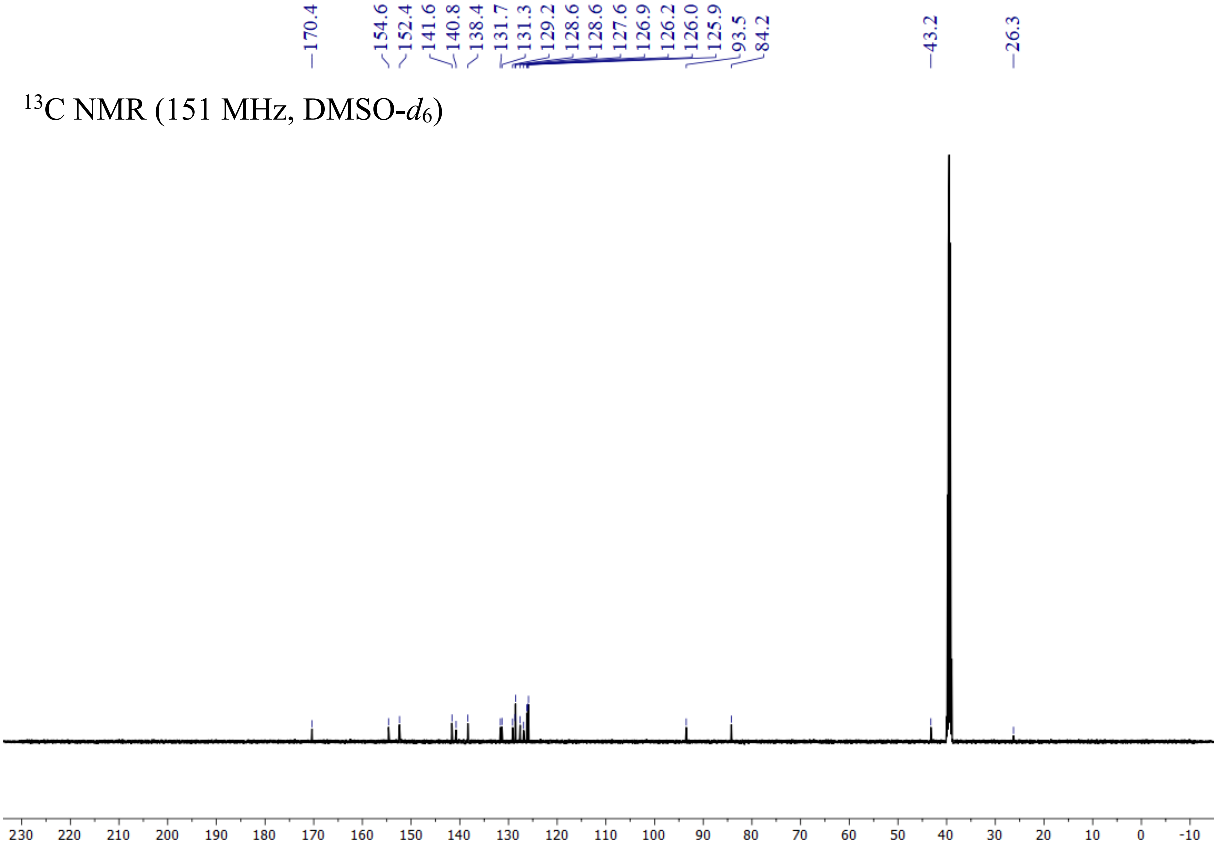

(5-(benzylamino)-3-cyclohexyl-1*H*-pyrazol-1-yl)(2-iodophenyl)methanone (9h)

^{1}H NMR (600 MHz, DMSO-d_6)

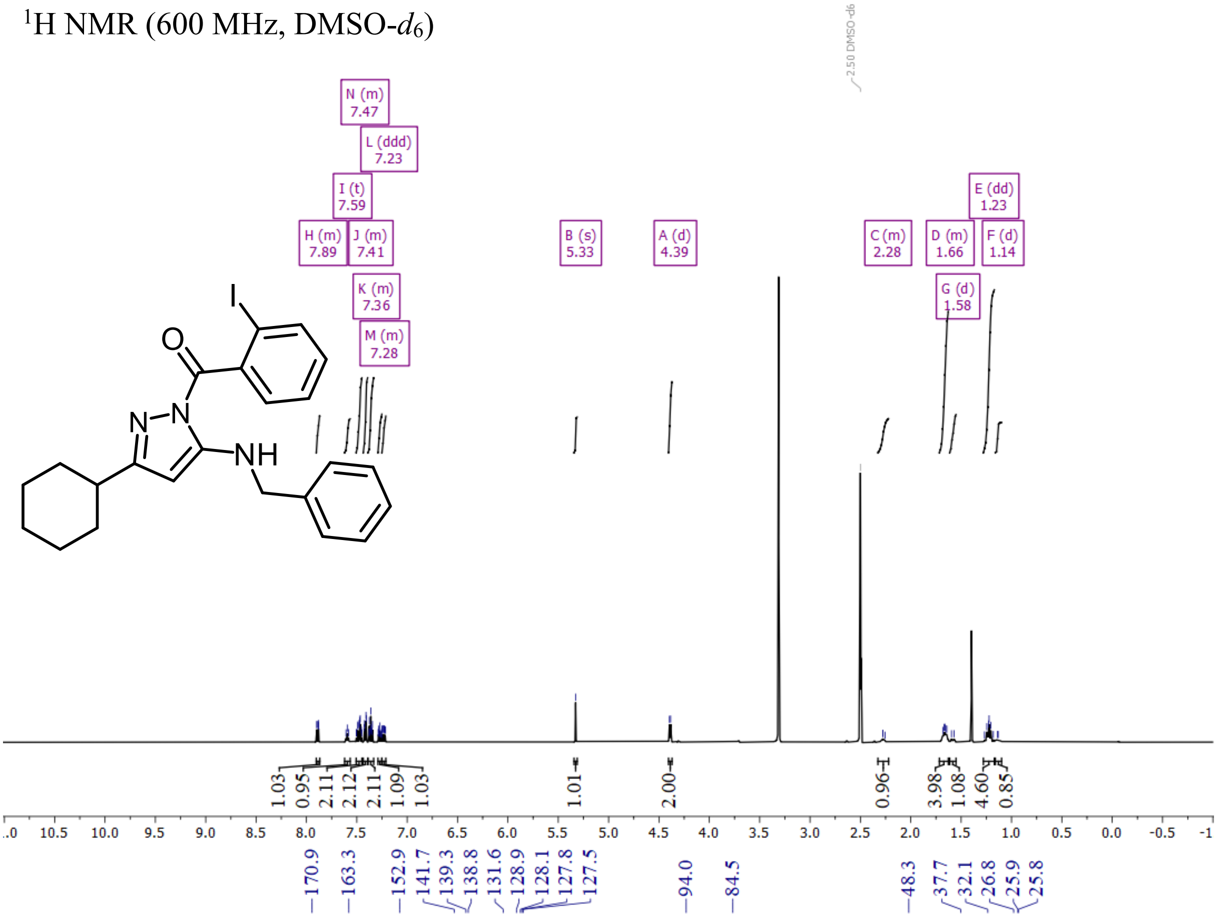

c^{13}C NMR (151 MHz, DMSO-d_6)

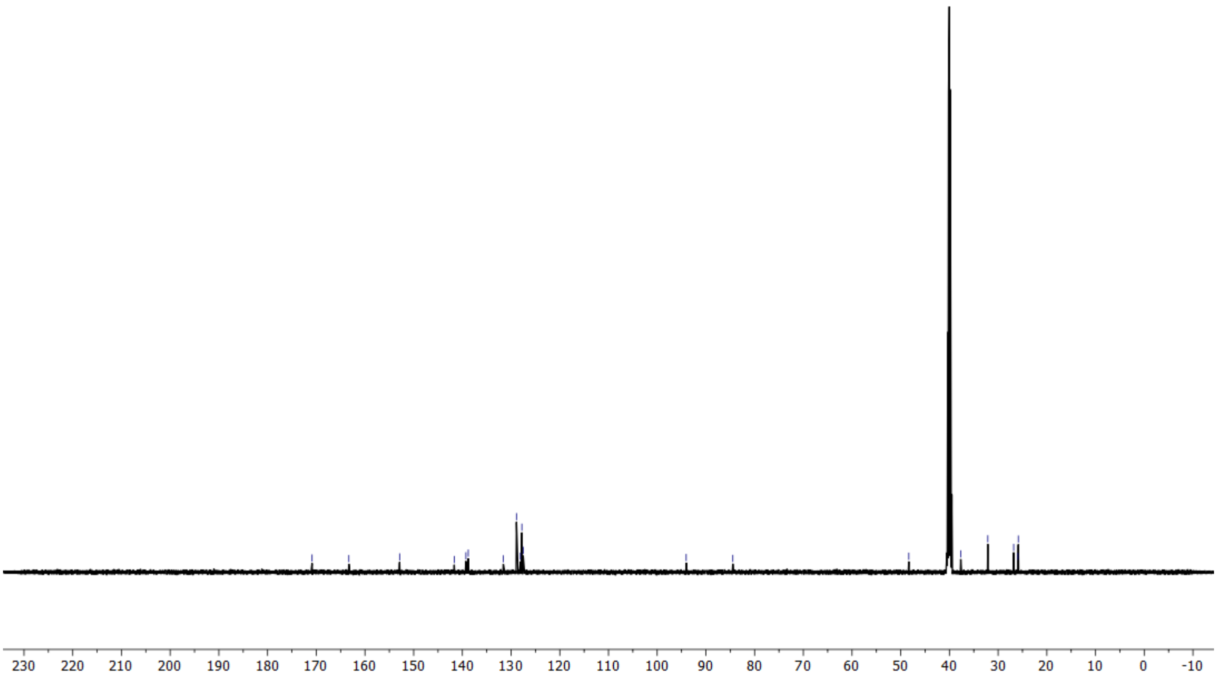

(5-(((5-chlorothiophen-2-yl)methyl)amino)-3-cyclohexyl-1*H*-pyrazol-1-yl)(2-iodophenyl)methanone (9i)

^{1}H NMR (600 MHz, DMSO-d_6)

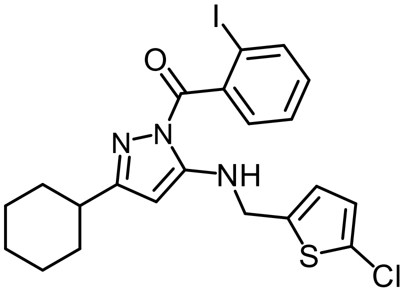

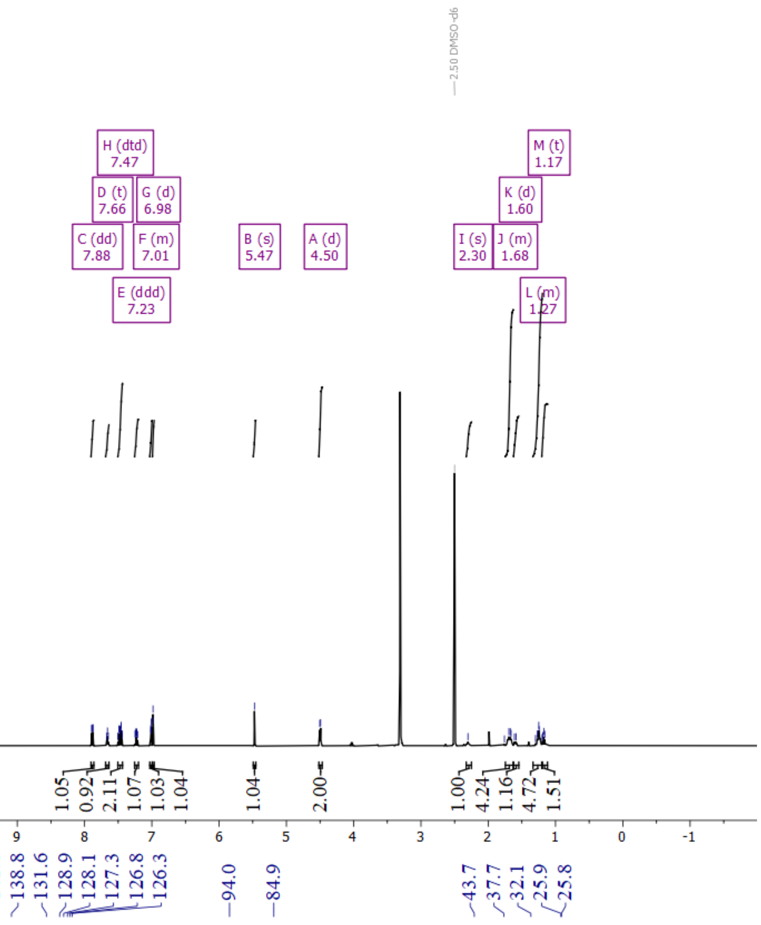

^{1}H NMR (600 MHz, DMSO-d_6)

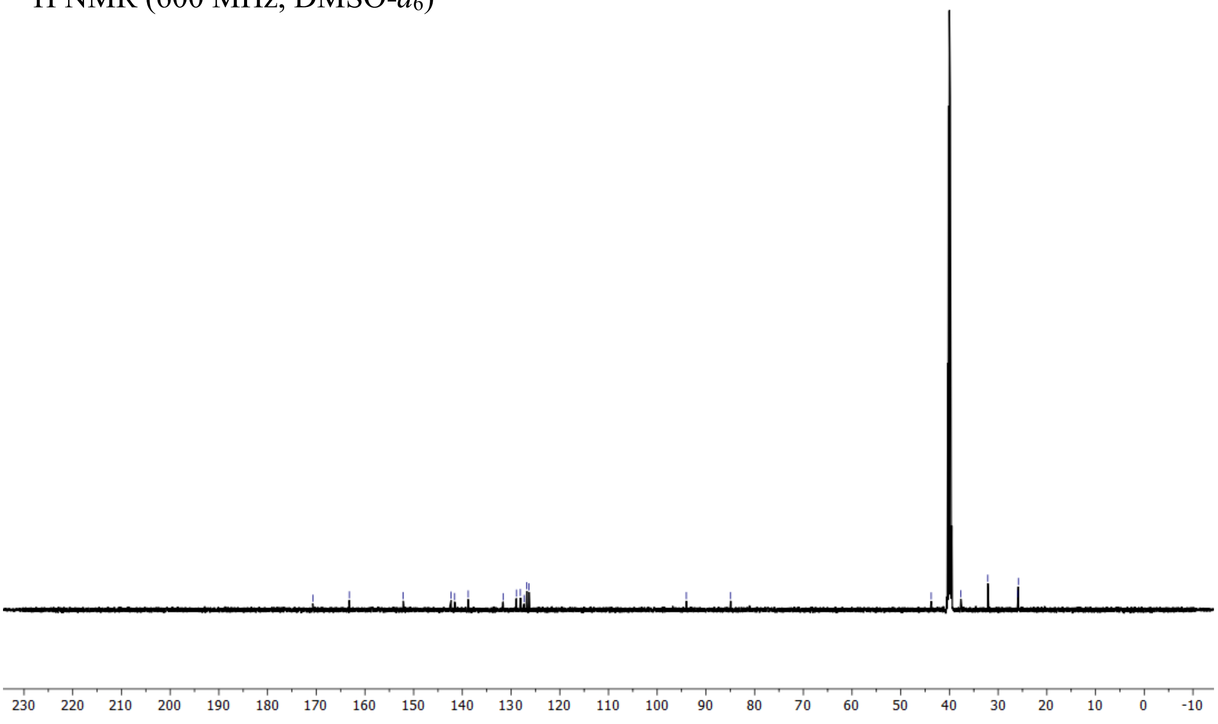

4-benzyl-2-(pyridin-3-yl)pyrazolo[5,1-*b*]quinazolin-9(4*H*)-one (10a)

^{1}H NMR (600 MHz, DMSO-d_6)

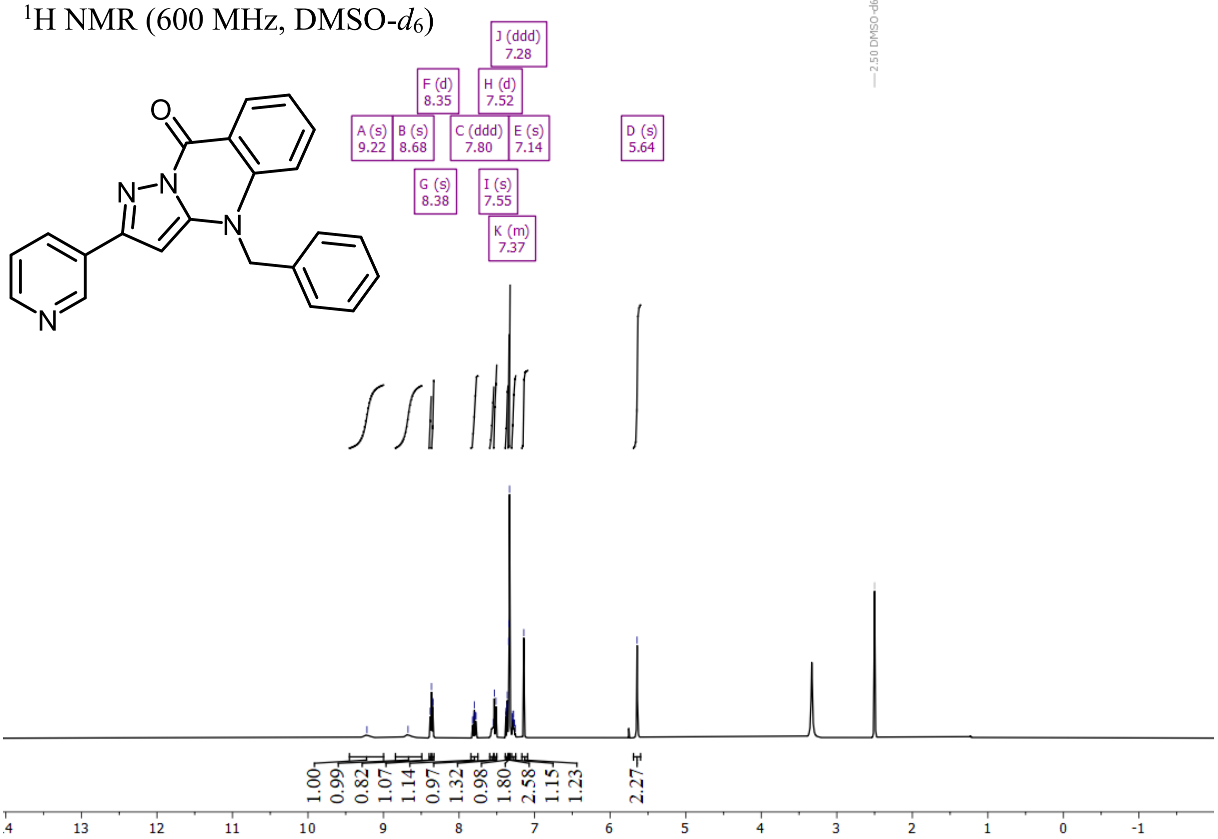

^{1}H NMR (600 MHz, DMSO-d_6)

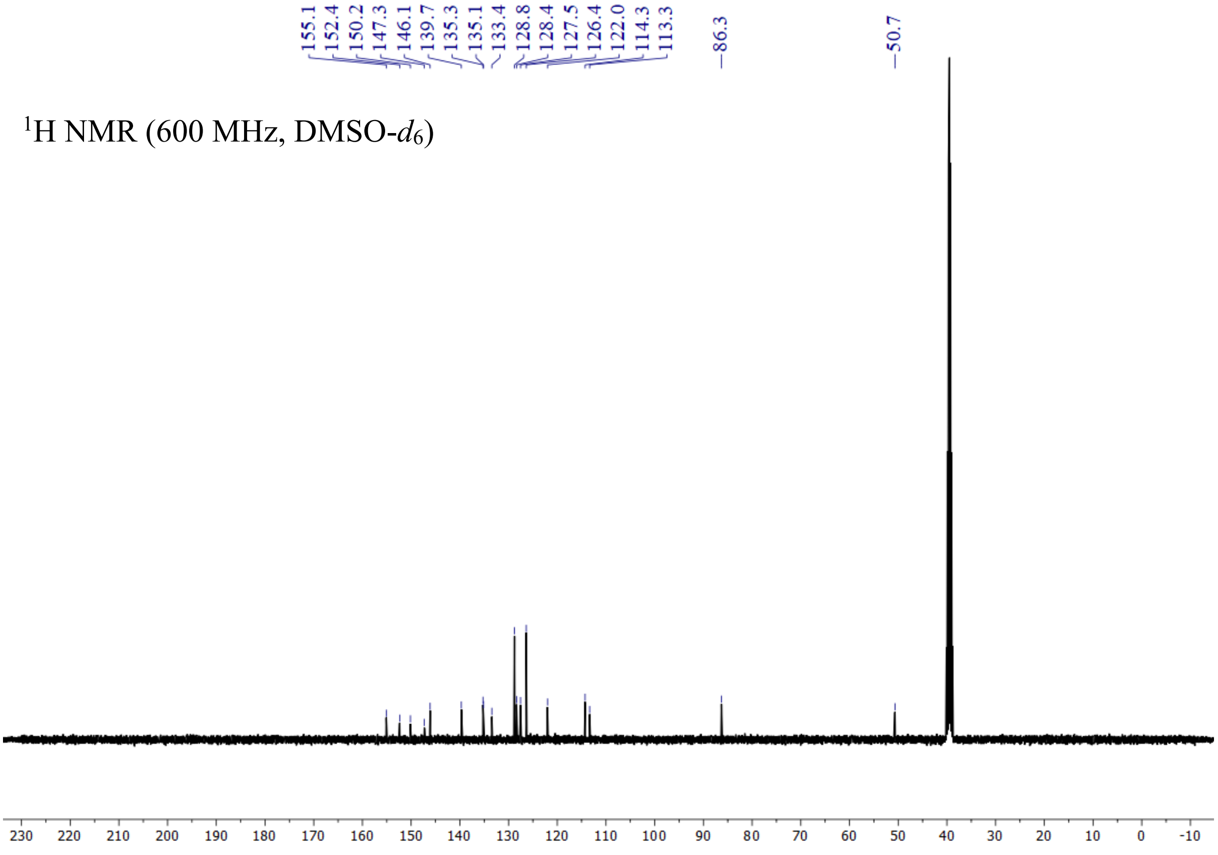

4-(4-methoxybenzyl)-2-(pyridin-3-yl)pyrazolo[5,1-*b*]quinazolin-9(4*H*)-one (10b)

^{1}H NMR (600 MHz, DMSO-*d*$_6$)

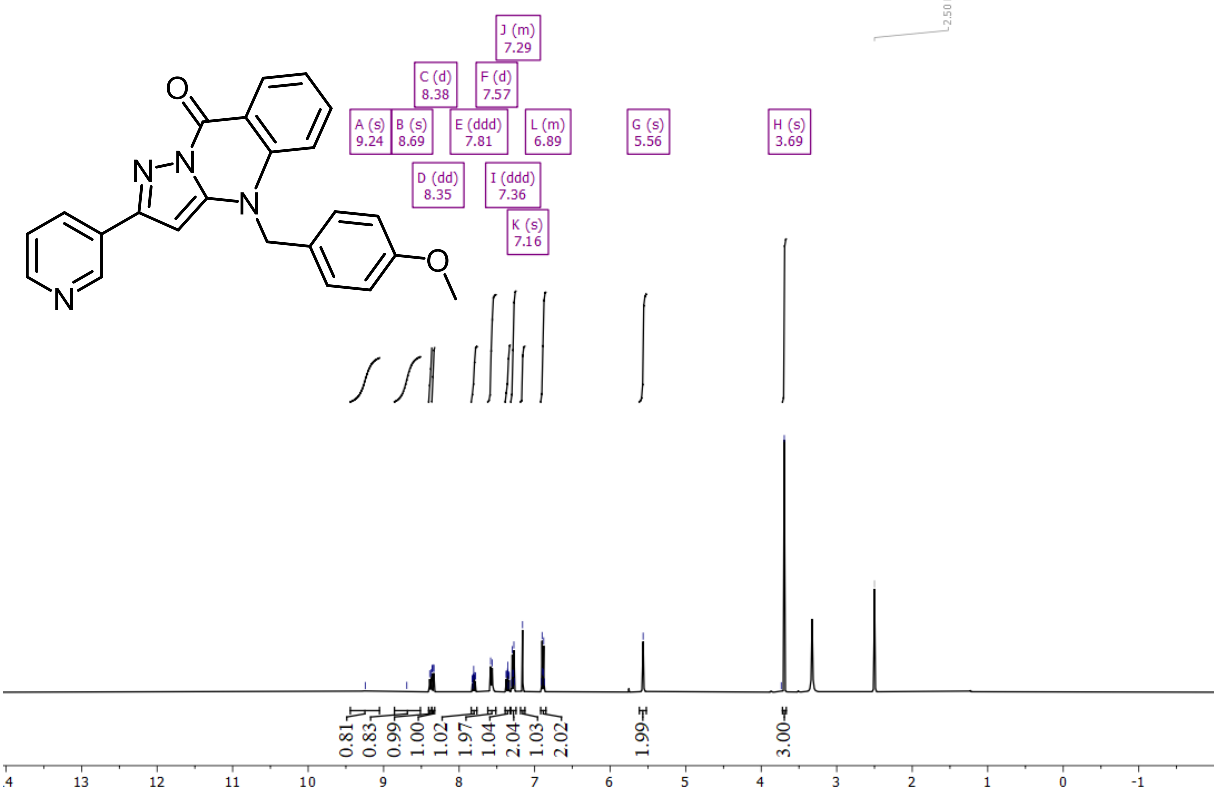

^{1}H NMR (600 MHz, DMSO-*d*$_6$)

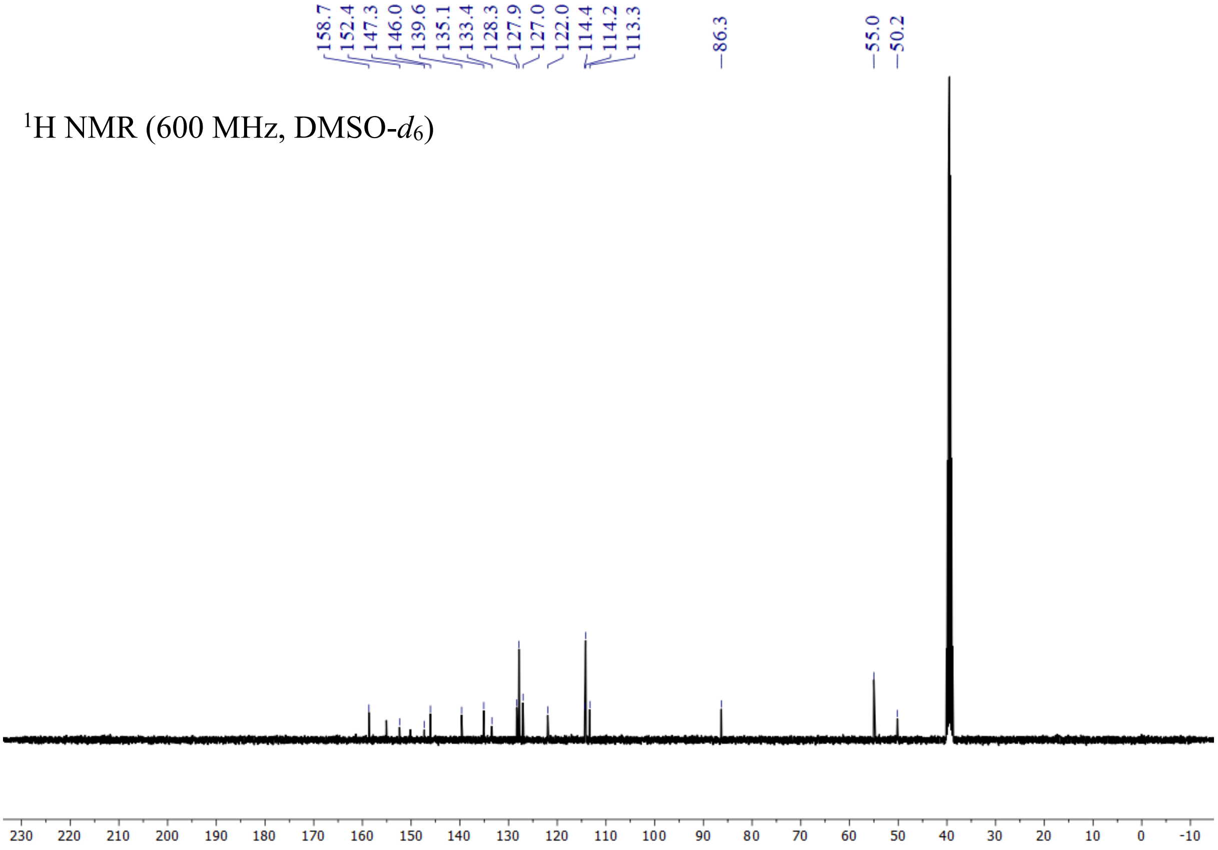

4-(naphthalen-1-ylmethyl)-2-(pyridin-3-yl)pyrazolo[5,1-*b*]quinazolin-9(4*H*)-one (10c)

^{1}H NMR (600 MHz, DMSO-d_6)

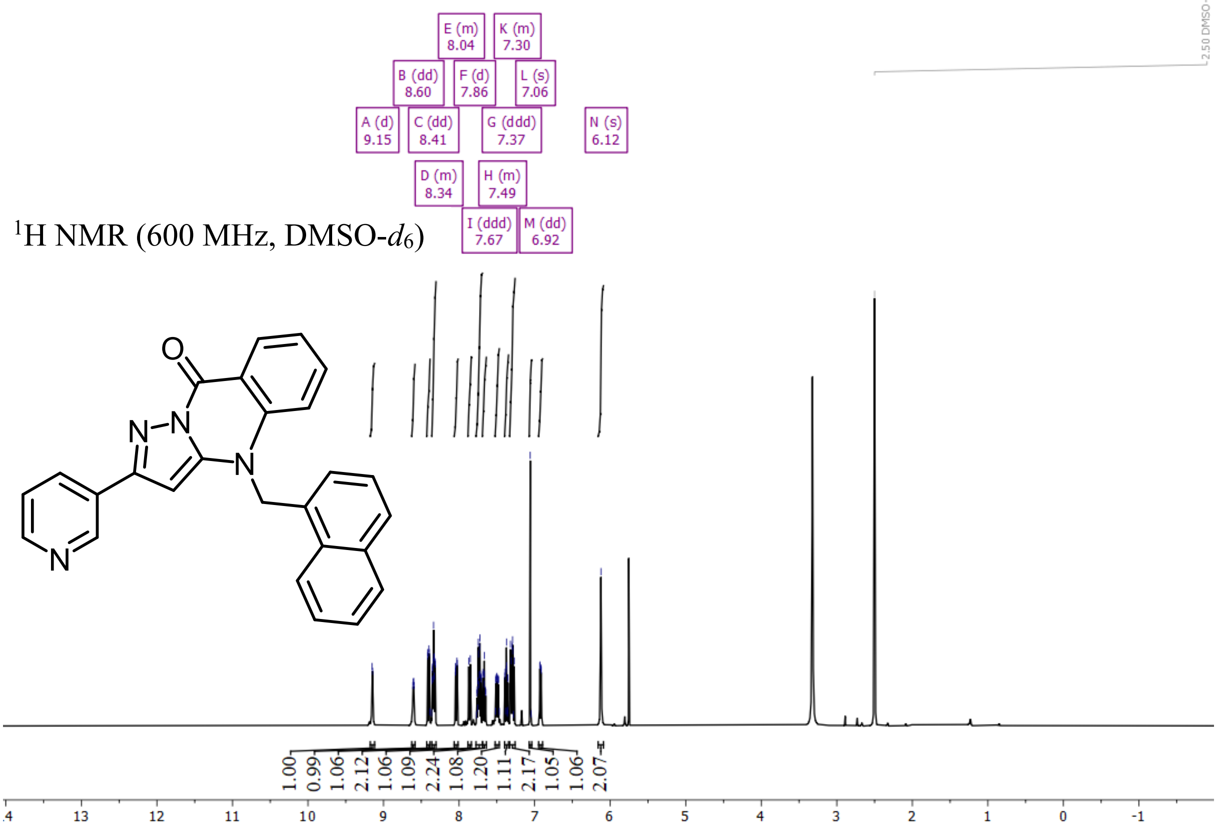

^{1}H NMR (600 MHz, DMSO-d_6)

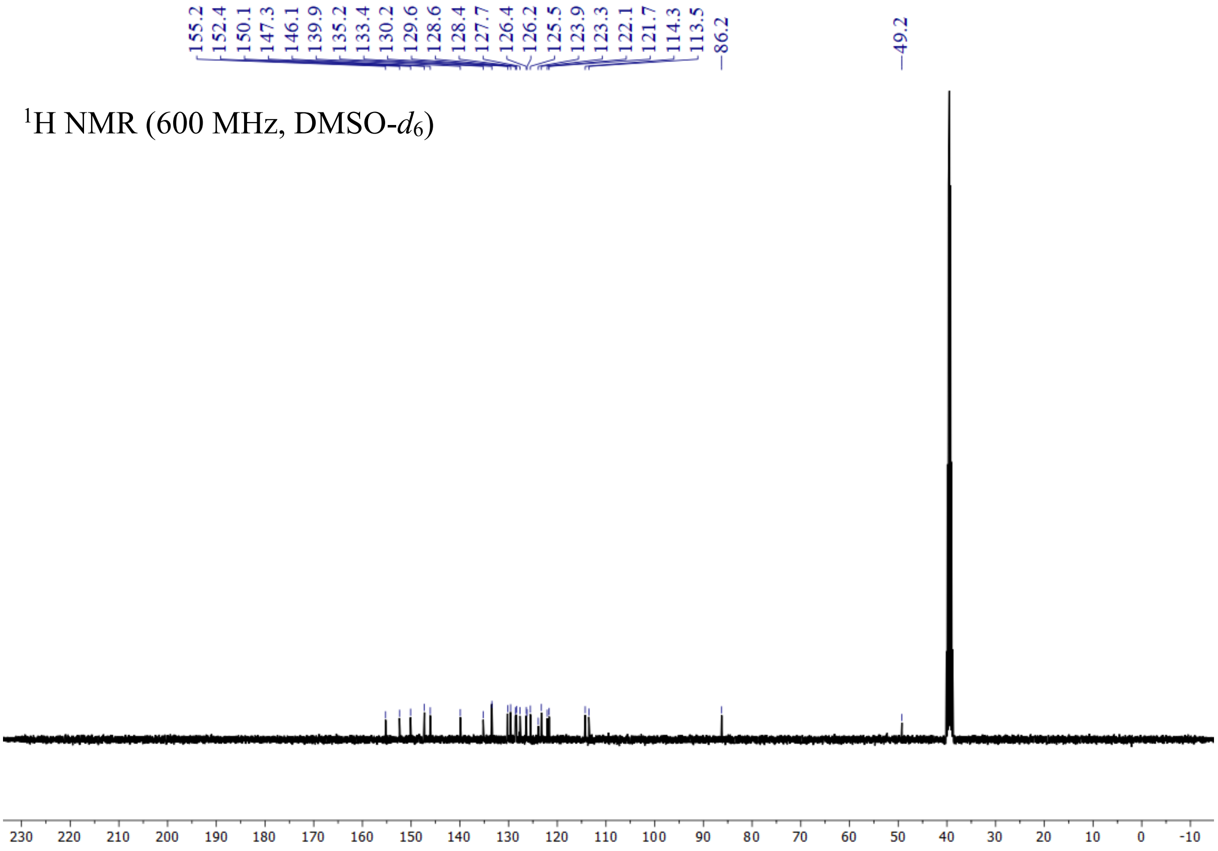

4-(furan-2-ylmethyl)-2-(pyridin-3-yl)pyrazolo[5,1-*b*]quinazolin-9(4*H*)-one (10d)

^{1}H NMR (600 MHz, DMSO-d_6)

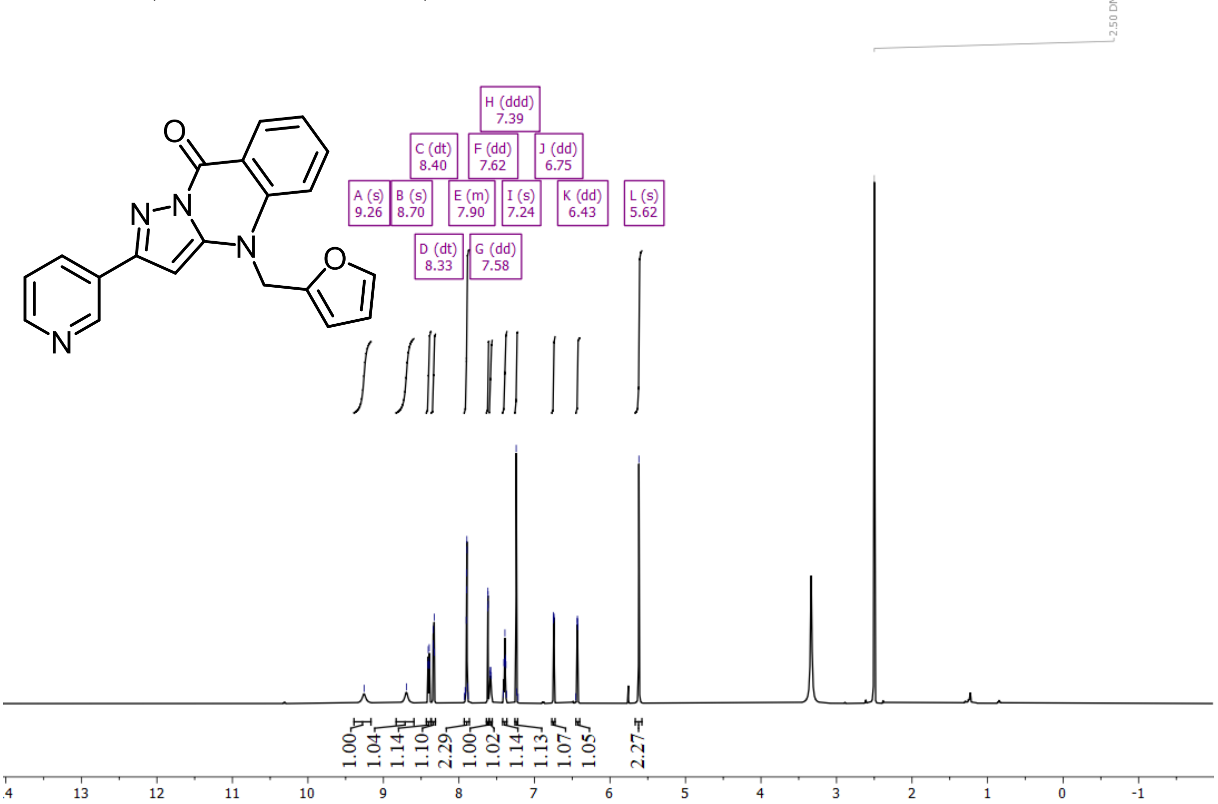

^{13}C NMR (151 MHz, DMSO-d_6)

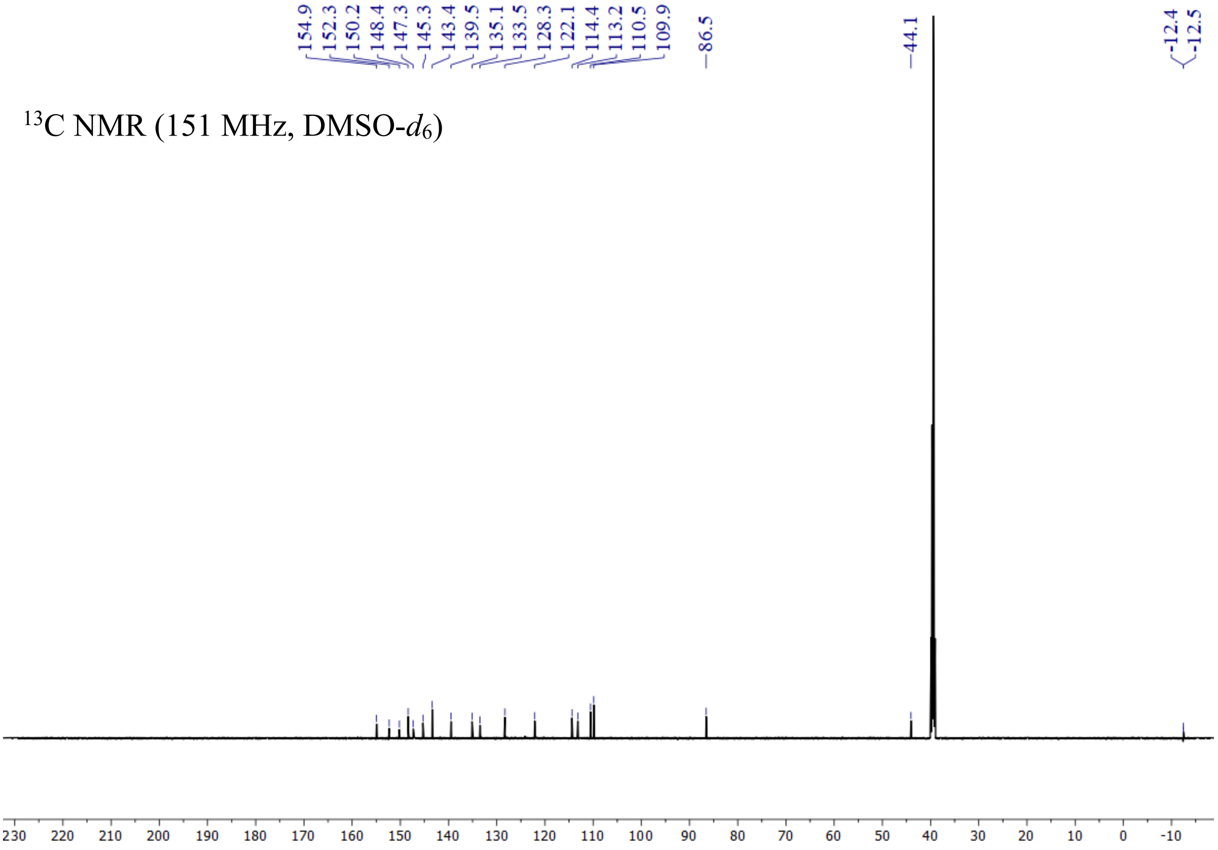

4-((5-chlorothiophen-2-yl)methyl)-2-(pyridin-3-yl)pyrazolo[5,1-*b*]quinazolin-9(4*H*)-one (10e)

^{1}H NMR (600 MHz, DMSO-d_6)

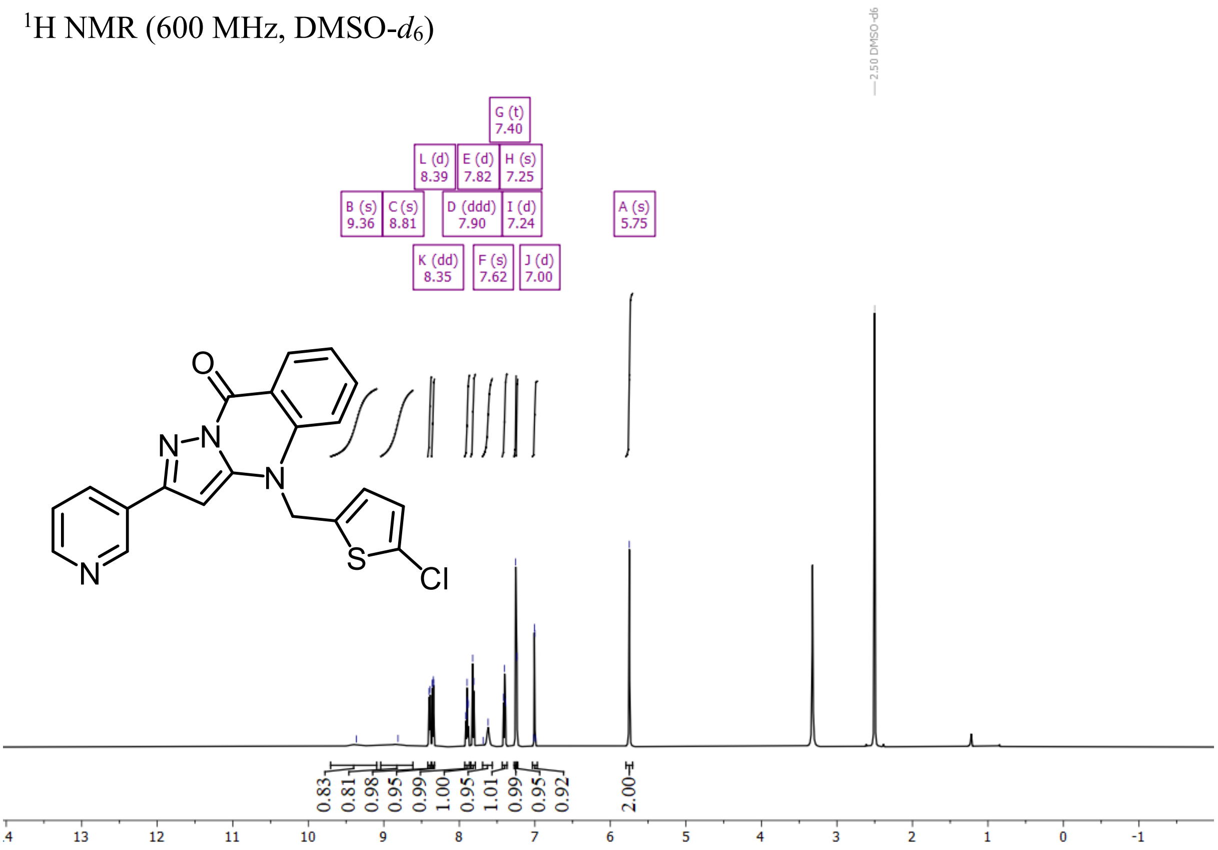

^{13}C NMR (151 MHz, DMSO-d_6)

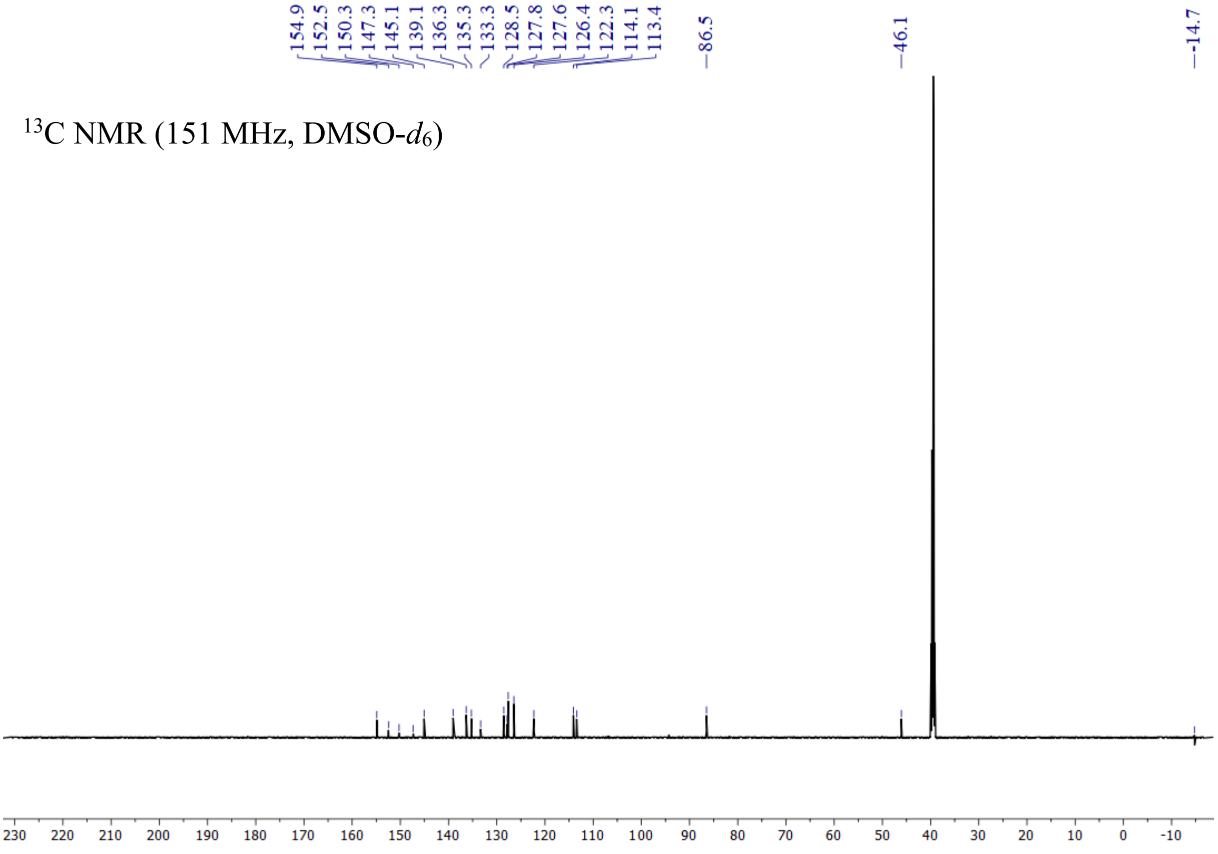

4-(cyclohexylmethyl)-2-(pyridin-3-yl)pyrazolo[5,1-*b*]quinazolin-9(4*H*)-one (10f)

^{1}H NMR (600 MHz, DMSO-d_6)

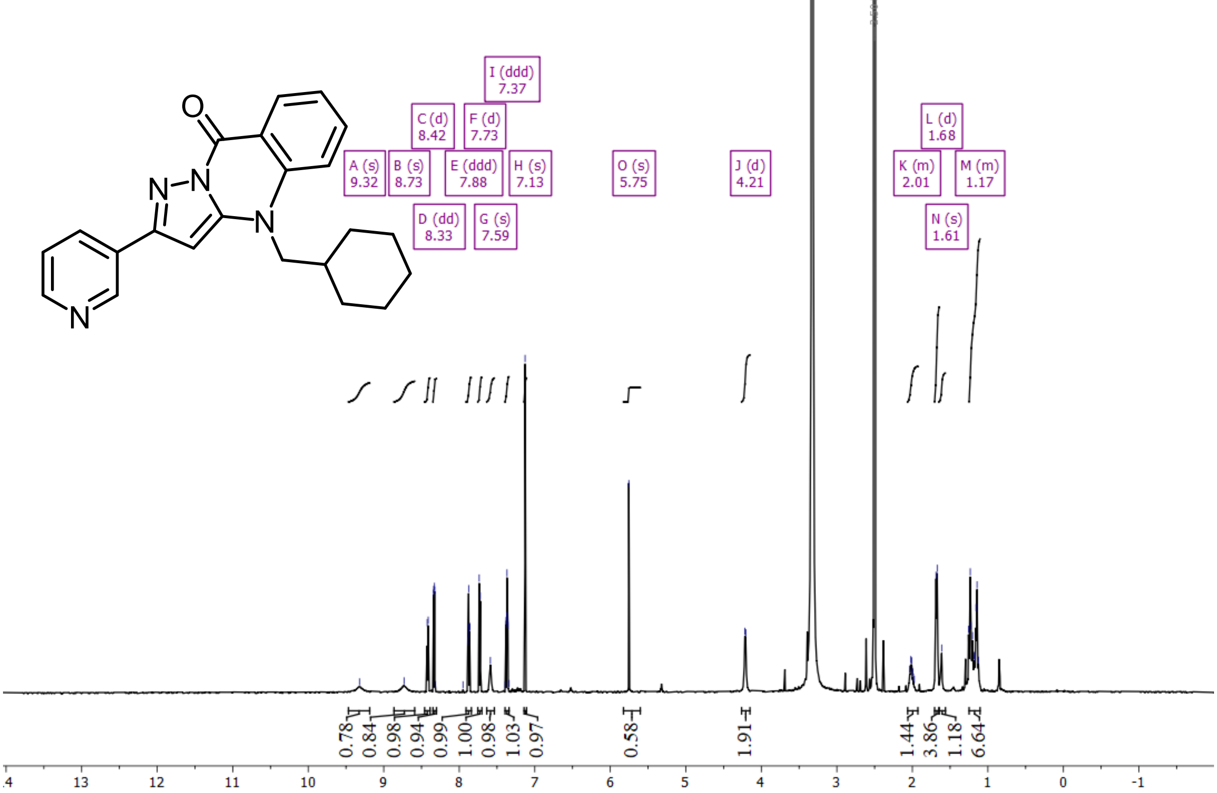

4-((5-chlorothiophen-2-yl)methyl)-2-phenylpyrazolo[5,1-*b*]quinazolin-9(4*H*)-one (10g)

^{1}H NMR (600 MHz, DMSO-d_6)

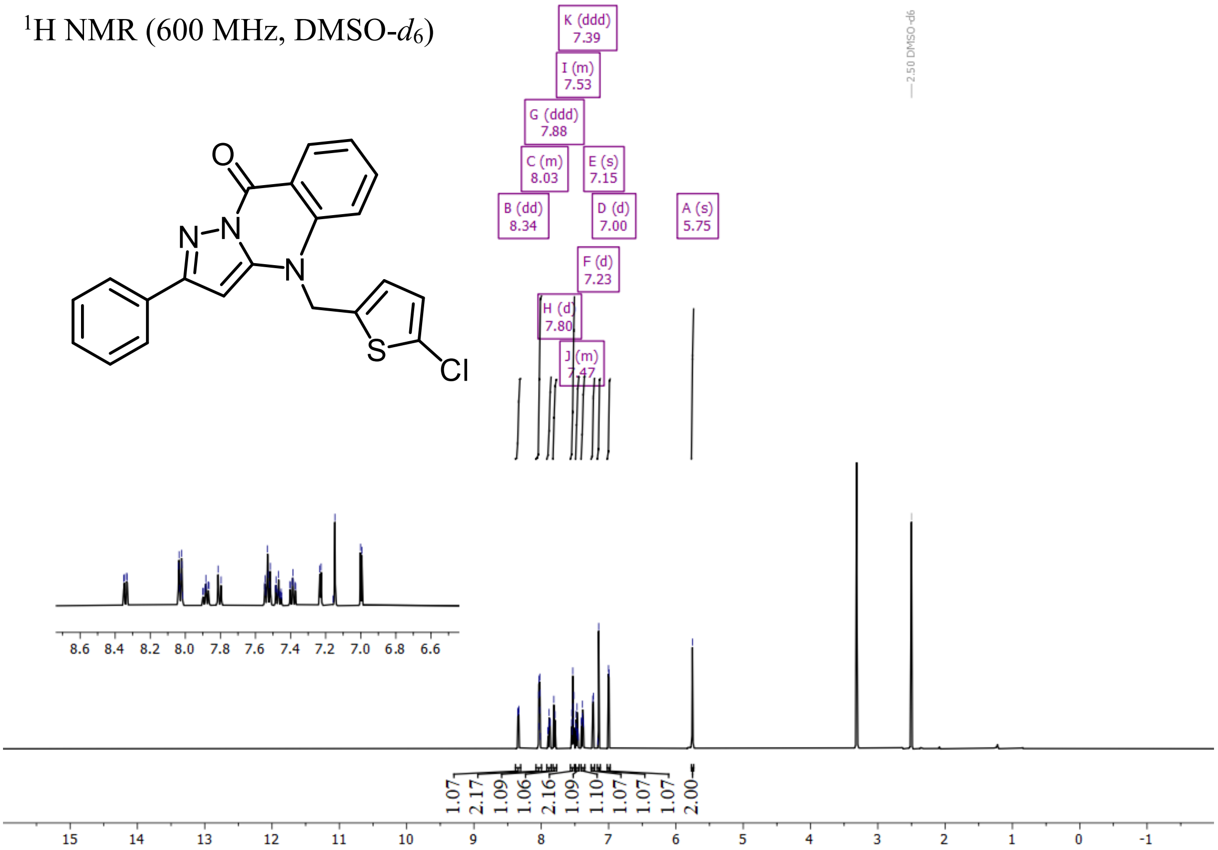

^{13}C NMR (151 MHz, DMSO-d_6)

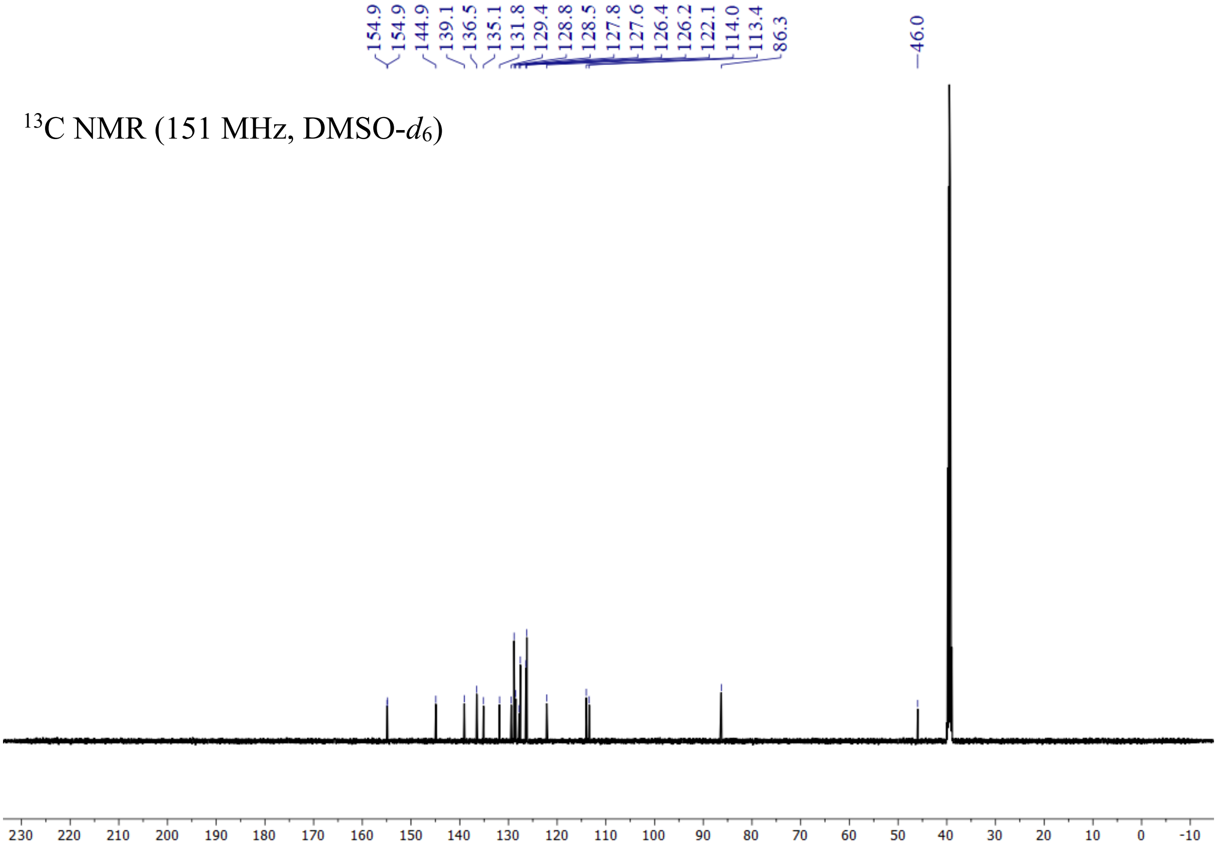

4-benzyl-2-cyclohexylpyrazolo[5,1-*b*]quinazolin-9(4*H*)-one (10h)

^{1}H NMR (600 MHz, DMSO-d_6)

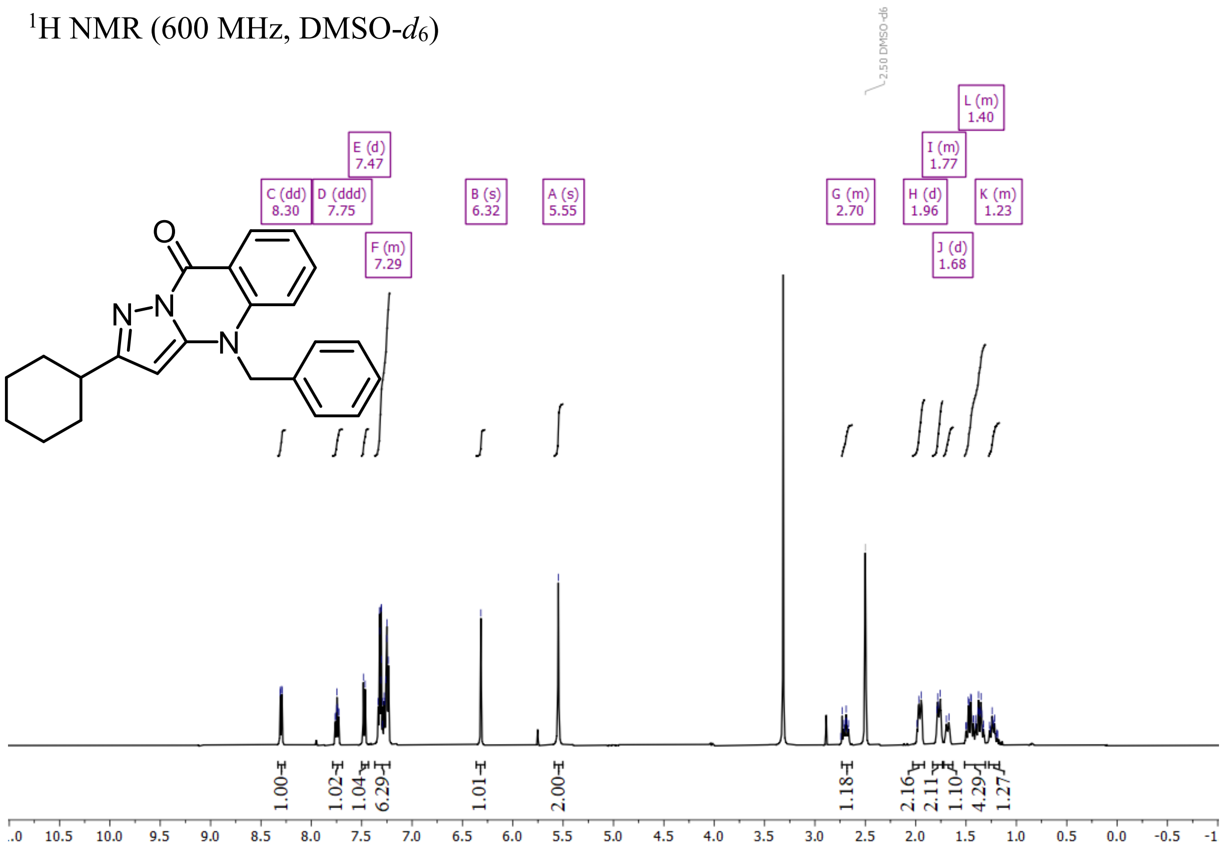

^{13}C NMR (151 MHz, DMSO-d_6)

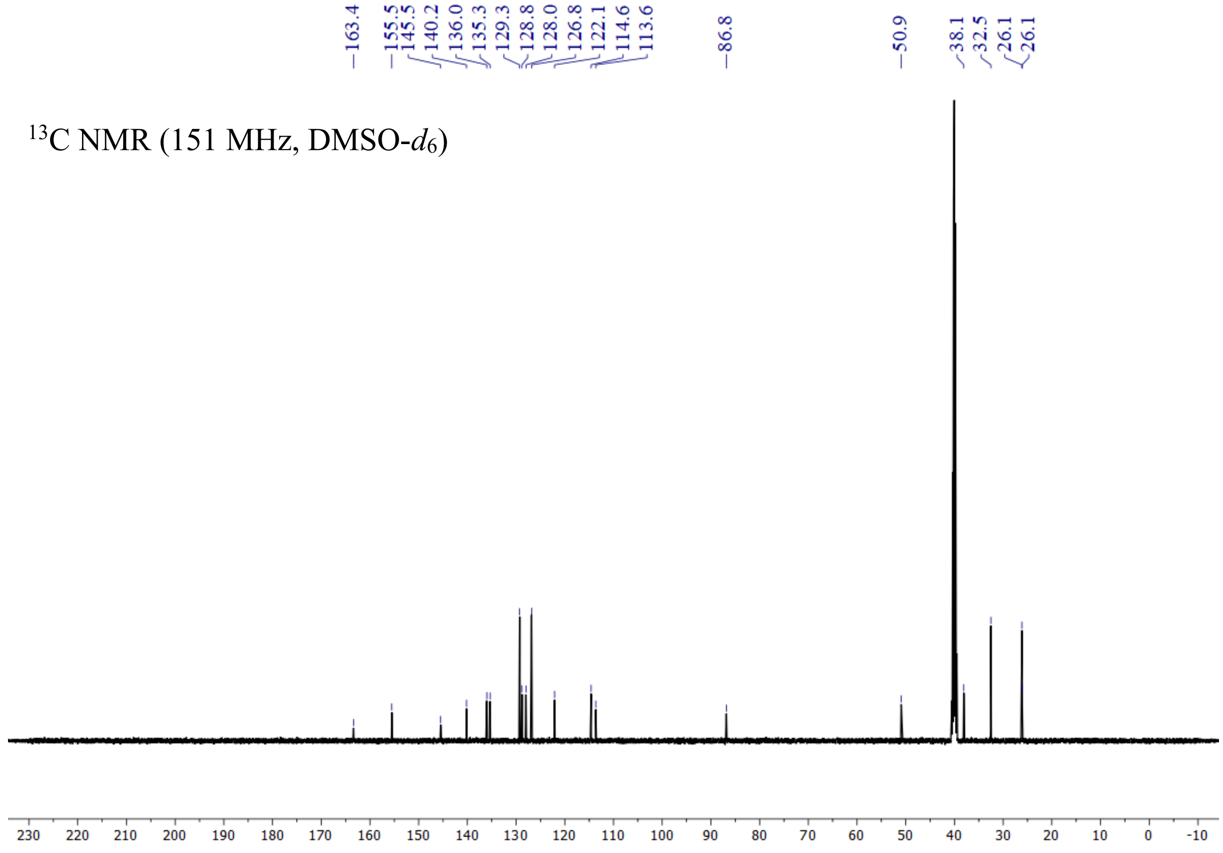

4-((5-chlorothiophen-2-yl)methyl)-2-cyclohexylpyrazolo[5,1-*b*]quinazolin-9(4*H*)-one (10i)

^{1}H NMR (600 MHz, DMSO-d_6)

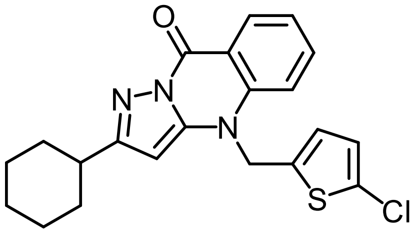

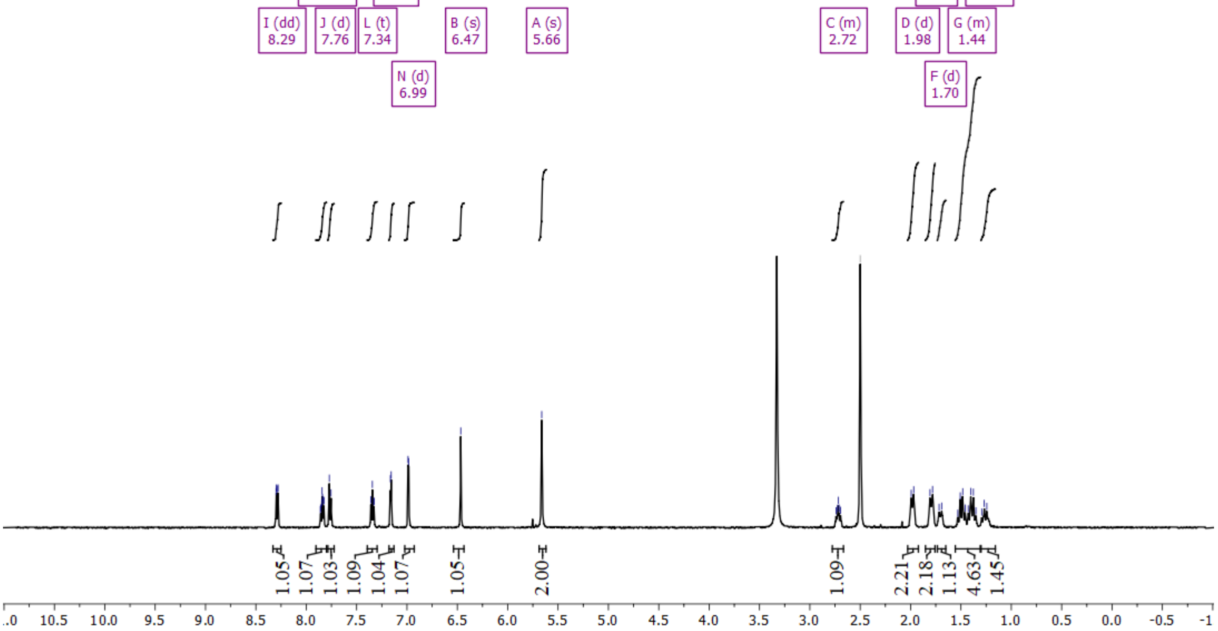

^{13}C NMR (151 MHz, DMSO-d_6)

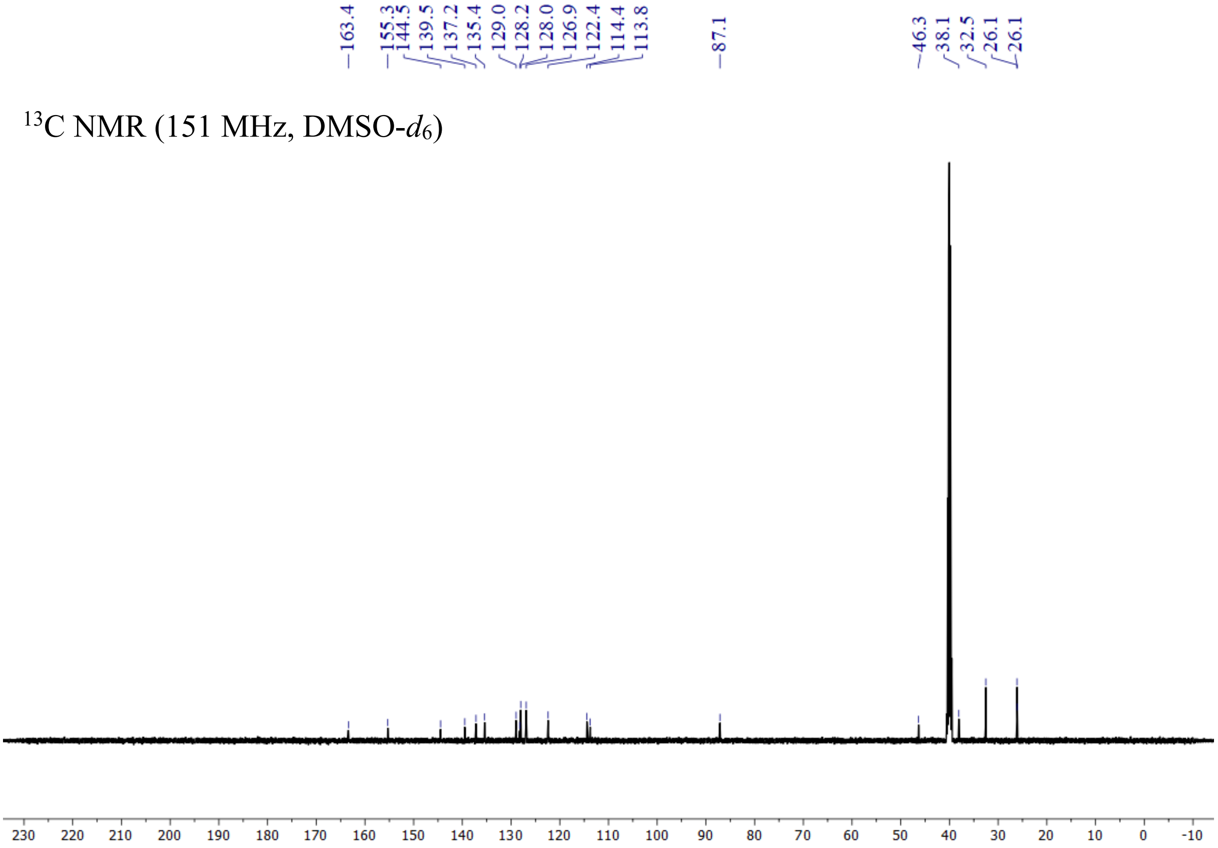

2-iodo-*N*-(3-(pyridin-3-yl)-1*H*-pyrazol-5-yl)benzamide (14)

^{1}H NMR (600 MHz, DMSO-d_6)

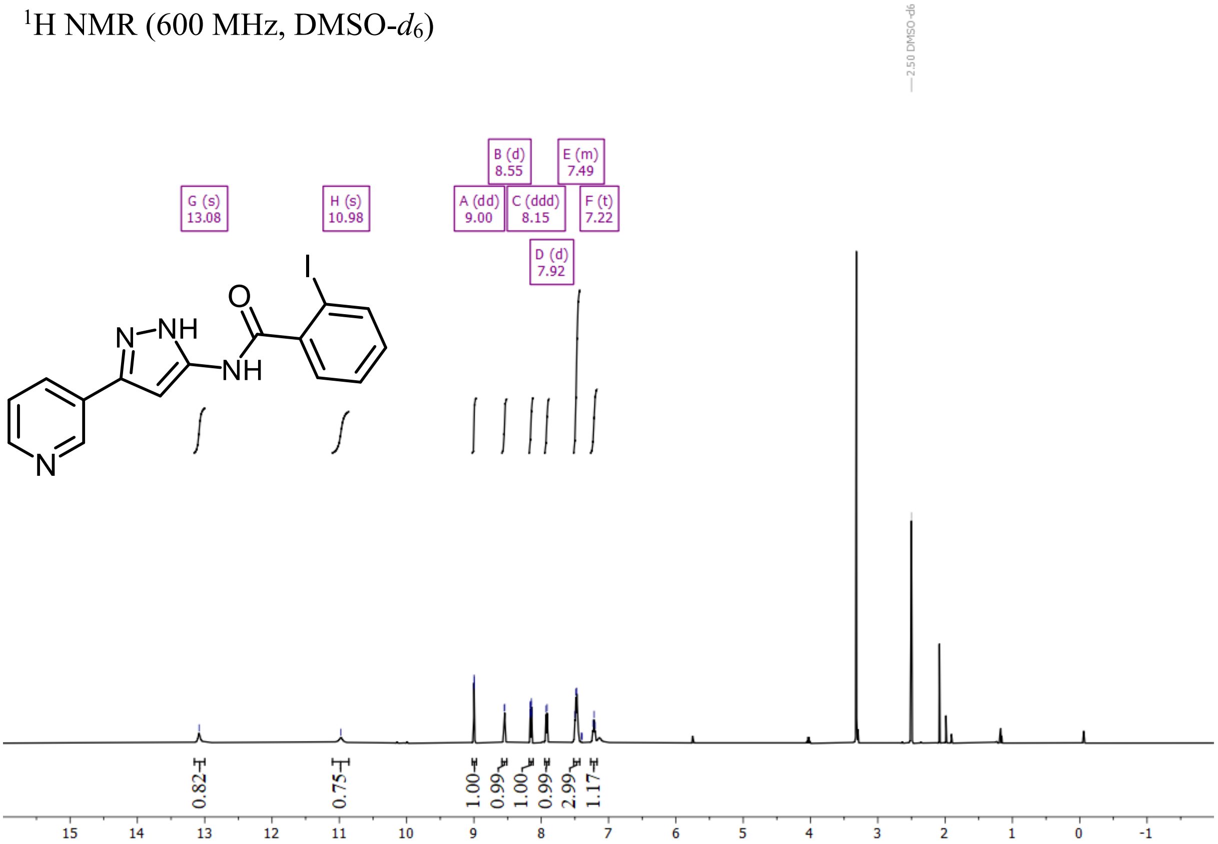

^{13}C NMR (151 MHz, DMSO-d_6)

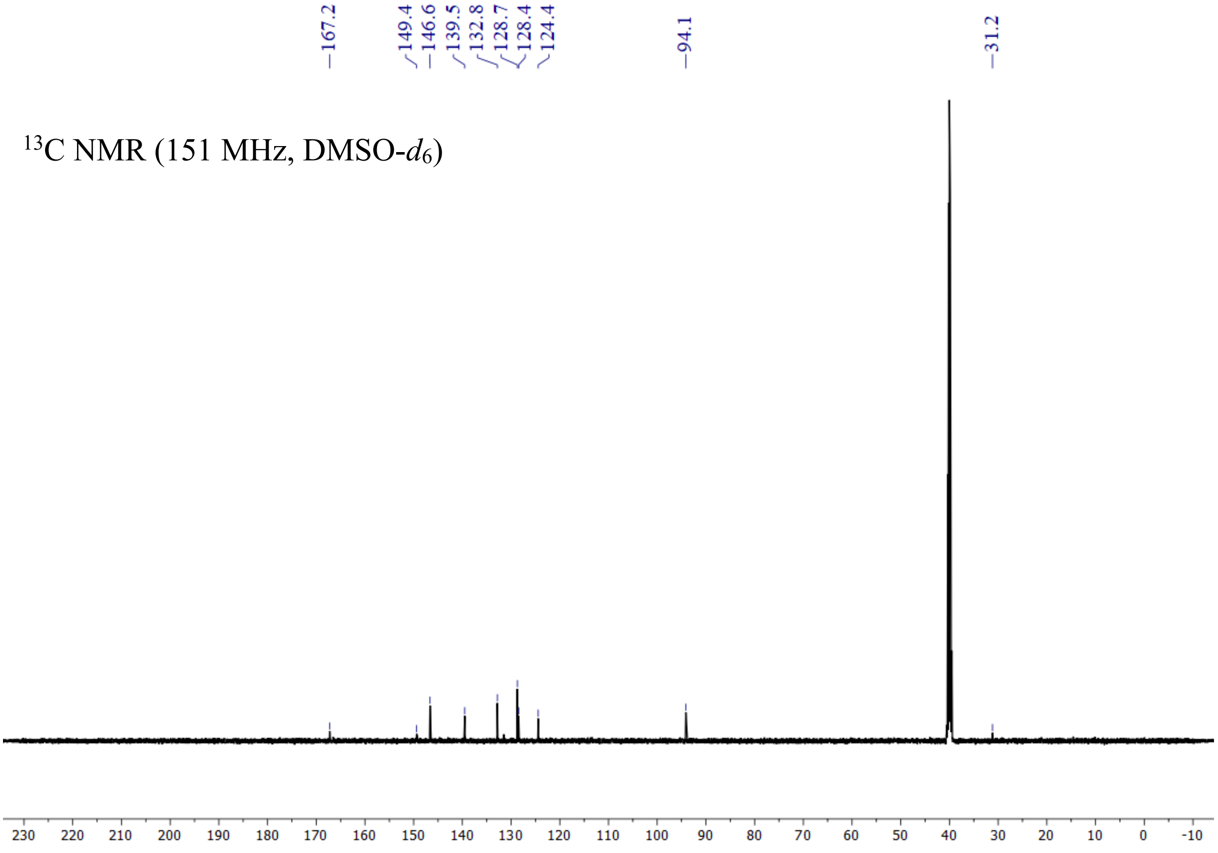

2-(pyridin-3-yl)pyrazolo[1,5-*a*]quinazolin-5(4*H*)-one (15)

^{1}H NMR (600 MHz, DMSO-d_6)

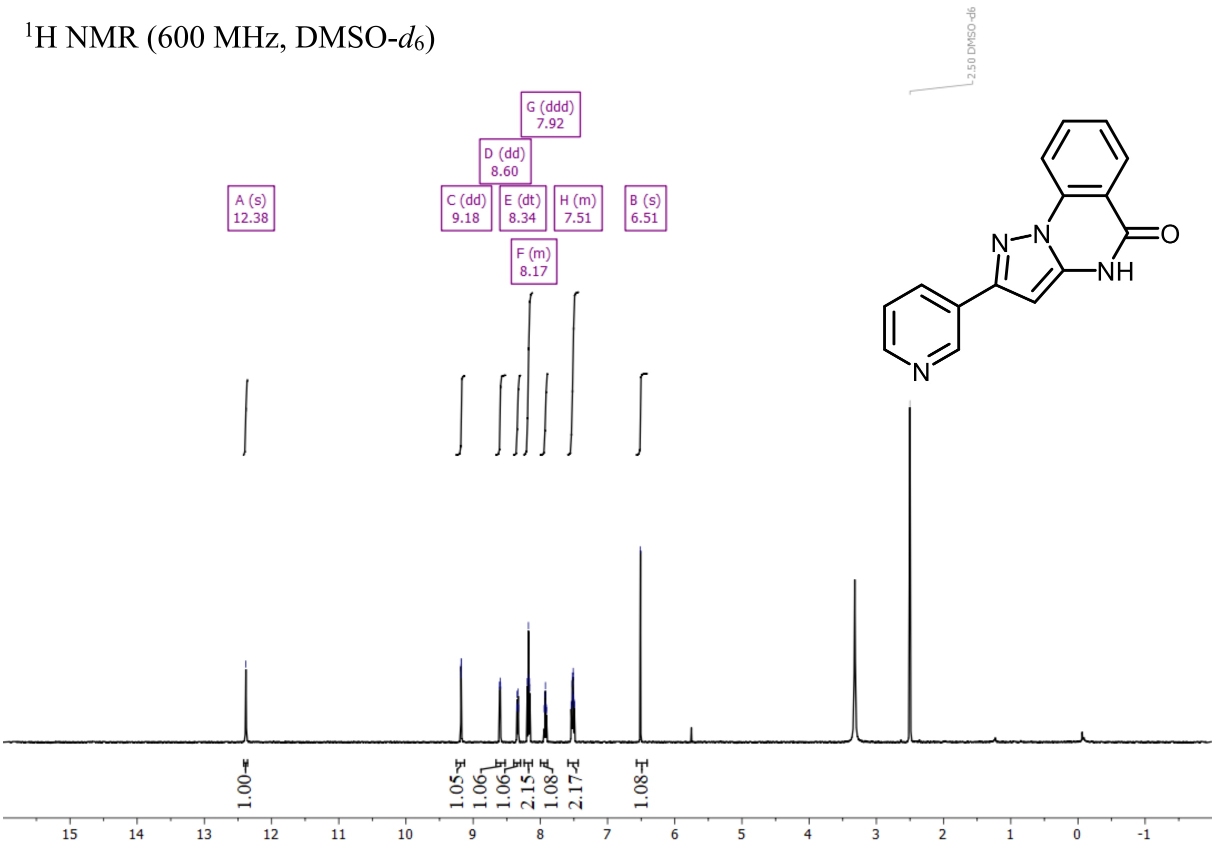

^{13}C NMR (151 MHz, DMSO-d_6)

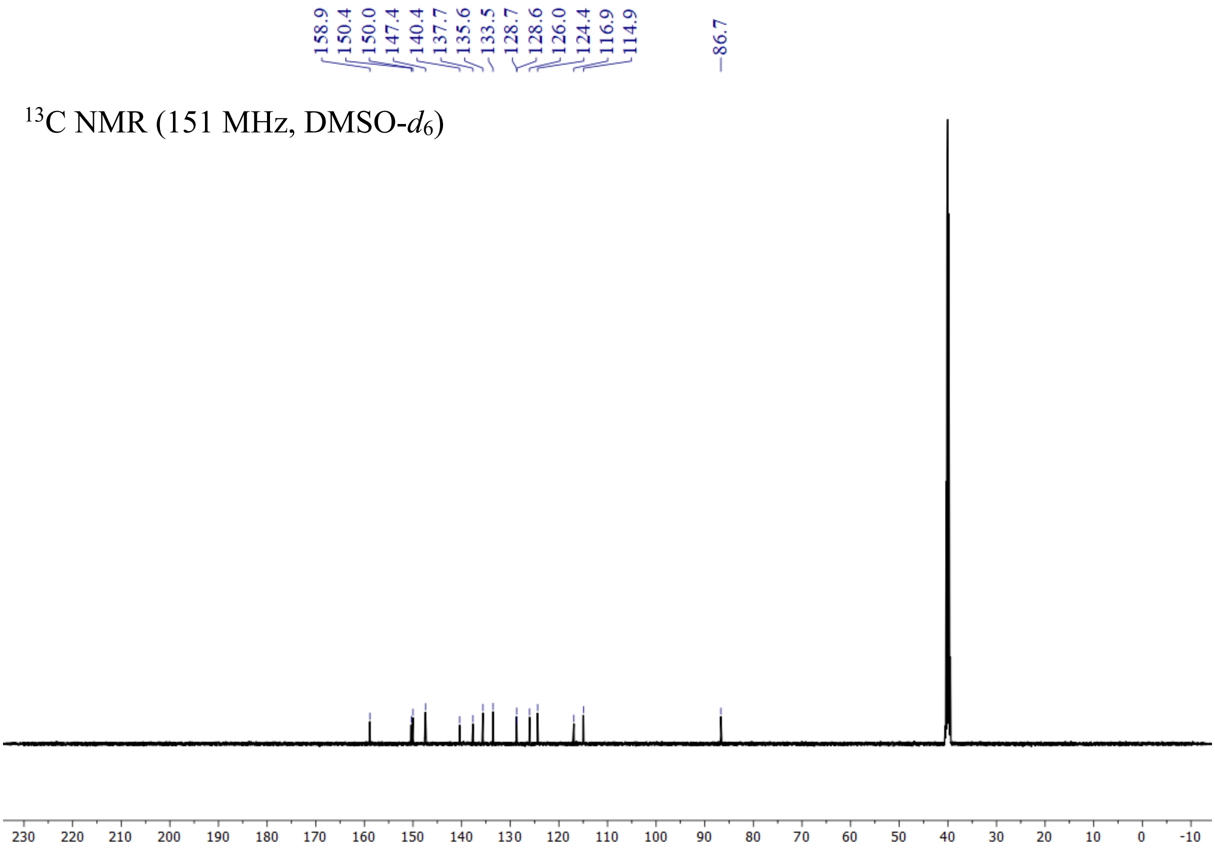

(5-amino-3-(pyridin-3-yl)-1*H*-1,2,4-triazol-1-yl)(2-iodophenyl)methanone (16)

^{1}H NMR (600 MHz, DMSO-d_6)

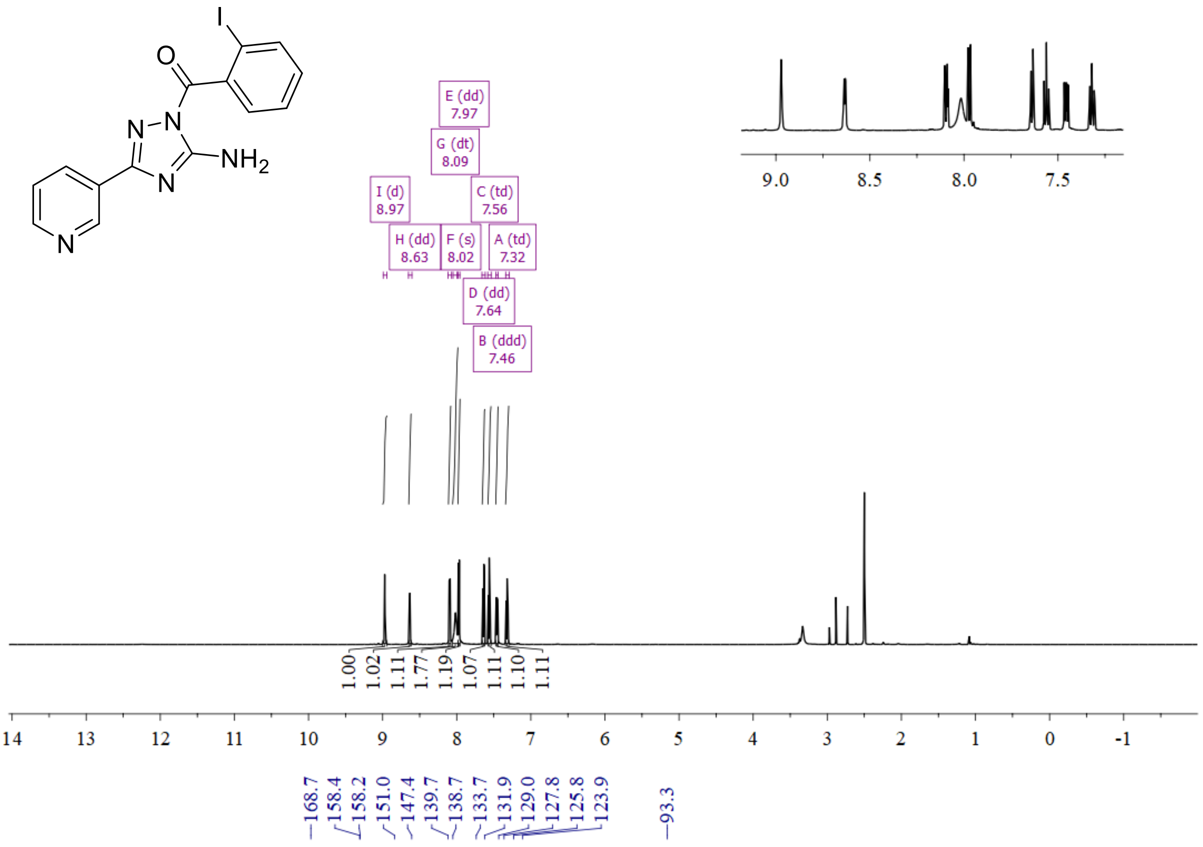

^{13}C NMR (151 MHz, DMSO-d_6)

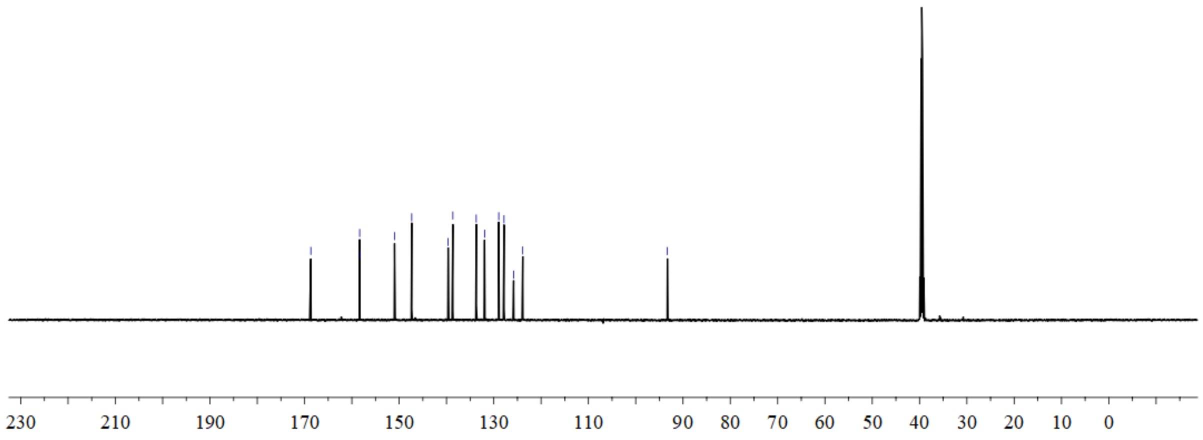

2-iodo-*N*-(3-(pyridin-3-yl)-1*H*-1,2,4-triazol-5-yl)benzamide (16')

^{1}H NMR (600 MHz, DMSO-d_6)

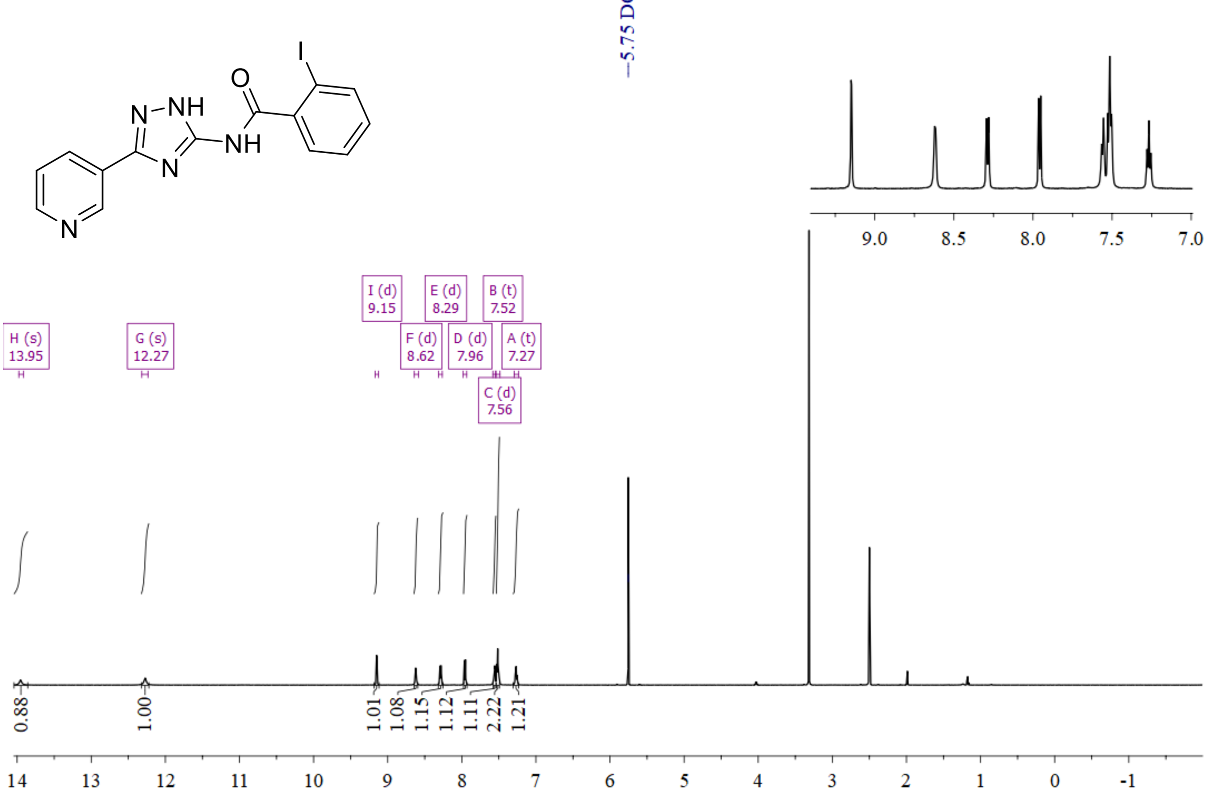

^{13}C NMR (151 MHz, DMSO-d_6)

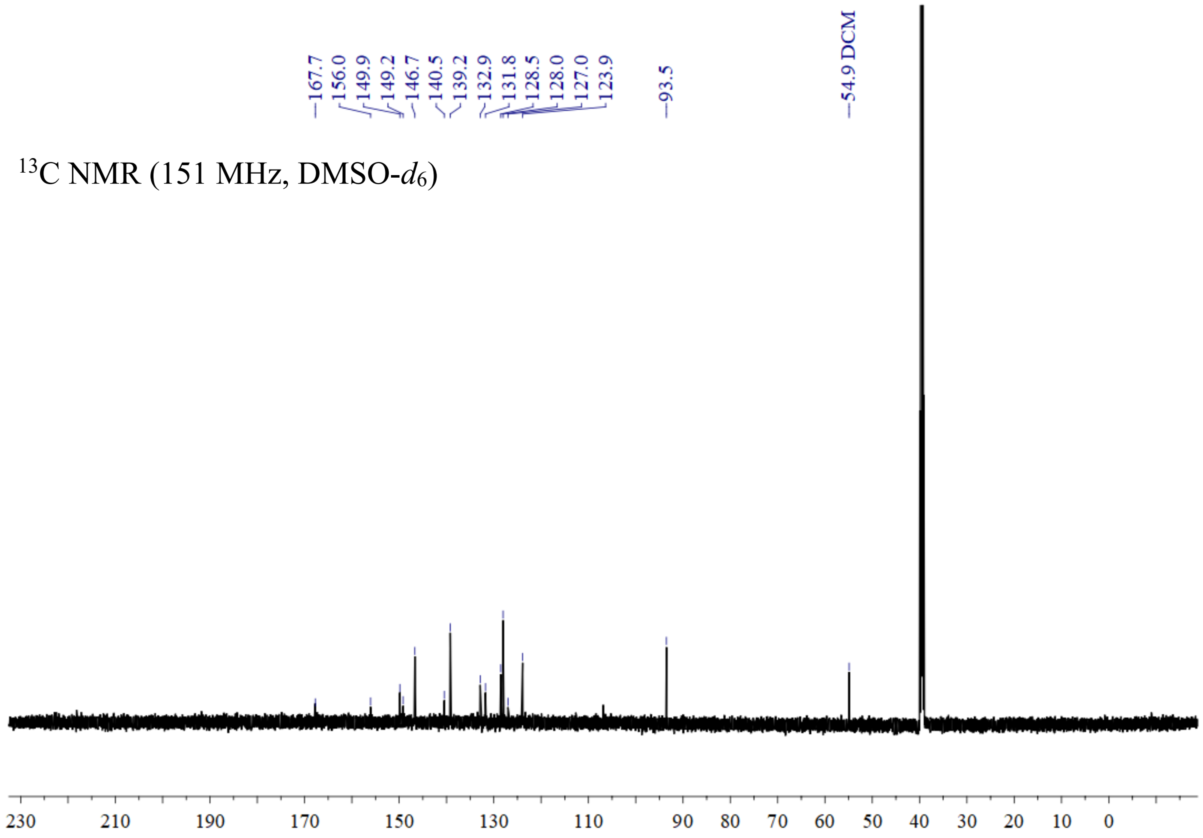

(5-amino-3-(pyridin-4-yl)-1*H*-1,2,4-triazol-1-yl)(2-iodophenyl)methanone (17)

^{1}H NMR (400 MHz, DMSO-d_6)

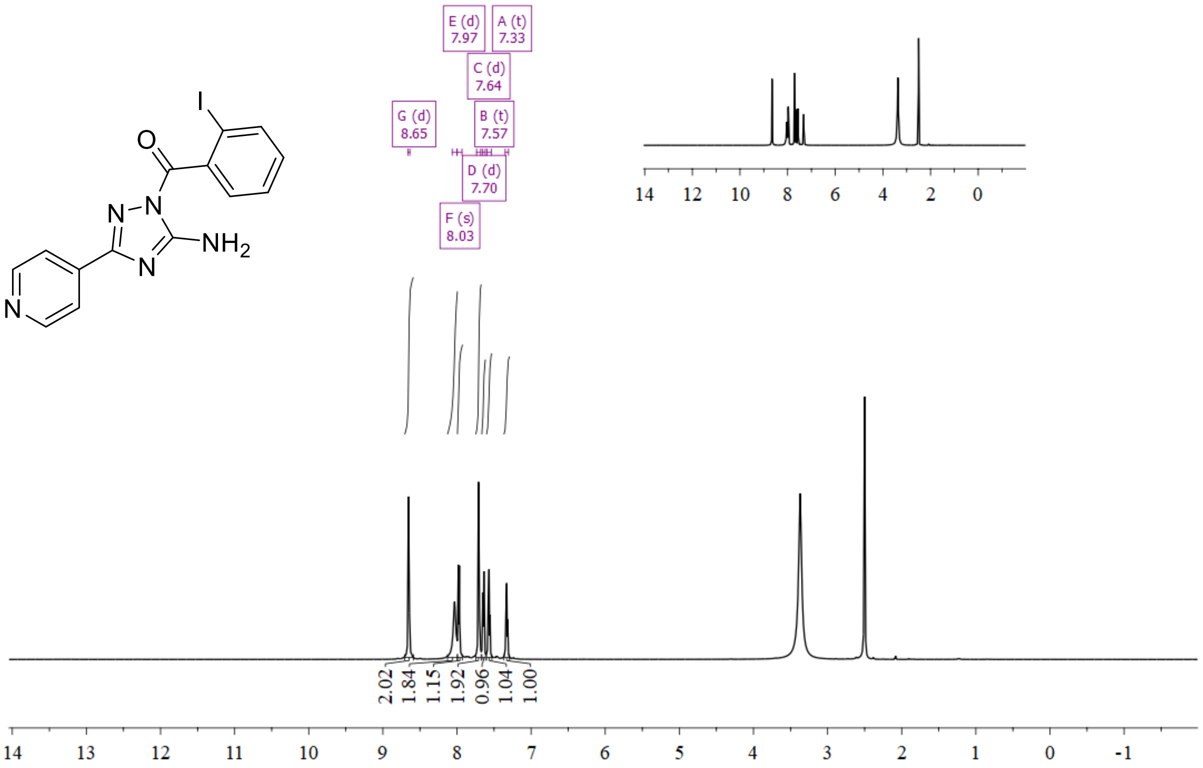

^{13}C NMR (101 MHz, DMSO-d_6)

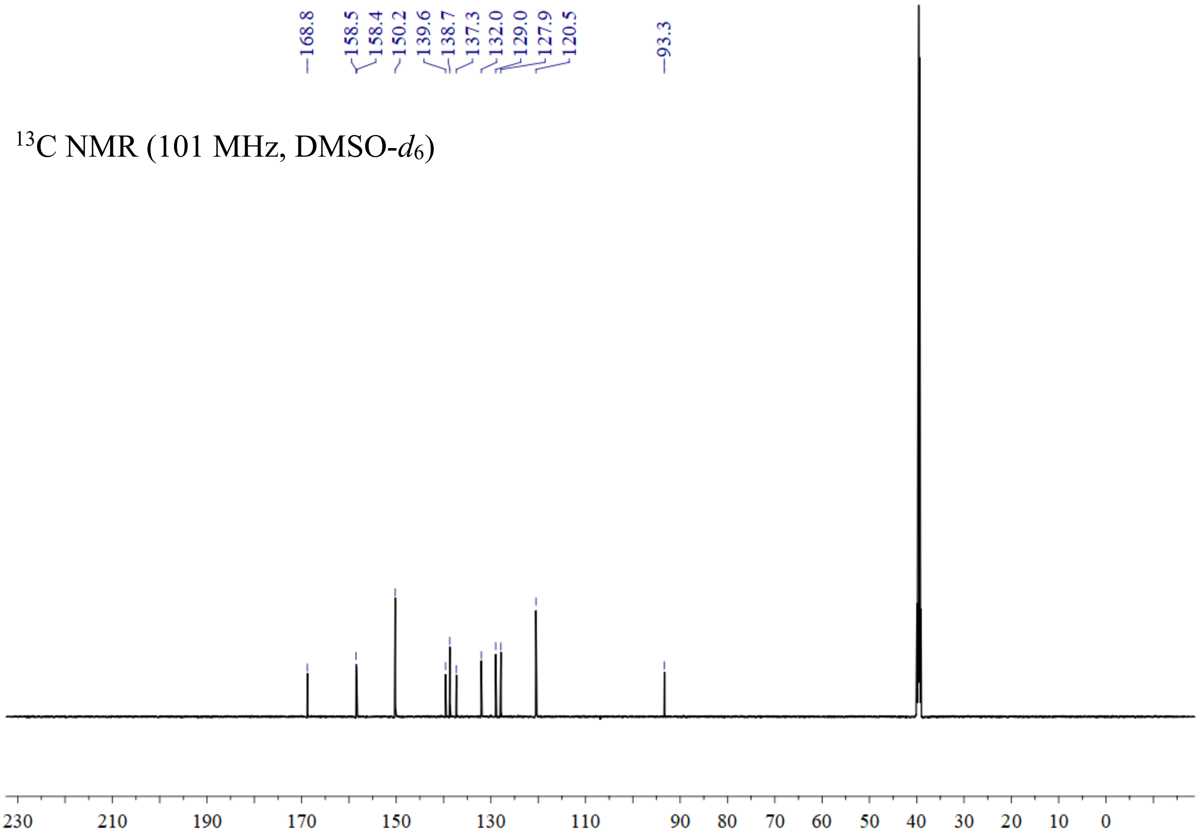

2-iodo-*N*-(3-(pyridin-4-yl)-1*H*-1,2,4-triazol-5-yl)benzamide (17')

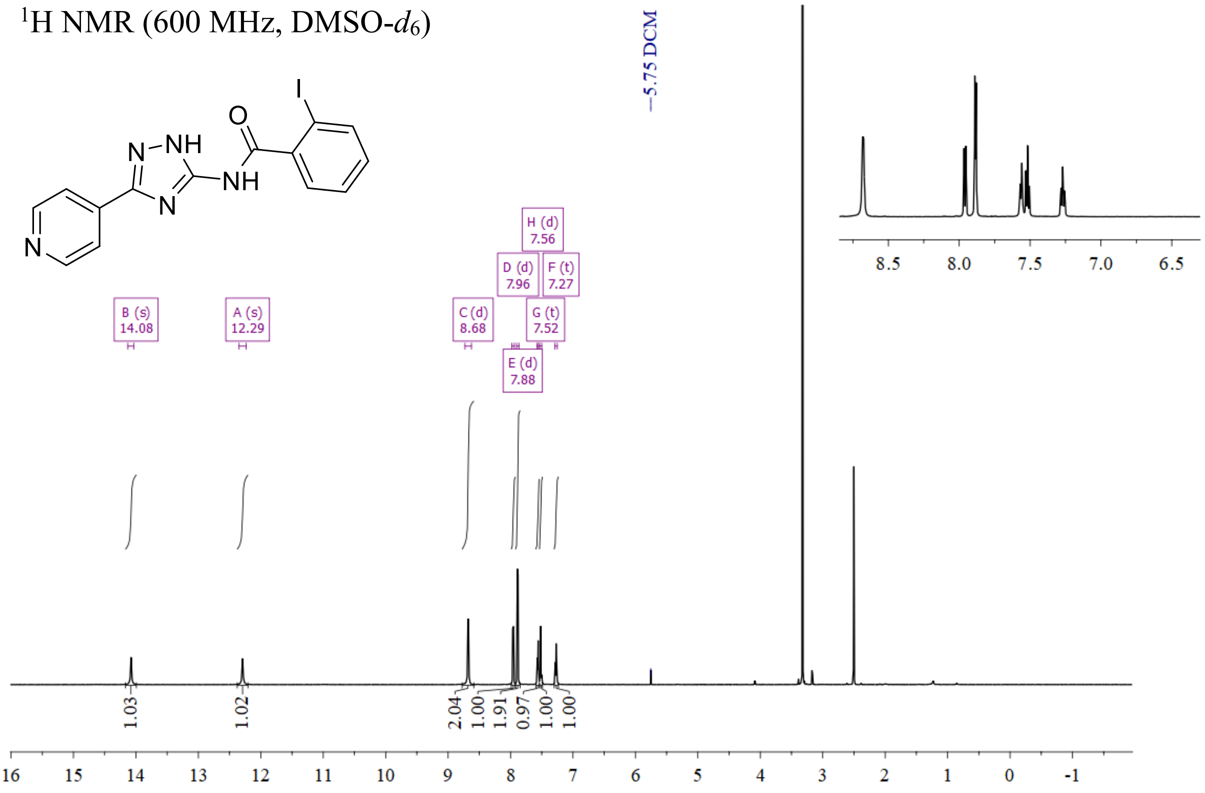

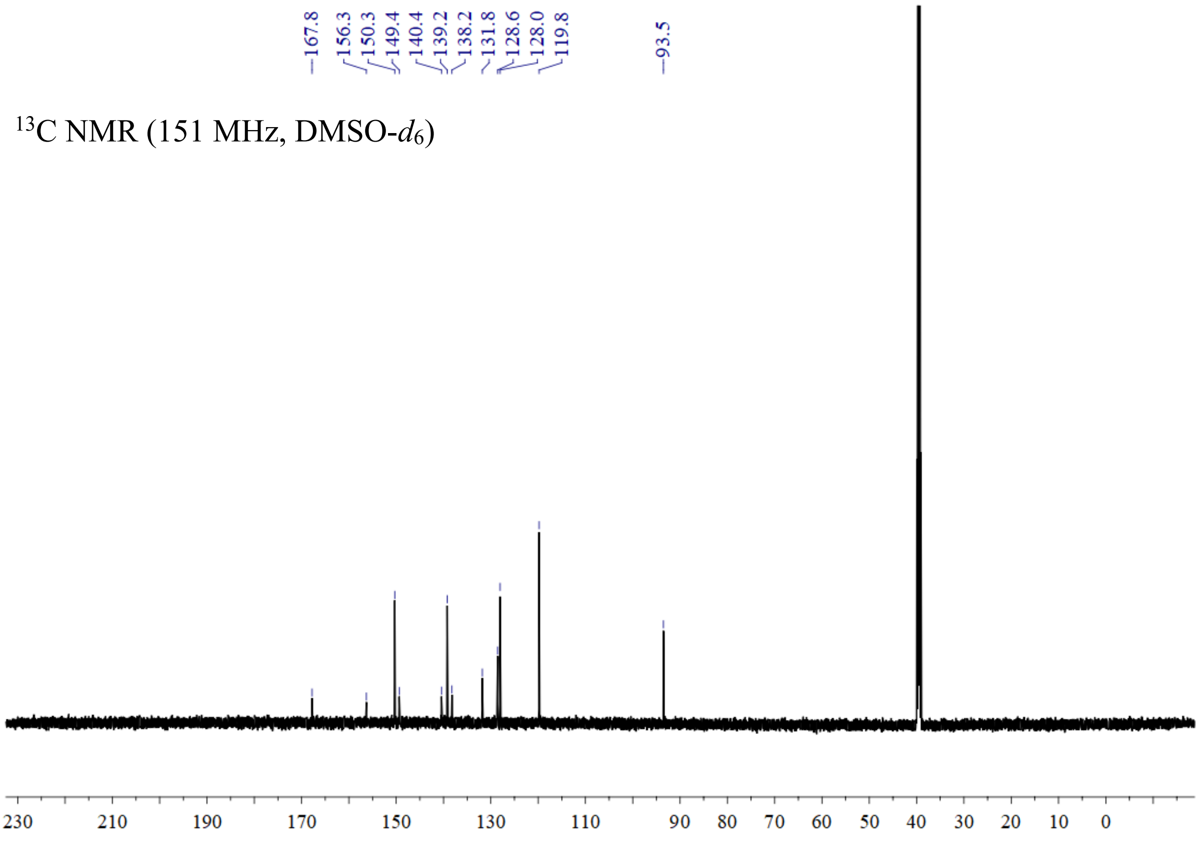

4-[(5-chlorothiophen-2-yl)methyl]-2-(pyrazin-2-yl)-[1,2,4]triazolo[5,1-*b*]quinazolin-9(4*H*)-one (19)

^{1}H NMR (600 MHz, DMSO-d_6)

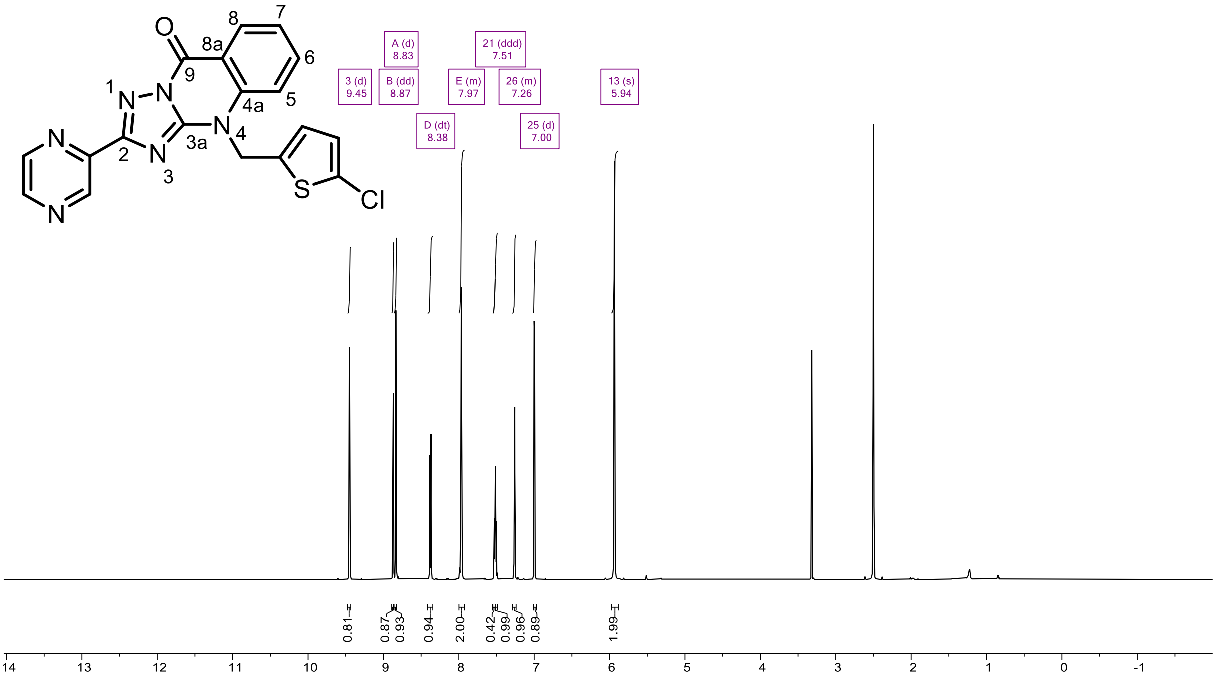

^{13}C NMR (151 MHz, DMSO-d_6)

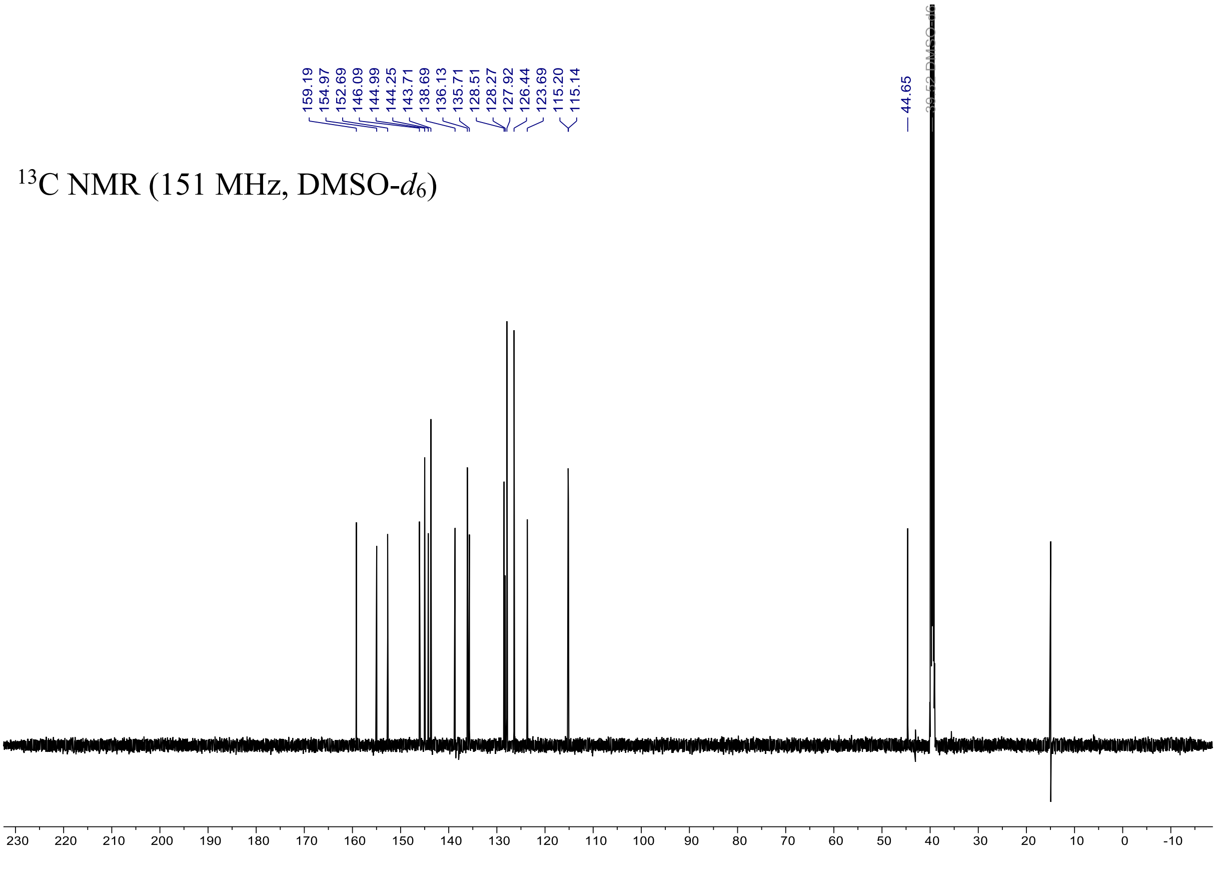

2-(pyridin-3-yl)-[1,2,4]triazolo[5,1-*b*]quinazolin-9(4*H*)-one (20)

^{1}H NMR (600 MHz, DMSO-d_6)

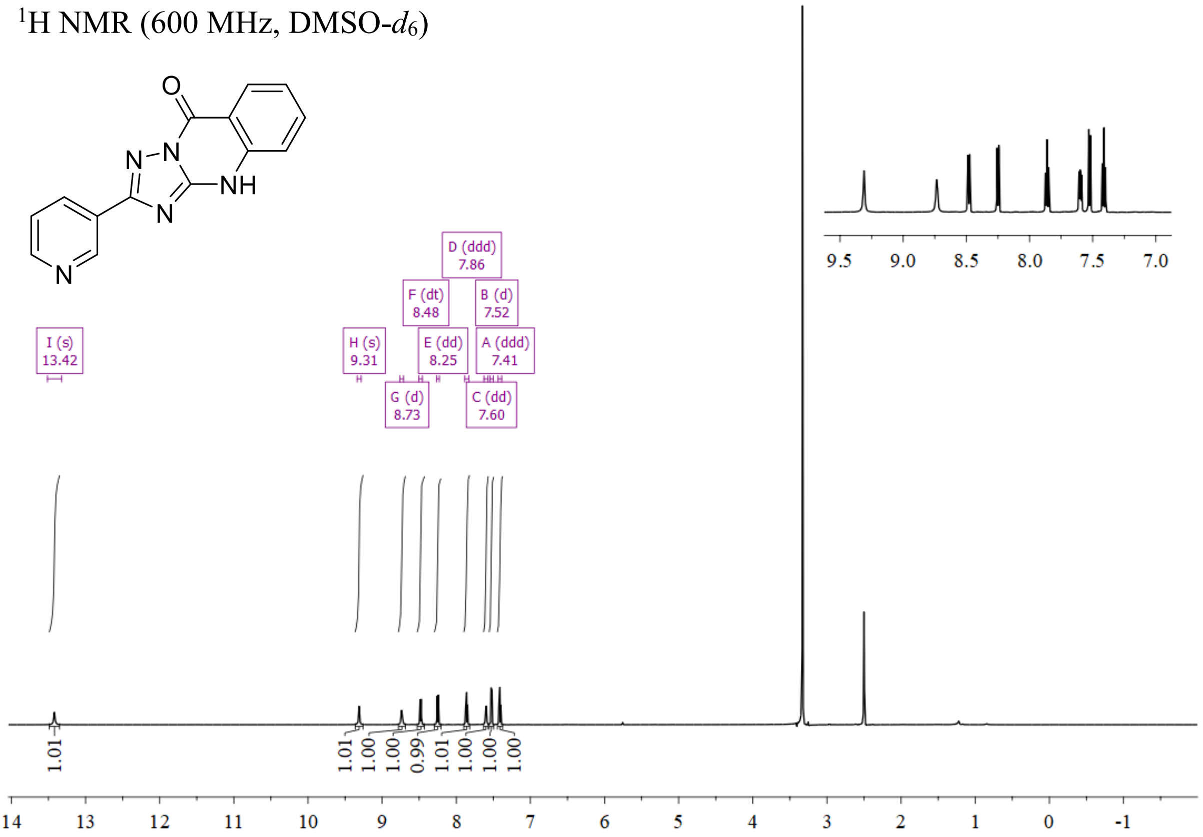

^{13}C NMR (151 MHz, DMSO-d_6)

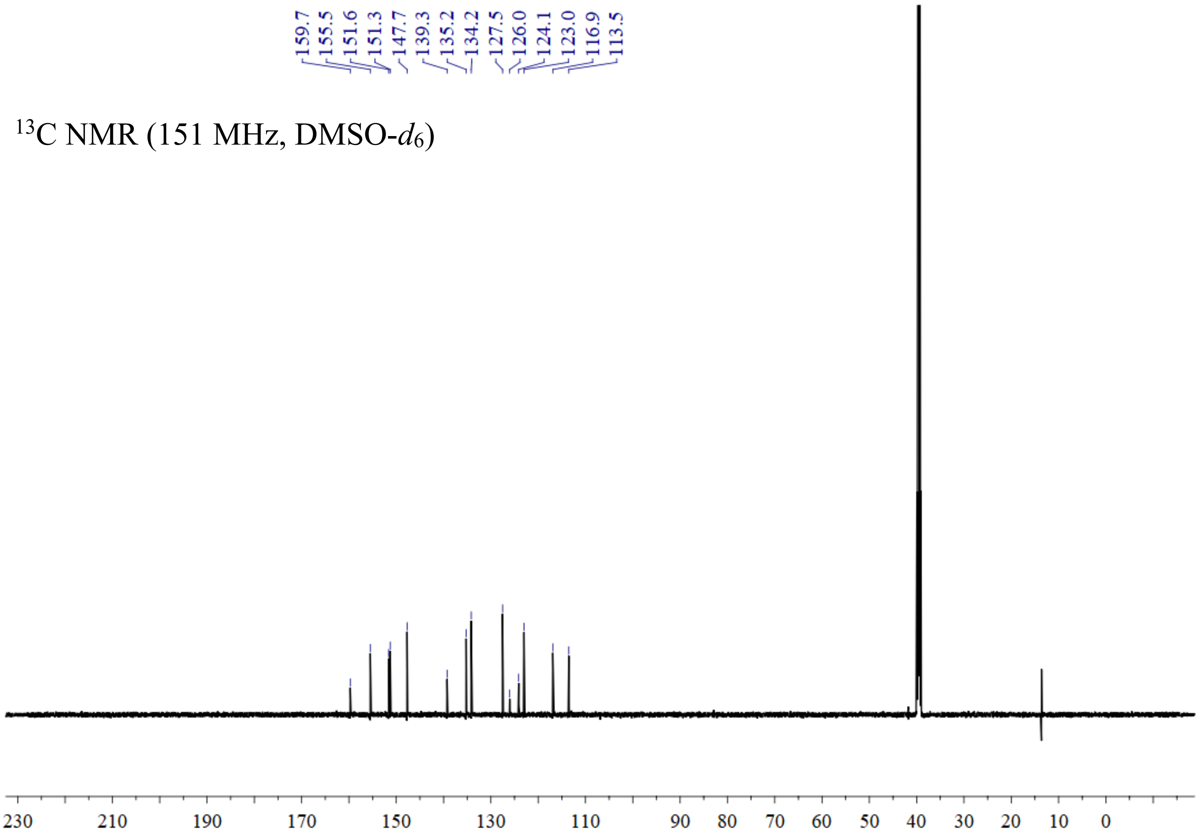

2-(pyridin-3-yl)-[1,2,4]triazolo[1,5-*a*]quinazolin-5(4*H*)-one (21).

^{1}H NMR (600 MHz, DMSO-d_6)

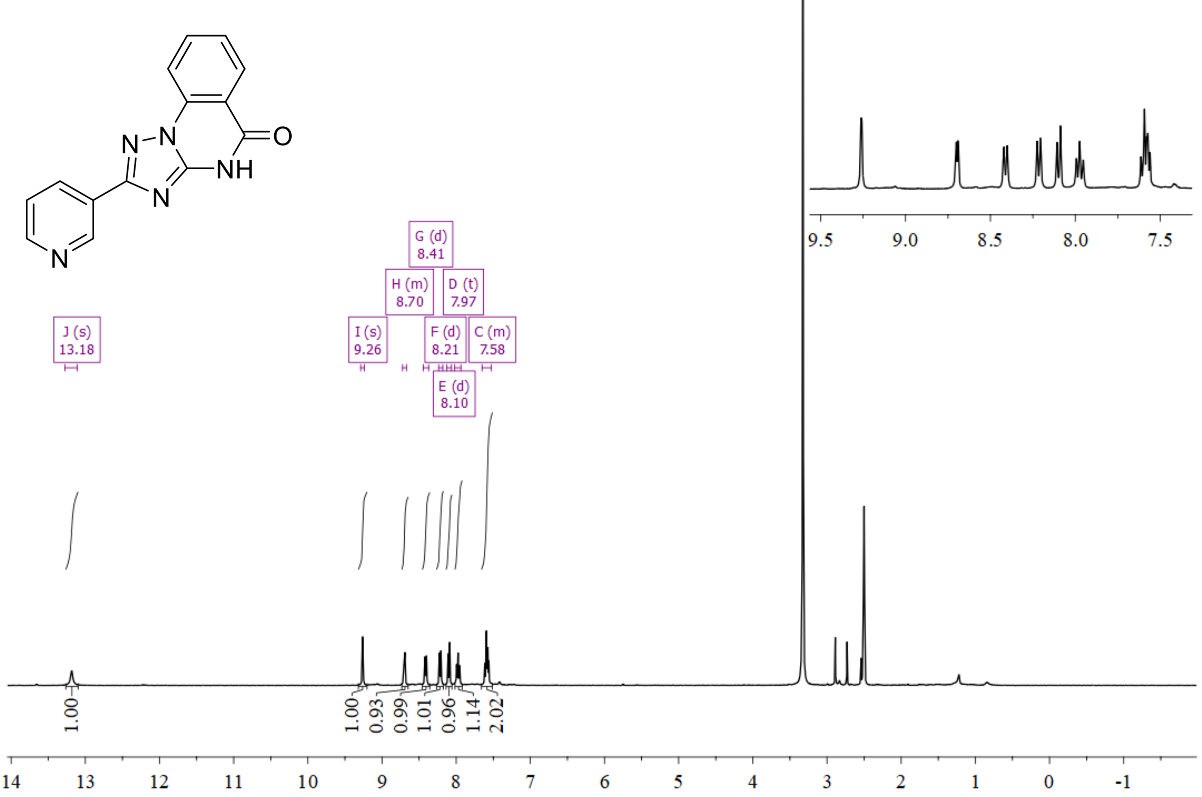

^{13}C NMR (151 MHz, DMSO-d_6)

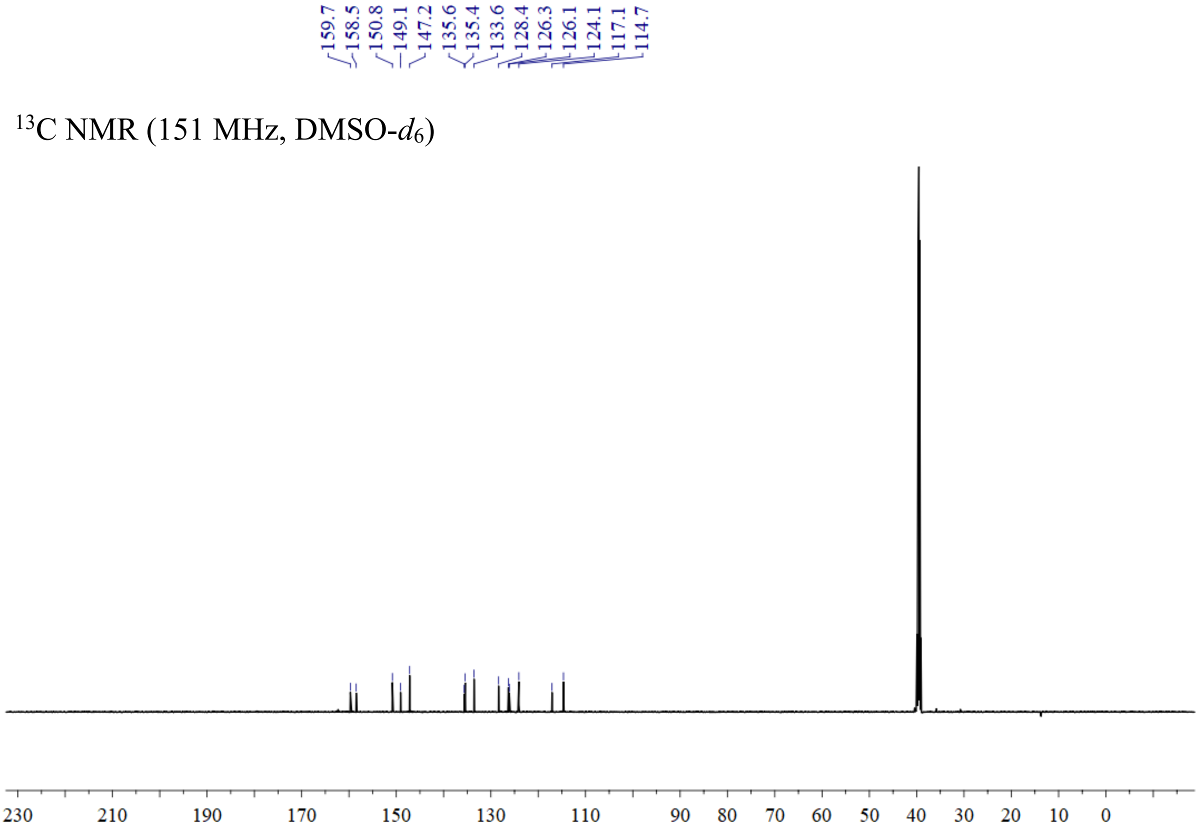

2-(pyridin-4-yl)-[1,2,4]triazolo[5,1-*b*]quinazolin-9(4*H*)-one (22)

^{1}H NMR (600 MHz, DMSO-*d*$_6$)

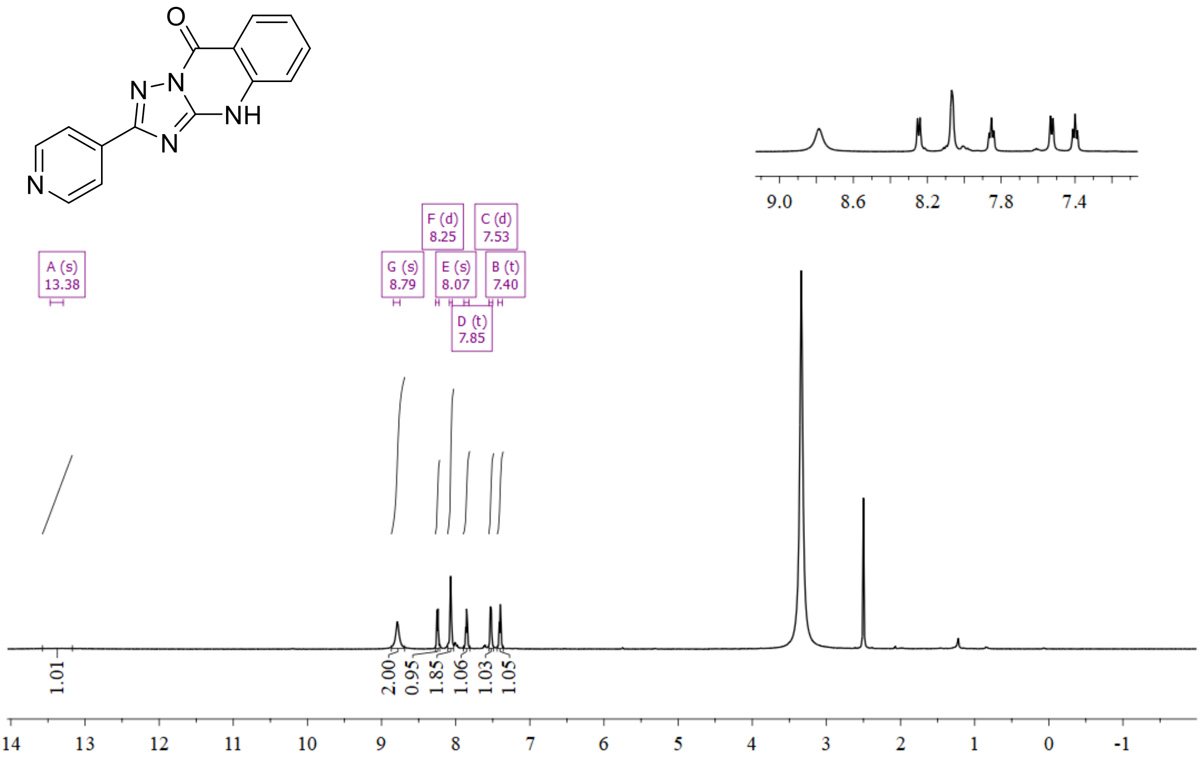

^{13}C NMR (151 MHz, DMSO-*d*$_6$)

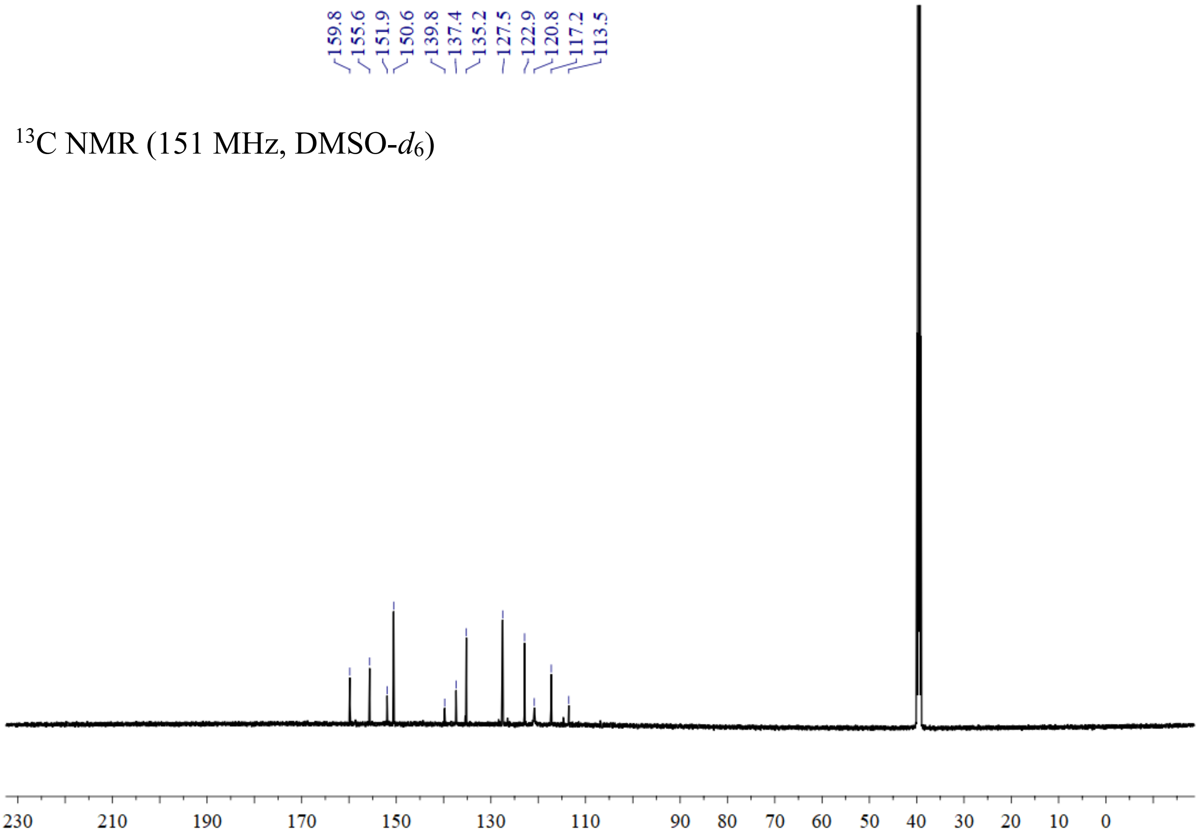

2-(pyridin-4-yl)-[1,2,4]triazolo[1,5-*a*]quinazolin-5(4*H*)-one (23)

^{1}H NMR (600 MHz, DMSO-d_6)

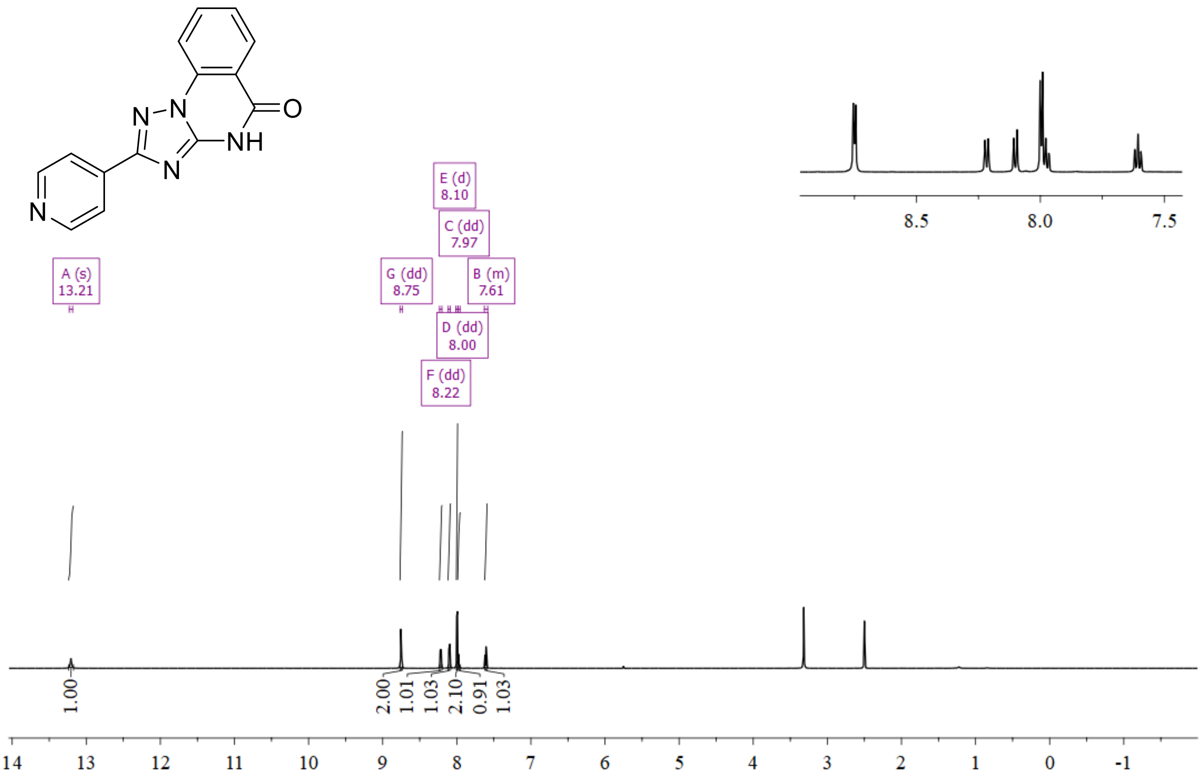

^{13}C NMR (151 MHz, DMSO-d_6)

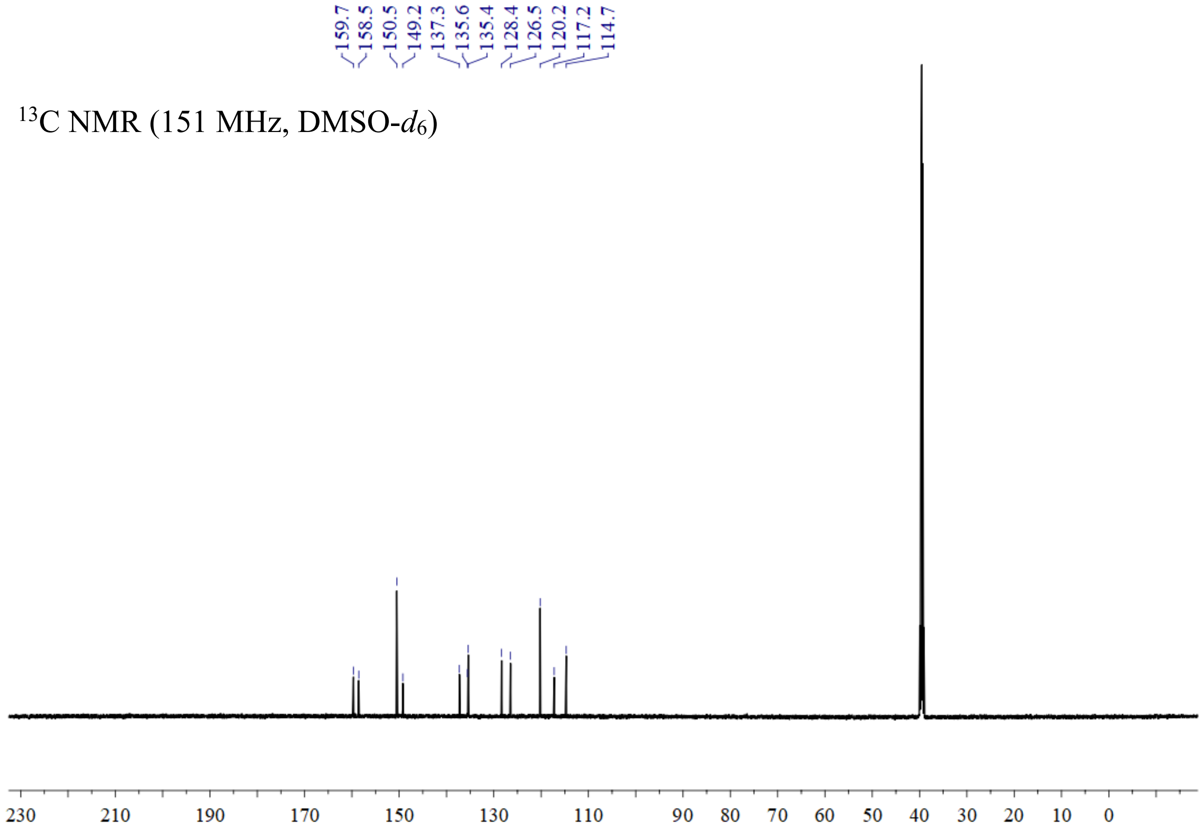

1-{5-[(4-methoxybenzyl)amino]-3-(pyridin-3-yl)-1*H*-pyrazol-1-yl}-2,2-dimethylpropan-1-one (24b)

^{1}H NMR (600 MHz, DMSO-d_6)

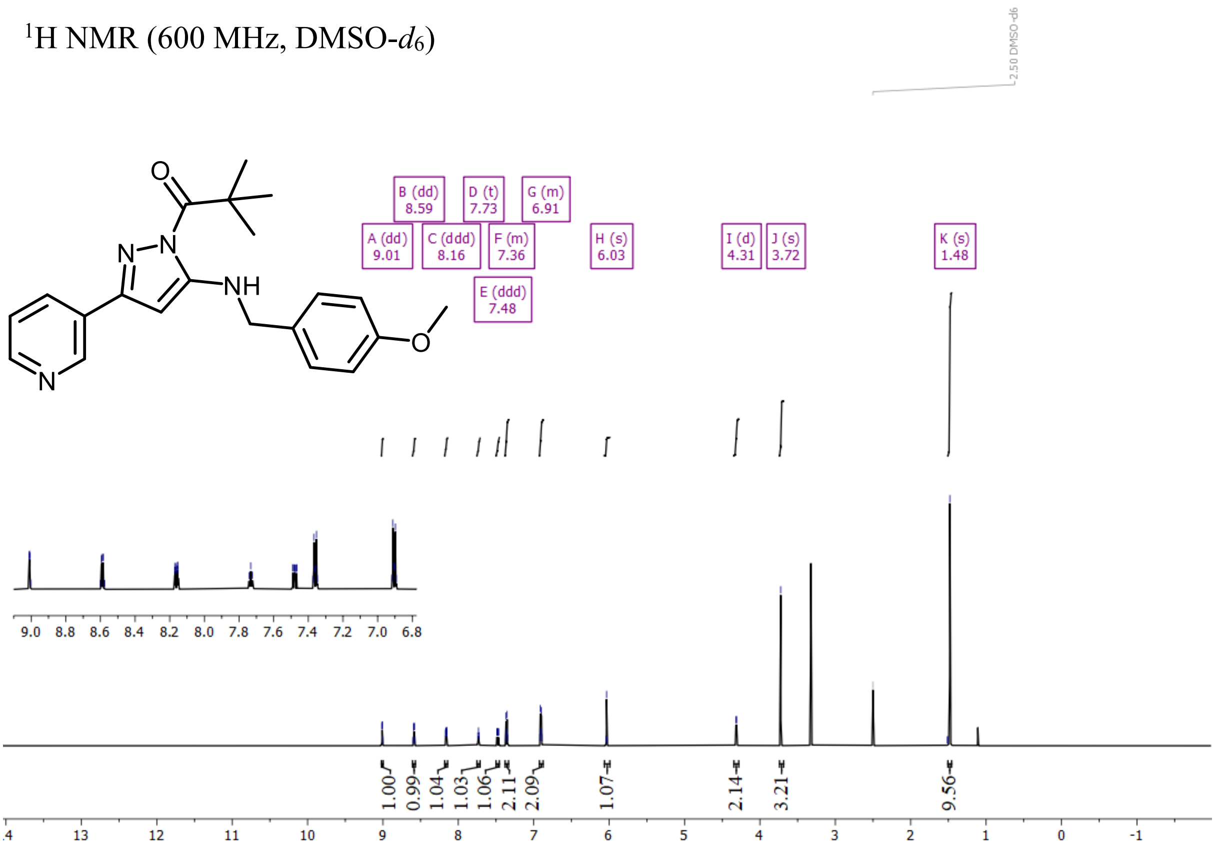

^{13}C NMR (151 MHz, DMSO-d_6)

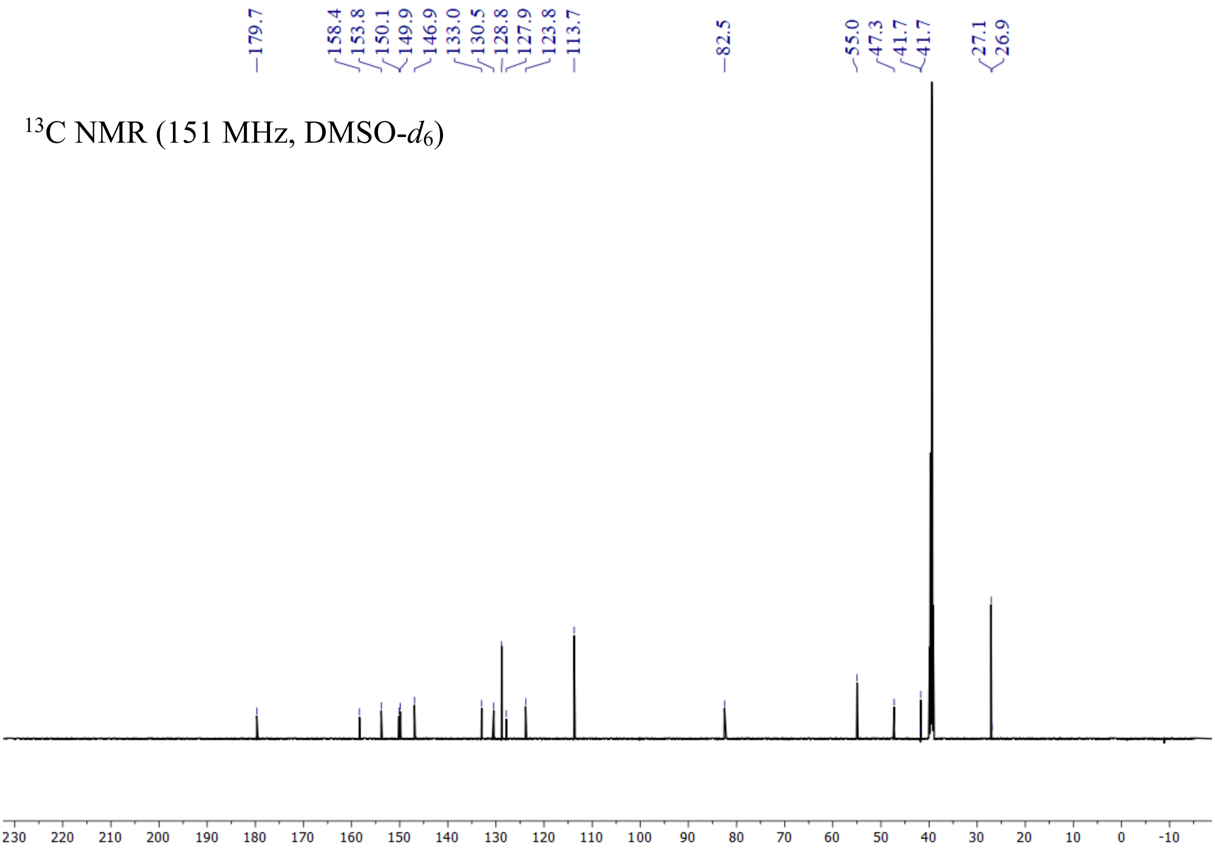

2,2-dimethyl-1-(5-{[(naphthalen-1-yl)methyl]amino}-3-(pyridin-3-yl)-1*H*-pyrazol-1-yl)propan-1-one (24c)

^{1}H NMR (600 MHz, DMSO-d_6)

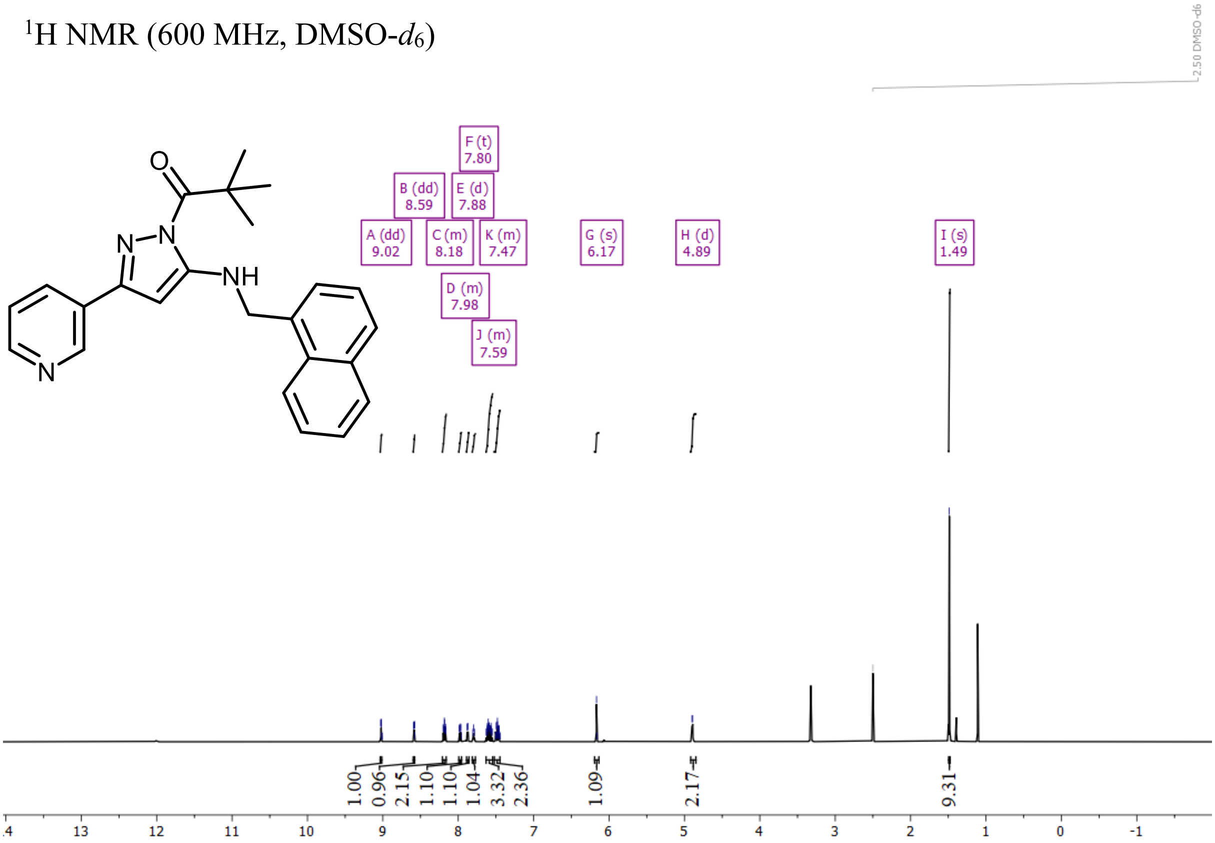

^{13}C NMR (151 MHz, DMSO-d_6)

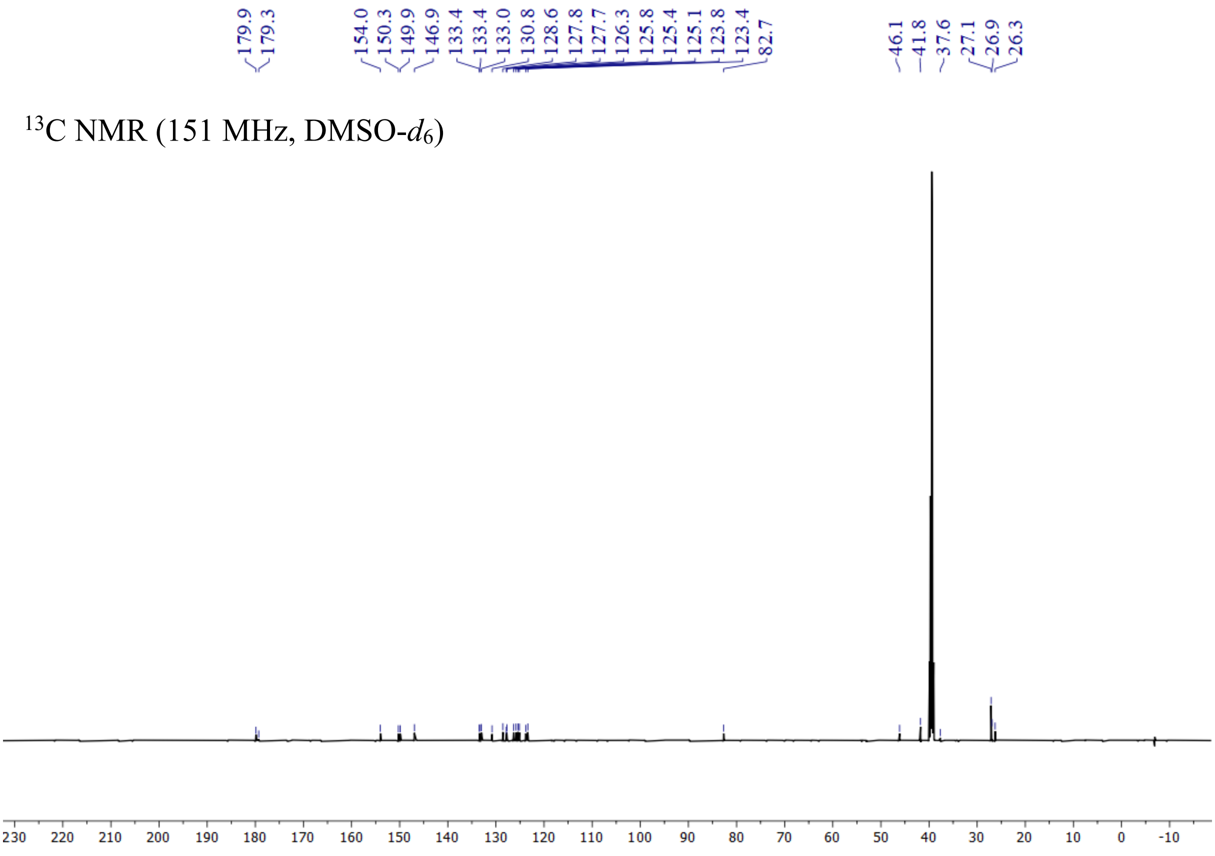

1-(5-{[(furan-2-yl)methyl]amino}-3-(pyridin-3-yl)-1*H*-pyrazol-1-yl)-2,2-dimethylpropan-1-one (24d)

^{1}H NMR (600 MHz, DMSO-d_6)

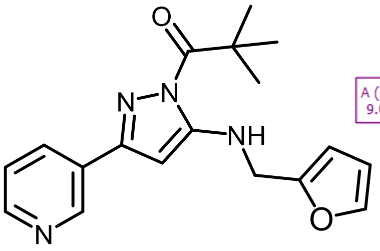

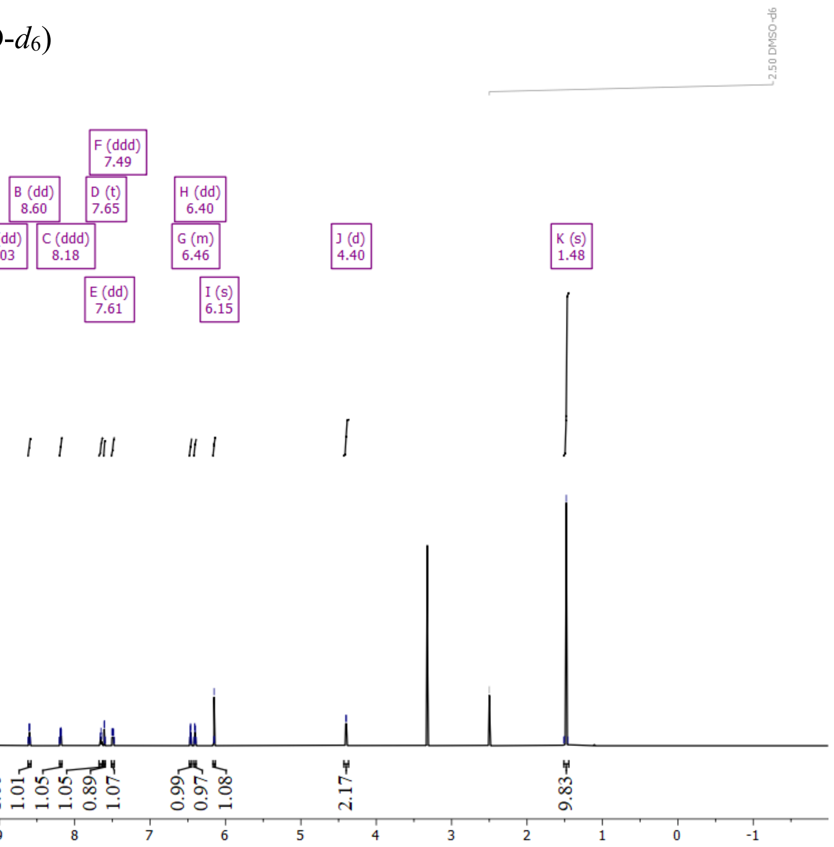

1-(5-{[(5-chlorothiophen-2-yl)methyl]amino}-3-(pyridin-3-yl)-1*H*-pyrazol-1-yl)-2,2-dimethylpropan-1-one (24e)

^{1}H NMR (600 MHz, DMSO-d_6)

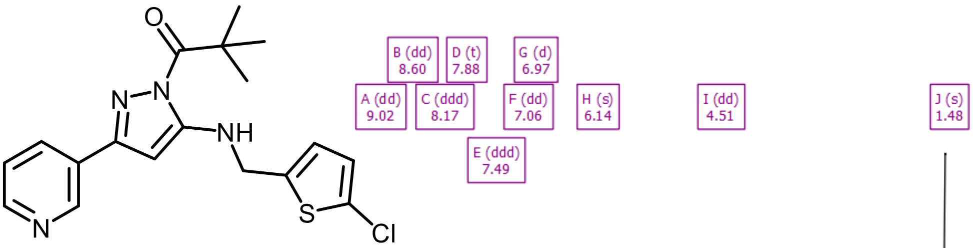

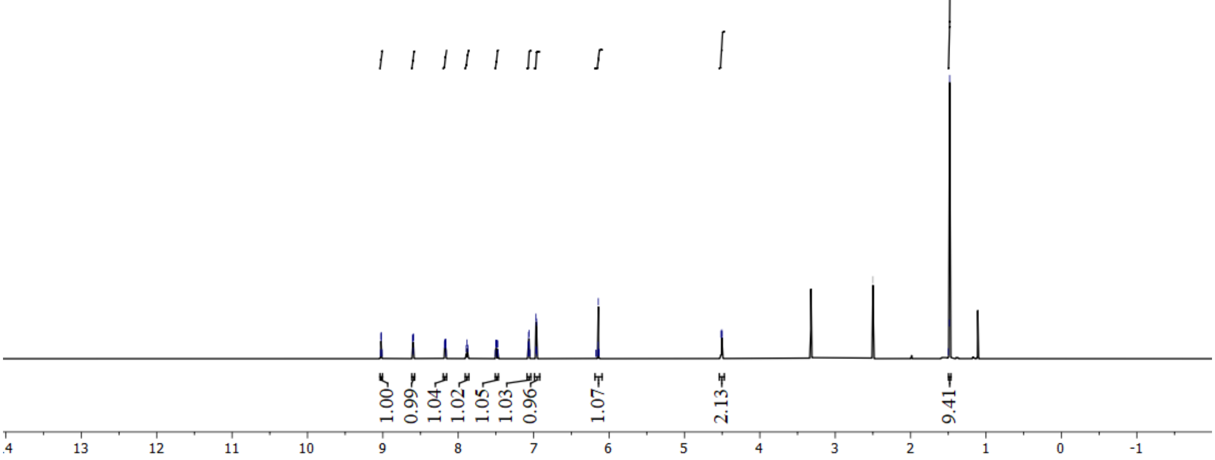

^{13}C NMR (151 MHz, DMSO-d_6)

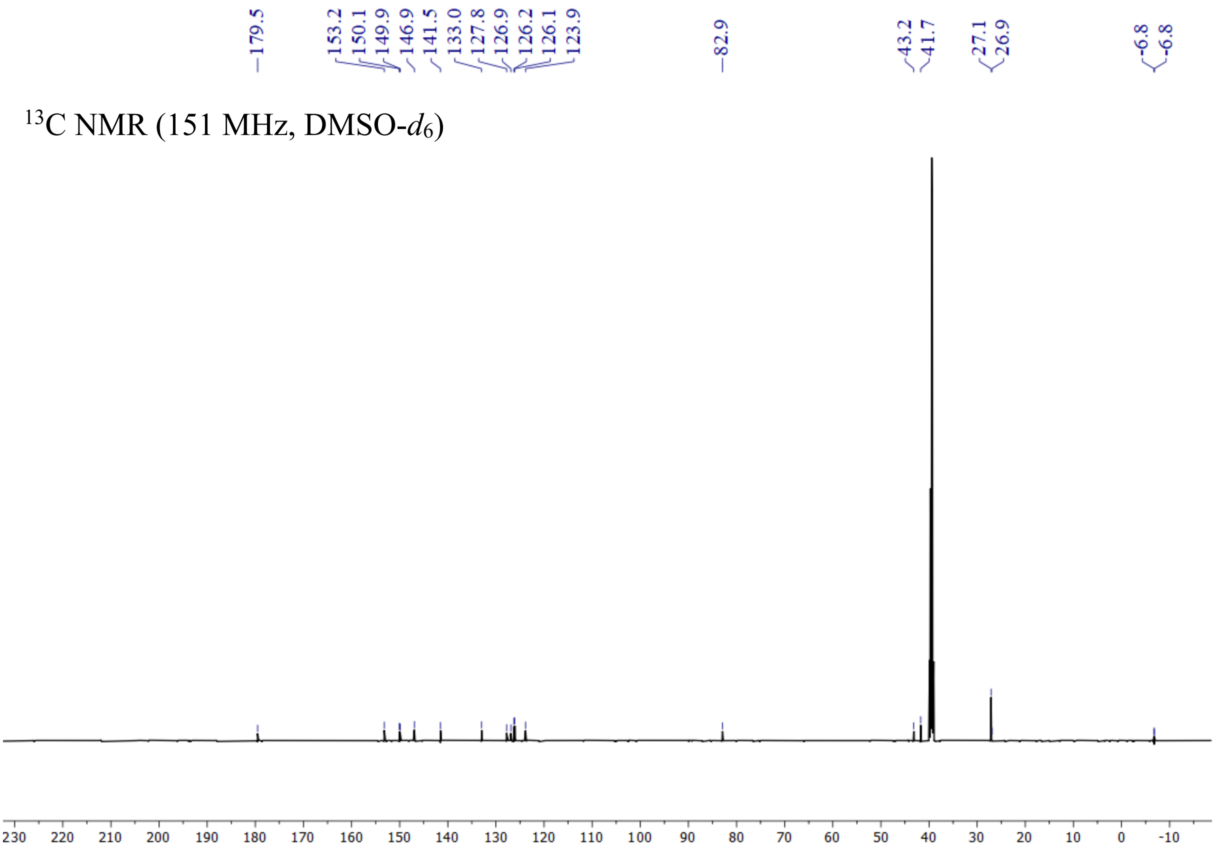

1-(5-{[(5-chlorothiophen-2-yl)methyl]amino}-3-phenyl-1*H*-pyrazol-1-yl)-2,2-dimethylpropan-1-one (24g)

^{1}H NMR (600 MHz, DMSO-d_6)

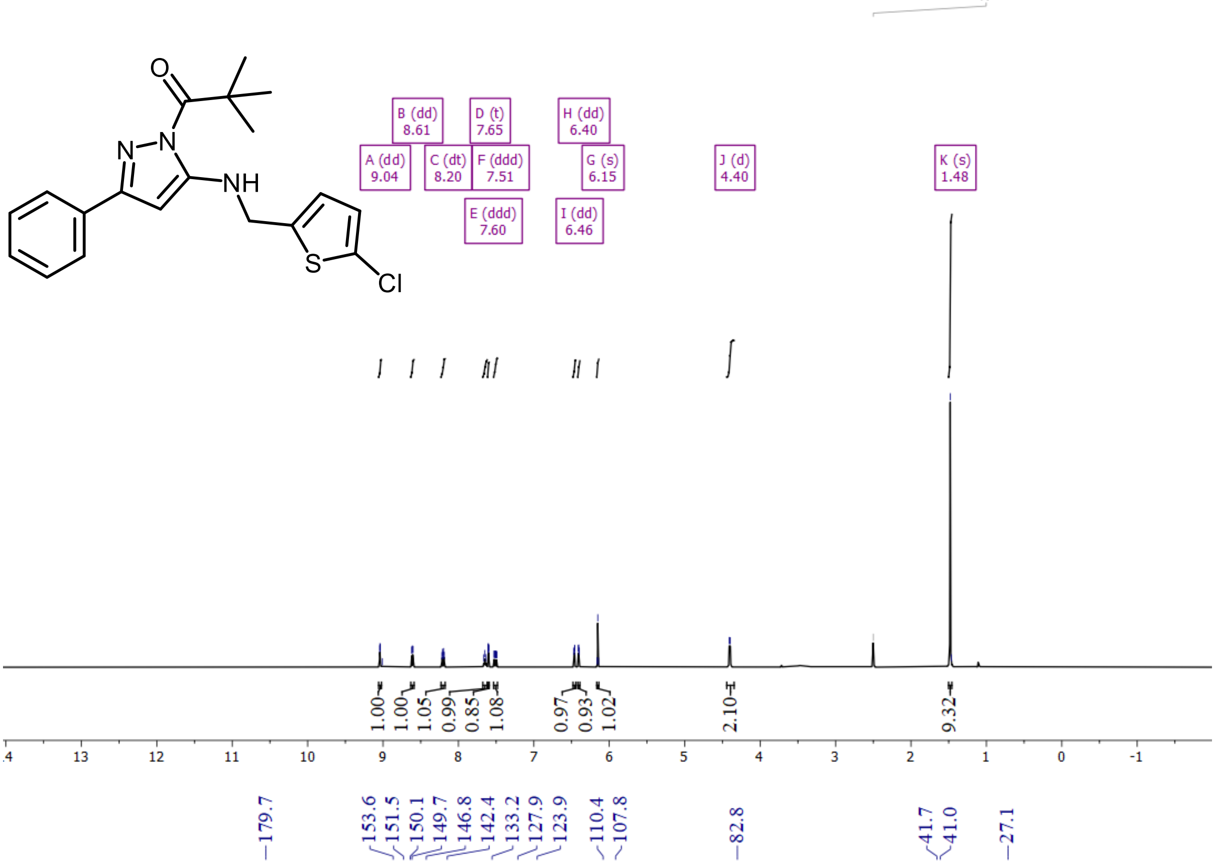

^{13}C NMR (151 MHz, DMSO-d_6)

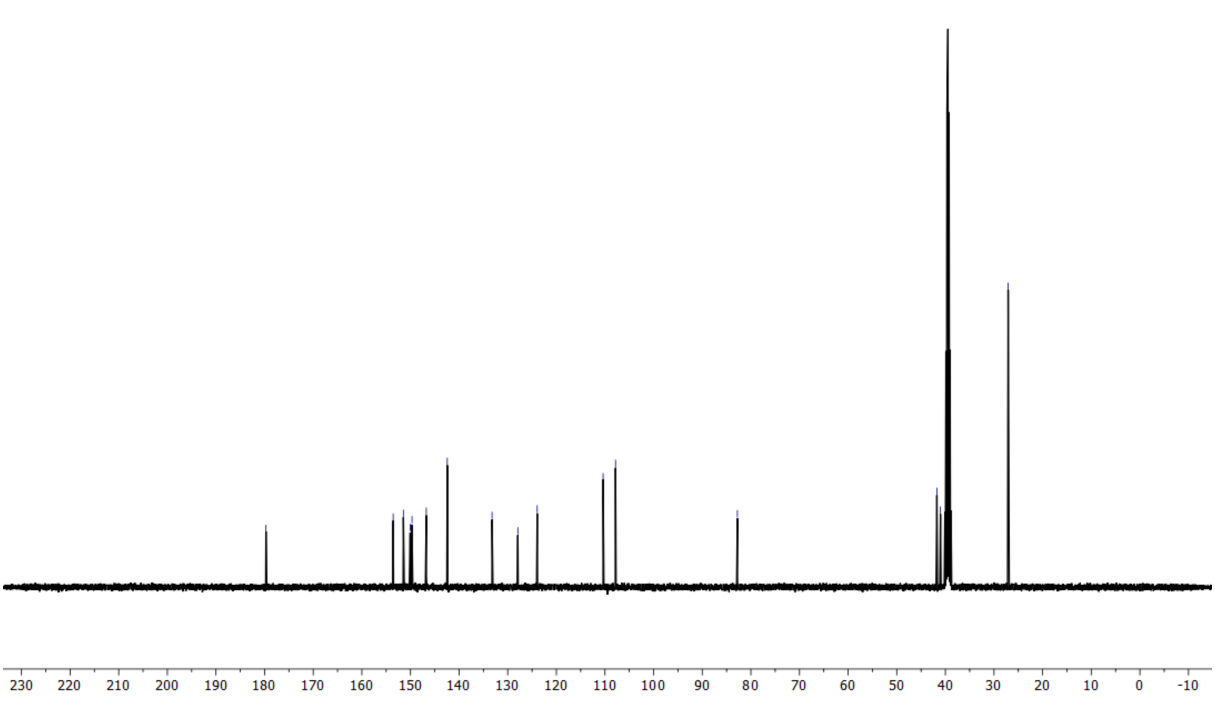

1-[5-(benzylamino)-3-cyclohexyl-1*H*-pyrazol-1-yl]-2,2-dimethylpropan-1-one (24h)

^{1}H NMR (600 MHz, DMSO-d_6)

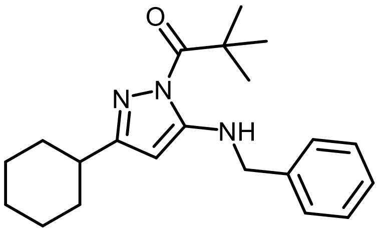

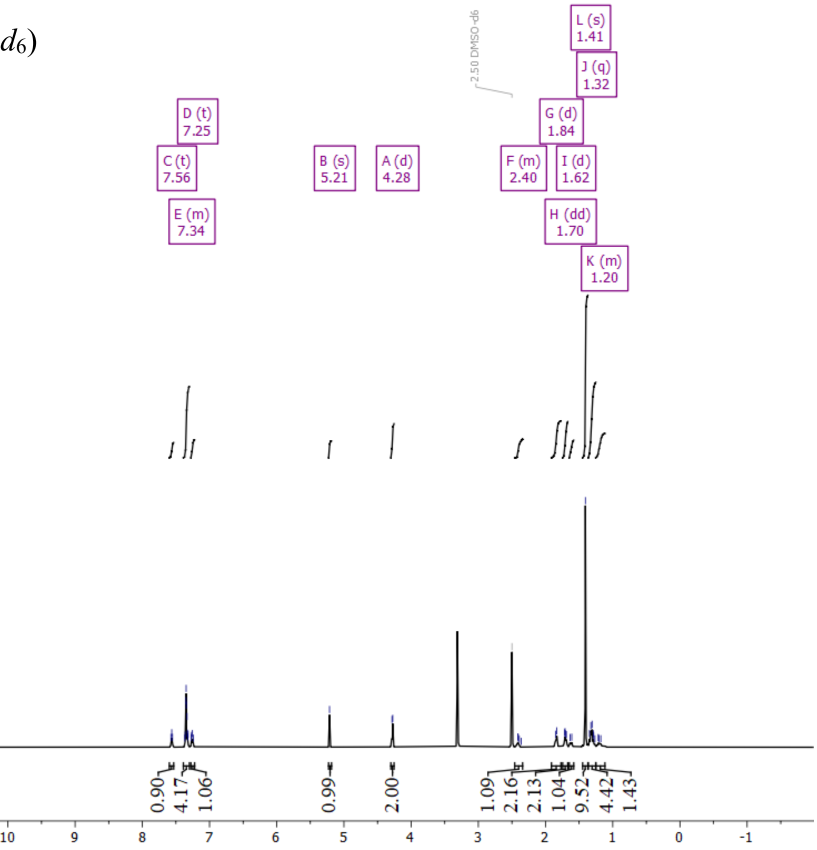

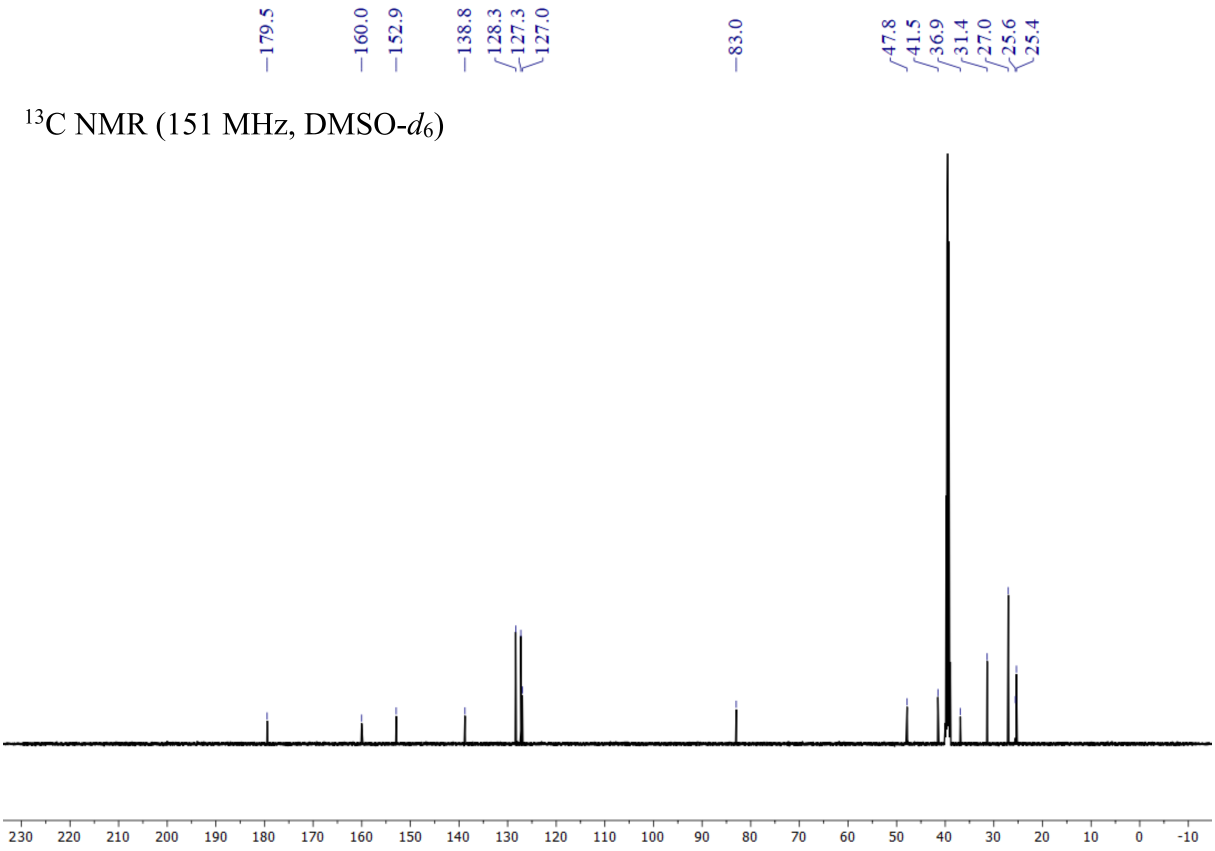

^{13}C NMR (151 MHz, DMSO-d_6)

1-(5-{[(5-chlorothiophen-2-yl)methyl]amino}-3-cyclohexyl-1*H*-pyrazol-1-yl)-2,2-dimethylpropan-1-one (24i)

^{1}H NMR (600 MHz, DMSO-d_6)

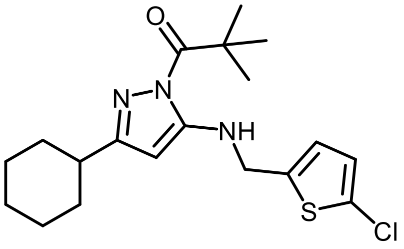

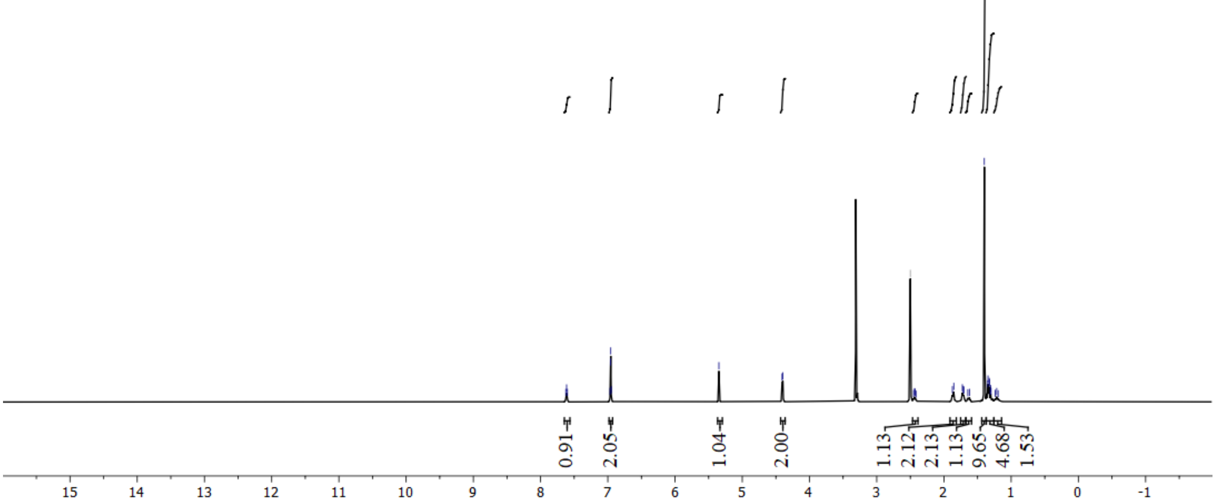

^{13}C NMR (151 MHz, DMSO-d_6)

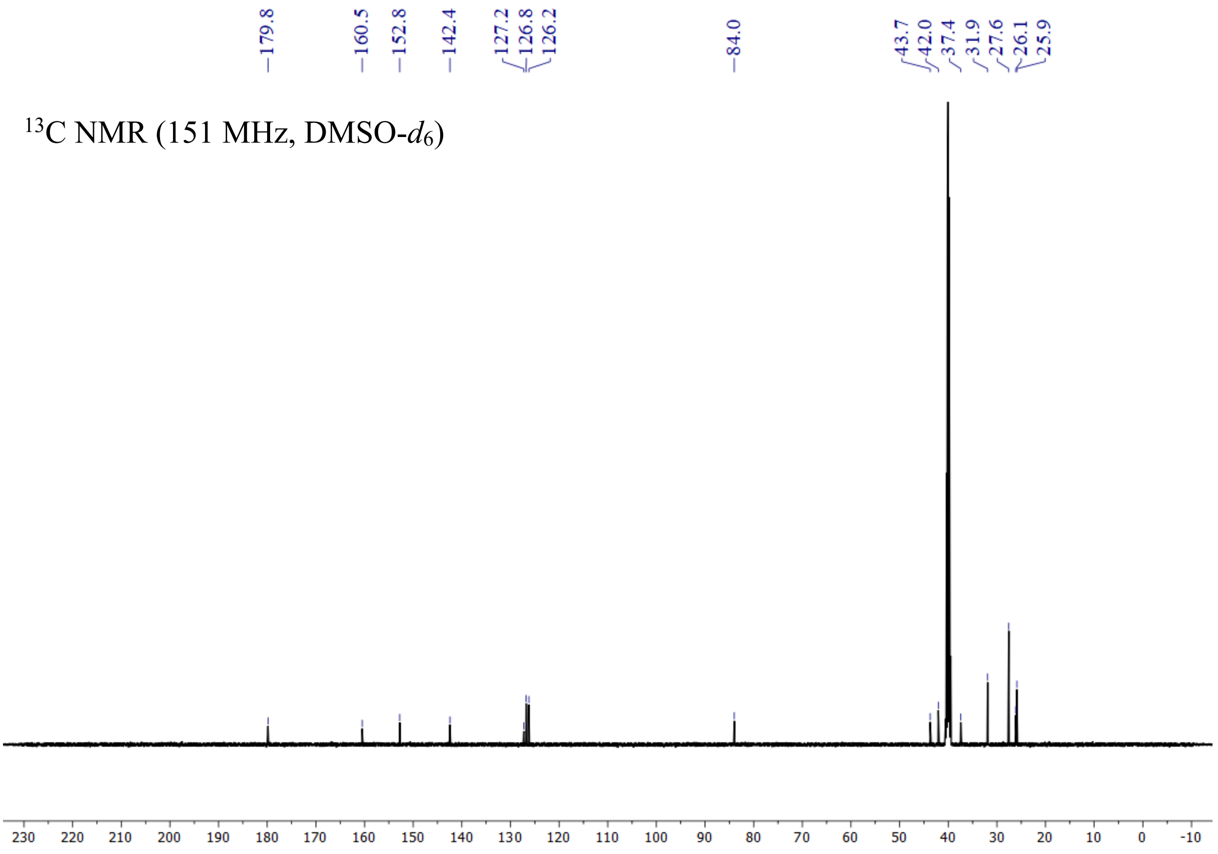

(5-{[(5-chlorothiophen-2-yl)methyl]amino}-3-(pyridin-3-yl)-1*H*-pyrazol-1-yl)(phenyl)methanone (25)

^{1}H NMR (600 MHz, DMSO-d_6)

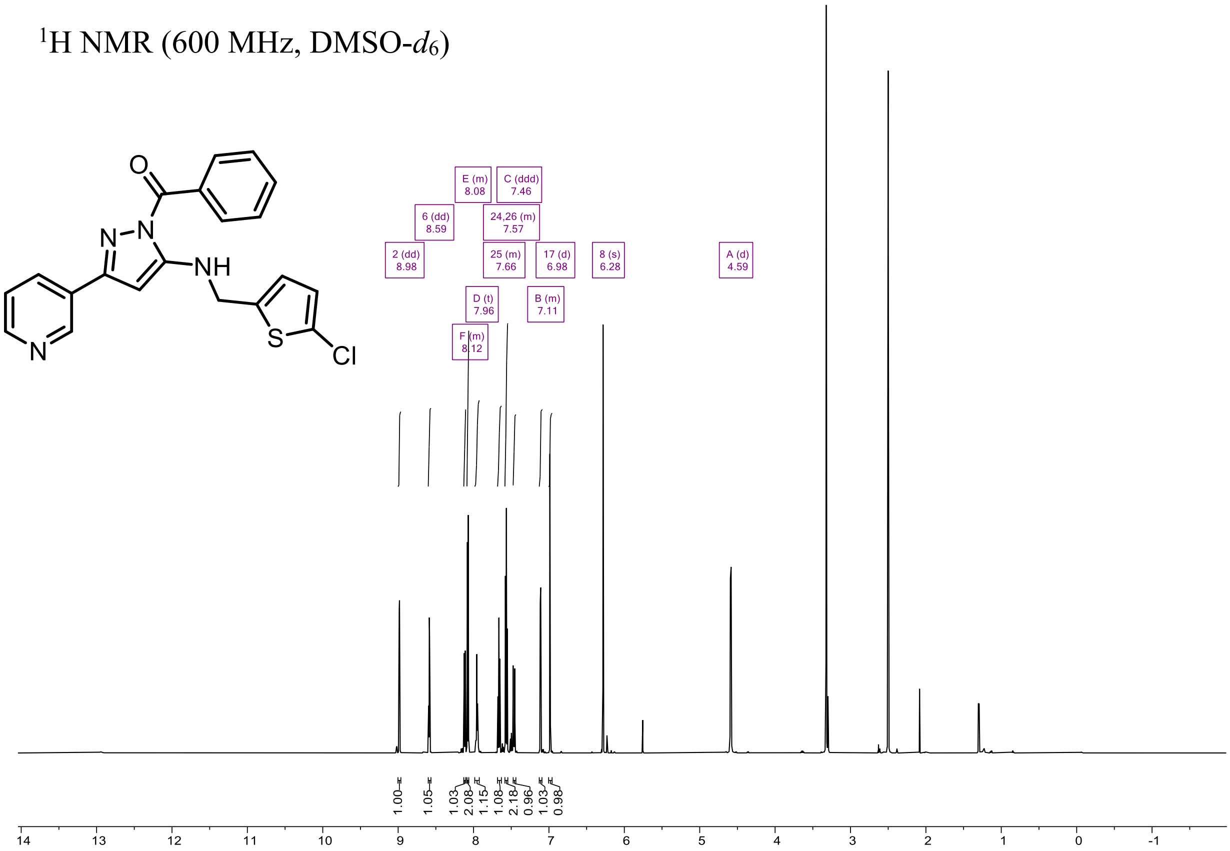

^{13}C NMR (151 MHz, DMSO-d_6)

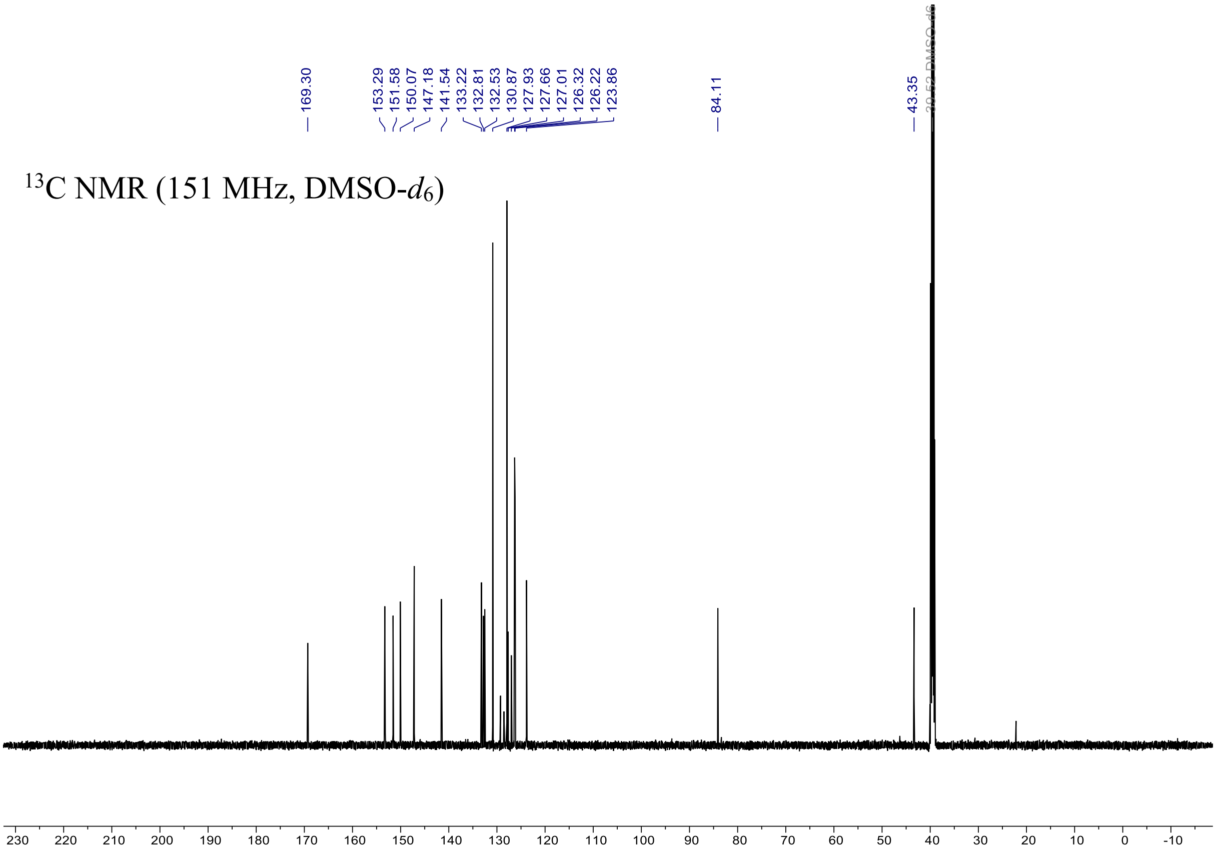

***N*-[3-(pyridin-3-yl)-1*H*-pyrazol-5-yl]benzamide (26a)**

^{1}H NMR (600 MHz, DMSO-d_6)

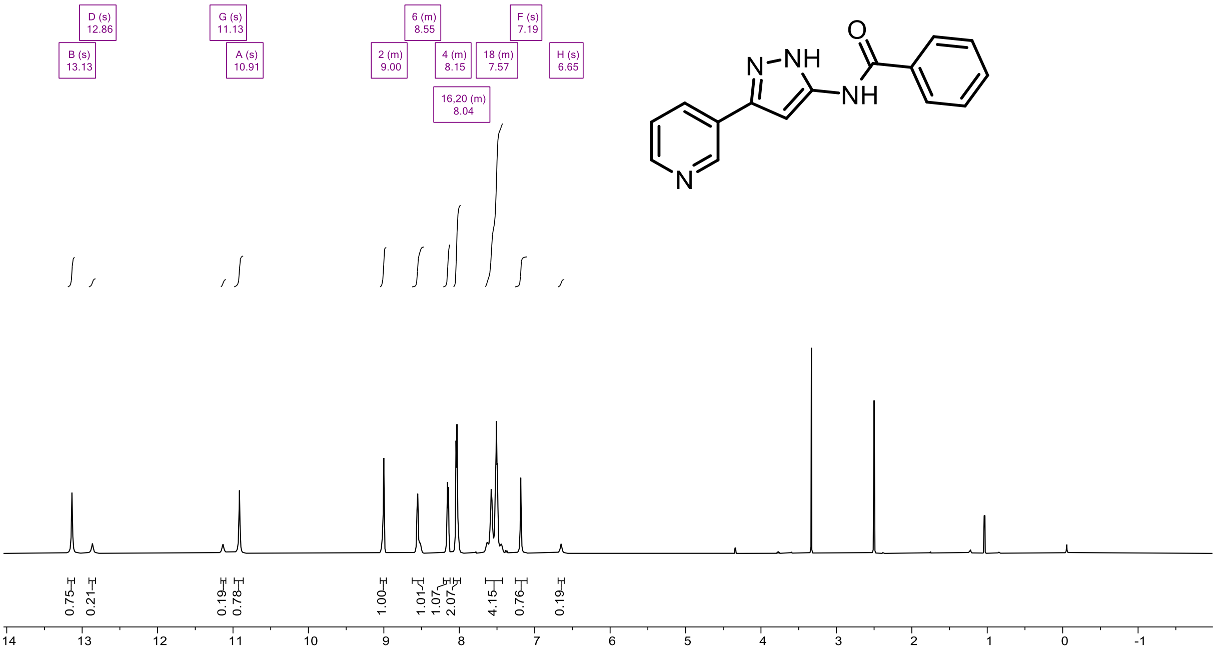

^{13}C NMR (151 MHz, DMSO-d_6)

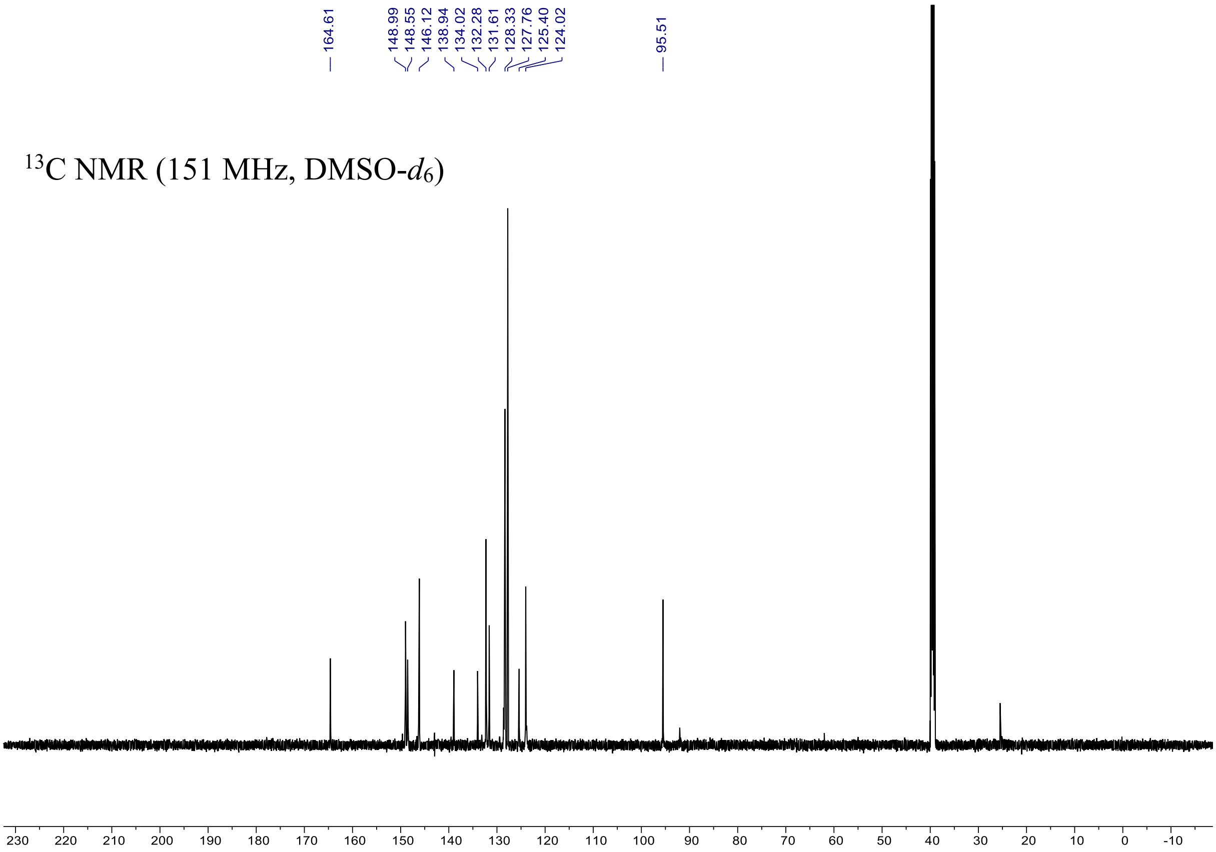

The ratio of tautomers is 8:2.

N-(3-phenyl-1*H*-pyrazol-5-yl)benzamide (26b)

^{1}H NMR (600 MHz, DMSO-d_6)

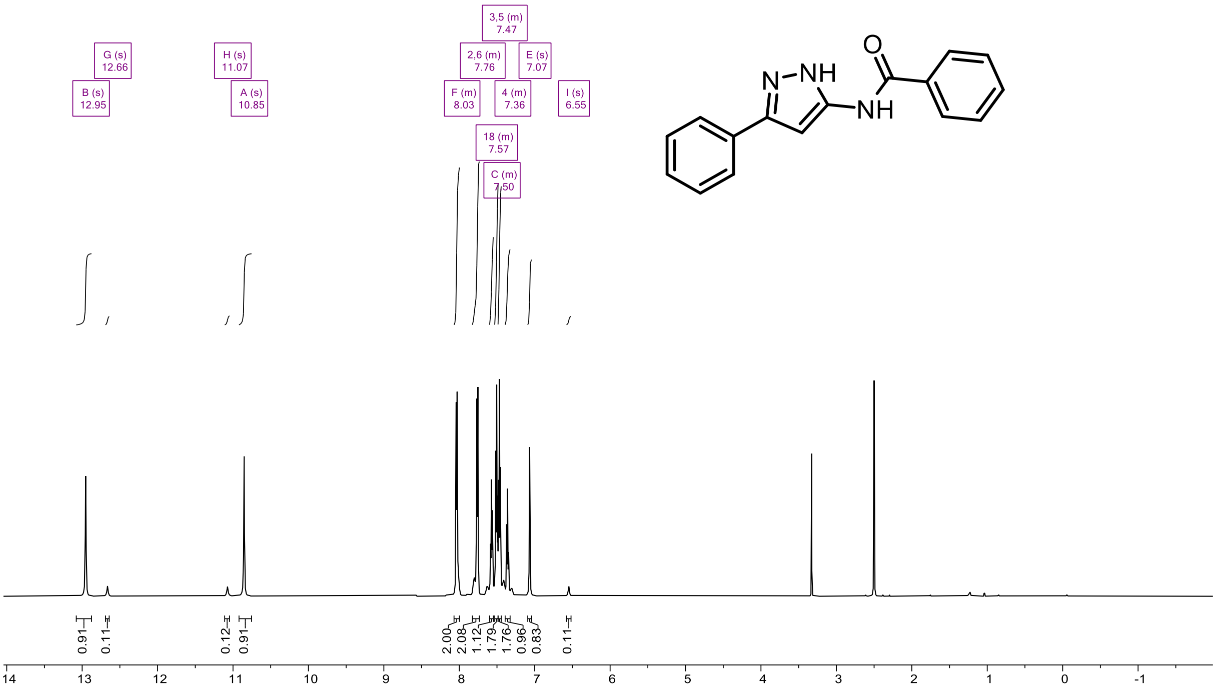

^{13}C NMR (151 MHz, DMSO-d_6)

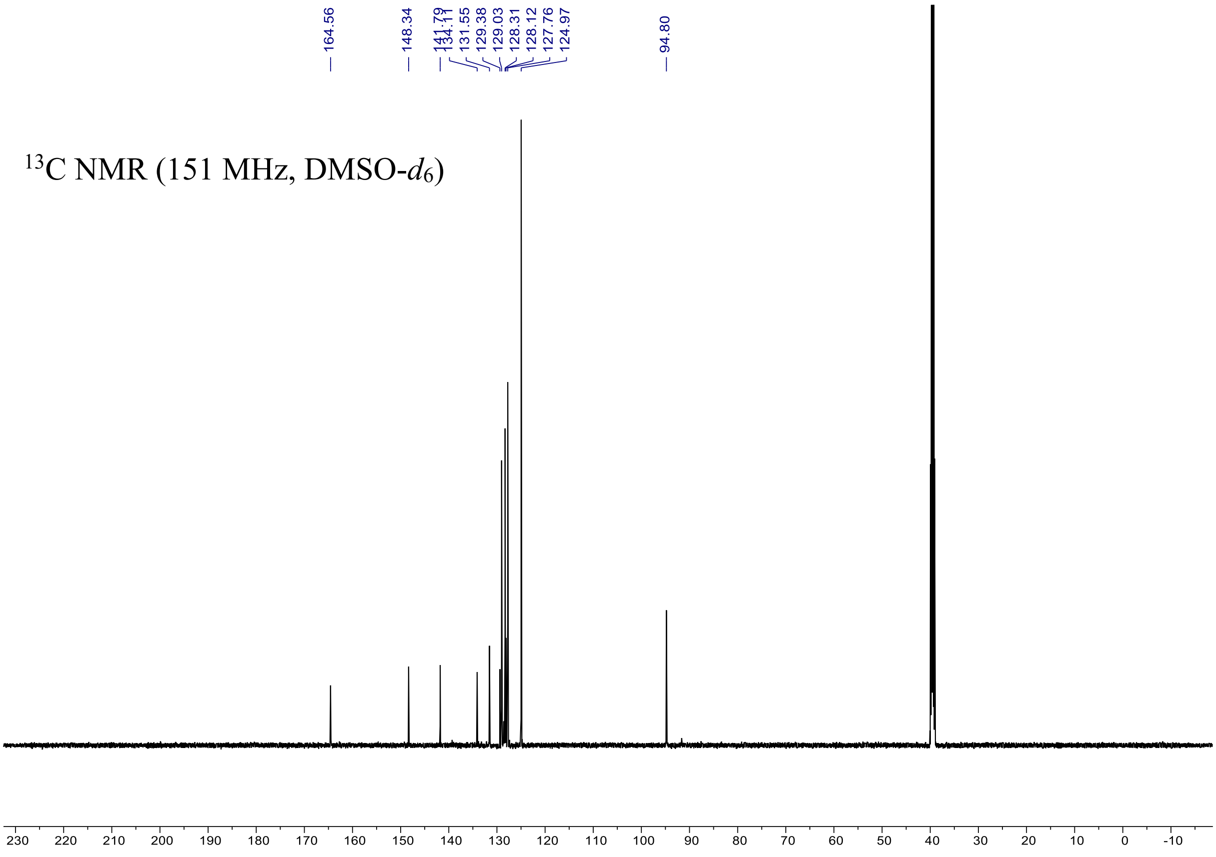

The ratio of tautomers is 9:1.

3-oxo-3-phenylpropanenitrile (28b)

^{1}H NMR (400 MHz, DMSO-d_6)

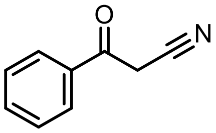

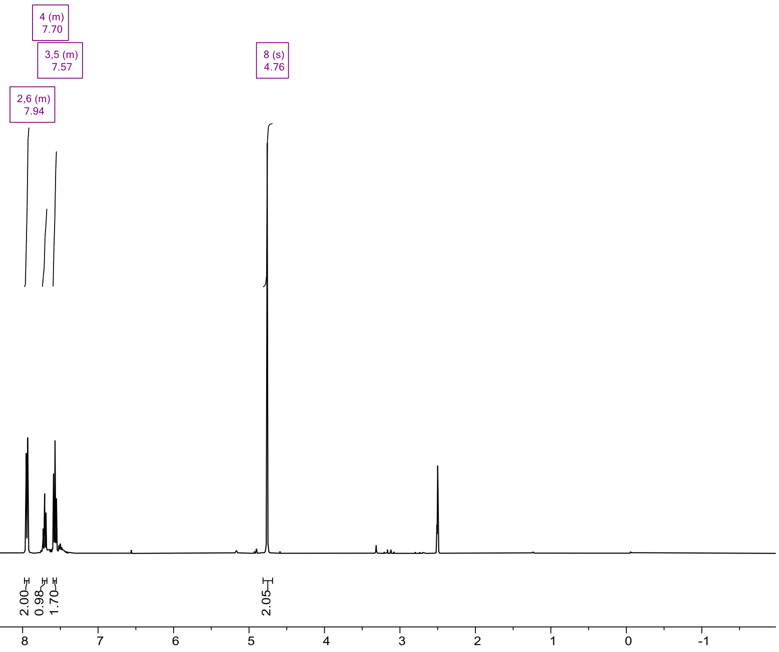

^{13}C NMR (101 MHz, DMSO-d_6)

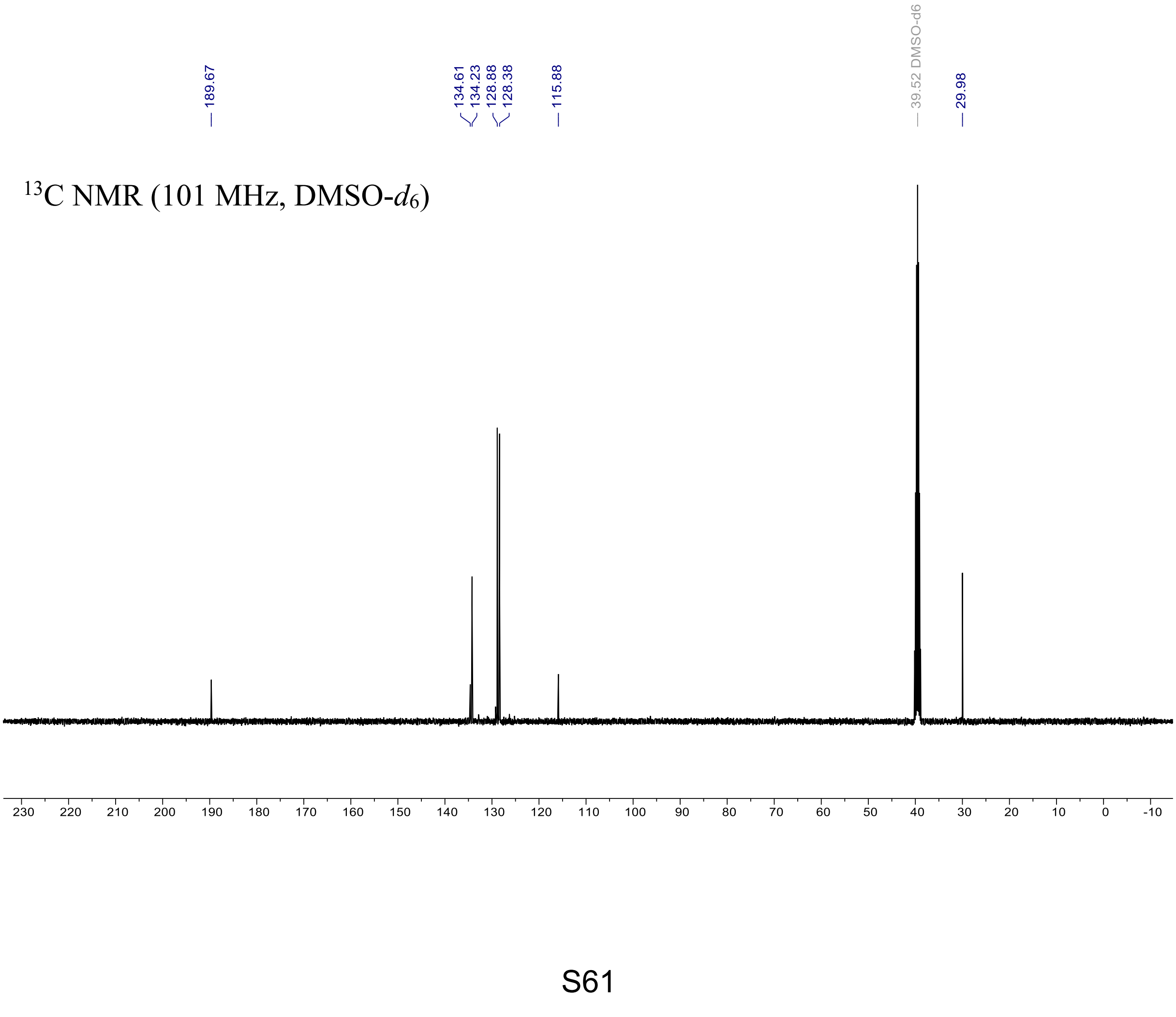

[5-amino-3-(pyridin-3-yl)-1*H*-pyrazol-1-yl](phenyl)methanone (29a)

^{1}H NMR (600 MHz, DMSO-d_6)

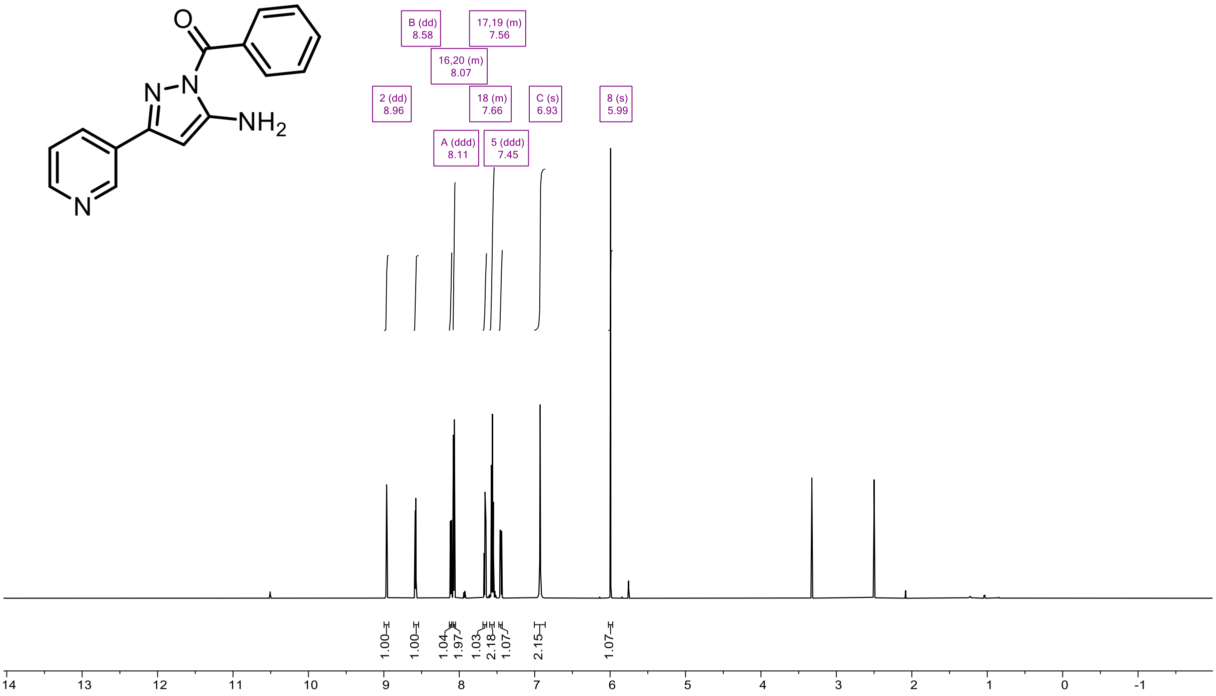

^{13}C NMR (151 MHz, DMSO-d_6)

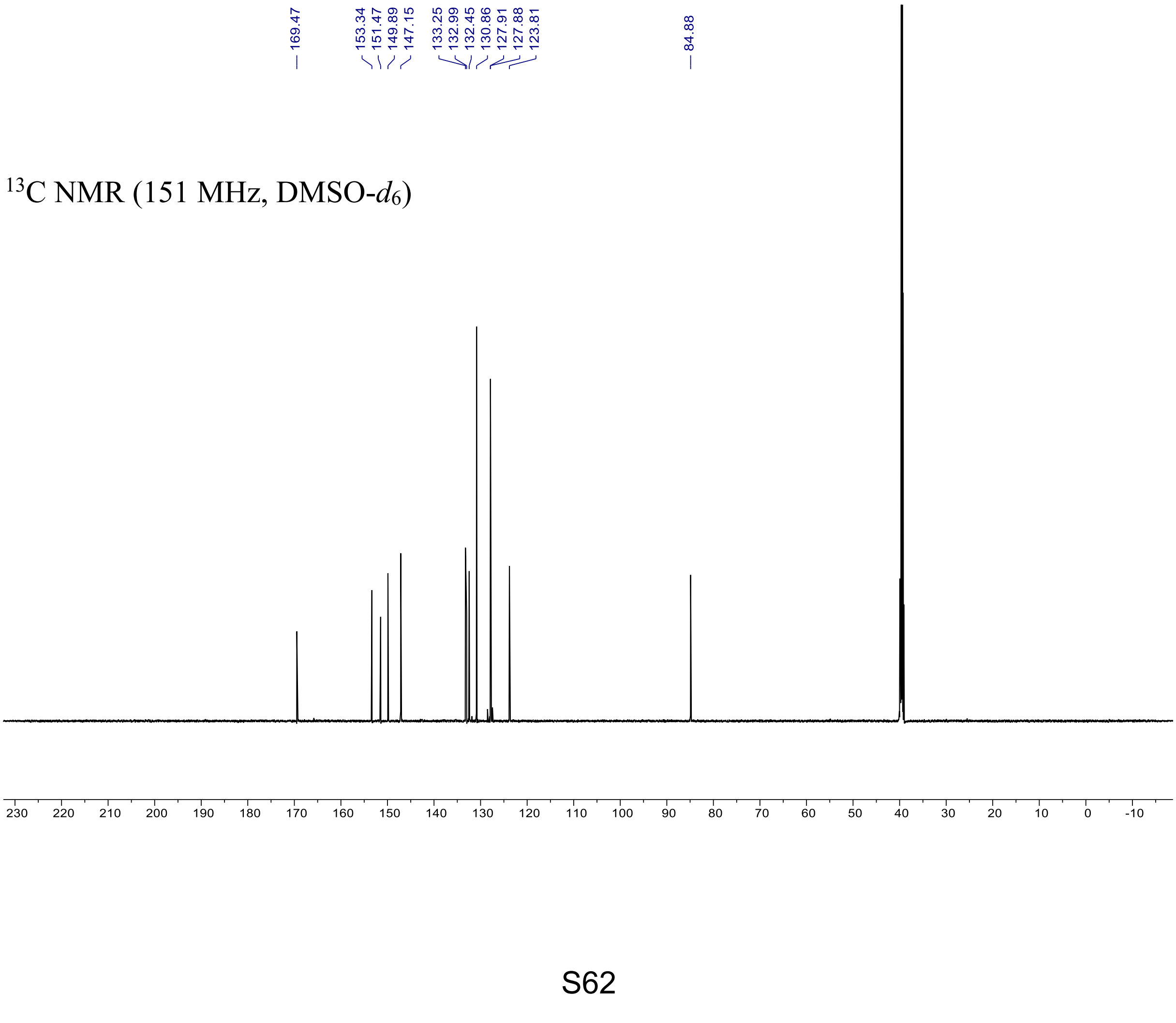

(5-amino-3-phenyl-1*H*-pyrazol-1-yl)(phenyl)methanone (29b)

^{1}H NMR (600 MHz, DMSO-d_6)

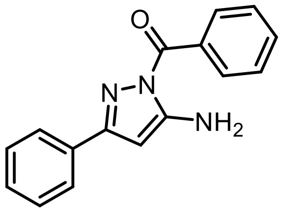

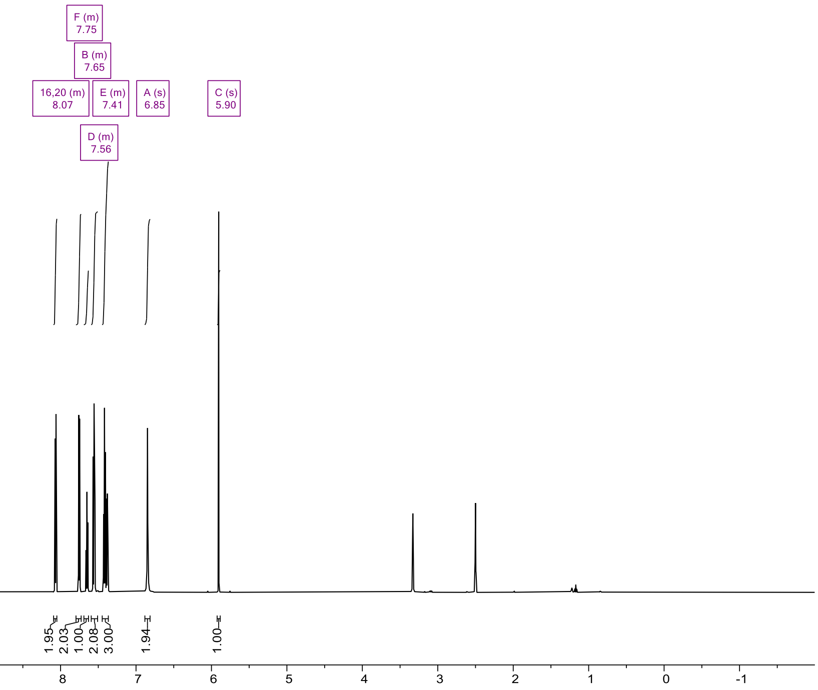

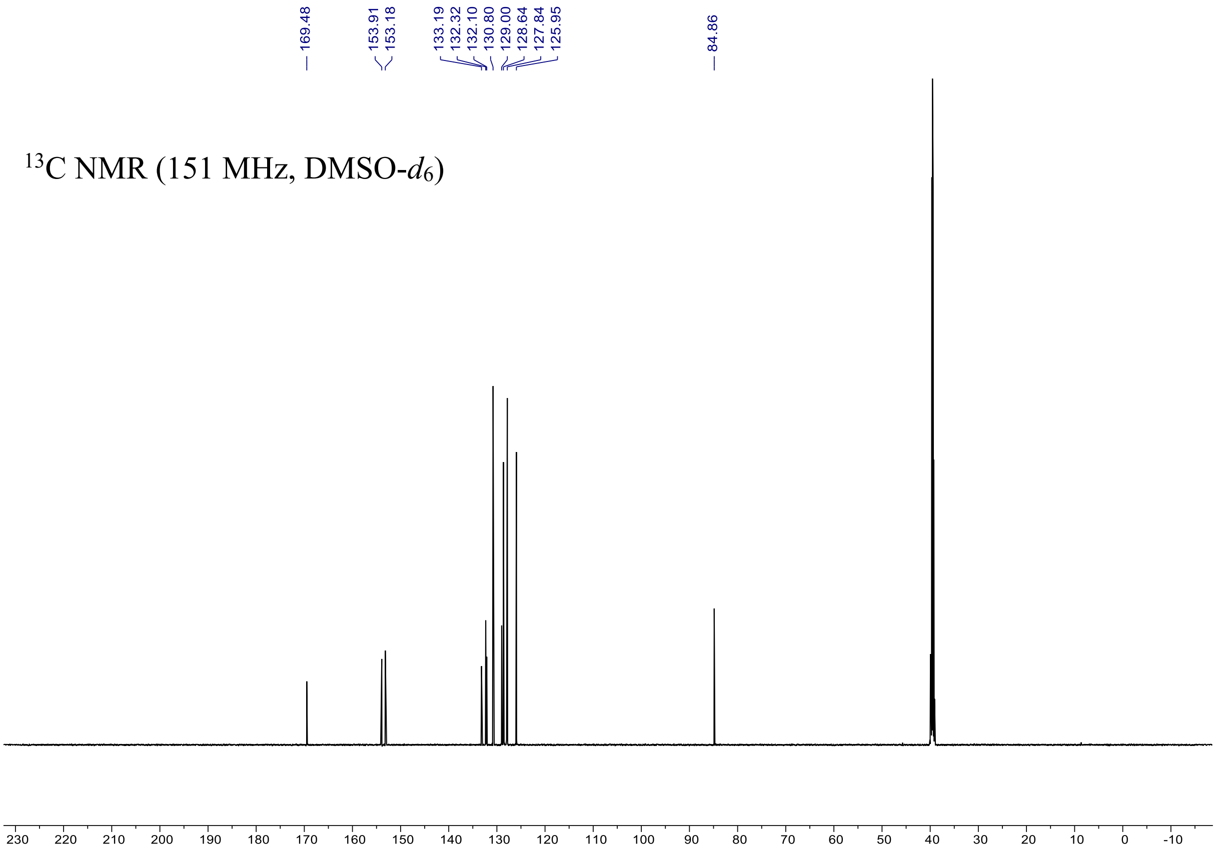

2-fluoro-3-oxo-3-phenylpropanenitrile (31)

^{1}H NMR (400 MHz, $CDCl_3$)

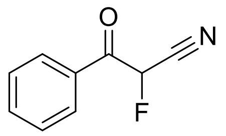

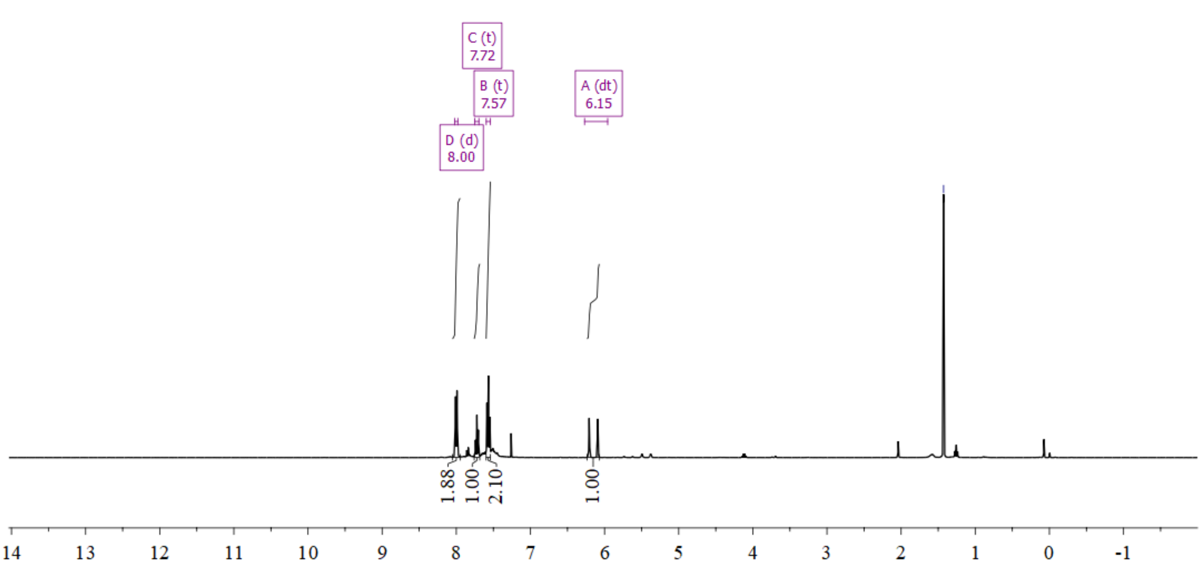

3-amino-4-fluoro-5-phenyl-1*H*-pyrazole (32)

^{1}H NMR (400 MHz, $CDCl_3$)

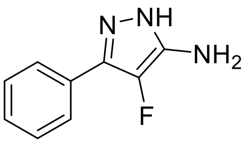

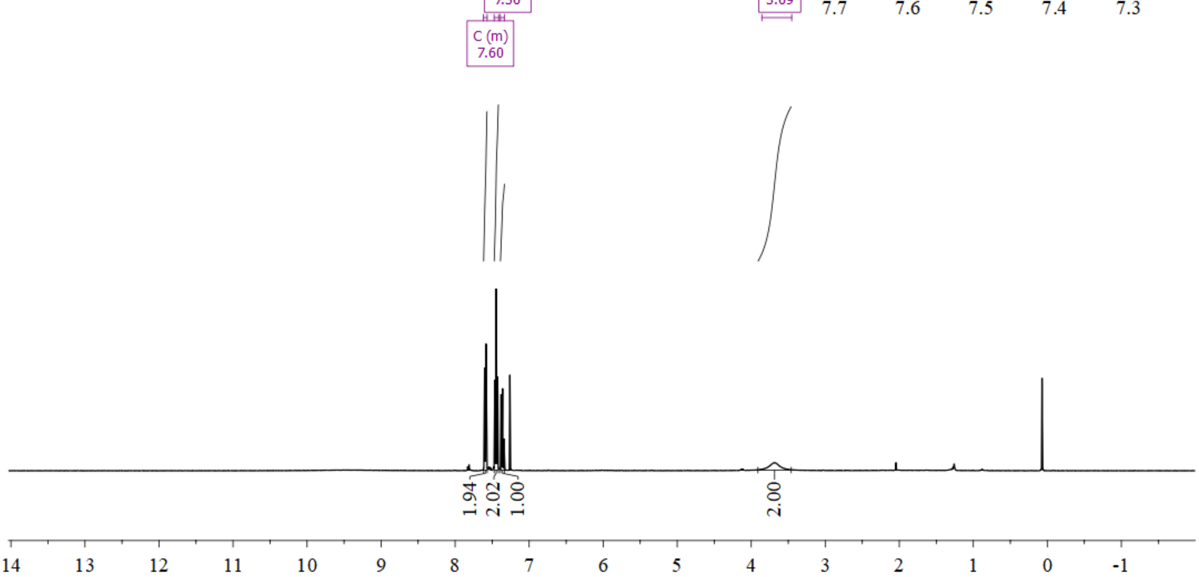

The signal for $NH_{pyrazole}$ is not seen in the spectrum due to tautomerism.

^{13}C NMR (101 MHz, $CDCl_3$)

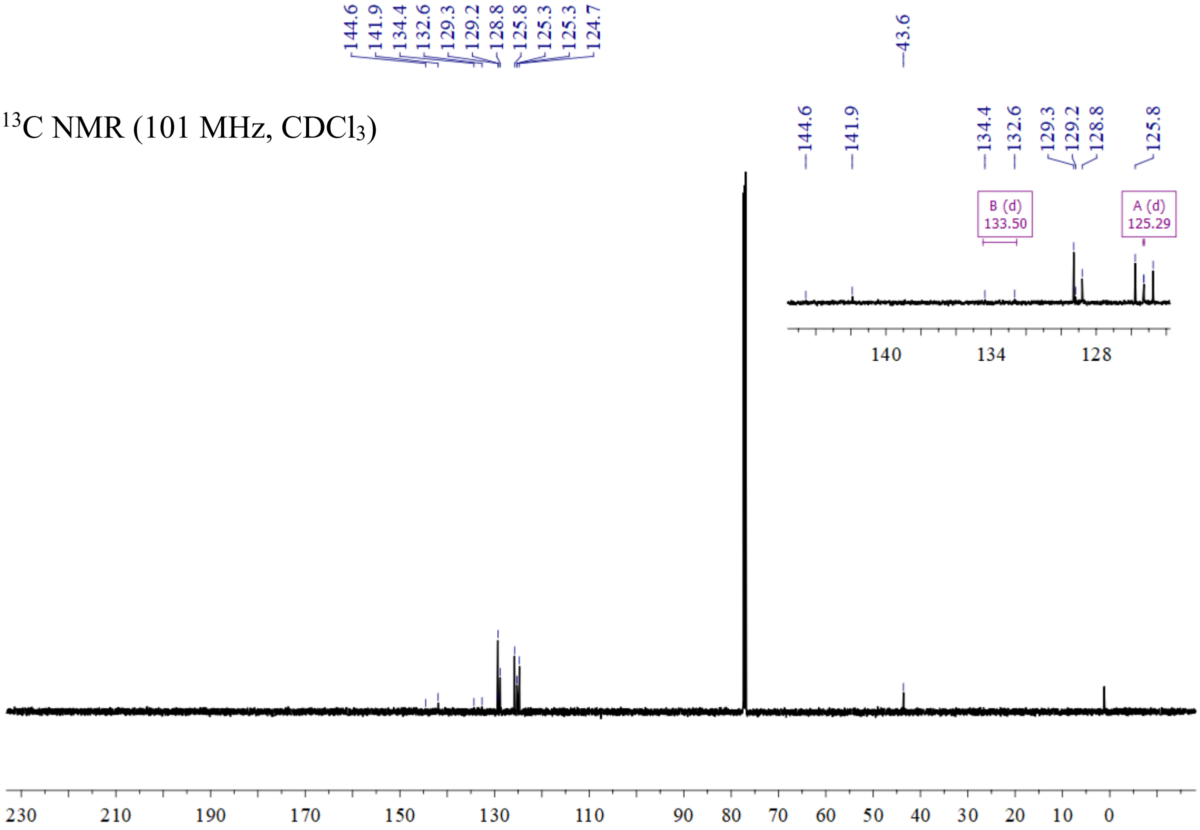

The signal for C-$3_{pyrazole}$ is not seen in the spectrum due to tautomerism.

^{19}F NMR (376 MHz, $CDCl_3$)

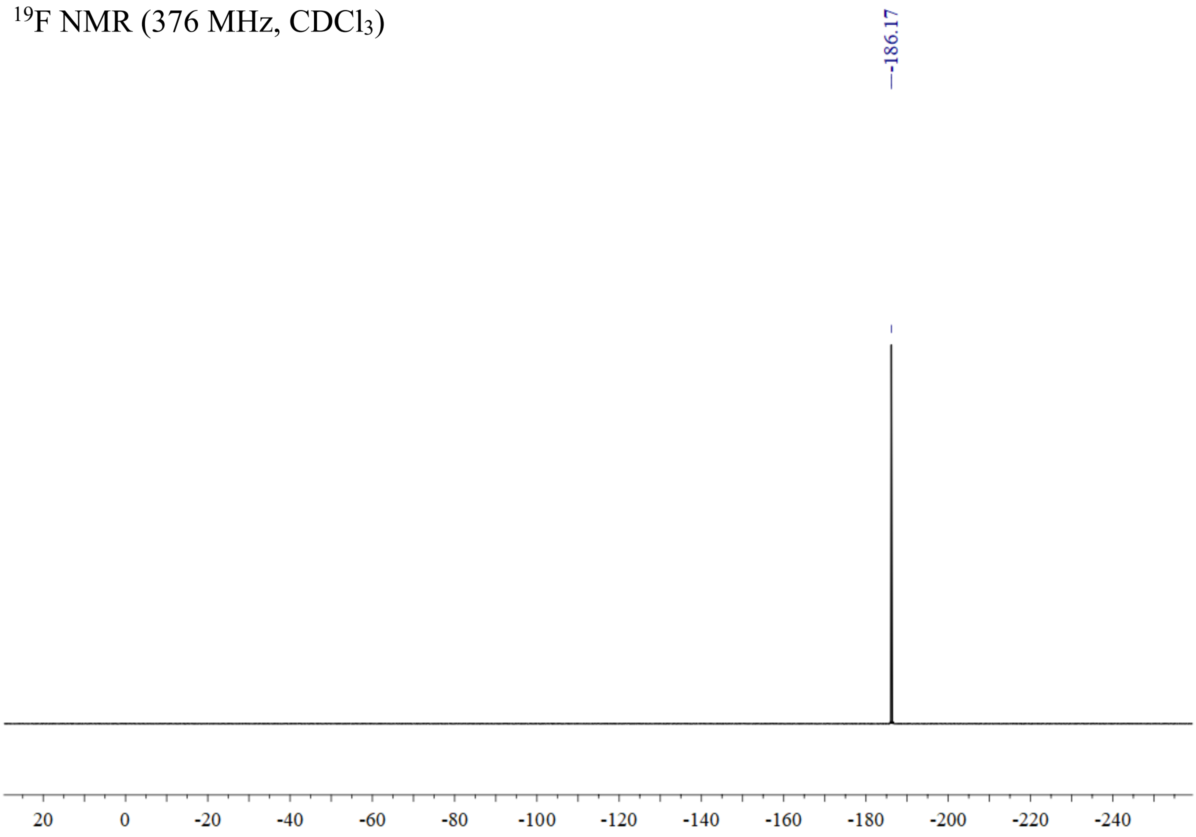

_N_-((5-chlorothiophen-2-yl)methyl)-4-fluoro-5-phenyl-1_H_-pyrazol-3-amine (33)

^{1}H NMR (600 MHz, $CDCl_3$)

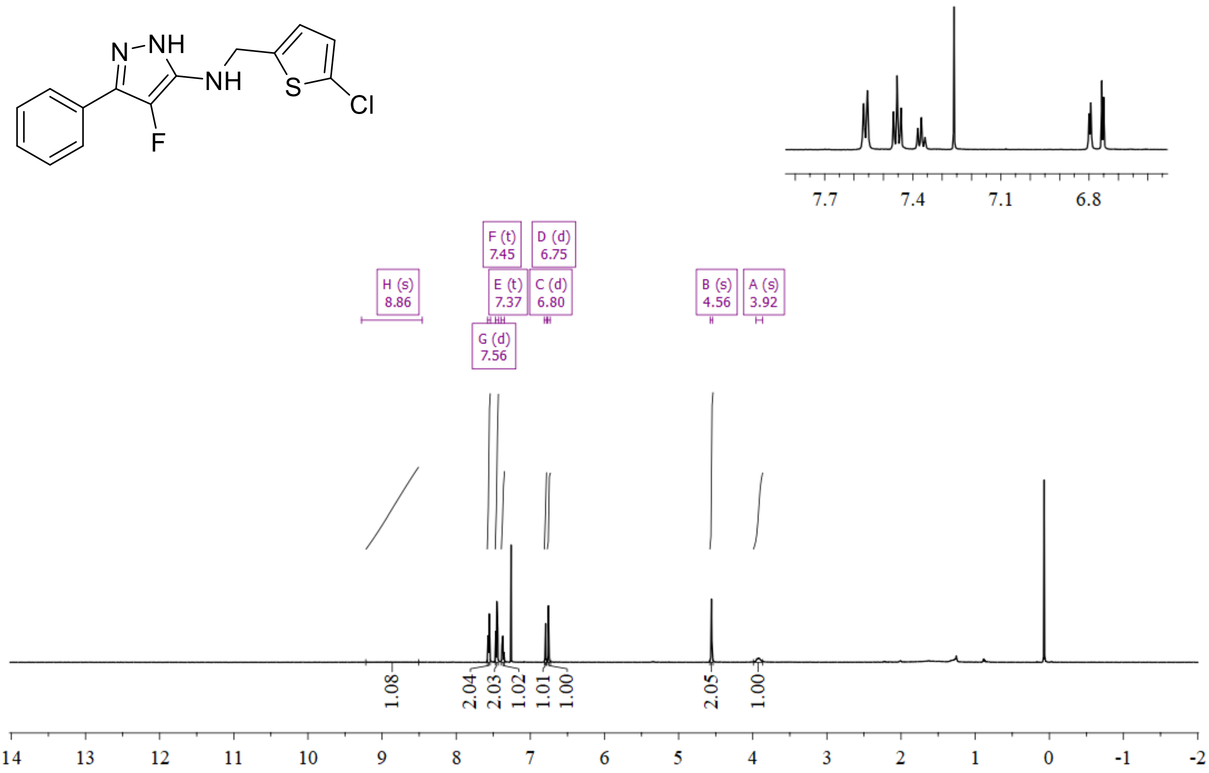

^{13}C NMR (151 MHz, $CDCl_3$)

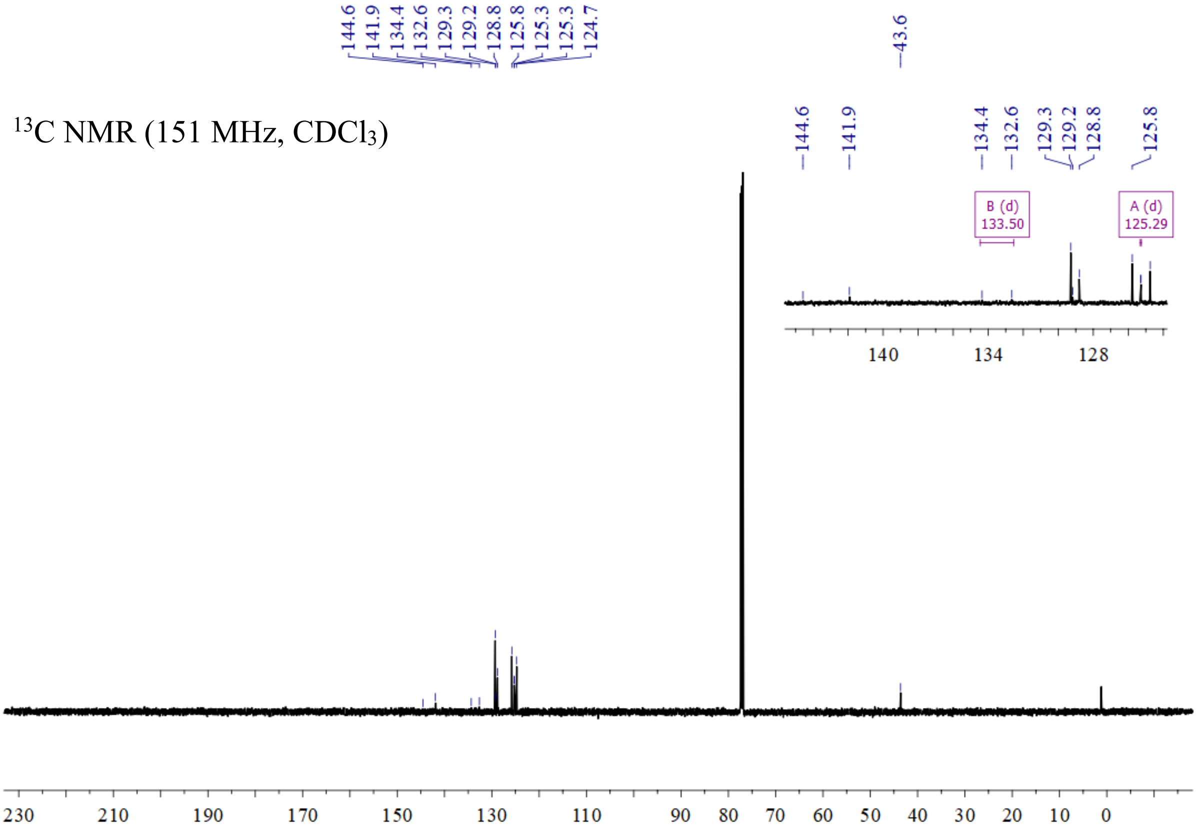

The signal for C-5$_{pyrazole}$ is not seen in the spectrum due to tautomerism.

^{19}F NMR (376 MHz, $CDCl_3$)

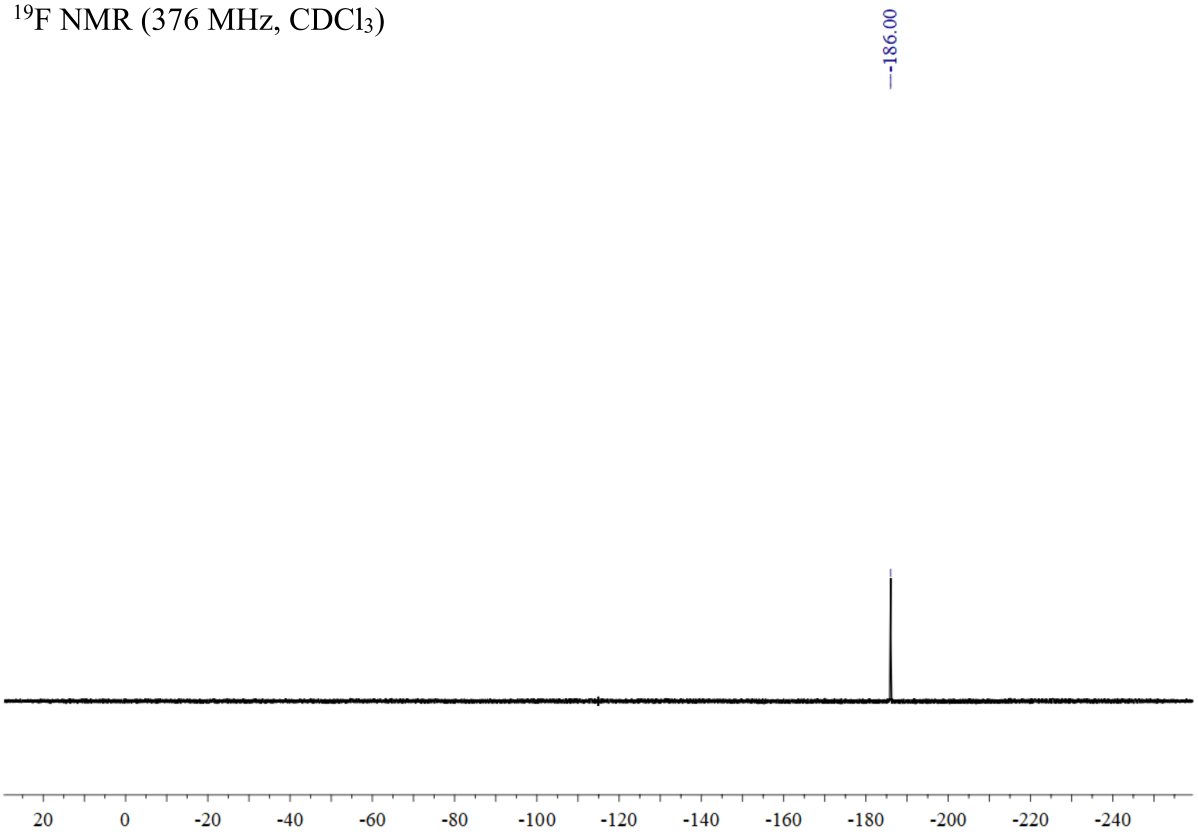

1-(5-(((5-chlorothiophen-2-yl)methyl)amino)-4-fluoro-3-phenyl-1*H*-pyrazol-1-yl)-2,2-dimethylpropan-1-one (34a)

^{1}H NMR (600 MHz, $CDCl_3$)

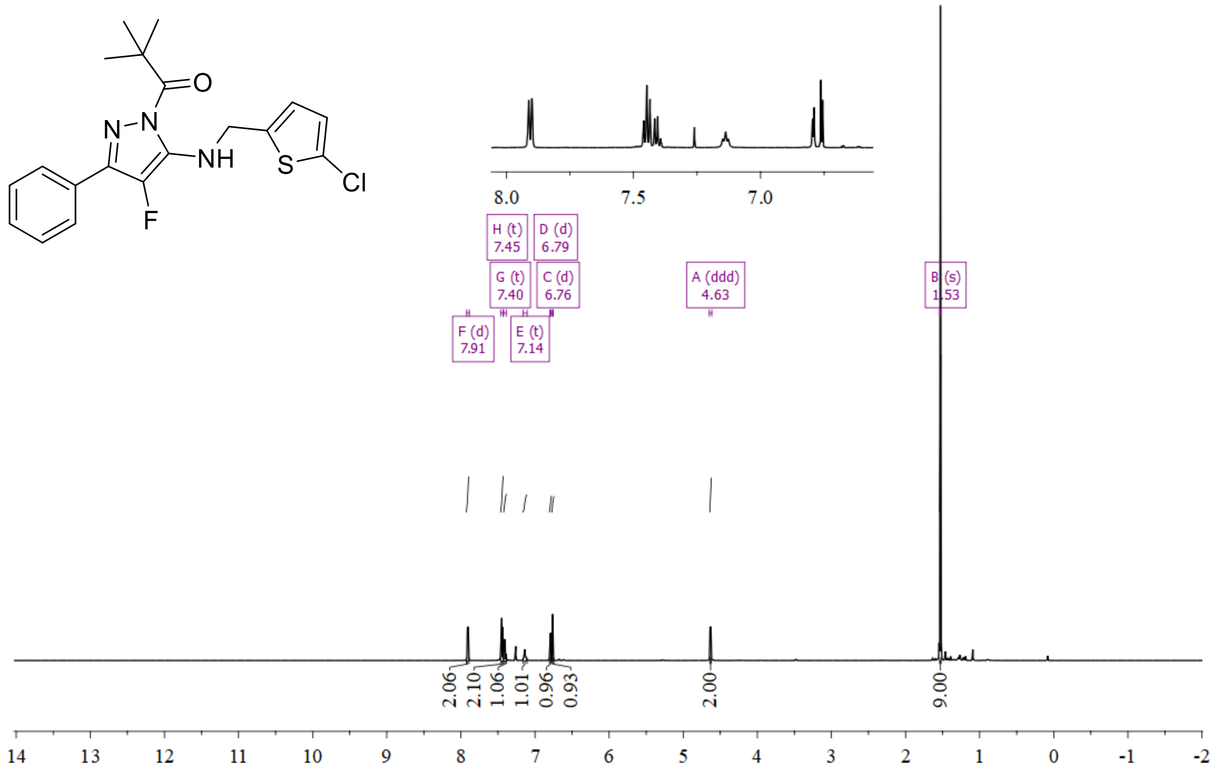

^{13}C NMR (151 MHz, $CDCl_3$)

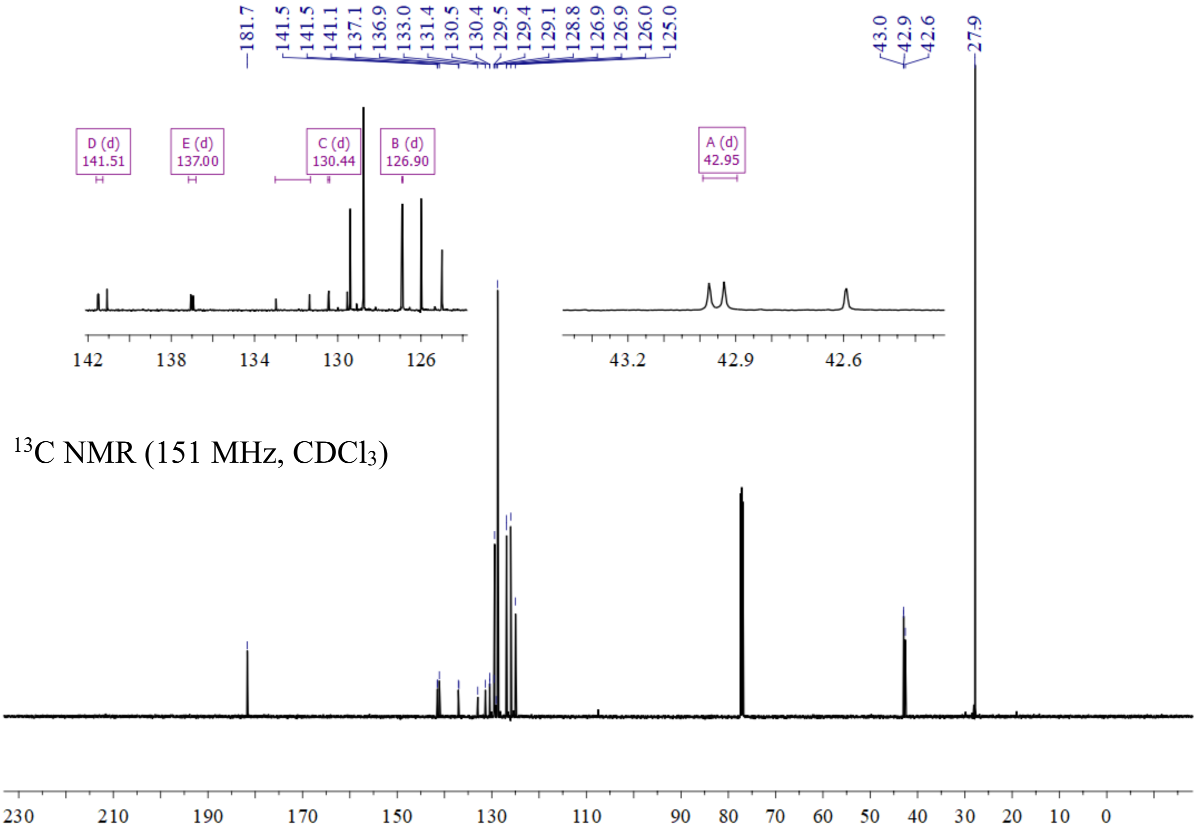

^{19}F NMR (376 MHz, $CDCl_3$)

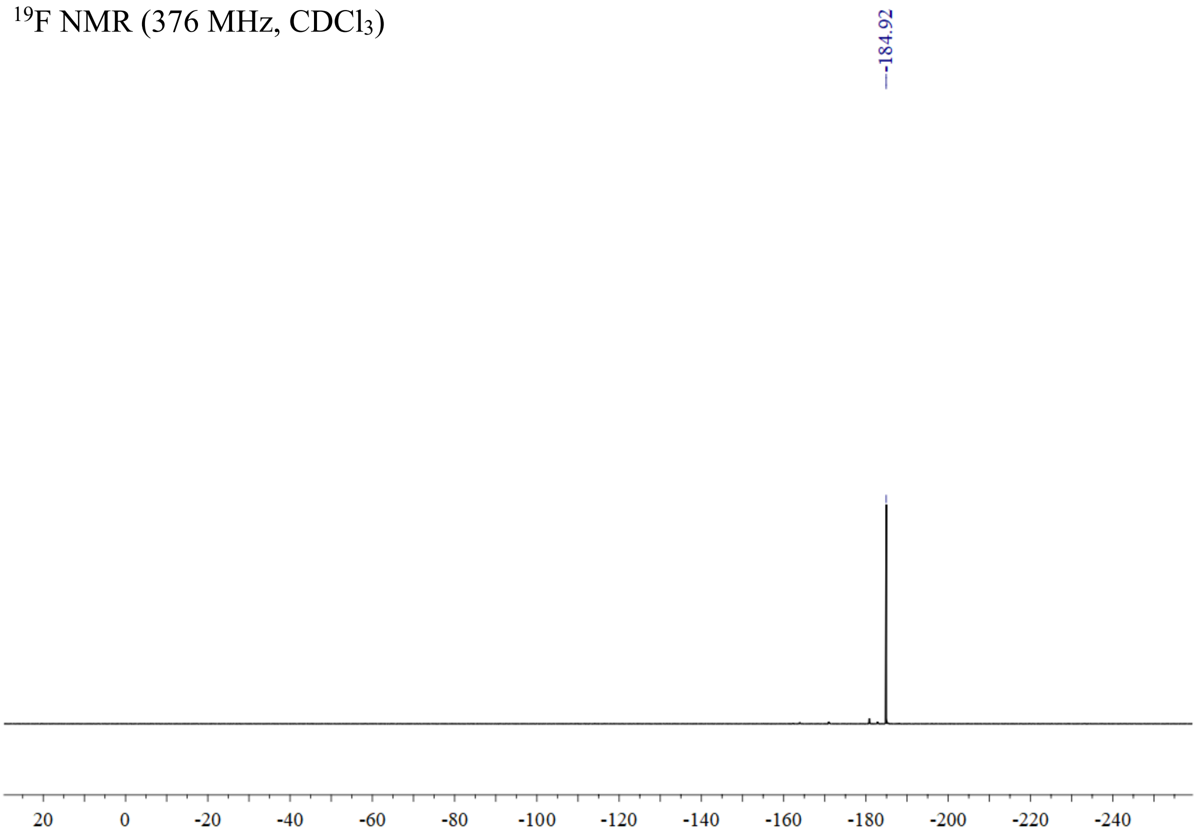

(5-(((5-chlorothiophen-2-yl)methyl)amino)-4-fluoro-3-phenyl-1*H*-pyrazol-1-yl)(phenyl)methanone (34b)

^{1}H NMR (600 MHz, $CDCl_3$)

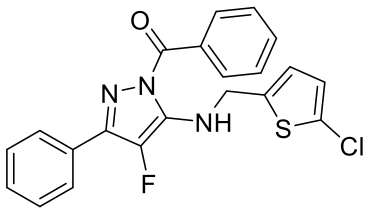

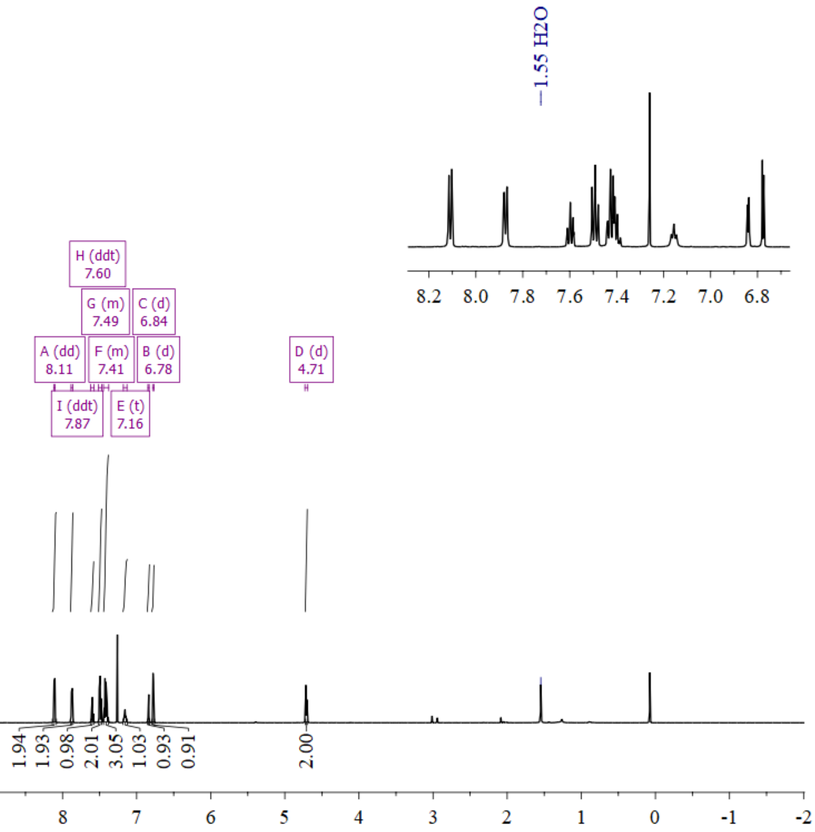

^{13}C NMR (151 MHz, $CDCl_3$)

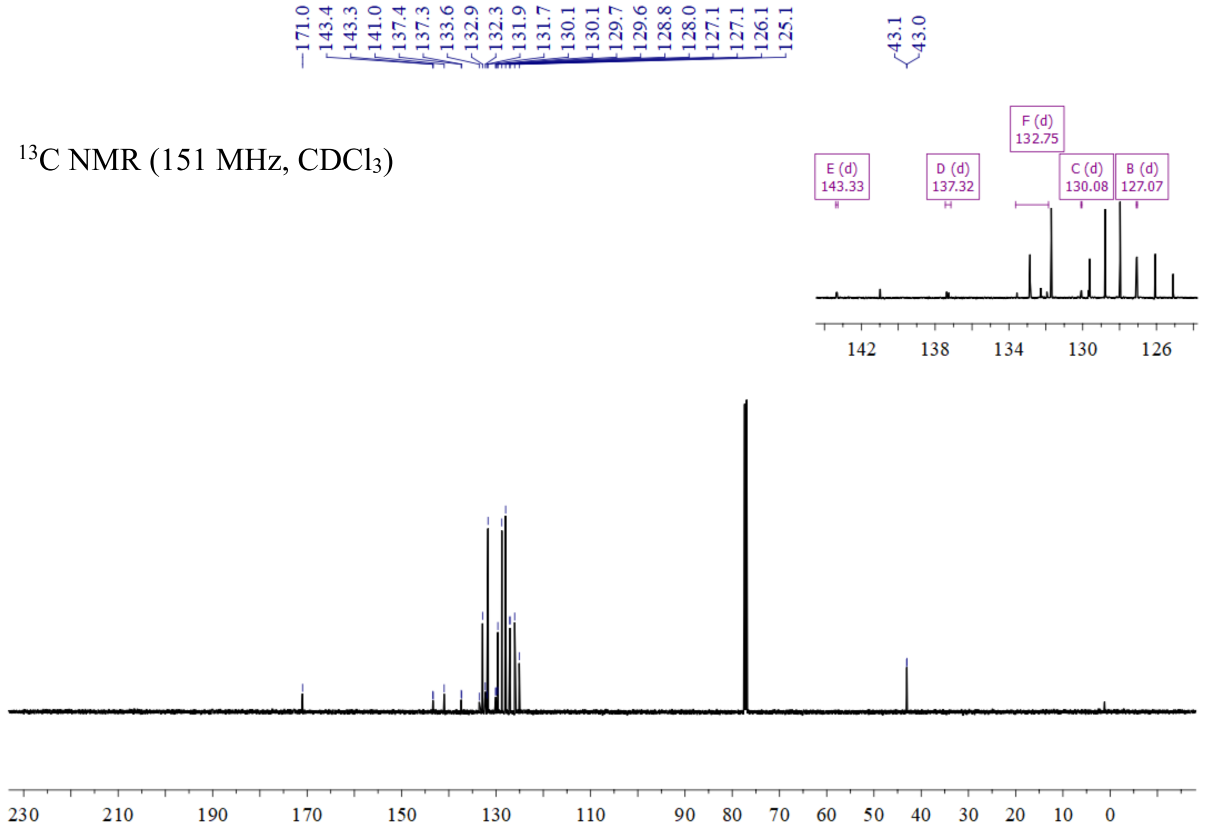

^{19}F NMR (376 MHz, $CDCl_3$)

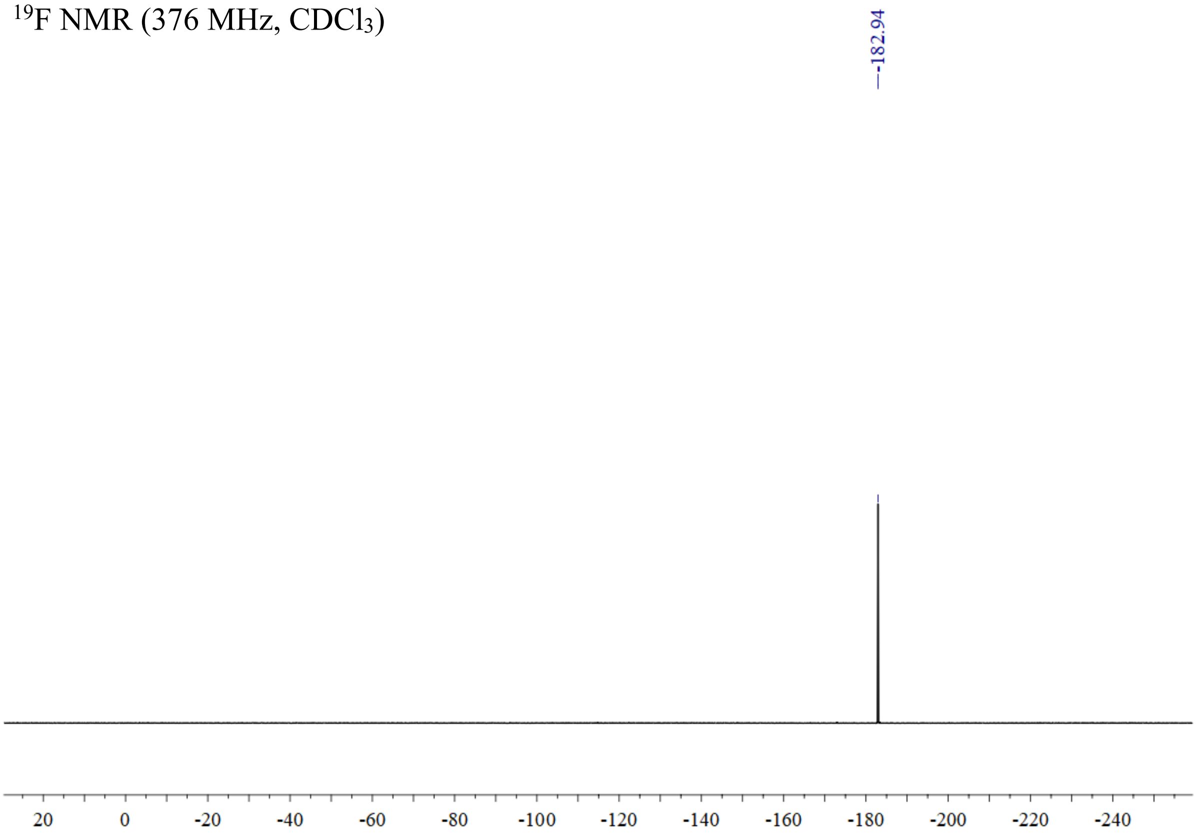

REFERENCES

1. Bruker AXS (**2021**) *APEX4 Version 2021.4-0*, *SAINT Version 8.40B* and *SADABS Bruker AXS area detector scaling and absorption correction Version 2016/2*, Bruker AXS Inc., Madison, Wisconsin, USA.
2. Sheldrick, G. M., *SHELXT – Integrated space-group and crystal-structure determination*, *Acta Cryst.,* **2015**, *A71*, 3-8.
3. Sheldrick, G.M., *Crystal structure refinement with SHELXL*, *Acta Cryst.*, **2015**, *C71 (1)*, 3-8.
4. Bruker AXS (**1998**) *XP – Interactive molecular graphics, Version 5.1,* Bruker AXS Inc., Madison, Wisconsin, USA.
